# What happened after the initial global spread of pandemic human influenza virus A (H1N1)? A population genetics approach

**DOI:** 10.1186/1743-422X-7-196

**Published:** 2010-08-20

**Authors:** Fernando Martinez-Hernandez, Diego Emiliano Jimenez-Gonzalez, Arony Martinez-Flores, Guiehdani Villalobos-Castillejos, Gilberto Vaughan, Simon Kawa-Karasik, Ana Flisser, Pablo Maravilla, Mirza Romero-Valdovinos

**Affiliations:** 1 Departamento de Ecología de Agentes Patogenos, Hospital General "Dr. Manuel Gea Gonzalez", Calzada de Tlalpan 4800, DF 14080, Mexico; 2 Departamanto de Parasitologia Escuela Nacional de Ciencias Biologicas, Prolongación Carpio s/n, Instituto Politecnico Nacional, DF 11340, Mexico; 3 Departamento de Investigaciones Inmunologicas, Instituto de Diagnostico y Referencia Epidemiologicos, Carpio 470 SSA, DF 11340, Mexico; 4 Departamento de Microbiologia y Parasitologia, Facultad de Medicina, Av. Universidad 3000, Universidad Nacional Autonoma de Mexico, DF 04510, Mexico

## Abstract

Viral population evolution dynamics of influenza A is crucial for surveillance and control. In this paper we analyzed viral genetic features during the recent pandemic caused by the new influenza human virus A H1N1, using a conventional population genetics approach based on 4689 hemagglutinin (HA) and neuraminidase (NA) sequences available in GenBank submitted between March and December of 2009. This analysis showed several relevant aspects: a) a scarce initial genetic variability within the viral isolates from some countries that increased along 2009 when influenza was dispersed around the world; b) a worldwide virus polarized behavior identified when comparing paired countries, low differentiation and high gene flow were found in some pairs and high differentiation and moderate or scarce gene flow in others, independently of their geographical closeness, c) lack of positive selection in HA and NA due to increase of the population size of virus variants, d) HA and NA variants spread in a few months all over the world being identified in the same countries in different months along 2009, and e) containment of viral variants in Mexico at the beginning of the outbreak, probably due to the control measures applied by the government.

## Findings

In April 2009 the Mexican Secretariat of Health reported an outbreak of respiratory disease. A new human influenza virus A H1N1 with molecular features of North American and Eurasian swine, avian, and human influenza viruses was identified [1]. In the same month, the World Health Organization (WHO) classified the global spread of this virus as a public health event of international concern. After documentation of human to human transmission of the virus in at least two WHO regions, the highest pandemic level was declared [2]. As a result of the epidemiological surveillance, large amounts of A H1N1 genetic sequences were accumulated in the GenBank and several molecular epidemiological studies monitoring evolutionary inferences of viral gene flow in time and space were reported [3-6]. In December 2009, A H1N1 was worldwide spread, affecting 208 countries, with at least 12,220 deaths [7]. Thus, more sequences were reported but no overall population genetics studies were performed, and also no comparison of the initial and the viral variants (VV) has been reported. The goal of the present study is to provide an overview with a phylogeographic behavior during the initial spread and subsequent worldwide establishment of influenza pandemic.

Analysis of genetic diversity within and between populations were calculated using DnaSP v4 [8-10] and included nucleotide diversity (π), haplotype polymorphism (θ), genetic differentiation index (G_ST_), coancestry coefficient (F_ST_) and migration (Nm). These indexes refer to: π, average proportion of nucleotide differences between all possible pairs of sequences in the sample; θ, proportion of nucleotide sites that are expected to be polymorphic in any suitable sample from this region of the genome. Both indexes are used to assess polymorphisms at the DNA level and monitor diversity within or between ecological populations, and examine the genetic variation in related species or their evolutionary relationships [9]. F_ST _and G_ST _are two equivalent genetic statistics used to measure differentiation between or among populations; F_ST _is used when there are only two alleles at a locus, and G_ST _with multiple alleles; common used values for genetic differentiation are: 0 to 0.5 small; 0.05 to 0.15 moderate; 0.15 to 0.25, great, and values above 0.25 indicate huge genetic differentiation, while negative values are due to small sample size [8] and thus, when found, zero value was assigned [11,12]. The gene flow or migration index (Nm) refers to movement of organisms among subpopulations, those strongly differentiated have a Nm < < 1, while Nm > 4 behave as a single panmictic unit [9].

The previously described genetic diversity analyses were performed with A H1N1 Influenza Database [13] with sequences submitted between April and December 2009 (collection dates and sequence origin are found in addition file 1), including three or more sequences per country of 500 continuous base pairs (bp), recorded during the initial four months of the pandemics and, for the global analysis, those having at least 750 continuous bp were used. Multiple alignments were performed by CLUSTAL W program v1.8 [14] and adjusted using MEGA program v4 [15,16]. A median joining method for constructing networks from recombination-free population data, featuring Kruskal's algorithm for finding minimum spanning trees [17] was used with the program Network 4v.5.1.6 [18].

**Figure 1:**
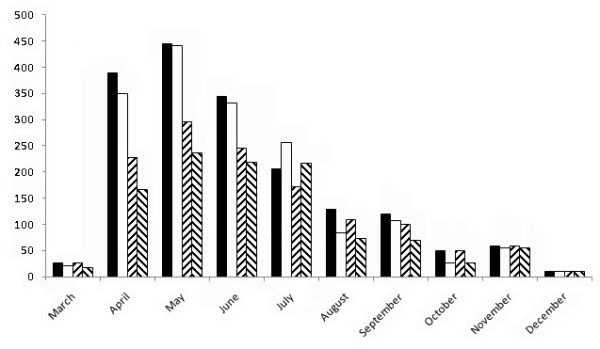
**Number of sequences and influenza variants of HA and NA identified monthly along 2009**. Full bars correspond to HA sequences, empty bars to NA sequences; left dash bars to HA variants and right dash bars to NA variants.

**Figure 2:**
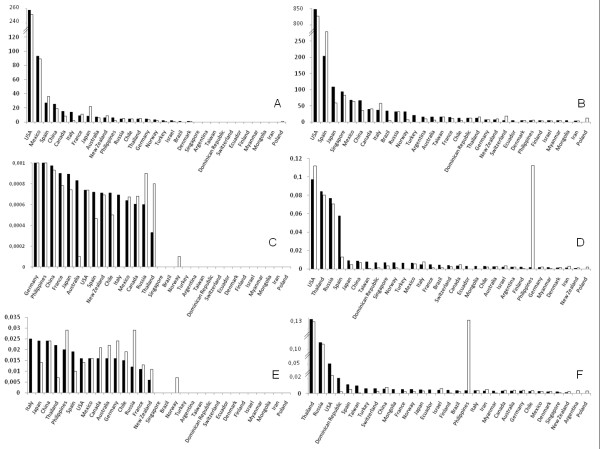
**Number of influenza sequences of HA (full bars) and NA (empty bars) reported during the initial four months (2A) and for the global analysis (2B), θ values found for the same sequences and periods are seen in figures 2C and 2D, while π values are in figures 2E and 2F**.

Up to 3462 sequences (1779 of HA and 1683 of NA) with 2208 VV (1216 of HA and 992 of NA) from 31 countries were used, interestingly 80% were recorded between April and July (Figure 1). Figure 2 shows the number of sequences analyzed (first row), θ values (second row) and π values (third row) for the analysis performed of the sequences obtained in the initial four months (left column) or of the global analysis (right column). As it can be seen few countries provided most variants. Theta and Pi showed a similar high trend in around 50% of the countries in the analysis of the initial four months (average π = 0.0025 for HA and π = 0.0016 for NA)). In contrast, the overall analysis shows that polymorphism increased in all the countries (π = 0.0125 for HA and π = 0.0153 for NA), with higher levels for USA, Russia, Thailand, Philippines and Spain.

**Figure 3:**
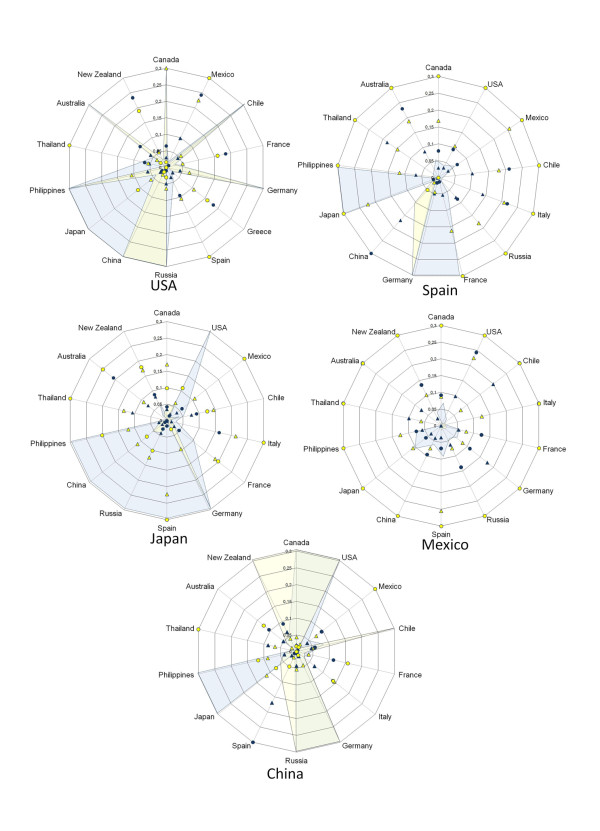
**Radial plots of countries with HA and NA reported along the first four months (April-July, 2009) of the pandemic show population genetic indexes from countries that reported the higher number of influenza sequences paired against all those countries with A H1N1**. Yellow and blue areas correspond to gene flow (Nm × 10^2^) for HA and NA respectively; triangles correspond to F_ST _values, full for HA and empty for NA; circles correspond to G_ST _values, full for HA and empty for NA. In order to facilitate viewing all values above 3 they are seen as 3.

**Figure 4:**
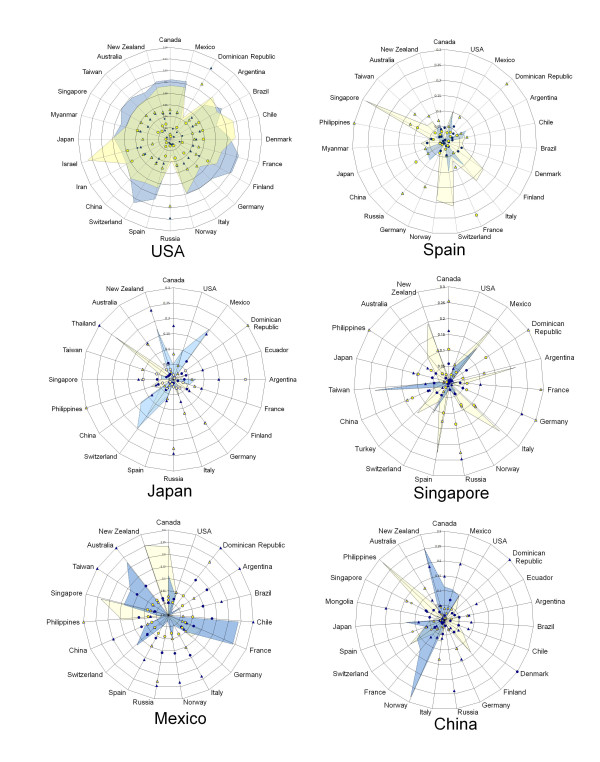
**Radial plots of countries with HA and NA reported between April and December 2009 show population genetic indexes from countries that reported the higher number of influenza sequences paired against all those with A H1N1**. Yellow and blue areas correspond to gene flow (Nm × 10^2^) for HA and NA respectively; triangles correspond to F_ST _values, full for HA and empty for NA; circles correspond to G_ST _values, full for HA and empty for NA. In order to facilitate viewing all values 3 or above are seen as 3.

Genetic population indexes were compared in the countries with most sequences reported (USA, Spain, Japan, Mexico and China). Figure 3 shows, in five plots, the data of these countries paired against all those countries with HA and NA reported during the initial four months of the pandemic. For example in USA it can be seen that genetic differentiation parameters (F_ST _and G_ST_) were high when this country was paired with Mexico, France, Greece or New Zealand (seen as full or empty dots or triangles), while the values of genetic flow (Nm) were higher when USA was compared to Chile, Germany, Russia, China, Philippines or Australia (seen as shadowed areas or star peaks). Following the same explanation for the other four countries, it can be seen that some showed high or low degree of differentiation for F_ST _and G_ST _but opposed for Nm. Thus, the highest flow is seen in USA followed by Japan, China and Spain, and the lowest was found in Mexico. Interestingly, in the image obtained when samples from April-December were used, a different pattern can be seen: USA shows a moderate flow with all countries used for comparison; while Mexico is the country with the highest differentiation. The in-between countries are Japan, China, Spain and Singapore; the latter country appears in figure 4 but not in 3 because there are no data reported for the early months. Additional file 2 includes all data obtained for F_ST_, G_ST _and Nm. Negative values for F_ST _and G_ST _indicate no differentiation; in some cases NA showed lower F_ST _values that those of HA with a similar trend. Tajima's D provided negative values: -2.619 and -2.380 in the initial four months and -1.802 and -2.358 in the overall analysis, for HA and NA, respectively, indicating arousal of new polymorphisms as a consequence of population size expansion along 2009 [9].

**Figure 5:**
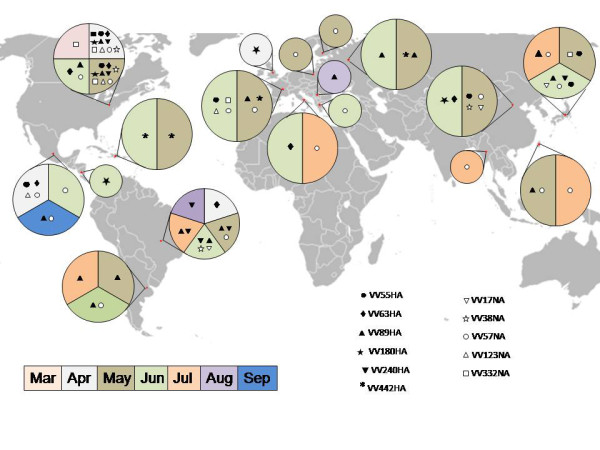
**World map showing HA and NA influenza variants found in more than three countries along the study**. Full geometric figures correspond to HA sequences; empty to NA.

Figure 5 shows the widespread distribution of the main HA and NA VV around the world and along the time; for example, VV57NA was identified in USA and Mexico in April; one month later it was also present in Brazil, France, Poland, Finland, China and Taiwan; in June in Chile, Greece and Japan; and in July also in Italy and Myanmar (see also additional file TS2).

**Figure 6:**
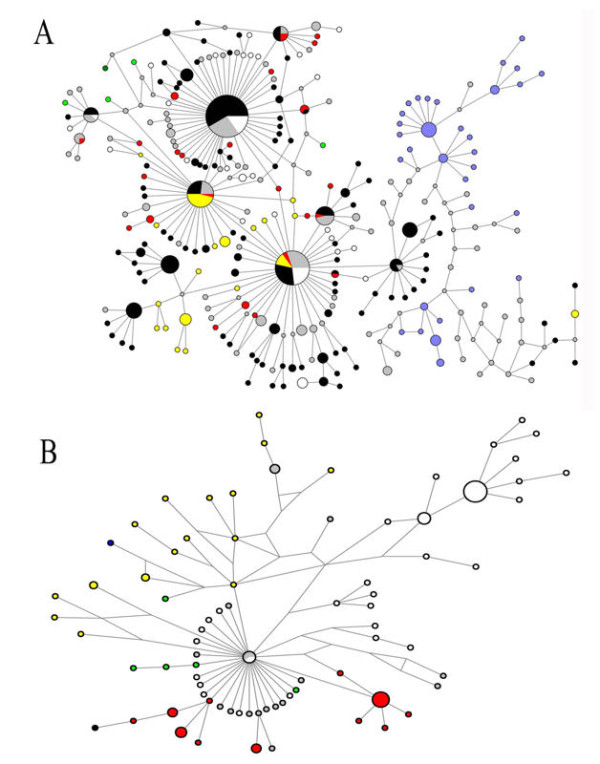
**Median joining network showing the HA variants identified during the first four months (A) or from July to December (B)**.The sizes of circles represent the frequency of VV. In black variants from USA, blue Spain, white Japan, green Singapore, yellow Mexico, red China and grey from other countries.

**Figure 7:**
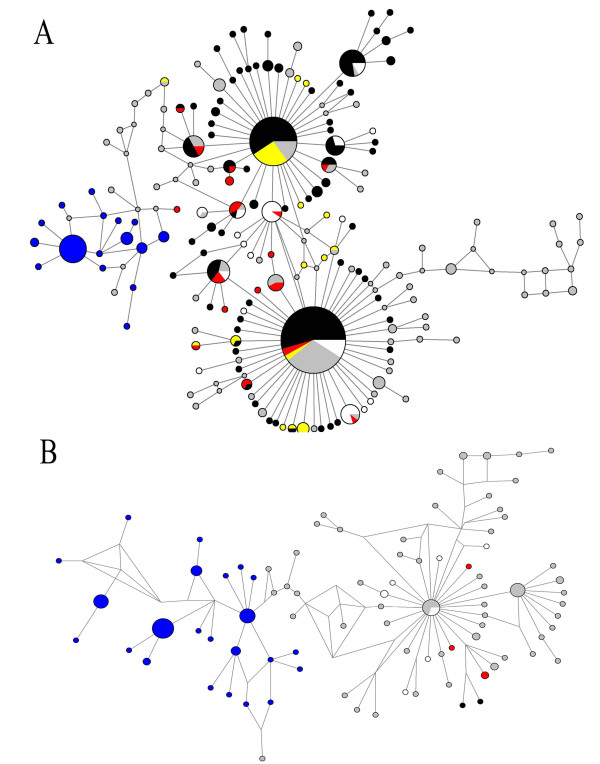
**Median joining network showing the NA variants identified during the first four months (A) or from July to December (B)**.The sizes of circles represent the frequency of VV. In black variants from USA, blue Spain, white Japan, green Singapore, yellow Mexico, red China and grey from other countries.

Figures 6 and 7 show the networks obtained for HA and NA during the first and the last four months (A and B respectively), with the Median Joining method that estimates genealogic relationships. Figure 6A shows three major dispersion centers for HA: one that clustered variants from USA and Asia, a second one that grouped VV mainly from USA, Mexico and China and the third with several Spanish variants. Using NA sequences (Figure 7A) two principal dispersion centers were identified: one clustering mainly VV form USA and another one that grouped VV form USA, Mexico and China; similarly to HA, several Spanish VV were dispersed. Networks obtained between July and December showed only one dispersion center, with several VV from Mexico, China and Singapore in the HA tree, as seen in figure 6B and numerous separated Spanish VV in the NA tree (Figure 7B).

Our study shows that a high viral diversity during the 2009 pandemic took place, as compared, for example, to a study of HA performed in 1999-2000 with samples from French infected patients with A/H3N2, which showed an average of π = 0.0034 [19] which is 440 times lower that the one found in our study (π~0.012 for HA), suggesting that the variability of a pandemic virus is higher than that of an epidemic virus. Negative values of Tajima's D for HA and NA imply that no selection force is yet influencing the success of the pandemic virus. Some studies show different extent of changes: a study with 423 complete genomes of human H3N2 influenza A virus collected between 1997 and 2005 in New York, USA, revealed that adaptive evolution occurred only sporadically, rather, a stochastic process of viral migration and clade reassortment played a vital role in shaping short-term evolutionary dynamics [20]. Another study analyzed 357 nucleotide sequences for HA from A H1N1 and found some codons under positive selection, suggesting that these changes may have predictive value for future epidemic variants [21]. Therefore, precaution should be taken because A H1N1 may peak again, since our data show that the variants are still in expansion. Network analysis showed that the major dispersion center was shared by China, Mexico and USA during the initial four months, and probably reflect the fact that there was a greater interest in the scientific community for submitting and reporting viral sequences in GenBank. Also, HA was more variable than NA, which is in accordance with the statement that the HA gene exhibits a rapid mutation rate [22].

When integrating data of F_ST_, G_ST _and Nm of this new A H1N1 it was observed that the virus had different behaviors along 2009 when comparing paired countries; which was, in general, independent of their geographical proximity. The extremes were found in USA and Mexico; the former showed a high distribution of virus variants to and from several countries in the initial four months of the pandemic, becoming a worldwide dispersion towards the end of the year, while in Mexico minimal influx of variants was seen in the initial four months. This was probably due to the governmental actions taken in April to contain the influenza outbreak in the whole Mexican Republic [23] or to the exclusion of small sequences for the analyses performed. Also, some countries decided to close their borders or send travel alerts recommending their citizens to avoid nonessential travel to Mexico [stated in 2009 in 24]. At the beginning of the pandemic, federal and local health authorities in Mexico established several measures, mainly focused in two lines 1) social spacing that included closing temporally churches, schools, restaurants, cinemas, theaters and other sites of massive human concentration, 2) intensive hygiene campaign that publicized basic aspects of health such as continuous hand washing, avoiding unprotected sneezing, using disposable surgical masks and surveillance of symptoms associated to flu.

## Abbreviations

F_ST_: coancestry coefficient statistics; G_ST_: genetic differentiation index; HA: hemagglutinin; NA: neuraminidase; NN: migration index; VV: viral variants; VV57NA: viral variant 57 of neuraminidase; WHO: World Health Organization; π: nucleotide diversity; θ: haplotype polymorphism.

## Competing interests

The authors declare that they have no competing interests.

## Authors' contributions

FMH, DEJG, AMF and GVC collected data and carried out the bioinformatics analysis. GV, SKK and AF participated in biological interpretations of results and in the discussion. PM and MRV formulated the idea. All authors contributed in writing the manuscript.

## Supplementary Material

Additional file 1 **A H1N1 gene sequences used for the genetic diversity analysis**. List of GenBank sequences of A H1N1, number of accession and country of origin. Click here for file

Additional file 2 **Population genetic indexes among paired sequences of A H1N1 obtained from different countries**. List of values (indexes) obtained for population genetic analysis among paired sequences from different countries after DnaSP v4 analysis. Click here for file
