# Supplementary material for: What happened after the initial global spread of pandemic human influenza virus A (H1N1)? A population genetics approach
Source: Virol J. 2010 Aug 20;7:196. doi: 10.1186/1743-422X-7-196 (PMC2936310; doi:10.1186/1743-422X-7-196)
Supplement: Additional file 1 — A H1N1 gene sequences used for the genetic diversity analysis. List of GenBank sequences of A H1N1, number of accession and country of origin. [file 1743-422X-7-196-S1.DOC]

| Supplementary table 1. Variants and sequences used during population genetics analysis | | | | | | | | |  |
| --- | --- | --- | --- | --- | --- | --- | --- | --- | --- |
| HA data | | | | | NA data | | | | |
| VV | Accession number | Sequence length | Country | Date of sample colection | VV | Accession number | Sequence length | Country | Date of sample colection |
| 1 | [CY053668](http://www.ncbi.nlm.nih.gov/entrez/viewer.fcgi?val=CY053668) | 1726 | Russia | Mar | 1 | [CY053669](http://www.ncbi.nlm.nih.gov/entrez/viewer.fcgi?val=CY053669) | 1429 | Russia | Mar |
| 2 | [GQ476011](http://www.ncbi.nlm.nih.gov/entrez/viewer.fcgi?val=GQ476011) | 1698 | USA | 01-mar | 2 | [GQ476012](http://www.ncbi.nlm.nih.gov/entrez/viewer.fcgi?val=GQ476012) | 1413 | USA | 01-mar |
| 3 | [GQ476081](http://www.ncbi.nlm.nih.gov/entrez/viewer.fcgi?val=GQ476081) | 1698 | USA | 01-mar |  | [GQ476121](http://www.ncbi.nlm.nih.gov/entrez/viewer.fcgi?val=GQ476121) | 1413 | USA | 03-mar |
| 4 | [GQ475971](http://www.ncbi.nlm.nih.gov/entrez/viewer.fcgi?val=GQ475971) | 1698 | USA | 03-mar | 3 | [GQ476082](http://www.ncbi.nlm.nih.gov/entrez/viewer.fcgi?val=GQ476082) | 1413 | USA | 01-mar |
| 5 | [GQ476120](http://www.ncbi.nlm.nih.gov/entrez/viewer.fcgi?val=GQ476120) | 1698 | USA | 03-mar | 4 | [GQ475972](http://www.ncbi.nlm.nih.gov/entrez/viewer.fcgi?val=GQ475972) | 1413 | USA | 03-mar |
| 6 | [GQ476087](http://www.ncbi.nlm.nih.gov/entrez/viewer.fcgi?val=GQ476087) | 1698 | USA | 07-mar | 5 | [GQ476088](http://www.ncbi.nlm.nih.gov/entrez/viewer.fcgi?val=GQ476088) | 1413 | USA | 07-mar |
| 7 | [GQ475933](http://www.ncbi.nlm.nih.gov/entrez/viewer.fcgi?val=GQ475933) | 1698 | USA | 09-mar | 6 | [GQ475934](http://www.ncbi.nlm.nih.gov/entrez/viewer.fcgi?val=GQ475934) | 1413 | USA | 09-mar |
| 8 | [GQ475981](http://www.ncbi.nlm.nih.gov/entrez/viewer.fcgi?val=GQ475981) | 1698 | USA | 09-mar | 7 | [GQ475982](http://www.ncbi.nlm.nih.gov/entrez/viewer.fcgi?val=GQ475982) | 1413 | USA | 09-mar |
| 9 | [GQ475984](http://www.ncbi.nlm.nih.gov/entrez/viewer.fcgi?val=GQ475984) | 1698 | USA | 09-mar | 8 | [GQ475985](http://www.ncbi.nlm.nih.gov/entrez/viewer.fcgi?val=GQ475985) | 1413 | USA | 09-mar |
|  | [GQ476063](http://www.ncbi.nlm.nih.gov/entrez/viewer.fcgi?val=GQ476063) | 1698 | USA | 27-Apr |  | [GQ476018](http://www.ncbi.nlm.nih.gov/entrez/viewer.fcgi?val=GQ476018) | 1413 | USA | 10-mar |
| 10 | [GQ476052](http://www.ncbi.nlm.nih.gov/entrez/viewer.fcgi?val=GQ476052) | 1698 | USA | 09-mar |  | [GQ476056](http://www.ncbi.nlm.nih.gov/entrez/viewer.fcgi?val=GQ476056) | 1413 | USA | 20-mar |
| 11 | [GQ476017](http://www.ncbi.nlm.nih.gov/entrez/viewer.fcgi?val=GQ476017) | 1698 | USA | 10-mar | 9 | [GQ476053](http://www.ncbi.nlm.nih.gov/entrez/viewer.fcgi?val=GQ476053) | 1413 | USA | 09-mar |
| 12 | [FJ912843](http://www.ncbi.nlm.nih.gov/entrez/viewer.fcgi?val=FJ912843) | 1566 | China | 11-mar | 10 | [GQ476061](http://www.ncbi.nlm.nih.gov/entrez/viewer.fcgi?val=GQ476061) | 1413 | USA | 12-mar |
| 13 | [GQ476060](http://www.ncbi.nlm.nih.gov/entrez/viewer.fcgi?val=GQ476060) | 1698 | USA | 12-mar | 11 | [GQ475988](http://www.ncbi.nlm.nih.gov/entrez/viewer.fcgi?val=GQ475988) | 1413 | USA | 13-mar |
| 14 | [GQ475987](http://www.ncbi.nlm.nih.gov/entrez/viewer.fcgi?val=GQ475987) | 1698 | USA | 13-mar |  | [GQ476021](http://www.ncbi.nlm.nih.gov/entrez/viewer.fcgi?val=GQ476021) | 1413 | USA | 13-mar |
| 15 | [GQ476020](http://www.ncbi.nlm.nih.gov/entrez/viewer.fcgi?val=GQ476020) | 1698 | USA | 13-mar |  | [GQ476064](http://www.ncbi.nlm.nih.gov/entrez/viewer.fcgi?val=GQ476064) | 1413 | USA | 27-Apr |
| 16 | [GQ475888](http://www.ncbi.nlm.nih.gov/entrez/viewer.fcgi?val=GQ475888) | 1698 | USA | 16-mar |  | [GQ475937](http://www.ncbi.nlm.nih.gov/entrez/viewer.fcgi?val=GQ475937) | 1413 | USA | 27-Apr |
| 17 | [GQ476123](http://www.ncbi.nlm.nih.gov/entrez/viewer.fcgi?val=GQ476123) | 1698 | USA | 16-mar | 12 | [GQ475877](http://www.ncbi.nlm.nih.gov/entrez/viewer.fcgi?val=GQ475877) | 1413 | USA | 17-mar |
| 18 | [GQ475876](http://www.ncbi.nlm.nih.gov/entrez/viewer.fcgi?val=GQ475876) | 1698 | USA | 17-mar | 13 | [GQ476015](http://www.ncbi.nlm.nih.gov/entrez/viewer.fcgi?val=GQ476015) | 1413 | USA | 17-mar |
| 19 | [GQ476014](http://www.ncbi.nlm.nih.gov/entrez/viewer.fcgi?val=GQ476014) | 1698 | USA | 17-mar | 14 | [GQ475883](http://www.ncbi.nlm.nih.gov/entrez/viewer.fcgi?val=GQ475883) | 1413 | USA | 18-mar |
| 20 | [GQ475882](http://www.ncbi.nlm.nih.gov/entrez/viewer.fcgi?val=GQ475882) | 1698 | USA | 18-mar | 15 | [GQ475886](http://www.ncbi.nlm.nih.gov/entrez/viewer.fcgi?val=GQ475886) | 1413 | USA | 22-mar |
| 21 | [GQ476055](http://www.ncbi.nlm.nih.gov/entrez/viewer.fcgi?val=GQ476055) | 1698 | USA | 20-mar | 16 | [GQ476126](http://www.ncbi.nlm.nih.gov/entrez/viewer.fcgi?val=GQ476126) | 1413 | USA | 25-mar |
| 22 | [GQ475885](http://www.ncbi.nlm.nih.gov/entrez/viewer.fcgi?val=GQ475885) | 1698 | USA | 22-mar | 17 | [FJ966956](http://www.ncbi.nlm.nih.gov/entrez/viewer.fcgi?val=FJ966956) | 1410 | USA | 30-mar |
| 23 | [GQ476125](http://www.ncbi.nlm.nih.gov/entrez/viewer.fcgi?val=GQ476125) | 1698 | USA | 25-mar |  | [GQ117105](http://www.ncbi.nlm.nih.gov/entrez/viewer.fcgi?val=GQ117105) | 1410 | USA | Apr |
| 24 | [FJ966952](http://www.ncbi.nlm.nih.gov/entrez/viewer.fcgi?val=FJ966952) | 1701 | USA | 30-mar |  | [GQ221802](http://www.ncbi.nlm.nih.gov/entrez/viewer.fcgi?val=GQ221802) | 1410 | USA | Apr |
| 25 | [FJ969511](http://www.ncbi.nlm.nih.gov/entrez/viewer.fcgi?val=FJ969511) | 1701 | USA | 8-Apr |  | [FJ971075](http://www.ncbi.nlm.nih.gov/entrez/viewer.fcgi?val=FJ971075) | 1410 | USA | 16-Apr |
|  | [GQ160607](http://www.ncbi.nlm.nih.gov/entrez/viewer.fcgi?val=GQ160607) | 1701 | USA | 21-Apr |  | [CY043088](http://www.ncbi.nlm.nih.gov/entrez/viewer.fcgi?val=CY043088) | 1410 | USA | 22-Apr |
|  | [CY043094](http://www.ncbi.nlm.nih.gov/entrez/viewer.fcgi?val=CY043094) | 1701 | USA | 21-Apr |  | [GQ221819](http://www.ncbi.nlm.nih.gov/entrez/viewer.fcgi?val=GQ221819) | 1410 | USA | 24-Apr |
|  | [GQ160579](http://www.ncbi.nlm.nih.gov/entrez/viewer.fcgi?val=GQ160579) | 1701 | USA | 24-Apr |  | [GQ221821](http://www.ncbi.nlm.nih.gov/entrez/viewer.fcgi?val=GQ221821) | 1410 | USA | 26-Apr |
|  | [GQ323483](http://www.ncbi.nlm.nih.gov/entrez/viewer.fcgi?val=GQ323483) | 1701 | USA | 30-Apr |  | [GQ221813](http://www.ncbi.nlm.nih.gov/entrez/viewer.fcgi?val=GQ221813) | 1410 | USA | 27-Apr |
|  | [GQ338358](http://www.ncbi.nlm.nih.gov/entrez/viewer.fcgi?val=GQ338358) | 1701 | USA | 30-Apr |  | [GQ117118](http://www.ncbi.nlm.nih.gov/entrez/viewer.fcgi?val=GQ117118) | 1410 | USA | 27-Apr |
|  | [GQ397644](http://www.ncbi.nlm.nih.gov/entrez/viewer.fcgi?val=GQ397644) | 935 | Spain | 31-mar |  | [GQ323554](http://www.ncbi.nlm.nih.gov/entrez/viewer.fcgi?val=GQ323554) | 1410 | USA | 28-Apr |
| 26 | [GQ475968](http://www.ncbi.nlm.nih.gov/entrez/viewer.fcgi?val=GQ475968) | 1698 | USA | 31-mar |  | [CY050069](http://www.ncbi.nlm.nih.gov/entrez/viewer.fcgi?val=CY050069) | 1410 | USA | 28-Apr |
| 27 | [GQ122099](http://www.ncbi.nlm.nih.gov/entrez/viewer.fcgi?val=GQ122099) | 1604 | Spain | Apr |  | [GQ338360](http://www.ncbi.nlm.nih.gov/entrez/viewer.fcgi?val=GQ338360) | 1410 | USA | 30-Apr |
| 28 | [GQ122102](http://www.ncbi.nlm.nih.gov/entrez/viewer.fcgi?val=GQ122102) | 1600 | Spain | Apr |  | [GQ377100](http://www.ncbi.nlm.nih.gov/entrez/viewer.fcgi?val=GQ377100) | 1410 | USA | 30-Apr |
| 29 | [GQ150338](http://www.ncbi.nlm.nih.gov/entrez/viewer.fcgi?val=GQ150338) | 1688 | New Zealand | Apr |  | [GQ377084](http://www.ncbi.nlm.nih.gov/entrez/viewer.fcgi?val=GQ377084) | 1410 | USA | 01-may |
| 30 | [GQ160534](http://www.ncbi.nlm.nih.gov/entrez/viewer.fcgi?val=GQ160534) | 1701 | USA | Apr |  | [GQ338351](http://www.ncbi.nlm.nih.gov/entrez/viewer.fcgi?val=GQ338351) | 1410 | USA | 02-may |
| 31 | [GQ160568](http://www.ncbi.nlm.nih.gov/entrez/viewer.fcgi?val=GQ160568) | 1701 | USA | Apr |  | [CY049869](http://www.ncbi.nlm.nih.gov/entrez/viewer.fcgi?val=CY049869) | 1410 | USA | 02-may |
| 32 | [GQ915017](http://www.ncbi.nlm.nih.gov/entrez/viewer.fcgi?val=GQ915017) | 1701 | Brazil | 19-jul |  | [CY049877](http://www.ncbi.nlm.nih.gov/entrez/viewer.fcgi?val=CY049877) | 1410 | USA | 02-may |
|  | [GQ166752](http://www.ncbi.nlm.nih.gov/entrez/viewer.fcgi?val=GQ166752) | 1677 | Portugal | Apr |  | [CY049845](http://www.ncbi.nlm.nih.gov/entrez/viewer.fcgi?val=CY049845) | 1410 | USA | 04-may |
| 33 | [GQ166760](http://www.ncbi.nlm.nih.gov/entrez/viewer.fcgi?val=GQ166760) | 1664 | Israel | Apr |  | [CY049822](http://www.ncbi.nlm.nih.gov/entrez/viewer.fcgi?val=CY049822) | 1410 | USA | 05-may |
| 34 | [GQ168645](http://www.ncbi.nlm.nih.gov/entrez/viewer.fcgi?val=GQ168645) | 1701 | USA | Apr |  | [GQ323458](http://www.ncbi.nlm.nih.gov/entrez/viewer.fcgi?val=GQ323458) | 1410 | USA | 05-may |
| 35 | [GQ168659](http://www.ncbi.nlm.nih.gov/entrez/viewer.fcgi?val=GQ168659) | 1701 | USA | Apr |  | [GQ329102](http://www.ncbi.nlm.nih.gov/entrez/viewer.fcgi?val=GQ329102) | 1410 | France | 17-may |
| 36 | [GQ221801](http://www.ncbi.nlm.nih.gov/entrez/viewer.fcgi?val=GQ221801) | 1701 | USA | Apr |  | [GU014805](http://www.ncbi.nlm.nih.gov/entrez/viewer.fcgi?val=GU014805) | 1410 | Japan | 26-may |
| 37 | [GQ221803](http://www.ncbi.nlm.nih.gov/entrez/viewer.fcgi?val=GQ221803) | 1701 | USA | Apr | 18 | [GQ475969](http://www.ncbi.nlm.nih.gov/entrez/viewer.fcgi?val=GQ475969) | 1413 | USA | 31-mar |
| 38 | [GQ265532](http://www.ncbi.nlm.nih.gov/entrez/viewer.fcgi?val=GQ265532) | 1552 | Spain | Apr | 19 | [GQ122100](http://www.ncbi.nlm.nih.gov/entrez/viewer.fcgi?val=GQ122100) | 1289 | Spain | Apr |
| 39 | [CY041968](http://www.ncbi.nlm.nih.gov/entrez/viewer.fcgi?val=CY041968) | 1762 | Israel | Apr | 20 | [GQ122101](http://www.ncbi.nlm.nih.gov/entrez/viewer.fcgi?val=GQ122101) | 1270 | Spain | Apr |
| 40 | [CY041973](http://www.ncbi.nlm.nih.gov/entrez/viewer.fcgi?val=CY041973) | 1679 | Israel | Apr | 21 | [GQ132186](http://www.ncbi.nlm.nih.gov/entrez/viewer.fcgi?val=GQ132186) | 1293 | Spain | Apr |
| 41 | [CY041976](http://www.ncbi.nlm.nih.gov/entrez/viewer.fcgi?val=CY041976) | 1688 | Israel | Apr | 22 | [GQ150336](http://www.ncbi.nlm.nih.gov/entrez/viewer.fcgi?val=GQ150336) | 1396 | New Zealand | Apr |
| 42 | [FJ974024](http://www.ncbi.nlm.nih.gov/entrez/viewer.fcgi?val=FJ974024) | 1774 | Canada | Apr | 23 | [GQ160533](http://www.ncbi.nlm.nih.gov/entrez/viewer.fcgi?val=GQ160533) | 1410 | USA | Apr |
| 43 | [CY053650](http://www.ncbi.nlm.nih.gov/entrez/viewer.fcgi?val=CY053650) | 1727 | Russia | Apr |  | [GQ365448](http://www.ncbi.nlm.nih.gov/entrez/viewer.fcgi?val=GQ365448) | 1410 | Japan | 11-jun |
| 44 | [CY053658](http://www.ncbi.nlm.nih.gov/entrez/viewer.fcgi?val=CY053658) | 1763 | Russia | Apr |  | [GU065291](http://www.ncbi.nlm.nih.gov/entrez/viewer.fcgi?val=GU065291) | 1410 | Poland | 30-jun |
| 45 | [CY053664](http://www.ncbi.nlm.nih.gov/entrez/viewer.fcgi?val=CY053664) | 1760 | Russia | Apr |  | [GU123914](http://www.ncbi.nlm.nih.gov/entrez/viewer.fcgi?val=GU123914) | 1410 | Italy | Jul |
| 46 | [CY053670](http://www.ncbi.nlm.nih.gov/entrez/viewer.fcgi?val=CY053670) | 1724 | Russia | Apr |  | [GU134718](http://www.ncbi.nlm.nih.gov/entrez/viewer.fcgi?val=GU134718) | 1410 | Italy | Jul |
| 47 | [CY053674](http://www.ncbi.nlm.nih.gov/entrez/viewer.fcgi?val=CY053674) | 1697 | Russia | Apr |  | [GU134721](http://www.ncbi.nlm.nih.gov/entrez/viewer.fcgi?val=GU134721) | 1410 | Italy | Jul |
| 48 | [CY053676](http://www.ncbi.nlm.nih.gov/entrez/viewer.fcgi?val=CY053676) | 1725 | Russia | Apr | 24 | [GQ166750](http://www.ncbi.nlm.nih.gov/entrez/viewer.fcgi?val=GQ166750) | 1403 | Portugal | Apr |
| 49 | [CY053656](http://www.ncbi.nlm.nih.gov/entrez/viewer.fcgi?val=CY053656) | 1676 | Russia | Apr | 25 | [GQ166759](http://www.ncbi.nlm.nih.gov/entrez/viewer.fcgi?val=GQ166759) | 1371 | Israel | Apr |
| 50 | [FJ966082](http://www.ncbi.nlm.nih.gov/entrez/viewer.fcgi?val=FJ966082) | 1701 | USA | 1-Apr | 26 | [GQ168658](http://www.ncbi.nlm.nih.gov/entrez/viewer.fcgi?val=GQ168658) | 1410 | USA | Apr |
| 51 | [GQ117044](http://www.ncbi.nlm.nih.gov/entrez/viewer.fcgi?val=GQ117044) | 1701 | USA | 1-Apr |  | [GQ225367](http://www.ncbi.nlm.nih.gov/entrez/viewer.fcgi?val=GQ225367) | 1410 | China | 22-may |
|  | [GQ338402](http://www.ncbi.nlm.nih.gov/entrez/viewer.fcgi?val=GQ338402) | 1701 | USA | 30-Apr | 27 | [GQ221804](http://www.ncbi.nlm.nih.gov/entrez/viewer.fcgi?val=GQ221804) | 1410 | USA | Apr |
|  | [GQ149651](http://www.ncbi.nlm.nih.gov/entrez/viewer.fcgi?val=GQ149651) | 993 | Mexico | 2-Apr | 28 | [CY041970](http://www.ncbi.nlm.nih.gov/entrez/viewer.fcgi?val=CY041970) | 1438 | Israel | Apr |
| 52 | [GQ149665](http://www.ncbi.nlm.nih.gov/entrez/viewer.fcgi?val=GQ149665) | 993 | Mexico | 14-Apr | 29 | [CY041977](http://www.ncbi.nlm.nih.gov/entrez/viewer.fcgi?val=CY041977) | 1440 | Israel | Apr |
|  | [GQ149654](http://www.ncbi.nlm.nih.gov/entrez/viewer.fcgi?val=GQ149654) | 1701 | Mexico | 2-Apr | 30 | [CY045932](http://www.ncbi.nlm.nih.gov/entrez/viewer.fcgi?val=CY045932) | 1427 | Canada | Apr |
| 53 | [GQ149662](http://www.ncbi.nlm.nih.gov/entrez/viewer.fcgi?val=GQ149662) | 1701 | Mexico | 2-Apr | 31 | [CY053651](http://www.ncbi.nlm.nih.gov/entrez/viewer.fcgi?val=CY053651) | 1399 | Russia | Apr |
|  | [GQ149668](http://www.ncbi.nlm.nih.gov/entrez/viewer.fcgi?val=GQ149668) | 1701 | Mexico | 2-Apr | 32 | [CY053657](http://www.ncbi.nlm.nih.gov/entrez/viewer.fcgi?val=CY053657) | 1453 | Russia | Apr |
|  | [GQ149689](http://www.ncbi.nlm.nih.gov/entrez/viewer.fcgi?val=GQ149689) | 1701 | Mexico | 2-Apr | 33 | [CY053659](http://www.ncbi.nlm.nih.gov/entrez/viewer.fcgi?val=CY053659) | 1453 | Russia | Apr |
|  | [GQ223112](http://www.ncbi.nlm.nih.gov/entrez/viewer.fcgi?val=GQ223112) | 1701 | Mexico | 3-Apr | 34 | [CY053665](http://www.ncbi.nlm.nih.gov/entrez/viewer.fcgi?val=CY053665) | 1457 | Russia | Apr |
|  | [GQ162170](http://www.ncbi.nlm.nih.gov/entrez/viewer.fcgi?val=GQ162170) | 1701 | Mexico | 2-Apr | 35 | [CY053671](http://www.ncbi.nlm.nih.gov/entrez/viewer.fcgi?val=CY053671) | 1453 | Russia | Apr |
| 54 | [GQ162194](http://www.ncbi.nlm.nih.gov/entrez/viewer.fcgi?val=GQ162194) | 1701 | Mexico | 2-Apr | 36 | [CY053677](http://www.ncbi.nlm.nih.gov/entrez/viewer.fcgi?val=CY053677) | 1429 | Russia | Apr |
| 55 | [GQ149692](http://www.ncbi.nlm.nih.gov/entrez/viewer.fcgi?val=GQ149692) | 1701 | Mexico | 7-Apr | 37 | [FJ966084](http://www.ncbi.nlm.nih.gov/entrez/viewer.fcgi?val=FJ966084) | 1410 | USA | 1-Apr |
| 56 | [GQ162182](http://www.ncbi.nlm.nih.gov/entrez/viewer.fcgi?val=GQ162182) | 1701 | Mexico | 13-Apr |  | [FJ969517](http://www.ncbi.nlm.nih.gov/entrez/viewer.fcgi?val=FJ969517) | 1410 | USA | 1-Apr |
|  | [GQ162204](http://www.ncbi.nlm.nih.gov/entrez/viewer.fcgi?val=GQ162204) | 1701 | Mexico | 20-Apr |  | [FJ984386](http://www.ncbi.nlm.nih.gov/entrez/viewer.fcgi?val=FJ984386) | 1410 | USA | 9-Apr |
|  | [GQ162174](http://www.ncbi.nlm.nih.gov/entrez/viewer.fcgi?val=GQ162174) | 1701 | Mexico | 20-Apr |  | [GQ377050](http://www.ncbi.nlm.nih.gov/entrez/viewer.fcgi?val=GQ377050) | 1410 | USA | 9-Apr |
|  | [GQ117067](http://www.ncbi.nlm.nih.gov/entrez/viewer.fcgi?val=GQ117067) | 1701 | USA | 22-Apr |  | [GQ377079](http://www.ncbi.nlm.nih.gov/entrez/viewer.fcgi?val=GQ377079) | 1410 | USA | 9-Apr |
|  | [GQ232028](http://www.ncbi.nlm.nih.gov/entrez/viewer.fcgi?val=GQ232028) | 1701 | USA | 24-Apr |  | [FJ966973](http://www.ncbi.nlm.nih.gov/entrez/viewer.fcgi?val=FJ966973) | 1410 | USA | 15-Apr |
|  | [GQ160538](http://www.ncbi.nlm.nih.gov/entrez/viewer.fcgi?val=GQ160538) | 1701 | USA | 26-Apr |  | [GQ338404](http://www.ncbi.nlm.nih.gov/entrez/viewer.fcgi?val=GQ338404) | 1410 | USA | 30-Apr |
|  | [GQ221805](http://www.ncbi.nlm.nih.gov/entrez/viewer.fcgi?val=GQ221805) | 1701 | USA | 26-Apr |  | [GQ334332](http://www.ncbi.nlm.nih.gov/entrez/viewer.fcgi?val=GQ334332) | 1410 | Japan | 07-jun |
|  | [GQ338391](http://www.ncbi.nlm.nih.gov/entrez/viewer.fcgi?val=GQ338391) | 1701 | USA | 27-Apr |  | [GQ334340](http://www.ncbi.nlm.nih.gov/entrez/viewer.fcgi?val=GQ334340) | 1410 | Japan | 07-jun |
|  | [GQ168623](http://www.ncbi.nlm.nih.gov/entrez/viewer.fcgi?val=GQ168623) | 1701 | USA | 28-Apr |  | [GQ365438](http://www.ncbi.nlm.nih.gov/entrez/viewer.fcgi?val=GQ365438) | 1410 | Japan | 13-jun |
|  | [GQ168631](http://www.ncbi.nlm.nih.gov/entrez/viewer.fcgi?val=GQ168631) | 1701 | USA | 28-Apr | 38 | [GQ149650](http://www.ncbi.nlm.nih.gov/entrez/viewer.fcgi?val=GQ149650) | 1410 | Mexico | 2-Apr |
|  | [GQ200208](http://www.ncbi.nlm.nih.gov/entrez/viewer.fcgi?val=GQ200208) | 1701 | USA | 28-Apr |  | [GQ160554](http://www.ncbi.nlm.nih.gov/entrez/viewer.fcgi?val=GQ160554) | 1410 | USA | Apr |
|  | [GQ160578](http://www.ncbi.nlm.nih.gov/entrez/viewer.fcgi?val=GQ160578) | 1701 | USA | 28-Apr |  | [GQ160558](http://www.ncbi.nlm.nih.gov/entrez/viewer.fcgi?val=GQ160558) | 1410 | USA | Apr |
|  | [GQ200255](http://www.ncbi.nlm.nih.gov/entrez/viewer.fcgi?val=GQ200255) | 1701 | USA | 29-Apr |  | [GQ149688](http://www.ncbi.nlm.nih.gov/entrez/viewer.fcgi?val=GQ149688) | 1410 | Mexico | 2-Apr |
|  | [GQ377087](http://www.ncbi.nlm.nih.gov/entrez/viewer.fcgi?val=GQ377087) | 1701 | USA | 29-Apr |  | [GQ149656](http://www.ncbi.nlm.nih.gov/entrez/viewer.fcgi?val=GQ149656) | 1410 | Mexico | 2-Apr |
|  | [GQ338372](http://www.ncbi.nlm.nih.gov/entrez/viewer.fcgi?val=GQ338372) | 1701 | USA | 29-Apr |  | [GQ149659](http://www.ncbi.nlm.nih.gov/entrez/viewer.fcgi?val=GQ149659) | 1410 | Mexico | 2-Apr |
|  | [GQ377075](http://www.ncbi.nlm.nih.gov/entrez/viewer.fcgi?val=GQ377075) | 1701 | USA | 30-Apr |  | [GQ162169](http://www.ncbi.nlm.nih.gov/entrez/viewer.fcgi?val=GQ162169) | 1410 | Mexico | 2-Apr |
|  | [GQ232052](http://www.ncbi.nlm.nih.gov/entrez/viewer.fcgi?val=GQ232052) | 1701 | USA | 30-Apr |  | [GQ162193](http://www.ncbi.nlm.nih.gov/entrez/viewer.fcgi?val=GQ162193) | 1410 | Mexico | 2-Apr |
|  | [CY046283](http://www.ncbi.nlm.nih.gov/entrez/viewer.fcgi?val=CY046283) | 1701 | USA | 03-may |  | [GQ223113](http://www.ncbi.nlm.nih.gov/entrez/viewer.fcgi?val=GQ223113) | 1410 | Mexico | 3-Apr |
|  | [GQ231993](http://www.ncbi.nlm.nih.gov/entrez/viewer.fcgi?val=GQ231993) | 1701 | USA | 05-may |  | [GQ149691](http://www.ncbi.nlm.nih.gov/entrez/viewer.fcgi?val=GQ149691) | 1410 | Mexico | 7-Apr |
|  | [GQ323509](http://www.ncbi.nlm.nih.gov/entrez/viewer.fcgi?val=GQ323509) | 1701 | USA | 07-may |  | [GQ149622](http://www.ncbi.nlm.nih.gov/entrez/viewer.fcgi?val=GQ149622) | 1410 | Mexico | 14-Apr |
|  | [GQ165814](http://www.ncbi.nlm.nih.gov/entrez/viewer.fcgi?val=GQ165814) | 1701 | Japan | 08-may |  | [GQ149644](http://www.ncbi.nlm.nih.gov/entrez/viewer.fcgi?val=GQ149644) | 1410 | Mexico | 14-Apr |
|  | [GQ165815](http://www.ncbi.nlm.nih.gov/entrez/viewer.fcgi?val=GQ165815) | 1701 | Japan | 08-may |  | [GQ149664](http://www.ncbi.nlm.nih.gov/entrez/viewer.fcgi?val=GQ149664) | 1410 | Mexico | 14-Apr |
|  | [GQ249337](http://www.ncbi.nlm.nih.gov/entrez/viewer.fcgi?val=GQ249337) | 1701 | France | 11-may |  | [FJ966981](http://www.ncbi.nlm.nih.gov/entrez/viewer.fcgi?val=FJ966981) | 1410 | USA | 14-Apr |
|  | [GQ225373](http://www.ncbi.nlm.nih.gov/entrez/viewer.fcgi?val=GQ225373) | 1701 | China | 15-may |  | [FJ981614](http://www.ncbi.nlm.nih.gov/entrez/viewer.fcgi?val=FJ981614) | 1410 | USA | 14-Apr |
|  | [GQ219580](http://www.ncbi.nlm.nih.gov/entrez/viewer.fcgi?val=GQ219580) | 1701 | Japan | 16-may |  | [GQ221799](http://www.ncbi.nlm.nih.gov/entrez/viewer.fcgi?val=GQ221799) | 1410 | USA | 14-Apr |
|  | [GQ219581](http://www.ncbi.nlm.nih.gov/entrez/viewer.fcgi?val=GQ219581) | 1701 | Japan | 17-may |  | [GQ162187](http://www.ncbi.nlm.nih.gov/entrez/viewer.fcgi?val=GQ162187) | 1410 | Mexico | 14-Apr |
|  | [GQ219575](http://www.ncbi.nlm.nih.gov/entrez/viewer.fcgi?val=GQ219575) | 1701 | Japan | 17-may |  | [GQ377071](http://www.ncbi.nlm.nih.gov/entrez/viewer.fcgi?val=GQ377071) | 1410 | USA | 14-Apr |
|  | [GQ219576](http://www.ncbi.nlm.nih.gov/entrez/viewer.fcgi?val=GQ219576) | 1701 | Japan | 17-may |  | [CY044245](http://www.ncbi.nlm.nih.gov/entrez/viewer.fcgi?val=CY044245) | 1410 | USA | 15-Apr |
|  | [GQ261272](http://www.ncbi.nlm.nih.gov/entrez/viewer.fcgi?val=GQ261272) | 1701 | Japan | 18-may |  | [GQ457486](http://www.ncbi.nlm.nih.gov/entrez/viewer.fcgi?val=GQ457486) | 1410 | USA | 15-Apr |
|  | [GQ287621](http://www.ncbi.nlm.nih.gov/entrez/viewer.fcgi?val=GQ287621) | 1701 | Japan | 19-may |  | [GQ162171](http://www.ncbi.nlm.nih.gov/entrez/viewer.fcgi?val=GQ162171) | 1410 | Mexico | 15-Apr |
|  | [AB514227](http://www.ncbi.nlm.nih.gov/entrez/viewer.fcgi?val=AB514227) | 1701 | Japan | 21-may |  | [GQ323512](http://www.ncbi.nlm.nih.gov/entrez/viewer.fcgi?val=GQ323512) | 1410 | USA | 15-Apr |
|  | [GU014794](http://www.ncbi.nlm.nih.gov/entrez/viewer.fcgi?val=GU014794) | 1701 | Japan | 18-jun |  | [GQ397120](http://www.ncbi.nlm.nih.gov/entrez/viewer.fcgi?val=GQ397120) | 1410 | Mexico | 20-Apr |
|  | [GQ476023](http://www.ncbi.nlm.nih.gov/entrez/viewer.fcgi?val=GQ476023) | 1698 | USA | 8-Apr |  | [FJ984383](http://www.ncbi.nlm.nih.gov/entrez/viewer.fcgi?val=FJ984383) | 1410 | USA | 23-Apr |
| 57 | [FJ966974](http://www.ncbi.nlm.nih.gov/entrez/viewer.fcgi?val=FJ966974) | 1701 | USA | 9-Apr |  | [GQ117048](http://www.ncbi.nlm.nih.gov/entrez/viewer.fcgi?val=GQ117048) | 1410 | USA | 24-Apr |
|  | [FJ971076](http://www.ncbi.nlm.nih.gov/entrez/viewer.fcgi?val=FJ971076) | 1701 | USA | 9-Apr |  | [GQ168632](http://www.ncbi.nlm.nih.gov/entrez/viewer.fcgi?val=GQ168632) | 1410 | USA | 24-Apr |
|  | [FJ981613](http://www.ncbi.nlm.nih.gov/entrez/viewer.fcgi?val=FJ981613) | 1701 | USA | 9-Apr |  | [GQ323526](http://www.ncbi.nlm.nih.gov/entrez/viewer.fcgi?val=GQ323526) | 1410 | USA | 24-Apr |
|  | [GQ377052](http://www.ncbi.nlm.nih.gov/entrez/viewer.fcgi?val=GQ377052) | 1701 | USA | 9-Apr |  | [GQ377060](http://www.ncbi.nlm.nih.gov/entrez/viewer.fcgi?val=GQ377060) | 1410 | USA | 25-Apr |
|  | [FJ969540](http://www.ncbi.nlm.nih.gov/entrez/viewer.fcgi?val=FJ969540) | 1701 | USA | 9-Apr |  | [GQ338411](http://www.ncbi.nlm.nih.gov/entrez/viewer.fcgi?val=GQ338411) | 1410 | USA | 26-Apr |
| 58 | [GQ476148](http://www.ncbi.nlm.nih.gov/entrez/viewer.fcgi?val=GQ476148) | 1698 | USA | 9-Apr |  | [GQ221795](http://www.ncbi.nlm.nih.gov/entrez/viewer.fcgi?val=GQ221795) | 1410 | USA | 26-Apr |
| 59 | [GQ476078](http://www.ncbi.nlm.nih.gov/entrez/viewer.fcgi?val=GQ476078) | 1698 | USA | 13-Apr |  | [GQ221806](http://www.ncbi.nlm.nih.gov/entrez/viewer.fcgi?val=GQ221806) | 1410 | USA | 26-Apr |
| 60 | [FJ966982](http://www.ncbi.nlm.nih.gov/entrez/viewer.fcgi?val=FJ966982) | 1701 | USA | 14-Apr |  | [GQ117053](http://www.ncbi.nlm.nih.gov/entrez/viewer.fcgi?val=GQ117053) | 1410 | USA | 26-Apr |
| 61 | [FJ981612](http://www.ncbi.nlm.nih.gov/entrez/viewer.fcgi?val=FJ981612) | 1701 | USA | 14-Apr |  | [GQ221810](http://www.ncbi.nlm.nih.gov/entrez/viewer.fcgi?val=GQ221810) | 1410 | USA | 27-Apr |
|  | [FJ981615](http://www.ncbi.nlm.nih.gov/entrez/viewer.fcgi?val=FJ981615) | 1701 | USA | 14-Apr |  | [GQ323441](http://www.ncbi.nlm.nih.gov/entrez/viewer.fcgi?val=GQ323441) | 1410 | USA | 27-Apr |
|  | [CY044235](http://www.ncbi.nlm.nih.gov/entrez/viewer.fcgi?val=CY044235) | 1701 | USA | 14-Apr |  | [GQ221824](http://www.ncbi.nlm.nih.gov/entrez/viewer.fcgi?val=GQ221824) | 1410 | USA | 28-Apr |
|  | [FJ966959](http://www.ncbi.nlm.nih.gov/entrez/viewer.fcgi?val=FJ966959) | 1701 | USA | 15-Apr |  | [GQ166659](http://www.ncbi.nlm.nih.gov/entrez/viewer.fcgi?val=GQ166659) | 1410 | United Kingdom | 28-Apr |
|  | [GQ168861](http://www.ncbi.nlm.nih.gov/entrez/viewer.fcgi?val=GQ168861) | 1701 | USA | 15-Apr |  | [GQ160540](http://www.ncbi.nlm.nih.gov/entrez/viewer.fcgi?val=GQ160540) | 1410 | USA | 28-Apr |
|  | [FJ984385](http://www.ncbi.nlm.nih.gov/entrez/viewer.fcgi?val=FJ984385) | 1701 | USA | 23-Apr |  | [GQ160581](http://www.ncbi.nlm.nih.gov/entrez/viewer.fcgi?val=GQ160581) | 1410 | USA | 28-Apr |
|  | [GQ117051](http://www.ncbi.nlm.nih.gov/entrez/viewer.fcgi?val=GQ117051) | 1701 | USA | 24-Apr |  | [GQ160593](http://www.ncbi.nlm.nih.gov/entrez/viewer.fcgi?val=GQ160593) | 1410 | USA | 28-Apr |
|  | [GQ117059](http://www.ncbi.nlm.nih.gov/entrez/viewer.fcgi?val=GQ117059) | 1701 | USA | 24-Apr |  | [GQ338396](http://www.ncbi.nlm.nih.gov/entrez/viewer.fcgi?val=GQ338396) | 1410 | USA | 28-Apr |
|  | [GQ200221](http://www.ncbi.nlm.nih.gov/entrez/viewer.fcgi?val=GQ200221) | 1701 | USA | 24-Apr |  | [GQ338357](http://www.ncbi.nlm.nih.gov/entrez/viewer.fcgi?val=GQ338357) | 1410 | USA | 28-Apr |
|  | [GQ168671](http://www.ncbi.nlm.nih.gov/entrez/viewer.fcgi?val=GQ168671) | 1701 | USA | 25-Apr |  | [GQ323568](http://www.ncbi.nlm.nih.gov/entrez/viewer.fcgi?val=GQ323568) | 1410 | USA | 28-Apr |
|  | [GQ160574](http://www.ncbi.nlm.nih.gov/entrez/viewer.fcgi?val=GQ160574) | 1701 | USA | 26-Apr |  | [GQ338377](http://www.ncbi.nlm.nih.gov/entrez/viewer.fcgi?val=GQ338377) | 1410 | USA | 29-Apr |
|  | [GQ221791](http://www.ncbi.nlm.nih.gov/entrez/viewer.fcgi?val=GQ221791) | 1701 | USA | 28-Apr |  | [GQ323521](http://www.ncbi.nlm.nih.gov/entrez/viewer.fcgi?val=GQ323521) | 1410 | USA | 29-Apr |
|  | [FJ998208](http://www.ncbi.nlm.nih.gov/entrez/viewer.fcgi?val=FJ998208) | 1743 | Mexico | 14-Apr |  | [CY049830](http://www.ncbi.nlm.nih.gov/entrez/viewer.fcgi?val=CY049830) | 1410 | USA | 30-Apr |
| 62 | [GQ402190](http://www.ncbi.nlm.nih.gov/entrez/viewer.fcgi?val=GQ402190) | 1743 | Mexico | 29-Apr |  | [GQ323556](http://www.ncbi.nlm.nih.gov/entrez/viewer.fcgi?val=GQ323556) | 1410 | USA | 30-Apr |
|  | [GQ402196](http://www.ncbi.nlm.nih.gov/entrez/viewer.fcgi?val=GQ402196) | 1743 | Canada | 04-may |  | [GQ323557](http://www.ncbi.nlm.nih.gov/entrez/viewer.fcgi?val=GQ323557) | 1410 | USA | 30-Apr |
|  | [GQ402201](http://www.ncbi.nlm.nih.gov/entrez/viewer.fcgi?val=GQ402201) | 1743 | Canada | 24-may |  | [GQ338330](http://www.ncbi.nlm.nih.gov/entrez/viewer.fcgi?val=GQ338330) | 1410 | USA | 01-may |
|  | [GQ402205](http://www.ncbi.nlm.nih.gov/entrez/viewer.fcgi?val=GQ402205) | 1743 | Canada | 29-may |  | [GQ377077](http://www.ncbi.nlm.nih.gov/entrez/viewer.fcgi?val=GQ377077) | 1410 | USA | 01-may |
|  | [GQ149623](http://www.ncbi.nlm.nih.gov/entrez/viewer.fcgi?val=GQ149623) | 1701 | Mexico | 14-Apr |  | [GQ222423](http://www.ncbi.nlm.nih.gov/entrez/viewer.fcgi?val=GQ222423) | 1410 | France | 01-may |
| 63 | [GQ149647](http://www.ncbi.nlm.nih.gov/entrez/viewer.fcgi?val=GQ149647) | 1701 | Mexico | 14-Apr |  | [GQ323571](http://www.ncbi.nlm.nih.gov/entrez/viewer.fcgi?val=GQ323571) | 1410 | USA | 04-may |
|  | [GQ162185](http://www.ncbi.nlm.nih.gov/entrez/viewer.fcgi?val=GQ162185) | 1701 | Mexico | 14-Apr |  | [GQ323459](http://www.ncbi.nlm.nih.gov/entrez/viewer.fcgi?val=GQ323459) | 1410 | USA | 05-may |
|  | [GQ162202](http://www.ncbi.nlm.nih.gov/entrez/viewer.fcgi?val=GQ162202) | 1701 | Mexico | 14-Apr |  | [GQ323546](http://www.ncbi.nlm.nih.gov/entrez/viewer.fcgi?val=GQ323546) | 1410 | USA | 07-may |
|  | [GQ149671](http://www.ncbi.nlm.nih.gov/entrez/viewer.fcgi?val=GQ149671) | 1701 | Mexico | 14-Apr |  | [GQ254714](http://www.ncbi.nlm.nih.gov/entrez/viewer.fcgi?val=GQ254714) | 1410 | France | 11-may |
| 64 | [GQ149674](http://www.ncbi.nlm.nih.gov/entrez/viewer.fcgi?val=GQ149674) | 1701 | Mexico | 14-Apr |  | [CY041962](http://www.ncbi.nlm.nih.gov/entrez/viewer.fcgi?val=CY041962) | 1410 | Dominican Republic | 24-may |
|  | [GQ149677](http://www.ncbi.nlm.nih.gov/entrez/viewer.fcgi?val=GQ149677) | 1701 | Mexico | 14-Apr | 39 | [GQ149666](http://www.ncbi.nlm.nih.gov/entrez/viewer.fcgi?val=GQ149666) | 1381 | Mexico | 2-Apr |
|  | [GQ162190](http://www.ncbi.nlm.nih.gov/entrez/viewer.fcgi?val=GQ162190) | 1701 | Mexico | 14-Apr | 40 | [GQ476024](http://www.ncbi.nlm.nih.gov/entrez/viewer.fcgi?val=GQ476024) | 1413 | USA | 8-Apr |
|  | [GQ339881](http://www.ncbi.nlm.nih.gov/entrez/viewer.fcgi?val=GQ339881) | 1701 | Mexico | 15-Apr | 41 | [GQ377078](http://www.ncbi.nlm.nih.gov/entrez/viewer.fcgi?val=GQ377078) | 1410 | USA | 9-Apr |
|  | [GQ149630](http://www.ncbi.nlm.nih.gov/entrez/viewer.fcgi?val=GQ149630) | 1701 | Mexico | 19-Apr | 42 | [GQ476149](http://www.ncbi.nlm.nih.gov/entrez/viewer.fcgi?val=GQ476149) | 1413 | USA | 9-Apr |
|  | [GQ149634](http://www.ncbi.nlm.nih.gov/entrez/viewer.fcgi?val=GQ149634) | 1701 | Mexico | 19-Apr | 43 | [GQ162181](http://www.ncbi.nlm.nih.gov/entrez/viewer.fcgi?val=GQ162181) | 1410 | Mexico | 13-Apr |
|  | [GQ149684](http://www.ncbi.nlm.nih.gov/entrez/viewer.fcgi?val=GQ149684) | 1701 | Mexico | 19-Apr |  | [GQ168643](http://www.ncbi.nlm.nih.gov/entrez/viewer.fcgi?val=GQ168643) | 1410 | USA | 24-Apr |
|  | [GQ162191](http://www.ncbi.nlm.nih.gov/entrez/viewer.fcgi?val=GQ162191) | 1701 | Mexico | 20-Apr |  | [GQ232025](http://www.ncbi.nlm.nih.gov/entrez/viewer.fcgi?val=GQ232025) | 1410 | USA | 24-Apr |
|  | [GQ162197](http://www.ncbi.nlm.nih.gov/entrez/viewer.fcgi?val=GQ162197) | 1701 | Mexico | 20-Apr |  | [GQ323478](http://www.ncbi.nlm.nih.gov/entrez/viewer.fcgi?val=GQ323478) | 1410 | USA | 24-Apr |
|  | [GQ168620](http://www.ncbi.nlm.nih.gov/entrez/viewer.fcgi?val=GQ168620) | 1701 | USA | 26-Apr | 44 | [GQ476079](http://www.ncbi.nlm.nih.gov/entrez/viewer.fcgi?val=GQ476079) | 1413 | USA | 13-Apr |
|  | [GQ117112](http://www.ncbi.nlm.nih.gov/entrez/viewer.fcgi?val=GQ117112) | 1701 | USA | 26-Apr | 45 | [FJ998214](http://www.ncbi.nlm.nih.gov/entrez/viewer.fcgi?val=FJ998214) | 1415 | Mexico | 14-Apr |
|  | [GQ160605](http://www.ncbi.nlm.nih.gov/entrez/viewer.fcgi?val=GQ160605) | 1701 | USA | 27-Apr | 46 | [GQ149670](http://www.ncbi.nlm.nih.gov/entrez/viewer.fcgi?val=GQ149670) | 1410 | Mexico | 14-Apr |
|  | [GQ338414](http://www.ncbi.nlm.nih.gov/entrez/viewer.fcgi?val=GQ338414) | 1701 | USA | 27-Apr |  | [GQ149672](http://www.ncbi.nlm.nih.gov/entrez/viewer.fcgi?val=GQ149672) | 1410 | Mexico | 14-Apr |
|  | [GQ247724](http://www.ncbi.nlm.nih.gov/entrez/viewer.fcgi?val=GQ247724) | 1701 | Brazil | 29-Apr |  | [GQ162189](http://www.ncbi.nlm.nih.gov/entrez/viewer.fcgi?val=GQ162189) | 1410 | Mexico | 14-Apr |
|  | [GQ232035](http://www.ncbi.nlm.nih.gov/entrez/viewer.fcgi?val=GQ232035) | 1701 | USA | 04-may |  | [GQ379820](http://www.ncbi.nlm.nih.gov/entrez/viewer.fcgi?val=GQ379820) | 1410 | Mexico | 14-Apr |
|  | [GQ338361](http://www.ncbi.nlm.nih.gov/entrez/viewer.fcgi?val=GQ338361) | 1701 | USA | 07-may | 47 | [GQ162201](http://www.ncbi.nlm.nih.gov/entrez/viewer.fcgi?val=GQ162201) | 1410 | Mexico | 14-Apr |
|  | [GQ232060](http://www.ncbi.nlm.nih.gov/entrez/viewer.fcgi?val=GQ232060) | 1701 | USA | 11-may | 48 | [CY044237](http://www.ncbi.nlm.nih.gov/entrez/viewer.fcgi?val=CY044237) | 1410 | USA | 14-Apr |
|  | [CY046063](http://www.ncbi.nlm.nih.gov/entrez/viewer.fcgi?val=CY046063) | 1701 | Italy | 10-jun |  | [CY044253](http://www.ncbi.nlm.nih.gov/entrez/viewer.fcgi?val=CY044253) | 1410 | USA | 23-Apr |
|  | [GQ463200](http://www.ncbi.nlm.nih.gov/entrez/viewer.fcgi?val=GQ463200) | 1701 | China | 13-jun |  | [GQ323513](http://www.ncbi.nlm.nih.gov/entrez/viewer.fcgi?val=GQ323513) | 1410 | USA | 23-Apr |
|  | [GQ894816](http://www.ncbi.nlm.nih.gov/entrez/viewer.fcgi?val=GQ894816) | 1701 | USA | 13-jun |  | [GQ323573](http://www.ncbi.nlm.nih.gov/entrez/viewer.fcgi?val=GQ323573) | 1410 | USA | 23-Apr |
|  | [AB530249](http://www.ncbi.nlm.nih.gov/entrez/viewer.fcgi?val=AB530249) | 1701 | Japan | 13-jul | 49 | [FJ966969](http://www.ncbi.nlm.nih.gov/entrez/viewer.fcgi?val=FJ966969) | 1403 | USA | 15-Apr |
|  | [GQ221798](http://www.ncbi.nlm.nih.gov/entrez/viewer.fcgi?val=GQ221798) | 1701 | USA | 14-Apr | 50 | [GQ122096](http://www.ncbi.nlm.nih.gov/entrez/viewer.fcgi?val=GQ122096) | 1410 | USA | 15-Apr |
| 65 | [GQ303340](http://www.ncbi.nlm.nih.gov/entrez/viewer.fcgi?val=GQ303340) | 1701 | Mexico | 14-Apr | 51 | [GQ402234](http://www.ncbi.nlm.nih.gov/entrez/viewer.fcgi?val=GQ402234) | 1422 | Canada | 16-Apr |
| 66 | [GQ377072](http://www.ncbi.nlm.nih.gov/entrez/viewer.fcgi?val=GQ377072) | 1701 | USA | 14-Apr |  | [GQ465702](http://www.ncbi.nlm.nih.gov/entrez/viewer.fcgi?val=GQ465702) | 1422 | Canada | 01-may |
| 67 | [FJ966971](http://www.ncbi.nlm.nih.gov/entrez/viewer.fcgi?val=FJ966971) | 993 | USA | 15-Apr | 52 | [GQ476035](http://www.ncbi.nlm.nih.gov/entrez/viewer.fcgi?val=GQ476035) | 1413 | USA | 16-Apr |
| 68 | [GQ122097](http://www.ncbi.nlm.nih.gov/entrez/viewer.fcgi?val=GQ122097) | 1701 | USA | 15-Apr | 53 | [GQ149631](http://www.ncbi.nlm.nih.gov/entrez/viewer.fcgi?val=GQ149631) | 1410 | Mexico | 19-Apr |
| 69 | [GQ162172](http://www.ncbi.nlm.nih.gov/entrez/viewer.fcgi?val=GQ162172) | 1701 | Mexico | 15-Apr |  | [GU123908](http://www.ncbi.nlm.nih.gov/entrez/viewer.fcgi?val=GU123908) | 1410 | Italy | Jul |
| 70 | [GQ457487](http://www.ncbi.nlm.nih.gov/entrez/viewer.fcgi?val=GQ457487) | 1701 | USA | 15-Apr | 54 | [GQ149640](http://www.ncbi.nlm.nih.gov/entrez/viewer.fcgi?val=GQ149640) | 1410 | Mexico | 19-Apr |
| 71 | [GQ232076](http://www.ncbi.nlm.nih.gov/entrez/viewer.fcgi?val=GQ232076) | 1701 | USA | 23-Apr | 55 | [GQ149681](http://www.ncbi.nlm.nih.gov/entrez/viewer.fcgi?val=GQ149681) | 1410 | Mexico | 19-Apr |
|  | [CY044243](http://www.ncbi.nlm.nih.gov/entrez/viewer.fcgi?val=CY044243) | 1701 | USA | 15-Apr | 56 | [GQ162173](http://www.ncbi.nlm.nih.gov/entrez/viewer.fcgi?val=GQ162173) | 1410 | Mexico | 20-Apr |
| 72 | [FJ966960](http://www.ncbi.nlm.nih.gov/entrez/viewer.fcgi?val=FJ966960) | 1701 | USA | 16-Apr | 56 | [GQ168656](http://www.ncbi.nlm.nih.gov/entrez/viewer.fcgi?val=GQ168656) | 1410 | USA | Apr |
| 73 | [GQ402194](http://www.ncbi.nlm.nih.gov/entrez/viewer.fcgi?val=GQ402194) | 1743 | Canada | 16-Apr | 57 | [GQ162196](http://www.ncbi.nlm.nih.gov/entrez/viewer.fcgi?val=GQ162196) | 1410 | Mexico | 20-Apr |
| 74 | [GQ465677](http://www.ncbi.nlm.nih.gov/entrez/viewer.fcgi?val=GQ465677) | 1743 | Canada | 26-Apr |  | [GQ377037](http://www.ncbi.nlm.nih.gov/entrez/viewer.fcgi?val=GQ377037) | 1410 | USA | Apr |
|  | [GQ476034](http://www.ncbi.nlm.nih.gov/entrez/viewer.fcgi?val=GQ476034) | 1698 | USA | 16-Apr |  | [GQ396745](http://www.ncbi.nlm.nih.gov/entrez/viewer.fcgi?val=GQ396745) | 1410 | USA | 24-Apr |
| 75 | [GQ162195](http://www.ncbi.nlm.nih.gov/entrez/viewer.fcgi?val=GQ162195) | 1701 | Mexico | 17-Apr |  | [GQ894876](http://www.ncbi.nlm.nih.gov/entrez/viewer.fcgi?val=GQ894876) | 1410 | USA | 24-Apr |
| 76 | [GQ149641](http://www.ncbi.nlm.nih.gov/entrez/viewer.fcgi?val=GQ149641) | 1701 | Mexico | 19-Apr |  | [GQ117099](http://www.ncbi.nlm.nih.gov/entrez/viewer.fcgi?val=GQ117099) | 1410 | USA | 24-Apr |
| 77 | [GQ162183](http://www.ncbi.nlm.nih.gov/entrez/viewer.fcgi?val=GQ162183) | 1701 | Mexico | 20-Apr |  | [FJ984357](http://www.ncbi.nlm.nih.gov/entrez/viewer.fcgi?val=FJ984357) | 1410 | USA | 24-Apr |
| 78 | [GQ162200](http://www.ncbi.nlm.nih.gov/entrez/viewer.fcgi?val=GQ162200) | 1701 | Mexico | 20-Apr |  | [FJ984362](http://www.ncbi.nlm.nih.gov/entrez/viewer.fcgi?val=FJ984362) | 1410 | USA | 24-Apr |
| 79 | [GQ232010](http://www.ncbi.nlm.nih.gov/entrez/viewer.fcgi?val=GQ232010) | 1701 | USA | 21-Apr |  | [GQ117022](http://www.ncbi.nlm.nih.gov/entrez/viewer.fcgi?val=GQ117022) | 1410 | USA | 24-Apr |
| 80 | [GQ117097](http://www.ncbi.nlm.nih.gov/entrez/viewer.fcgi?val=GQ117097) | 1701 | USA | 22-Apr |  | [GQ168660](http://www.ncbi.nlm.nih.gov/entrez/viewer.fcgi?val=GQ168660) | 1410 | USA | 24-Apr |
| 81 | [GQ338349](http://www.ncbi.nlm.nih.gov/entrez/viewer.fcgi?val=GQ338349) | 1701 | USA | 24-Apr |  | [GQ168669](http://www.ncbi.nlm.nih.gov/entrez/viewer.fcgi?val=GQ168669) | 1410 | USA | 24-Apr |
|  | [GQ160606](http://www.ncbi.nlm.nih.gov/entrez/viewer.fcgi?val=GQ160606) | 1701 | USA | 22-Apr |  | [GQ200251](http://www.ncbi.nlm.nih.gov/entrez/viewer.fcgi?val=GQ200251) | 1410 | USA | 24-Apr |
| 82 | [GQ377093](http://www.ncbi.nlm.nih.gov/entrez/viewer.fcgi?val=GQ377093) | 1701 | USA | 22-Apr |  | [GQ323479](http://www.ncbi.nlm.nih.gov/entrez/viewer.fcgi?val=GQ323479) | 1410 | USA | 24-Apr |
| 83 | [CY043086](http://www.ncbi.nlm.nih.gov/entrez/viewer.fcgi?val=CY043086) | 1701 | USA | 22-Apr |  | [GQ323567](http://www.ncbi.nlm.nih.gov/entrez/viewer.fcgi?val=GQ323567) | 1410 | USA | 24-Apr |
| 84 | [GQ232002](http://www.ncbi.nlm.nih.gov/entrez/viewer.fcgi?val=GQ232002) | 1701 | USA | 23-Apr |  | [GQ323565](http://www.ncbi.nlm.nih.gov/entrez/viewer.fcgi?val=GQ323565) | 1410 | USA | 25-Apr |
| 85 | [CY044251](http://www.ncbi.nlm.nih.gov/entrez/viewer.fcgi?val=CY044251) | 1701 | USA | 23-Apr |  | [GQ338348](http://www.ncbi.nlm.nih.gov/entrez/viewer.fcgi?val=GQ338348) | 1410 | USA | 25-Apr |
| 86 | [FJ969521](http://www.ncbi.nlm.nih.gov/entrez/viewer.fcgi?val=FJ969521) | 985 | USA | 24-Apr |  | [GQ323481](http://www.ncbi.nlm.nih.gov/entrez/viewer.fcgi?val=GQ323481) | 1410 | USA | 25-Apr |
| 87 | [FJ969523](http://www.ncbi.nlm.nih.gov/entrez/viewer.fcgi?val=FJ969523) | 1002 | USA | 24-Apr |  | [GQ323482](http://www.ncbi.nlm.nih.gov/entrez/viewer.fcgi?val=GQ323482) | 1410 | USA | 25-Apr |
| 88 | [FJ969535](http://www.ncbi.nlm.nih.gov/entrez/viewer.fcgi?val=FJ969535) | 979 | USA | 24-Apr |  | [GQ323525](http://www.ncbi.nlm.nih.gov/entrez/viewer.fcgi?val=GQ323525) | 1410 | USA | 25-Apr |
| 89 | [FJ984360](http://www.ncbi.nlm.nih.gov/entrez/viewer.fcgi?val=FJ984360) | 1701 | USA | 24-Apr |  | [GQ221787](http://www.ncbi.nlm.nih.gov/entrez/viewer.fcgi?val=GQ221787) | 1410 | USA | 25-Apr |
| 90 | [GQ160566](http://www.ncbi.nlm.nih.gov/entrez/viewer.fcgi?val=GQ160566) | 1701 | USA | 1-Apr |  | [CY041124](http://www.ncbi.nlm.nih.gov/entrez/viewer.fcgi?val=CY041124) | 1410 | USA | 25-Apr |
|  | [GQ168657](http://www.ncbi.nlm.nih.gov/entrez/viewer.fcgi?val=GQ168657) | 1701 | USA | 2-Apr |  | [GQ168672](http://www.ncbi.nlm.nih.gov/entrez/viewer.fcgi?val=GQ168672) | 1410 | USA | 25-Apr |
|  | [GQ117024](http://www.ncbi.nlm.nih.gov/entrez/viewer.fcgi?val=GQ117024) | 1701 | USA | 24-Apr |  | [GQ117081](http://www.ncbi.nlm.nih.gov/entrez/viewer.fcgi?val=GQ117081) | 1410 | USA | 25-Apr |
|  | [GQ160601](http://www.ncbi.nlm.nih.gov/entrez/viewer.fcgi?val=GQ160601) | 1701 | USA | 24-Apr |  | [GQ117084](http://www.ncbi.nlm.nih.gov/entrez/viewer.fcgi?val=GQ117084) | 1410 | USA | 25-Apr |
|  | [GQ200250](http://www.ncbi.nlm.nih.gov/entrez/viewer.fcgi?val=GQ200250) | 1701 | USA | 24-Apr |  | [GQ117036](http://www.ncbi.nlm.nih.gov/entrez/viewer.fcgi?val=GQ117036) | 1410 | USA | 25-Apr |
|  | [GQ221814](http://www.ncbi.nlm.nih.gov/entrez/viewer.fcgi?val=GQ221814) | 1701 | USA | 24-Apr |  | [FJ984340](http://www.ncbi.nlm.nih.gov/entrez/viewer.fcgi?val=FJ984340) | 1410 | USA | 25-Apr |
|  | [GQ168617](http://www.ncbi.nlm.nih.gov/entrez/viewer.fcgi?val=GQ168617) | 1701 | USA | 24-Apr |  | [FJ984350](http://www.ncbi.nlm.nih.gov/entrez/viewer.fcgi?val=FJ984350) | 1410 | USA | 25-Apr |
|  | [GQ457497](http://www.ncbi.nlm.nih.gov/entrez/viewer.fcgi?val=GQ457497) | 1701 | USA | 25-Apr |  | [FJ984371](http://www.ncbi.nlm.nih.gov/entrez/viewer.fcgi?val=FJ984371) | 1410 | USA | 25-Apr |
|  | [GQ221786](http://www.ncbi.nlm.nih.gov/entrez/viewer.fcgi?val=GQ221786) | 1701 | USA | 25-Apr |  | [FJ984378](http://www.ncbi.nlm.nih.gov/entrez/viewer.fcgi?val=FJ984378) | 1410 | USA | 25-Apr |
|  | [GQ160602](http://www.ncbi.nlm.nih.gov/entrez/viewer.fcgi?val=GQ160602) | 1701 | USA | 25-Apr |  | [FJ984390](http://www.ncbi.nlm.nih.gov/entrez/viewer.fcgi?val=FJ984390) | 1410 | USA | 25-Apr |
|  | [GQ117040](http://www.ncbi.nlm.nih.gov/entrez/viewer.fcgi?val=GQ117040) | 1701 | USA | 25-Apr |  | [GQ117102](http://www.ncbi.nlm.nih.gov/entrez/viewer.fcgi?val=GQ117102) | 1410 | USA | 25-Apr |
|  | [GQ168851](http://www.ncbi.nlm.nih.gov/entrez/viewer.fcgi?val=GQ168851) | 1701 | USA | 25-Apr |  | [GQ117114](http://www.ncbi.nlm.nih.gov/entrez/viewer.fcgi?val=GQ117114) | 1410 | USA | 25-Apr |
|  | [GQ168886](http://www.ncbi.nlm.nih.gov/entrez/viewer.fcgi?val=GQ168886) | 1701 | USA | 25-Apr |  | [GQ168648](http://www.ncbi.nlm.nih.gov/entrez/viewer.fcgi?val=GQ168648) | 1410 | USA | 25-Apr |
|  | [FJ984375](http://www.ncbi.nlm.nih.gov/entrez/viewer.fcgi?val=FJ984375) | 1701 | USA | 25-Apr |  | [GQ168651](http://www.ncbi.nlm.nih.gov/entrez/viewer.fcgi?val=GQ168651) | 1410 | USA | 25-Apr |
|  | [FJ984394](http://www.ncbi.nlm.nih.gov/entrez/viewer.fcgi?val=FJ984394) | 1701 | USA | 25-Apr |  | [GQ168654](http://www.ncbi.nlm.nih.gov/entrez/viewer.fcgi?val=GQ168654) | 1410 | USA | 25-Apr |
|  | [FJ969509](http://www.ncbi.nlm.nih.gov/entrez/viewer.fcgi?val=FJ969509) | 1701 | USA | 25-Apr |  | [CY040639](http://www.ncbi.nlm.nih.gov/entrez/viewer.fcgi?val=CY040639) | 1410 | USA | 25-Apr |
|  | [FJ984347](http://www.ncbi.nlm.nih.gov/entrez/viewer.fcgi?val=FJ984347) | 1701 | USA | 25-Apr |  | [GQ457483](http://www.ncbi.nlm.nih.gov/entrez/viewer.fcgi?val=GQ457483) | 1410 | USA | 25-Apr |
|  | [FJ984355](http://www.ncbi.nlm.nih.gov/entrez/viewer.fcgi?val=FJ984355) | 1701 | USA | 25-Apr |  | [GQ377102](http://www.ncbi.nlm.nih.gov/entrez/viewer.fcgi?val=GQ377102) | 1410 | USA | 25-Apr |
|  | [GQ117082](http://www.ncbi.nlm.nih.gov/entrez/viewer.fcgi?val=GQ117082) | 1701 | USA | 25-Apr |  | [GQ377057](http://www.ncbi.nlm.nih.gov/entrez/viewer.fcgi?val=GQ377057) | 1410 | USA | 26-Apr |
|  | [GQ117086](http://www.ncbi.nlm.nih.gov/entrez/viewer.fcgi?val=GQ117086) | 1701 | USA | 25-Apr |  | [GQ457481](http://www.ncbi.nlm.nih.gov/entrez/viewer.fcgi?val=GQ457481) | 1410 | USA | 26-Apr |
|  | [GQ117103](http://www.ncbi.nlm.nih.gov/entrez/viewer.fcgi?val=GQ117103) | 1701 | USA | 25-Apr |  | [GQ117042](http://www.ncbi.nlm.nih.gov/entrez/viewer.fcgi?val=GQ117042) | 1410 | USA | 26-Apr |
|  | [GQ117116](http://www.ncbi.nlm.nih.gov/entrez/viewer.fcgi?val=GQ117116) | 1701 | USA | 25-Apr |  | [GQ160547](http://www.ncbi.nlm.nih.gov/entrez/viewer.fcgi?val=GQ160547) | 1410 | USA | 27-Apr |
|  | [GQ160543](http://www.ncbi.nlm.nih.gov/entrez/viewer.fcgi?val=GQ160543) | 1701 | USA | 25-Apr |  | [GQ160589](http://www.ncbi.nlm.nih.gov/entrez/viewer.fcgi?val=GQ160589) | 1410 | USA | 27-Apr |
|  | [CY040637](http://www.ncbi.nlm.nih.gov/entrez/viewer.fcgi?val=CY040637) | 1701 | USA | 25-Apr |  | [GQ200238](http://www.ncbi.nlm.nih.gov/entrez/viewer.fcgi?val=GQ200238) | 1410 | USA | 27-Apr |
|  | [GQ160556](http://www.ncbi.nlm.nih.gov/entrez/viewer.fcgi?val=GQ160556) | 1701 | USA | 25-Apr |  | [GQ323545](http://www.ncbi.nlm.nih.gov/entrez/viewer.fcgi?val=GQ323545) | 1410 | USA | 27-Apr |
|  | [GQ117043](http://www.ncbi.nlm.nih.gov/entrez/viewer.fcgi?val=GQ117043) | 1701 | USA | 26-Apr |  | [GQ338370](http://www.ncbi.nlm.nih.gov/entrez/viewer.fcgi?val=GQ338370) | 1410 | USA | 27-Apr |
|  | [GQ457482](http://www.ncbi.nlm.nih.gov/entrez/viewer.fcgi?val=GQ457482) | 1701 | USA | 26-Apr |  | [GQ323566](http://www.ncbi.nlm.nih.gov/entrez/viewer.fcgi?val=GQ323566) | 1410 | USA | 27-Apr |
|  | [GQ160591](http://www.ncbi.nlm.nih.gov/entrez/viewer.fcgi?val=GQ160591) | 1701 | USA | 27-Apr |  | [GQ232041](http://www.ncbi.nlm.nih.gov/entrez/viewer.fcgi?val=GQ232041) | 1410 | USA | 28-Apr |
|  | [GQ160599](http://www.ncbi.nlm.nih.gov/entrez/viewer.fcgi?val=GQ160599) | 1701 | USA | 27-Apr |  | [CY040856](http://www.ncbi.nlm.nih.gov/entrez/viewer.fcgi?val=CY040856) | 1410 | USA | 29-Apr |
|  | [GQ232023](http://www.ncbi.nlm.nih.gov/entrez/viewer.fcgi?val=GQ232023) | 1701 | USA | 27-Apr |  | [CY040864](http://www.ncbi.nlm.nih.gov/entrez/viewer.fcgi?val=CY040864) | 1410 | USA | 29-Apr |
|  | [GQ323564](http://www.ncbi.nlm.nih.gov/entrez/viewer.fcgi?val=GQ323564) | 1701 | USA | 28-Apr |  | [CY040647](http://www.ncbi.nlm.nih.gov/entrez/viewer.fcgi?val=CY040647) | 1410 | USA | 30-Apr |
|  | [GQ377043](http://www.ncbi.nlm.nih.gov/entrez/viewer.fcgi?val=GQ377043) | 1701 | USA | 04-may |  | [GQ323442](http://www.ncbi.nlm.nih.gov/entrez/viewer.fcgi?val=GQ323442) | 1410 | USA | 30-Apr |
|  | [GQ457511](http://www.ncbi.nlm.nih.gov/entrez/viewer.fcgi?val=GQ457511) | 1701 | USA | 05-may |  | [GQ323450](http://www.ncbi.nlm.nih.gov/entrez/viewer.fcgi?val=GQ323450) | 1410 | USA | 30-Apr |
|  | [GQ323455](http://www.ncbi.nlm.nih.gov/entrez/viewer.fcgi?val=GQ323455) | 1701 | USA | 11-may |  | [CY049861](http://www.ncbi.nlm.nih.gov/entrez/viewer.fcgi?val=CY049861) | 1410 | USA | 02-may |
|  | [GQ184630](http://www.ncbi.nlm.nih.gov/entrez/viewer.fcgi?val=GQ184630) | 1701 | Russia | 21-may |  | [GQ377040](http://www.ncbi.nlm.nih.gov/entrez/viewer.fcgi?val=GQ377040) | 1410 | USA | 04-may |
|  | [CY044204](http://www.ncbi.nlm.nih.gov/entrez/viewer.fcgi?val=CY044204) | 1701 | Taiwan | 22-may |  | [GQ323468](http://www.ncbi.nlm.nih.gov/entrez/viewer.fcgi?val=GQ323468) | 1410 | USA | 04-may |
|  | [GQ377047](http://www.ncbi.nlm.nih.gov/entrez/viewer.fcgi?val=GQ377047) | 1701 | USA | 22-may |  | [GQ232005](http://www.ncbi.nlm.nih.gov/entrez/viewer.fcgi?val=GQ232005) | 1410 | USA | 04-may |
|  | [CY053901](http://www.ncbi.nlm.nih.gov/entrez/viewer.fcgi?val=CY053901) | 1701 | Argentina | 27-may |  | [GQ457510](http://www.ncbi.nlm.nih.gov/entrez/viewer.fcgi?val=GQ457510) | 1410 | USA | 05-may |
|  | [GQ414765](http://www.ncbi.nlm.nih.gov/entrez/viewer.fcgi?val=GQ414765) | 1701 | Brazil | 29-may |  | [GQ329090](http://www.ncbi.nlm.nih.gov/entrez/viewer.fcgi?val=GQ329090) | 1410 | France | 06-may |
|  | [GQ414767](http://www.ncbi.nlm.nih.gov/entrez/viewer.fcgi?val=GQ414767) | 1701 | Brazil | 29-may |  | [GQ329108](http://www.ncbi.nlm.nih.gov/entrez/viewer.fcgi?val=GQ329108) | 1410 | France | 06-may |
|  | [CY053910](http://www.ncbi.nlm.nih.gov/entrez/viewer.fcgi?val=CY053910) | 1701 | Argentina | 01-jun |  | [GQ338406](http://www.ncbi.nlm.nih.gov/entrez/viewer.fcgi?val=GQ338406) | 1410 | USA | 08-may |
|  | [FN423713](http://www.ncbi.nlm.nih.gov/entrez/viewer.fcgi?val=FN423713) | 1701 | Luxembourg | 01-jun |  | [GQ323445](http://www.ncbi.nlm.nih.gov/entrez/viewer.fcgi?val=GQ323445) | 1410 | USA | 09-may |
|  | [GQ329070](http://www.ncbi.nlm.nih.gov/entrez/viewer.fcgi?val=GQ329070) | 1701 | France | 02-jun |  | [GQ323475](http://www.ncbi.nlm.nih.gov/entrez/viewer.fcgi?val=GQ323475) | 1410 | USA | 10-may |
|  | [GQ334346](http://www.ncbi.nlm.nih.gov/entrez/viewer.fcgi?val=GQ334346) | 1701 | Japan | 02-jun |  | [GQ183634](http://www.ncbi.nlm.nih.gov/entrez/viewer.fcgi?val=GQ183634) | 1410 | Finland | 10-may |
|  | [GU014776](http://www.ncbi.nlm.nih.gov/entrez/viewer.fcgi?val=GU014776) | 1701 | Japan | 02-jun |  | [GQ200288](http://www.ncbi.nlm.nih.gov/entrez/viewer.fcgi?val=GQ200288) | 1410 | China | 10-may |
|  | [GQ255901](http://www.ncbi.nlm.nih.gov/entrez/viewer.fcgi?val=GQ255901) | 1701 | Russia | 04-jun |  | [GQ323477](http://www.ncbi.nlm.nih.gov/entrez/viewer.fcgi?val=GQ323477) | 1410 | USA | 15-may |
|  | [GQ396732](http://www.ncbi.nlm.nih.gov/entrez/viewer.fcgi?val=GQ396732) | 1701 | USA | 05-jun |  | [GQ377067](http://www.ncbi.nlm.nih.gov/entrez/viewer.fcgi?val=GQ377067) | 1410 | USA | 18-may |
|  | [GQ368667](http://www.ncbi.nlm.nih.gov/entrez/viewer.fcgi?val=GQ368667) | 1701 | Brazil | 05-jun |  | [CY044198](http://www.ncbi.nlm.nih.gov/entrez/viewer.fcgi?val=CY044198) | 1410 | Taiwan | 19-may |
|  | [GQ287623](http://www.ncbi.nlm.nih.gov/entrez/viewer.fcgi?val=GQ287623) | 1701 | Japan | 06-jun |  | [CY044206](http://www.ncbi.nlm.nih.gov/entrez/viewer.fcgi?val=CY044206) | 1410 | Taiwan | 22-may |
|  | [GQ287627](http://www.ncbi.nlm.nih.gov/entrez/viewer.fcgi?val=GQ287627) | 1701 | Japan | 09-jun |  | [CY044214](http://www.ncbi.nlm.nih.gov/entrez/viewer.fcgi?val=CY044214) | 1410 | Taiwan | 22-may |
|  | [GQ365426](http://www.ncbi.nlm.nih.gov/entrez/viewer.fcgi?val=GQ365426) | 1701 | Japan | 09-jun |  | [GQ329084](http://www.ncbi.nlm.nih.gov/entrez/viewer.fcgi?val=GQ329084) | 1410 | France | 22-may |
|  | [CY052350](http://www.ncbi.nlm.nih.gov/entrez/viewer.fcgi?val=CY052350) | 1701 | Brazil | 10-jun |  | [GQ377046](http://www.ncbi.nlm.nih.gov/entrez/viewer.fcgi?val=GQ377046) | 1410 | USA | 22-may |
|  | [GQ365410](http://www.ncbi.nlm.nih.gov/entrez/viewer.fcgi?val=GQ365410) | 1701 | Japan | 11-jun |  | [GQ338336](http://www.ncbi.nlm.nih.gov/entrez/viewer.fcgi?val=GQ338336) | 1410 | USA | 23-may |
|  | [GU014792](http://www.ncbi.nlm.nih.gov/entrez/viewer.fcgi?val=GU014792) | 1701 | Japan | 12-jun |  | [GQ225359](http://www.ncbi.nlm.nih.gov/entrez/viewer.fcgi?val=GQ225359) | 1410 | China | 23-may |
|  | [GQ457465](http://www.ncbi.nlm.nih.gov/entrez/viewer.fcgi?val=GQ457465) | 1701 | USA | 14-jun |  | [GQ253495](http://www.ncbi.nlm.nih.gov/entrez/viewer.fcgi?val=GQ253495) | 1410 | China | 24-may |
|  | [GQ457470](http://www.ncbi.nlm.nih.gov/entrez/viewer.fcgi?val=GQ457470) | 1701 | USA | 14-jun |  | [GU065289](http://www.ncbi.nlm.nih.gov/entrez/viewer.fcgi?val=GU065289) | 1410 | Poland | 26-may |
|  | [GU014774](http://www.ncbi.nlm.nih.gov/entrez/viewer.fcgi?val=GU014774) | 1701 | Japan | 16-jun |  | [CY049933](http://www.ncbi.nlm.nih.gov/entrez/viewer.fcgi?val=CY049933) | 1410 | USA | 27-may |
|  | [CY053965](http://www.ncbi.nlm.nih.gov/entrez/viewer.fcgi?val=CY053965) | 1701 | Argentina | 16-jun |  | [GQ283481](http://www.ncbi.nlm.nih.gov/entrez/viewer.fcgi?val=GQ283481) | 1410 | Italy | 27-may |
|  | [CY053936](http://www.ncbi.nlm.nih.gov/entrez/viewer.fcgi?val=CY053936) | 1701 | Argentina | 17-jun |  | [GQ368666](http://www.ncbi.nlm.nih.gov/entrez/viewer.fcgi?val=GQ368666) | 1410 | Brazil | 28-may |
|  | [CY053942](http://www.ncbi.nlm.nih.gov/entrez/viewer.fcgi?val=CY053942) | 1701 | Argentina | 24-jun |  | [GQ377105](http://www.ncbi.nlm.nih.gov/entrez/viewer.fcgi?val=GQ377105) | 1410 | USA | 31-may |
|  | [CY053968](http://www.ncbi.nlm.nih.gov/entrez/viewer.fcgi?val=CY053968) | 1701 | Argentina | 25-jun |  | [FN423714](http://www.ncbi.nlm.nih.gov/entrez/viewer.fcgi?val=FN423714) | 1410 | Luxembourg | 01-jun |
|  | [CY053956](http://www.ncbi.nlm.nih.gov/entrez/viewer.fcgi?val=CY053956) | 1701 | Argentina | 26-jun |  | [GQ329072](http://www.ncbi.nlm.nih.gov/entrez/viewer.fcgi?val=GQ329072) | 1410 | France | 02-jun |
|  | [GU014768](http://www.ncbi.nlm.nih.gov/entrez/viewer.fcgi?val=GU014768) | 1701 | Japan | 29-jun |  | [GQ287626](http://www.ncbi.nlm.nih.gov/entrez/viewer.fcgi?val=GQ287626) | 1410 | Japan | 02-jun |
|  | [CY049995](http://www.ncbi.nlm.nih.gov/entrez/viewer.fcgi?val=CY049995) | 1701 | Mexico | 29-jun |  | [GQ396731](http://www.ncbi.nlm.nih.gov/entrez/viewer.fcgi?val=GQ396731) | 1410 | USA | 05-jun |
|  | [GU014784](http://www.ncbi.nlm.nih.gov/entrez/viewer.fcgi?val=GU014784) | 1701 | Japan | 30-jun |  | [GQ457469](http://www.ncbi.nlm.nih.gov/entrez/viewer.fcgi?val=GQ457469) | 1410 | USA | 14-jun |
|  | [CY053913](http://www.ncbi.nlm.nih.gov/entrez/viewer.fcgi?val=CY053913) | 1701 | Argentina | 01-jul |  | [CY049925](http://www.ncbi.nlm.nih.gov/entrez/viewer.fcgi?val=CY049925) | 1410 | USA | 14-jun |
|  | [GU014754](http://www.ncbi.nlm.nih.gov/entrez/viewer.fcgi?val=GU014754) | 1701 | Japan | 21-jul |  | [GU014799](http://www.ncbi.nlm.nih.gov/entrez/viewer.fcgi?val=GU014799) | 1410 | Japan | 15-jun |
|  | [CY054282](http://www.ncbi.nlm.nih.gov/entrez/viewer.fcgi?val=CY054282) | 1701 | Brazil | 29-jul |  | [CY053966](http://www.ncbi.nlm.nih.gov/entrez/viewer.fcgi?val=CY053966) | 1410 | Argentina | 16-jun |
|  | [GU220604](http://www.ncbi.nlm.nih.gov/entrez/viewer.fcgi?val=GU220604) | 1701 | Serbia | 4-Aug |  | [GU014775](http://www.ncbi.nlm.nih.gov/entrez/viewer.fcgi?val=GU014775) | 1410 | Japan | 16-jun |
|  | [CY050035](http://www.ncbi.nlm.nih.gov/entrez/viewer.fcgi?val=CY050035) | 1701 | Mexico | 04-sep |  | [GQ328768](http://www.ncbi.nlm.nih.gov/entrez/viewer.fcgi?val=GQ328768) | 1410 | Greece | 16-jun |
|  | [FJ984364](http://www.ncbi.nlm.nih.gov/entrez/viewer.fcgi?val=FJ984364) | 1701 | USA | 24-Apr |  | [GU014751](http://www.ncbi.nlm.nih.gov/entrez/viewer.fcgi?val=GU014751) | 1410 | Japan | 17-jun |
| 91 | [GQ168628](http://www.ncbi.nlm.nih.gov/entrez/viewer.fcgi?val=GQ168628) | 1701 | USA | 24-Apr |  | [GU014779](http://www.ncbi.nlm.nih.gov/entrez/viewer.fcgi?val=GU014779) | 1410 | Japan | 18-jun |
|  | [FJ984397](http://www.ncbi.nlm.nih.gov/entrez/viewer.fcgi?val=FJ984397) | 1701 | USA | 24-Apr |  | [GQ365430](http://www.ncbi.nlm.nih.gov/entrez/viewer.fcgi?val=GQ365430) | 1410 | Japan | 18-jun |
| 92 | [FJ984401](http://www.ncbi.nlm.nih.gov/entrez/viewer.fcgi?val=FJ984401) | 1701 | USA | 24-Apr |  | [CY043120](http://www.ncbi.nlm.nih.gov/entrez/viewer.fcgi?val=CY043120) | 1410 | USA | 18-jun |
|  | [GQ338368](http://www.ncbi.nlm.nih.gov/entrez/viewer.fcgi?val=GQ338368) | 1701 | USA | 27-Apr |  | [CY044181](http://www.ncbi.nlm.nih.gov/entrez/viewer.fcgi?val=CY044181) | 1410 | USA | 18-jun |
|  | [GQ338405](http://www.ncbi.nlm.nih.gov/entrez/viewer.fcgi?val=GQ338405) | 1701 | USA | 08-may |  | [CY044189](http://www.ncbi.nlm.nih.gov/entrez/viewer.fcgi?val=CY044189) | 1410 | USA | 18-jun |
|  | [GQ225357](http://www.ncbi.nlm.nih.gov/entrez/viewer.fcgi?val=GQ225357) | 1701 | China | 23-may |  | [GU014811](http://www.ncbi.nlm.nih.gov/entrez/viewer.fcgi?val=GU014811) | 1410 | Japan | 22-jun |
|  | [GQ253492](http://www.ncbi.nlm.nih.gov/entrez/viewer.fcgi?val=GQ253492) | 1701 | China | 24-may |  | [GU014801](http://www.ncbi.nlm.nih.gov/entrez/viewer.fcgi?val=GU014801) | 1410 | Japan | 23-jun |
|  | [FJ998207](http://www.ncbi.nlm.nih.gov/entrez/viewer.fcgi?val=FJ998207) | 1743 | Canada | 24-Apr |  | [GQ365420](http://www.ncbi.nlm.nih.gov/entrez/viewer.fcgi?val=GQ365420) | 1410 | Japan | 23-jun |
| 93 | [GQ465674](http://www.ncbi.nlm.nih.gov/entrez/viewer.fcgi?val=GQ465674) | 1743 | Canada | 29-Apr |  | [CY049997](http://www.ncbi.nlm.nih.gov/entrez/viewer.fcgi?val=CY049997) | 1410 | Mexico | 29-jun |
|  | [GQ465675](http://www.ncbi.nlm.nih.gov/entrez/viewer.fcgi?val=GQ465675) | 1743 | Canada | 29-Apr |  | [GU014769](http://www.ncbi.nlm.nih.gov/entrez/viewer.fcgi?val=GU014769) | 1410 | Japan | 29-jun |
|  | [GQ465676](http://www.ncbi.nlm.nih.gov/entrez/viewer.fcgi?val=GQ465676) | 1743 | Canada | 30-Apr |  | [GU014785](http://www.ncbi.nlm.nih.gov/entrez/viewer.fcgi?val=GU014785) | 1410 | Japan | 30-jun |
|  | [FJ998209](http://www.ncbi.nlm.nih.gov/entrez/viewer.fcgi?val=FJ998209) | 1743 | Canada | 24-Apr |  | [GU123911](http://www.ncbi.nlm.nih.gov/entrez/viewer.fcgi?val=GU123911) | 1410 | Italy | Jul |
| 94 | [GQ465678](http://www.ncbi.nlm.nih.gov/entrez/viewer.fcgi?val=GQ465678) | 1743 | Canada | 27-Apr |  | [GU123915](http://www.ncbi.nlm.nih.gov/entrez/viewer.fcgi?val=GU123915) | 1410 | Italy | Jul |
|  | [GU290047](http://www.ncbi.nlm.nih.gov/entrez/viewer.fcgi?val=GU290047) | 1743 | Czech Republic | 27-jun |  | [GU134720](http://www.ncbi.nlm.nih.gov/entrez/viewer.fcgi?val=GU134720) | 1410 | Italy | Jul |
|  | [GQ117062](http://www.ncbi.nlm.nih.gov/entrez/viewer.fcgi?val=GQ117062) | 1701 | USA | 24-Apr |  | [GU014783](http://www.ncbi.nlm.nih.gov/entrez/viewer.fcgi?val=GU014783) | 1410 | Japan | 01-jul |
| 95 | [GQ117100](http://www.ncbi.nlm.nih.gov/entrez/viewer.fcgi?val=GQ117100) | 1701 | USA | 24-Apr |  | [GU014787](http://www.ncbi.nlm.nih.gov/entrez/viewer.fcgi?val=GU014787) | 1410 | Japan | 03-jul |
| 96 | [GQ168633](http://www.ncbi.nlm.nih.gov/entrez/viewer.fcgi?val=GQ168633) | 1701 | USA | 24-Apr |  | [CY050085](http://www.ncbi.nlm.nih.gov/entrez/viewer.fcgi?val=CY050085) | 1410 | Taiwan | 10-jul |
| 97 | [GQ168642](http://www.ncbi.nlm.nih.gov/entrez/viewer.fcgi?val=GQ168642) | 1701 | USA | 24-Apr |  | [GU014771](http://www.ncbi.nlm.nih.gov/entrez/viewer.fcgi?val=GU014771) | 1410 | Japan | 13-jul |
| 98 | [GQ168644](http://www.ncbi.nlm.nih.gov/entrez/viewer.fcgi?val=GQ168644) | 1701 | USA | 24-Apr |  | [GU014759](http://www.ncbi.nlm.nih.gov/entrez/viewer.fcgi?val=GU014759) | 1410 | Myanmar | 14-jul |
| 99 | [GQ168661](http://www.ncbi.nlm.nih.gov/entrez/viewer.fcgi?val=GQ168661) | 1701 | USA | 24-Apr |  | [GU014761](http://www.ncbi.nlm.nih.gov/entrez/viewer.fcgi?val=GU014761) | 1410 | Myanmar | 22-jul |
| 100 | [GQ221818](http://www.ncbi.nlm.nih.gov/entrez/viewer.fcgi?val=GQ221818) | 1701 | USA | 24-Apr |  | [GU014763](http://www.ncbi.nlm.nih.gov/entrez/viewer.fcgi?val=GU014763) | 1410 | Myanmar | 22-jul |
| 101 | [GQ232019](http://www.ncbi.nlm.nih.gov/entrez/viewer.fcgi?val=GQ232019) | 1701 | USA | 24-Apr |  | [CY050021](http://www.ncbi.nlm.nih.gov/entrez/viewer.fcgi?val=CY050021) | 1410 | Mexico | 02-sep |
| 102 | [GQ232049](http://www.ncbi.nlm.nih.gov/entrez/viewer.fcgi?val=GQ232049) | 1701 | USA | 24-Apr | 58 | [GQ397116](http://www.ncbi.nlm.nih.gov/entrez/viewer.fcgi?val=GQ397116) | 1410 | Mexico | 20-Apr |
| 103 | [GQ160542](http://www.ncbi.nlm.nih.gov/entrez/viewer.fcgi?val=GQ160542) | 1701 | USA | Apr |  | [GQ368663](http://www.ncbi.nlm.nih.gov/entrez/viewer.fcgi?val=GQ368663) | 1410 | Brazil | 29-Apr |
|  | [GQ168650](http://www.ncbi.nlm.nih.gov/entrez/viewer.fcgi?val=GQ168650) | 1701 | USA | 25-Apr |  | [CY049989](http://www.ncbi.nlm.nih.gov/entrez/viewer.fcgi?val=CY049989) | 1410 | Mexico | 19-jun |
|  | [GQ168668](http://www.ncbi.nlm.nih.gov/entrez/viewer.fcgi?val=GQ168668) | 1701 | USA | 25-Apr | 59 | [GQ397117](http://www.ncbi.nlm.nih.gov/entrez/viewer.fcgi?val=GQ397117) | 1410 | Mexico | 20-Apr |
|  | [GQ231999](http://www.ncbi.nlm.nih.gov/entrez/viewer.fcgi?val=GQ231999) | 1701 | USA | 25-Apr | 60 | [GQ894939](http://www.ncbi.nlm.nih.gov/entrez/viewer.fcgi?val=GQ894939) | 1410 | Mexico | 20-Apr |
|  | [GQ232064](http://www.ncbi.nlm.nih.gov/entrez/viewer.fcgi?val=GQ232064) | 1701 | USA | 25-Apr | 61 | [GQ323523](http://www.ncbi.nlm.nih.gov/entrez/viewer.fcgi?val=GQ323523) | 1410 | USA | 21-Apr |
|  | [CY041549](http://www.ncbi.nlm.nih.gov/entrez/viewer.fcgi?val=CY041549) | 1736 | USA | 24-Apr | 62 | [CY043096](http://www.ncbi.nlm.nih.gov/entrez/viewer.fcgi?val=CY043096) | 1410 | USA | 21-Apr |
| 104 | [CY041573](http://www.ncbi.nlm.nih.gov/entrez/viewer.fcgi?val=CY041573) | 1736 | USA | 28-Apr | 63 | [GQ117064](http://www.ncbi.nlm.nih.gov/entrez/viewer.fcgi?val=GQ117064) | 1410 | USA | 22-Apr |
|  | [CY041490](http://www.ncbi.nlm.nih.gov/entrez/viewer.fcgi?val=CY041490) | 1736 | USA | 28-Apr | 64 | [GQ117094](http://www.ncbi.nlm.nih.gov/entrez/viewer.fcgi?val=GQ117094) | 1410 | USA | 22-Apr |
|  | [CY041074](http://www.ncbi.nlm.nih.gov/entrez/viewer.fcgi?val=CY041074) | 1736 | USA | 02-may | 65 | [GQ377092](http://www.ncbi.nlm.nih.gov/entrez/viewer.fcgi?val=GQ377092) | 1410 | USA | 22-Apr |
|  | [FJ974026](http://www.ncbi.nlm.nih.gov/entrez/viewer.fcgi?val=FJ974026) | 1773 | Canada | 24-Apr |  | [GQ457516](http://www.ncbi.nlm.nih.gov/entrez/viewer.fcgi?val=GQ457516) | 1410 | USA | 01-may |
| 105 | [CY052044](http://www.ncbi.nlm.nih.gov/entrez/viewer.fcgi?val=CY052044) | 972 | Brazil | 24-Apr | 66 | [FJ969520](http://www.ncbi.nlm.nih.gov/entrez/viewer.fcgi?val=FJ969520) | 1387 | USA | 24-Apr |
| 106 | [FJ969542](http://www.ncbi.nlm.nih.gov/entrez/viewer.fcgi?val=FJ969542) | 984 | USA | 25-Apr |  | [FJ969541](http://www.ncbi.nlm.nih.gov/entrez/viewer.fcgi?val=FJ969541) | 1387 | USA | 25-Apr |
| 107 | [FJ973557](http://www.ncbi.nlm.nih.gov/entrez/viewer.fcgi?val=FJ973557) | 1576 | New Zealand | 25-Apr | 67 | [FJ969534](http://www.ncbi.nlm.nih.gov/entrez/viewer.fcgi?val=FJ969534) | 1407 | USA | 24-Apr |
| 108 | [FJ984337](http://www.ncbi.nlm.nih.gov/entrez/viewer.fcgi?val=FJ984337) | 1701 | USA | 25-Apr | 68 | [FJ998213](http://www.ncbi.nlm.nih.gov/entrez/viewer.fcgi?val=FJ998213) | 1421 | Canada | 24-Apr |
| 109 | [GQ117032](http://www.ncbi.nlm.nih.gov/entrez/viewer.fcgi?val=GQ117032) | 1701 | USA | 25-Apr | 69 | [FJ998215](http://www.ncbi.nlm.nih.gov/entrez/viewer.fcgi?val=FJ998215) | 1421 | Canada | 24-Apr |
| 110 | [GQ117091](http://www.ncbi.nlm.nih.gov/entrez/viewer.fcgi?val=GQ117091) | 1701 | USA | 25-Apr | 70 | [GQ117058](http://www.ncbi.nlm.nih.gov/entrez/viewer.fcgi?val=GQ117058) | 1410 | USA | 24-Apr |
| 111 | [GQ160613](http://www.ncbi.nlm.nih.gov/entrez/viewer.fcgi?val=GQ160613) | 1687 | New Zealand | 25-Apr |  | [GQ168670](http://www.ncbi.nlm.nih.gov/entrez/viewer.fcgi?val=GQ168670) | 1410 | USA | 25-Apr |
| 112 | [GQ160527](http://www.ncbi.nlm.nih.gov/entrez/viewer.fcgi?val=GQ160527) | 1701 | USA | 25-Apr |  | [GQ160571](http://www.ncbi.nlm.nih.gov/entrez/viewer.fcgi?val=GQ160571) | 1410 | USA | 26-Apr |
| 113 | [GQ160567](http://www.ncbi.nlm.nih.gov/entrez/viewer.fcgi?val=GQ160567) | 1701 | USA | 25-Apr | 71 | [GQ168627](http://www.ncbi.nlm.nih.gov/entrez/viewer.fcgi?val=GQ168627) | 1410 | USA | 24-Apr |
| 114 | [GQ168652](http://www.ncbi.nlm.nih.gov/entrez/viewer.fcgi?val=GQ168652) | 1701 | USA | 25-Apr | 72 | [GQ221815](http://www.ncbi.nlm.nih.gov/entrez/viewer.fcgi?val=GQ221815) | 1410 | USA | 24-Apr |
| 115 | [GQ168655](http://www.ncbi.nlm.nih.gov/entrez/viewer.fcgi?val=GQ168655) | 1701 | USA | 25-Apr | 73 | [CY041551](http://www.ncbi.nlm.nih.gov/entrez/viewer.fcgi?val=CY041551) | 1414 | USA | 24-Apr |
| 116 | [GQ232017](http://www.ncbi.nlm.nih.gov/entrez/viewer.fcgi?val=GQ232017) | 1701 | USA | 25-Apr |  | [CY041172](http://www.ncbi.nlm.nih.gov/entrez/viewer.fcgi?val=CY041172) | 1414 | USA | 26-Apr |
|  | [GQ168673](http://www.ncbi.nlm.nih.gov/entrez/viewer.fcgi?val=GQ168673) | 1701 | USA | 25-Apr |  | [CY041532](http://www.ncbi.nlm.nih.gov/entrez/viewer.fcgi?val=CY041532) | 1414 | USA | 26-Apr |
| 117 | [GQ169532](http://www.ncbi.nlm.nih.gov/entrez/viewer.fcgi?val=GQ169532) | 886 | Colombia | 25-Apr |  | [CY041736](http://www.ncbi.nlm.nih.gov/entrez/viewer.fcgi?val=CY041736) | 1414 | USA | 26-Apr |
| 118 | [GQ205434](http://www.ncbi.nlm.nih.gov/entrez/viewer.fcgi?val=GQ205434) | 1688 | New Zealand | 25-Apr |  | [CY041092](http://www.ncbi.nlm.nih.gov/entrez/viewer.fcgi?val=CY041092) | 1414 | USA | 28-Apr |
| 119 | [GQ205436](http://www.ncbi.nlm.nih.gov/entrez/viewer.fcgi?val=GQ205436) | 1688 | New Zealand | 25-Apr |  | [CY041647](http://www.ncbi.nlm.nih.gov/entrez/viewer.fcgi?val=CY041647) | 1414 | USA | 04-may |
|  | [GQ205438](http://www.ncbi.nlm.nih.gov/entrez/viewer.fcgi?val=GQ205438) | 1688 | New Zealand | 25-Apr |  | [CY041784](http://www.ncbi.nlm.nih.gov/entrez/viewer.fcgi?val=CY041784) | 1414 | USA | 04-may |
|  | [CY040888](http://www.ncbi.nlm.nih.gov/entrez/viewer.fcgi?val=CY040888) | 1701 | Mexico | 25-Apr |  | [CY043205](http://www.ncbi.nlm.nih.gov/entrez/viewer.fcgi?val=CY043205) | 1414 | USA | 04-may |
| 120 | [CY044163](http://www.ncbi.nlm.nih.gov/entrez/viewer.fcgi?val=CY044163) | 1701 | Mexico | 25-Apr |  | [CY043213](http://www.ncbi.nlm.nih.gov/entrez/viewer.fcgi?val=CY043213) | 1414 | USA | 13-may |
|  | [CY041122](http://www.ncbi.nlm.nih.gov/entrez/viewer.fcgi?val=CY041122) | 1701 | USA | 25-Apr |  | [CY041776](http://www.ncbi.nlm.nih.gov/entrez/viewer.fcgi?val=CY041776) | 1414 | USA | 16-may |
| 121 | [CY041130](http://www.ncbi.nlm.nih.gov/entrez/viewer.fcgi?val=CY041130) | 1705 | USA | 25-Apr |  | [CY044887](http://www.ncbi.nlm.nih.gov/entrez/viewer.fcgi?val=CY044887) | 1414 | USA | 19-may |
| 122 | [GQ231995](http://www.ncbi.nlm.nih.gov/entrez/viewer.fcgi?val=GQ231995) | 1701 | USA | 25-Apr |  | [CY044919](http://www.ncbi.nlm.nih.gov/entrez/viewer.fcgi?val=CY044919) | 1414 | USA | 19-may |
| 123 | [GQ231996](http://www.ncbi.nlm.nih.gov/entrez/viewer.fcgi?val=GQ231996) | 1701 | USA | 25-Apr |  | [CY043269](http://www.ncbi.nlm.nih.gov/entrez/viewer.fcgi?val=CY043269) | 1414 | USA | 19-may |
| 124 | [GQ232038](http://www.ncbi.nlm.nih.gov/entrez/viewer.fcgi?val=GQ232038) | 1701 | USA | 25-Apr |  | [CY044871](http://www.ncbi.nlm.nih.gov/entrez/viewer.fcgi?val=CY044871) | 1414 | USA | 20-may |
| 125 | [CY041541](http://www.ncbi.nlm.nih.gov/entrez/viewer.fcgi?val=CY041541) | 1737 | USA | 25-Apr |  | [CY044951](http://www.ncbi.nlm.nih.gov/entrez/viewer.fcgi?val=CY044951) | 1414 | USA | 22-may |
| 126 | [CY041557](http://www.ncbi.nlm.nih.gov/entrez/viewer.fcgi?val=CY041557) | 1737 | USA | 28-Apr |  | [CY045169](http://www.ncbi.nlm.nih.gov/entrez/viewer.fcgi?val=CY045169) | 1414 | USA | 02-jun |
|  | [CY041613](http://www.ncbi.nlm.nih.gov/entrez/viewer.fcgi?val=CY041613) | 1727 | USA | 25-Apr |  | [CY044114](http://www.ncbi.nlm.nih.gov/entrez/viewer.fcgi?val=CY044114) | 1414 | USA | 10-jun |
| 127 | [GQ377061](http://www.ncbi.nlm.nih.gov/entrez/viewer.fcgi?val=GQ377061) | 1701 | USA | 25-Apr | 74 | [GQ373263](http://www.ncbi.nlm.nih.gov/entrez/viewer.fcgi?val=GQ373263) | 1456 | Canada | 24-Apr |
| 128 | [GQ377103](http://www.ncbi.nlm.nih.gov/entrez/viewer.fcgi?val=GQ377103) | 1701 | USA | 25-Apr | 75 | [GQ396740](http://www.ncbi.nlm.nih.gov/entrez/viewer.fcgi?val=GQ396740) | 1410 | USA | 24-Apr |
| 129 | [FJ985753](http://www.ncbi.nlm.nih.gov/entrez/viewer.fcgi?val=FJ985753) | 949 | Spain | 26-Apr | 76 | [FJ973552](http://www.ncbi.nlm.nih.gov/entrez/viewer.fcgi?val=FJ973552) | 1341 | New Zealand | 25-Apr |
| 130 | [FJ985768](http://www.ncbi.nlm.nih.gov/entrez/viewer.fcgi?val=FJ985768) | 949 | Spain | 26-Apr | 77 | [FJ973555](http://www.ncbi.nlm.nih.gov/entrez/viewer.fcgi?val=FJ973555) | 1345 | New Zealand | 25-Apr |
|  | [GQ396529](http://www.ncbi.nlm.nih.gov/entrez/viewer.fcgi?val=GQ396529) | 949 | Spain | 28-Apr | 78 | [FJ984335](http://www.ncbi.nlm.nih.gov/entrez/viewer.fcgi?val=FJ984335) | 1410 | USA | 25-Apr |
|  | [FJ985758](http://www.ncbi.nlm.nih.gov/entrez/viewer.fcgi?val=FJ985758) | 948 | Spain | 26-Apr | 79 | [FJ984344](http://www.ncbi.nlm.nih.gov/entrez/viewer.fcgi?val=FJ984344) | 1410 | USA | 25-Apr |
| 131 | [GQ396579](http://www.ncbi.nlm.nih.gov/entrez/viewer.fcgi?val=GQ396579) | 948 | Spain | 29-Apr | 80 | [GQ117028](http://www.ncbi.nlm.nih.gov/entrez/viewer.fcgi?val=GQ117028) | 1410 | USA | 25-Apr |
| 132 | [FJ985763](http://www.ncbi.nlm.nih.gov/entrez/viewer.fcgi?val=FJ985763) | 949 | Spain | 26-Apr | 81 | [GQ160612](http://www.ncbi.nlm.nih.gov/entrez/viewer.fcgi?val=GQ160612) | 1396 | New Zealand | 25-Apr |
| 133 | [GQ117056](http://www.ncbi.nlm.nih.gov/entrez/viewer.fcgi?val=GQ117056) | 1701 | USA | 26-Apr |  | [GQ205437](http://www.ncbi.nlm.nih.gov/entrez/viewer.fcgi?val=GQ205437) | 1396 | New Zealand | 25-Apr |
| 134 | [GQ232054](http://www.ncbi.nlm.nih.gov/entrez/viewer.fcgi?val=GQ232054) | 1701 | USA | 30-Apr |  | [GQ168666](http://www.ncbi.nlm.nih.gov/entrez/viewer.fcgi?val=GQ168666) | 1410 | USA | 25-Apr |
|  | [GQ232070](http://www.ncbi.nlm.nih.gov/entrez/viewer.fcgi?val=GQ232070) | 1701 | USA | 04-may | 82 | [GQ205435](http://www.ncbi.nlm.nih.gov/entrez/viewer.fcgi?val=GQ205435) | 1397 | New Zealand | 25-Apr |
|  | [GQ117079](http://www.ncbi.nlm.nih.gov/entrez/viewer.fcgi?val=GQ117079) | 1701 | USA | 26-Apr |  | [GQ205439](http://www.ncbi.nlm.nih.gov/entrez/viewer.fcgi?val=GQ205439) | 1397 | New Zealand | 25-Apr |
| 135 | [GQ221788](http://www.ncbi.nlm.nih.gov/entrez/viewer.fcgi?val=GQ221788) | 1701 | USA | 26-Apr | 83 | [CY040890](http://www.ncbi.nlm.nih.gov/entrez/viewer.fcgi?val=CY040890) | 1410 | Mexico | 25-Apr |
|  | [CY039893](http://www.ncbi.nlm.nih.gov/entrez/viewer.fcgi?val=CY039893) | 1737 | USA | 26-Apr | 84 | [CY041132](http://www.ncbi.nlm.nih.gov/entrez/viewer.fcgi?val=CY041132) | 1411 | USA | 25-Apr |
| 136 | [GQ168676](http://www.ncbi.nlm.nih.gov/entrez/viewer.fcgi?val=GQ168676) | 1701 | USA | 26-Apr |  | [CY041591](http://www.ncbi.nlm.nih.gov/entrez/viewer.fcgi?val=CY041591) | 1411 | USA | 28-Apr |
| 137 | [GQ377082](http://www.ncbi.nlm.nih.gov/entrez/viewer.fcgi?val=GQ377082) | 1701 | USA | Apr | 85 | [CY041543](http://www.ncbi.nlm.nih.gov/entrez/viewer.fcgi?val=CY041543) | 1420 | USA | 25-Apr |
|  | [GQ221820](http://www.ncbi.nlm.nih.gov/entrez/viewer.fcgi?val=GQ221820) | 1701 | USA | 26-Apr |  | [CY041060](http://www.ncbi.nlm.nih.gov/entrez/viewer.fcgi?val=CY041060) | 1420 | USA | 26-Apr |
|  | [GQ200273](http://www.ncbi.nlm.nih.gov/entrez/viewer.fcgi?val=GQ200273) | 1701 | USA | 27-Apr |  | [CY041068](http://www.ncbi.nlm.nih.gov/entrez/viewer.fcgi?val=CY041068) | 1420 | USA | 26-Apr |
|  | [GQ221812](http://www.ncbi.nlm.nih.gov/entrez/viewer.fcgi?val=GQ221812) | 1701 | USA | 27-Apr |  | [CY041188](http://www.ncbi.nlm.nih.gov/entrez/viewer.fcgi?val=CY041188) | 1420 | USA | 28-Apr |
|  | [GQ117119](http://www.ncbi.nlm.nih.gov/entrez/viewer.fcgi?val=GQ117119) | 1701 | USA | 27-Apr |  | [CY041492](http://www.ncbi.nlm.nih.gov/entrez/viewer.fcgi?val=CY041492) | 1420 | USA | 28-Apr |
|  | [GQ221826](http://www.ncbi.nlm.nih.gov/entrez/viewer.fcgi?val=GQ221826) | 1701 | USA | 28-Apr |  | [CY041508](http://www.ncbi.nlm.nih.gov/entrez/viewer.fcgi?val=CY041508) | 1420 | USA | 28-Apr |
|  | [GQ231990](http://www.ncbi.nlm.nih.gov/entrez/viewer.fcgi?val=GQ231990) | 1701 | USA | 05-may |  | [CY041180](http://www.ncbi.nlm.nih.gov/entrez/viewer.fcgi?val=CY041180) | 1420 | USA | 01-may |
|  | [GQ221794](http://www.ncbi.nlm.nih.gov/entrez/viewer.fcgi?val=GQ221794) | 1701 | USA | 26-Apr |  | [CY050168](http://www.ncbi.nlm.nih.gov/entrez/viewer.fcgi?val=CY050168) | 1420 | Mexico | 10-may |
| 138 | [CY041050](http://www.ncbi.nlm.nih.gov/entrez/viewer.fcgi?val=CY041050) | 1735 | USA | 26-Apr |  | [CY043301](http://www.ncbi.nlm.nih.gov/entrez/viewer.fcgi?val=CY043301) | 1420 | USA | 12-may |
| 139 | [CY041098](http://www.ncbi.nlm.nih.gov/entrez/viewer.fcgi?val=CY041098) | 1735 | USA | 26-Apr |  | [CY043181](http://www.ncbi.nlm.nih.gov/entrez/viewer.fcgi?val=CY043181) | 1420 | USA | 12-may |
|  | [CY041565](http://www.ncbi.nlm.nih.gov/entrez/viewer.fcgi?val=CY041565) | 1735 | USA | 27-Apr |  | [CY043285](http://www.ncbi.nlm.nih.gov/entrez/viewer.fcgi?val=CY043285) | 1420 | USA | 13-may |
|  | [CY041581](http://www.ncbi.nlm.nih.gov/entrez/viewer.fcgi?val=CY041581) | 1735 | USA | 28-Apr |  | [CY043245](http://www.ncbi.nlm.nih.gov/entrez/viewer.fcgi?val=CY043245) | 1420 | USA | 16-may |
|  | [CY041058](http://www.ncbi.nlm.nih.gov/entrez/viewer.fcgi?val=CY041058) | 1727 | USA | 26-Apr |  | [CY043309](http://www.ncbi.nlm.nih.gov/entrez/viewer.fcgi?val=CY043309) | 1420 | USA | 17-may |
| 140 | [CY041066](http://www.ncbi.nlm.nih.gov/entrez/viewer.fcgi?val=CY041066) | 1727 | USA | 26-Apr |  | [CY043261](http://www.ncbi.nlm.nih.gov/entrez/viewer.fcgi?val=CY043261) | 1420 | USA | 20-may |
|  | [CY041186](http://www.ncbi.nlm.nih.gov/entrez/viewer.fcgi?val=CY041186) | 1727 | USA | 28-Apr |  | [CY053121](http://www.ncbi.nlm.nih.gov/entrez/viewer.fcgi?val=CY053121) | 1420 | USA | 07-jun |
|  | [CY041170](http://www.ncbi.nlm.nih.gov/entrez/viewer.fcgi?val=CY041170) | 1722 | USA | 26-Apr |  | [CY052132](http://www.ncbi.nlm.nih.gov/entrez/viewer.fcgi?val=CY052132) | 1420 | USA | 08-jun |
| 141 | [CY040645](http://www.ncbi.nlm.nih.gov/entrez/viewer.fcgi?val=CY040645) | 1722 | USA | 30-Apr |  | [CY053113](http://www.ncbi.nlm.nih.gov/entrez/viewer.fcgi?val=CY053113) | 1420 | USA | 11-jun |
|  | [GQ231981](http://www.ncbi.nlm.nih.gov/entrez/viewer.fcgi?val=GQ231981) | 1701 | USA | 26-Apr |  | [CY051889](http://www.ncbi.nlm.nih.gov/entrez/viewer.fcgi?val=CY051889) | 1420 | USA | 11-jun |
| 142 | [CY041530](http://www.ncbi.nlm.nih.gov/entrez/viewer.fcgi?val=CY041530) | 1729 | USA | 26-Apr |  | [CY052316](http://www.ncbi.nlm.nih.gov/entrez/viewer.fcgi?val=CY052316) | 1420 | USA | 13-jun |
| 143 | [CY041605](http://www.ncbi.nlm.nih.gov/entrez/viewer.fcgi?val=CY041605) | 1724 | USA | 26-Apr |  | [CY052276](http://www.ncbi.nlm.nih.gov/entrez/viewer.fcgi?val=CY052276) | 1420 | USA | 15-jun |
| 144 | [CY041522](http://www.ncbi.nlm.nih.gov/entrez/viewer.fcgi?val=CY041522) | 1724 | USA | 27-Apr |  | [CY051817](http://www.ncbi.nlm.nih.gov/entrez/viewer.fcgi?val=CY051817) | 1420 | USA | 15-jun |
|  | [CY041726](http://www.ncbi.nlm.nih.gov/entrez/viewer.fcgi?val=CY041726) | 1724 | USA | 29-Apr |  | [CY044122](http://www.ncbi.nlm.nih.gov/entrez/viewer.fcgi?val=CY044122) | 1420 | USA | 17-jun |
|  | [GQ283484](http://www.ncbi.nlm.nih.gov/entrez/viewer.fcgi?val=GQ283484) | 1724 | Italy | 27-may |  | [CY051873](http://www.ncbi.nlm.nih.gov/entrez/viewer.fcgi?val=CY051873) | 1420 | USA | 17-jun |
|  | [GQ265528](http://www.ncbi.nlm.nih.gov/entrez/viewer.fcgi?val=GQ265528) | 1540 | Spain | 26-Apr |  | [CY052100](http://www.ncbi.nlm.nih.gov/entrez/viewer.fcgi?val=CY052100) | 1420 | USA | 17-jun |
| 145 | [GQ265531](http://www.ncbi.nlm.nih.gov/entrez/viewer.fcgi?val=GQ265531) | 1540 | Spain | 27-Apr |  | [CY052417](http://www.ncbi.nlm.nih.gov/entrez/viewer.fcgi?val=CY052417) | 1420 | USA | 19-jun |
|  | [GQ265529](http://www.ncbi.nlm.nih.gov/entrez/viewer.fcgi?val=GQ265529) | 1550 | Spain | 26-Apr |  | [CY051561](http://www.ncbi.nlm.nih.gov/entrez/viewer.fcgi?val=CY051561) | 1420 | USA | 19-jun |
| 146 | [GQ265530](http://www.ncbi.nlm.nih.gov/entrez/viewer.fcgi?val=GQ265530) | 1551 | Spain | 26-Apr |  | [CY051865](http://www.ncbi.nlm.nih.gov/entrez/viewer.fcgi?val=CY051865) | 1420 | USA | 20-jun |
| 147 | [CY041734](http://www.ncbi.nlm.nih.gov/entrez/viewer.fcgi?val=CY041734) | 1728 | USA | 26-Apr |  | [CY052324](http://www.ncbi.nlm.nih.gov/entrez/viewer.fcgi?val=CY052324) | 1420 | USA | 20-jun |
| 148 | [GQ338409](http://www.ncbi.nlm.nih.gov/entrez/viewer.fcgi?val=GQ338409) | 1701 | USA | 26-Apr |  | [CY051537](http://www.ncbi.nlm.nih.gov/entrez/viewer.fcgi?val=CY051537) | 1420 | USA | 21-jun |
| 149 | [GQ396520](http://www.ncbi.nlm.nih.gov/entrez/viewer.fcgi?val=GQ396520) | 947 | Spain | 26-Apr |  | [CY052260](http://www.ncbi.nlm.nih.gov/entrez/viewer.fcgi?val=CY052260) | 1420 | USA | 22-jun |
| 150 | [GQ396586](http://www.ncbi.nlm.nih.gov/entrez/viewer.fcgi?val=GQ396586) | 956 | Spain | 26-Apr |  | [CY051553](http://www.ncbi.nlm.nih.gov/entrez/viewer.fcgi?val=CY051553) | 1420 | USA | 23-jun |
| 151 | [FJ974021](http://www.ncbi.nlm.nih.gov/entrez/viewer.fcgi?val=FJ974021) | 1446 | Germany | 27-Apr |  | [CY051529](http://www.ncbi.nlm.nih.gov/entrez/viewer.fcgi?val=CY051529) | 1420 | USA | 25-jun |
| 152 | [CY039901](http://www.ncbi.nlm.nih.gov/entrez/viewer.fcgi?val=CY039901) | 1735 | USA | 27-Apr |  | [CY052308](http://www.ncbi.nlm.nih.gov/entrez/viewer.fcgi?val=CY052308) | 1420 | USA | 25-jun |
| 153 | [CY039999](http://www.ncbi.nlm.nih.gov/entrez/viewer.fcgi?val=CY039999) | 1736 | USA | 27-Apr |  | [CY052156](http://www.ncbi.nlm.nih.gov/entrez/viewer.fcgi?val=CY052156) | 1420 | USA | 25-jun |
| 154 | [CY052138](http://www.ncbi.nlm.nih.gov/entrez/viewer.fcgi?val=CY052138) | 1736 | USA | 05-jun |  | [CY053168](http://www.ncbi.nlm.nih.gov/entrez/viewer.fcgi?val=CY053168) | 1420 | USA | 26-jun |
|  | [CY040007](http://www.ncbi.nlm.nih.gov/entrez/viewer.fcgi?val=CY040007) | 1724 | USA | 27-Apr |  | [CY052401](http://www.ncbi.nlm.nih.gov/entrez/viewer.fcgi?val=CY052401) | 1420 | USA | 29-jun |
| 155 | [CY040015](http://www.ncbi.nlm.nih.gov/entrez/viewer.fcgi?val=CY040015) | 1734 | USA | 27-Apr |  | [CY051833](http://www.ncbi.nlm.nih.gov/entrez/viewer.fcgi?val=CY051833) | 1420 | USA | 01-jul |
| 156 | [CY040709](http://www.ncbi.nlm.nih.gov/entrez/viewer.fcgi?val=CY040709) | 1734 | USA | 28-Apr |  | [CY051577](http://www.ncbi.nlm.nih.gov/entrez/viewer.fcgi?val=CY051577) | 1420 | USA | 01-jul |
|  | [CY047342](http://www.ncbi.nlm.nih.gov/entrez/viewer.fcgi?val=CY047342) | 1734 | USA | 01-may |  | [CY051585](http://www.ncbi.nlm.nih.gov/entrez/viewer.fcgi?val=CY051585) | 1420 | USA | 09-jul |
|  | [CY043155](http://www.ncbi.nlm.nih.gov/entrez/viewer.fcgi?val=CY043155) | 1734 | USA | 12-may |  | [CY051617](http://www.ncbi.nlm.nih.gov/entrez/viewer.fcgi?val=CY051617) | 1420 | USA | 09-jul |
|  | [CY043179](http://www.ncbi.nlm.nih.gov/entrez/viewer.fcgi?val=CY043179) | 1734 | USA | 12-may |  | [CY051641](http://www.ncbi.nlm.nih.gov/entrez/viewer.fcgi?val=CY051641) | 1420 | USA | 16-jul |
|  | [CY043251](http://www.ncbi.nlm.nih.gov/entrez/viewer.fcgi?val=CY043251) | 1734 | USA | 16-may |  | [CY051825](http://www.ncbi.nlm.nih.gov/entrez/viewer.fcgi?val=CY051825) | 1420 | USA | 24-jul |
|  | [CY043291](http://www.ncbi.nlm.nih.gov/entrez/viewer.fcgi?val=CY043291) | 1734 | USA | 16-may |  | [CY052369](http://www.ncbi.nlm.nih.gov/entrez/viewer.fcgi?val=CY052369) | 1420 | USA | 27-jul |
|  | [CY043307](http://www.ncbi.nlm.nih.gov/entrez/viewer.fcgi?val=CY043307) | 1734 | USA | 17-may |  | [CY052284](http://www.ncbi.nlm.nih.gov/entrez/viewer.fcgi?val=CY052284) | 1420 | USA | 29-jul |
|  | [CY044088](http://www.ncbi.nlm.nih.gov/entrez/viewer.fcgi?val=CY044088) | 1734 | USA | 18-may |  | [CY051681](http://www.ncbi.nlm.nih.gov/entrez/viewer.fcgi?val=CY051681) | 1420 | USA | 30-jul |
|  | [CY044909](http://www.ncbi.nlm.nih.gov/entrez/viewer.fcgi?val=CY044909) | 1734 | USA | 18-may |  | [CY051689](http://www.ncbi.nlm.nih.gov/entrez/viewer.fcgi?val=CY051689) | 1420 | USA | 6-Aug |
|  | [CY043139](http://www.ncbi.nlm.nih.gov/entrez/viewer.fcgi?val=CY043139) | 1734 | USA | 19-may |  | [CY052857](http://www.ncbi.nlm.nih.gov/entrez/viewer.fcgi?val=CY052857) | 1420 | USA | 02-sep |
|  | [CY044136](http://www.ncbi.nlm.nih.gov/entrez/viewer.fcgi?val=CY044136) | 1734 | USA | 19-may |  | [CY052601](http://www.ncbi.nlm.nih.gov/entrez/viewer.fcgi?val=CY052601) | 1420 | USA | 03-sep |
|  | [CY044949](http://www.ncbi.nlm.nih.gov/entrez/viewer.fcgi?val=CY044949) | 1734 | USA | 22-may |  | [CY052937](http://www.ncbi.nlm.nih.gov/entrez/viewer.fcgi?val=CY052937) | 1420 | USA | 03-sep |
|  | [CY045093](http://www.ncbi.nlm.nih.gov/entrez/viewer.fcgi?val=CY045093) | 1734 | USA | 22-may |  | [CY052889](http://www.ncbi.nlm.nih.gov/entrez/viewer.fcgi?val=CY052889) | 1420 | USA | 06-sep |
|  | [CY045029](http://www.ncbi.nlm.nih.gov/entrez/viewer.fcgi?val=CY045029) | 1734 | USA | 31-may |  | [CY051809](http://www.ncbi.nlm.nih.gov/entrez/viewer.fcgi?val=CY051809) | 1420 | USA | 10-sep |
|  | [CY045159](http://www.ncbi.nlm.nih.gov/entrez/viewer.fcgi?val=CY045159) | 1734 | USA | 02-jun |  | [CY052545](http://www.ncbi.nlm.nih.gov/entrez/viewer.fcgi?val=CY052545) | 1420 | USA | 13-sep |
|  | [CY045167](http://www.ncbi.nlm.nih.gov/entrez/viewer.fcgi?val=CY045167) | 1734 | USA | 02-jun |  | [CY052833](http://www.ncbi.nlm.nih.gov/entrez/viewer.fcgi?val=CY052833) | 1420 | USA | 13-sep |
|  | [CY045021](http://www.ncbi.nlm.nih.gov/entrez/viewer.fcgi?val=CY045021) | 1734 | USA | 02-jun |  | [CY052841](http://www.ncbi.nlm.nih.gov/entrez/viewer.fcgi?val=CY052841) | 1420 | USA | 13-sep |
|  | [CY052130](http://www.ncbi.nlm.nih.gov/entrez/viewer.fcgi?val=CY052130) | 1734 | USA | 08-jun | 86 | [CY041615](http://www.ncbi.nlm.nih.gov/entrez/viewer.fcgi?val=CY041615) | 1414 | USA | 25-Apr |
|  | [CY052290](http://www.ncbi.nlm.nih.gov/entrez/viewer.fcgi?val=CY052290) | 1734 | USA | 09-jun | 87 | [GQ323460](http://www.ncbi.nlm.nih.gov/entrez/viewer.fcgi?val=GQ323460) | 1410 | USA | 25-Apr |
|  | [CY045215](http://www.ncbi.nlm.nih.gov/entrez/viewer.fcgi?val=CY045215) | 1734 | USA | 10-jun | 88 | [GQ323542](http://www.ncbi.nlm.nih.gov/entrez/viewer.fcgi?val=GQ323542) | 1410 | USA | 25-Apr |
|  | [CY051887](http://www.ncbi.nlm.nih.gov/entrez/viewer.fcgi?val=CY051887) | 1734 | USA | 11-jun | 89 | [GQ323555](http://www.ncbi.nlm.nih.gov/entrez/viewer.fcgi?val=GQ323555) | 1410 | USA | 25-Apr |
|  | [CY052314](http://www.ncbi.nlm.nih.gov/entrez/viewer.fcgi?val=CY052314) | 1734 | USA | 13-jun | 90 | [CY044165](http://www.ncbi.nlm.nih.gov/entrez/viewer.fcgi?val=CY044165) | 1410 | Mexico | 25-Apr |
|  | [CY052391](http://www.ncbi.nlm.nih.gov/entrez/viewer.fcgi?val=CY052391) | 1734 | USA | 14-jun | 91 | [GQ117077](http://www.ncbi.nlm.nih.gov/entrez/viewer.fcgi?val=GQ117077) | 1410 | USA | 26-Apr |
|  | [CY051815](http://www.ncbi.nlm.nih.gov/entrez/viewer.fcgi?val=CY051815) | 1734 | USA | 15-jun |  | [GQ221789](http://www.ncbi.nlm.nih.gov/entrez/viewer.fcgi?val=GQ221789) | 1410 | USA | 26-Apr |
|  | [CY052415](http://www.ncbi.nlm.nih.gov/entrez/viewer.fcgi?val=CY052415) | 1734 | USA | 19-jun |  | [GQ338374](http://www.ncbi.nlm.nih.gov/entrez/viewer.fcgi?val=GQ338374) | 1410 | USA | 29-Apr |
|  | [CY052234](http://www.ncbi.nlm.nih.gov/entrez/viewer.fcgi?val=CY052234) | 1734 | USA | 19-jun | 92 | [GQ117108](http://www.ncbi.nlm.nih.gov/entrez/viewer.fcgi?val=GQ117108) | 1410 | USA | 26-Apr |
|  | [CY045069](http://www.ncbi.nlm.nih.gov/entrez/viewer.fcgi?val=CY045069) | 1734 | USA | 19-jun | 93 | [CY039895](http://www.ncbi.nlm.nih.gov/entrez/viewer.fcgi?val=CY039895) | 1414 | USA | 26-Apr |
|  | [CY052322](http://www.ncbi.nlm.nih.gov/entrez/viewer.fcgi?val=CY052322) | 1734 | USA | 20-jun |  | [CY041752](http://www.ncbi.nlm.nih.gov/entrez/viewer.fcgi?val=CY041752) | 1414 | USA | 06-may |
|  | [CY051863](http://www.ncbi.nlm.nih.gov/entrez/viewer.fcgi?val=CY051863) | 1734 | USA | 20-jun |  | [CY043221](http://www.ncbi.nlm.nih.gov/entrez/viewer.fcgi?val=CY043221) | 1414 | USA | 11-may |
|  | [CY051535](http://www.ncbi.nlm.nih.gov/entrez/viewer.fcgi?val=CY051535) | 1734 | USA | 21-jun | 94 | [GQ160536](http://www.ncbi.nlm.nih.gov/entrez/viewer.fcgi?val=GQ160536) | 1410 | USA | 26-Apr |
|  | [CY052258](http://www.ncbi.nlm.nih.gov/entrez/viewer.fcgi?val=CY052258) | 1734 | USA | 22-jun | 95 | [GQ168888](http://www.ncbi.nlm.nih.gov/entrez/viewer.fcgi?val=GQ168888) | 1235 | Spain | 26-Apr |
|  | [CY052306](http://www.ncbi.nlm.nih.gov/entrez/viewer.fcgi?val=CY052306) | 1734 | USA | 25-jun | 95 | [GQ168892](http://www.ncbi.nlm.nih.gov/entrez/viewer.fcgi?val=GQ168892) | 1235 | Spain | 27-Apr |
|  | [CY051527](http://www.ncbi.nlm.nih.gov/entrez/viewer.fcgi?val=CY051527) | 1734 | USA | 25-jun | 96 | [GQ168889](http://www.ncbi.nlm.nih.gov/entrez/viewer.fcgi?val=GQ168889) | 1232 | Spain | 26-Apr |
|  | [CY053166](http://www.ncbi.nlm.nih.gov/entrez/viewer.fcgi?val=CY053166) | 1734 | USA | 26-jun | 97 | [GQ168890](http://www.ncbi.nlm.nih.gov/entrez/viewer.fcgi?val=GQ168890) | 1235 | Spain | 26-Apr |
|  | [CY051831](http://www.ncbi.nlm.nih.gov/entrez/viewer.fcgi?val=CY051831) | 1734 | USA | 01-jul | 98 | [GQ183608](http://www.ncbi.nlm.nih.gov/entrez/viewer.fcgi?val=GQ183608) | 1242 | Spain | 26-Apr |
|  | [CY051583](http://www.ncbi.nlm.nih.gov/entrez/viewer.fcgi?val=CY051583) | 1734 | USA | 09-jul | 99 | [GQ183611](http://www.ncbi.nlm.nih.gov/entrez/viewer.fcgi?val=GQ183611) | 1233 | Spain | 26-Apr |
|  | [CY052250](http://www.ncbi.nlm.nih.gov/entrez/viewer.fcgi?val=CY052250) | 1734 | USA | 14-jul | 100 | [CY041052](http://www.ncbi.nlm.nih.gov/entrez/viewer.fcgi?val=CY041052) | 1420 | USA | 26-Apr |
|  | [CY051639](http://www.ncbi.nlm.nih.gov/entrez/viewer.fcgi?val=CY051639) | 1734 | USA | 16-jul | 100 | [CY041204](http://www.ncbi.nlm.nih.gov/entrez/viewer.fcgi?val=CY041204) | 1420 | USA | 28-Apr |
|  | [CY052375](http://www.ncbi.nlm.nih.gov/entrez/viewer.fcgi?val=CY052375) | 1734 | USA | 20-jul | 101 | [CY041100](http://www.ncbi.nlm.nih.gov/entrez/viewer.fcgi?val=CY041100) | 1412 | USA | 26-Apr |
|  | [CY052735](http://www.ncbi.nlm.nih.gov/entrez/viewer.fcgi?val=CY052735) | 1734 | USA | 09-sep |  | [CY041524](http://www.ncbi.nlm.nih.gov/entrez/viewer.fcgi?val=CY041524) | 1412 | USA | 27-Apr |
|  | [CY052463](http://www.ncbi.nlm.nih.gov/entrez/viewer.fcgi?val=CY052463) | 1734 | USA | 12-sep |  | [CY041575](http://www.ncbi.nlm.nih.gov/entrez/viewer.fcgi?val=CY041575) | 1412 | USA | 28-Apr |
|  | [CY040023](http://www.ncbi.nlm.nih.gov/entrez/viewer.fcgi?val=CY040023) | 1733 | USA | 27-Apr |  | [CY040631](http://www.ncbi.nlm.nih.gov/entrez/viewer.fcgi?val=CY040631) | 1412 | USA | 28-Apr |
| 157 | [CY043243](http://www.ncbi.nlm.nih.gov/entrez/viewer.fcgi?val=CY043243) | 1733 | USA | 16-may |  | [CY041164](http://www.ncbi.nlm.nih.gov/entrez/viewer.fcgi?val=CY041164) | 1412 | USA | 29-Apr |
|  | [CY044885](http://www.ncbi.nlm.nih.gov/entrez/viewer.fcgi?val=CY044885) | 1733 | USA | 19-may |  | [CY047352](http://www.ncbi.nlm.nih.gov/entrez/viewer.fcgi?val=CY047352) | 1412 | USA | 30-Apr |
|  | [CY043147](http://www.ncbi.nlm.nih.gov/entrez/viewer.fcgi?val=CY043147) | 1733 | USA | 20-may |  | [CY047336](http://www.ncbi.nlm.nih.gov/entrez/viewer.fcgi?val=CY047336) | 1412 | USA | 06-may |
|  | [CY044104](http://www.ncbi.nlm.nih.gov/entrez/viewer.fcgi?val=CY044104) | 1733 | USA | 05-jun |  | [CY043253](http://www.ncbi.nlm.nih.gov/entrez/viewer.fcgi?val=CY043253) | 1412 | USA | 16-may |
|  | [GQ160526](http://www.ncbi.nlm.nih.gov/entrez/viewer.fcgi?val=GQ160526) | 1701 | USA | 27-Apr |  | [CY044879](http://www.ncbi.nlm.nih.gov/entrez/viewer.fcgi?val=CY044879) | 1412 | USA | 21-may |
| 158 | [GQ160550](http://www.ncbi.nlm.nih.gov/entrez/viewer.fcgi?val=GQ160550) | 1701 | USA | 27-Apr |  | [CY046957](http://www.ncbi.nlm.nih.gov/entrez/viewer.fcgi?val=CY046957) | 1412 | USA | 26-may |
| 159 | [CY040042](http://www.ncbi.nlm.nih.gov/entrez/viewer.fcgi?val=CY040042) | 1732 | USA | 27-Apr |  | [CY047376](http://www.ncbi.nlm.nih.gov/entrez/viewer.fcgi?val=CY047376) | 1412 | USA | 01-jun |
| 160 | [GQ168664](http://www.ncbi.nlm.nih.gov/entrez/viewer.fcgi?val=GQ168664) | 1701 | USA | 27-Apr |  | [CY045079](http://www.ncbi.nlm.nih.gov/entrez/viewer.fcgi?val=CY045079) | 1412 | USA | 12-jun |
| 161 | [GQ183612](http://www.ncbi.nlm.nih.gov/entrez/viewer.fcgi?val=GQ183612) | 1549 | Spain | 27-Apr |  | [GQ351292](http://www.ncbi.nlm.nih.gov/entrez/viewer.fcgi?val=GQ351292) | 1412 | Italy | 14-jun |
| 162 | [GQ265534](http://www.ncbi.nlm.nih.gov/entrez/viewer.fcgi?val=GQ265534) | 1549 | Spain | 29-Apr | 102 | [GQ231980](http://www.ncbi.nlm.nih.gov/entrez/viewer.fcgi?val=GQ231980) | 1410 | USA | 26-Apr |
|  | [GQ200237](http://www.ncbi.nlm.nih.gov/entrez/viewer.fcgi?val=GQ200237) | 1701 | USA | 27-Apr | 103 | [CY041607](http://www.ncbi.nlm.nih.gov/entrez/viewer.fcgi?val=CY041607) | 1415 | USA | 26-Apr |
| 163 | [GQ200268](http://www.ncbi.nlm.nih.gov/entrez/viewer.fcgi?val=GQ200268) | 1701 | USA | 27-Apr |  | [CY041567](http://www.ncbi.nlm.nih.gov/entrez/viewer.fcgi?val=CY041567) | 1415 | USA | 27-Apr |
| 164 | [GQ160553](http://www.ncbi.nlm.nih.gov/entrez/viewer.fcgi?val=GQ160553) | 1701 | USA | Apr |  | [CY041768](http://www.ncbi.nlm.nih.gov/entrez/viewer.fcgi?val=CY041768) | 1415 | USA | 08-may |
|  | [GQ160560](http://www.ncbi.nlm.nih.gov/entrez/viewer.fcgi?val=GQ160560) | 1701 | USA | Apr |  | [CY044050](http://www.ncbi.nlm.nih.gov/entrez/viewer.fcgi?val=CY044050) | 1415 | USA | 20-may |
|  | [GQ160541](http://www.ncbi.nlm.nih.gov/entrez/viewer.fcgi?val=GQ160541) | 1701 | USA | 28-Apr | 104 | [GQ465700](http://www.ncbi.nlm.nih.gov/entrez/viewer.fcgi?val=GQ465700) | 1422 | Canada | 26-Apr |
|  | [GQ160582](http://www.ncbi.nlm.nih.gov/entrez/viewer.fcgi?val=GQ160582) | 1701 | USA | 28-Apr |  | [GQ402229](http://www.ncbi.nlm.nih.gov/entrez/viewer.fcgi?val=GQ402229) | 1422 | Mexico | 29-Apr |
|  | [GQ160594](http://www.ncbi.nlm.nih.gov/entrez/viewer.fcgi?val=GQ160594) | 1701 | USA | 28-Apr |  | [GQ402230](http://www.ncbi.nlm.nih.gov/entrez/viewer.fcgi?val=GQ402230) | 1422 | Mexico | 29-Apr |
|  | [GQ168636](http://www.ncbi.nlm.nih.gov/entrez/viewer.fcgi?val=GQ168636) | 1701 | USA | 28-Apr | 105 | [FJ984953](http://www.ncbi.nlm.nih.gov/entrez/viewer.fcgi?val=FJ984953) | 1109 | Germany | 27-Apr |
|  | [GQ200231](http://www.ncbi.nlm.nih.gov/entrez/viewer.fcgi?val=GQ200231) | 1701 | USA | 29-Apr | 106 | [GQ117071](http://www.ncbi.nlm.nih.gov/entrez/viewer.fcgi?val=GQ117071) | 1410 | USA | 27-Apr |
|  | [GQ160565](http://www.ncbi.nlm.nih.gov/entrez/viewer.fcgi?val=GQ160565) | 1701 | USA | 29-Apr |  | [GQ200218](http://www.ncbi.nlm.nih.gov/entrez/viewer.fcgi?val=GQ200218) | 1410 | USA | Apr |
|  | [CY040822](http://www.ncbi.nlm.nih.gov/entrez/viewer.fcgi?val=CY040822) | 1718 | USA | 27-Apr | 107 | [CY039903](http://www.ncbi.nlm.nih.gov/entrez/viewer.fcgi?val=CY039903) | 1412 | USA | 27-Apr |
| 165 | [CY040838](http://www.ncbi.nlm.nih.gov/entrez/viewer.fcgi?val=CY040838) | 1707 | USA | 27-Apr |  | [CY041623](http://www.ncbi.nlm.nih.gov/entrez/viewer.fcgi?val=CY041623) | 1412 | USA | 30-Apr |
| 166 | [GQ221809](http://www.ncbi.nlm.nih.gov/entrez/viewer.fcgi?val=GQ221809) | 1701 | USA | 27-Apr | 108 | [CY040001](http://www.ncbi.nlm.nih.gov/entrez/viewer.fcgi?val=CY040001) | 1422 | USA | 27-Apr |
| 167 | [CY041082](http://www.ncbi.nlm.nih.gov/entrez/viewer.fcgi?val=CY041082) | 1733 | USA | 27-Apr |  | [CY040044](http://www.ncbi.nlm.nih.gov/entrez/viewer.fcgi?val=CY040044) | 1422 | USA | 27-Apr |
| 168 | [GQ232003](http://www.ncbi.nlm.nih.gov/entrez/viewer.fcgi?val=GQ232003) | 1701 | USA | 27-Apr |  | [CY040711](http://www.ncbi.nlm.nih.gov/entrez/viewer.fcgi?val=CY040711) | 1422 | USA | 28-Apr |
| 169 | [CY041514](http://www.ncbi.nlm.nih.gov/entrez/viewer.fcgi?val=CY041514) | 1730 | USA | 27-Apr |  | [CY041559](http://www.ncbi.nlm.nih.gov/entrez/viewer.fcgi?val=CY041559) | 1422 | USA | 28-Apr |
| 170 | [CY041589](http://www.ncbi.nlm.nih.gov/entrez/viewer.fcgi?val=CY041589) | 1730 | USA | 28-Apr |  | [CY041583](http://www.ncbi.nlm.nih.gov/entrez/viewer.fcgi?val=CY041583) | 1422 | USA | 28-Apr |
|  | [GQ338364](http://www.ncbi.nlm.nih.gov/entrez/viewer.fcgi?val=GQ338364) | 1701 | USA | 27-Apr | 109 | [CY040009](http://www.ncbi.nlm.nih.gov/entrez/viewer.fcgi?val=CY040009) | 1425 | USA | 27-Apr |
| 171 | [GQ200217](http://www.ncbi.nlm.nih.gov/entrez/viewer.fcgi?val=GQ200217) | 1701 | USA | Apr | 110 | [CY040017](http://www.ncbi.nlm.nih.gov/entrez/viewer.fcgi?val=CY040017) | 1421 | USA | 27-Apr |
|  | [GQ232014](http://www.ncbi.nlm.nih.gov/entrez/viewer.fcgi?val=GQ232014) | 1701 | USA | 29-Apr | 111 | [CY040025](http://www.ncbi.nlm.nih.gov/entrez/viewer.fcgi?val=CY040025) | 1412 | USA | 27-Apr |
|  | [GQ323576](http://www.ncbi.nlm.nih.gov/entrez/viewer.fcgi?val=GQ323576) | 1701 | USA | 12-may |  | [CY040824](http://www.ncbi.nlm.nih.gov/entrez/viewer.fcgi?val=CY040824) | 1412 | USA | 27-Apr |
|  | [GQ396518](http://www.ncbi.nlm.nih.gov/entrez/viewer.fcgi?val=GQ396518) | 949 | Spain | 27-Apr |  | [CY040703](http://www.ncbi.nlm.nih.gov/entrez/viewer.fcgi?val=CY040703) | 1412 | USA | 28-Apr |
| 172 | [GQ402191](http://www.ncbi.nlm.nih.gov/entrez/viewer.fcgi?val=GQ402191) | 1744 | Mexico | 27-Apr |  | [CY047328](http://www.ncbi.nlm.nih.gov/entrez/viewer.fcgi?val=CY047328) | 1412 | USA | 28-Apr |
| 173 | [GQ475936](http://www.ncbi.nlm.nih.gov/entrez/viewer.fcgi?val=GQ475936) | 1698 | USA | 27-Apr |  | [CY040832](http://www.ncbi.nlm.nih.gov/entrez/viewer.fcgi?val=CY040832) | 1412 | USA | 29-Apr |
| 174 | [GQ894929](http://www.ncbi.nlm.nih.gov/entrez/viewer.fcgi?val=GQ894929) | 1701 | USA | 27-Apr |  | [CY041728](http://www.ncbi.nlm.nih.gov/entrez/viewer.fcgi?val=CY041728) | 1412 | USA | 29-Apr |
| 175 | [CY050476](http://www.ncbi.nlm.nih.gov/entrez/viewer.fcgi?val=CY050476) | 1723 | USA | 27-Apr |  | [CY040695](http://www.ncbi.nlm.nih.gov/entrez/viewer.fcgi?val=CY040695) | 1412 | USA | 30-Apr |
| 176 | [CY050612](http://www.ncbi.nlm.nih.gov/entrez/viewer.fcgi?val=CY050612) | 1706 | USA | 27-Apr |  | [CY045161](http://www.ncbi.nlm.nih.gov/entrez/viewer.fcgi?val=CY045161) | 1412 | USA | 02-jun |
| 177 | [CY050660](http://www.ncbi.nlm.nih.gov/entrez/viewer.fcgi?val=CY050660) | 1725 | USA | 27-Apr | 112 | [GQ160524](http://www.ncbi.nlm.nih.gov/entrez/viewer.fcgi?val=GQ160524) | 1410 | USA | 27-Apr |
| 178 | [GQ160531](http://www.ncbi.nlm.nih.gov/entrez/viewer.fcgi?val=GQ160531) | 1701 | USA | 28-Apr |  | [GQ323461](http://www.ncbi.nlm.nih.gov/entrez/viewer.fcgi?val=GQ323461) | 1410 | USA | 29-Apr |
| 179 | [GQ160586](http://www.ncbi.nlm.nih.gov/entrez/viewer.fcgi?val=GQ160586) | 1701 | USA | 28-Apr | 113 | [GQ168891](http://www.ncbi.nlm.nih.gov/entrez/viewer.fcgi?val=GQ168891) | 1234 | Spain | 27-Apr |
| 180 | [GQ166661](http://www.ncbi.nlm.nih.gov/entrez/viewer.fcgi?val=GQ166661) | 1701 | United Kingdom | 28-Apr |  | [GQ168894](http://www.ncbi.nlm.nih.gov/entrez/viewer.fcgi?val=GQ168894) | 1234 | Spain | 27-Apr |
| 181 | [GQ338400](http://www.ncbi.nlm.nih.gov/entrez/viewer.fcgi?val=GQ338400) | 1701 | USA | 28-Apr | 114 | [GQ168893](http://www.ncbi.nlm.nih.gov/entrez/viewer.fcgi?val=GQ168893) | 1236 | Spain | 27-Apr |
|  | [GQ338339](http://www.ncbi.nlm.nih.gov/entrez/viewer.fcgi?val=GQ338339) | 1701 | USA | 29-Apr |  | [GQ166190](http://www.ncbi.nlm.nih.gov/entrez/viewer.fcgi?val=GQ166190) | 1236 | Spain | 01-may |
|  | [GQ338416](http://www.ncbi.nlm.nih.gov/entrez/viewer.fcgi?val=GQ338416) | 1701 | USA | 29-Apr | 115 | [GQ168895](http://www.ncbi.nlm.nih.gov/entrez/viewer.fcgi?val=GQ168895) | 1235 | Spain | 27-Apr |
|  | [GQ232033](http://www.ncbi.nlm.nih.gov/entrez/viewer.fcgi?val=GQ232033) | 1701 | USA | 30-Apr | 116 | [GQ183607](http://www.ncbi.nlm.nih.gov/entrez/viewer.fcgi?val=GQ183607) | 1235 | Spain | 27-Apr |
|  | [GQ323495](http://www.ncbi.nlm.nih.gov/entrez/viewer.fcgi?val=GQ323495) | 1701 | USA | 07-may | 117 | [CY040840](http://www.ncbi.nlm.nih.gov/entrez/viewer.fcgi?val=CY040840) | 1413 | USA | 27-Apr |
|  | [GQ323530](http://www.ncbi.nlm.nih.gov/entrez/viewer.fcgi?val=GQ323530) | 1701 | USA | 23-may |  | [CY046701](http://www.ncbi.nlm.nih.gov/entrez/viewer.fcgi?val=CY046701) | 1413 | USA | 24-may |
|  | [CY046819](http://www.ncbi.nlm.nih.gov/entrez/viewer.fcgi?val=CY046819) | 1701 | USA | 30-may | 118 | [CY041084](http://www.ncbi.nlm.nih.gov/entrez/viewer.fcgi?val=CY041084) | 1412 | USA | 27-Apr |
|  | [GQ455032](http://www.ncbi.nlm.nih.gov/entrez/viewer.fcgi?val=GQ455032) | 1701 | China | 17-jun | 119 | [CY041516](http://www.ncbi.nlm.nih.gov/entrez/viewer.fcgi?val=CY041516) | 1421 | USA | 27-Apr |
|  | [CY044155](http://www.ncbi.nlm.nih.gov/entrez/viewer.fcgi?val=CY044155) | 1701 | Nicaragua | 26-jun |  | [CY046965](http://www.ncbi.nlm.nih.gov/entrez/viewer.fcgi?val=CY046965) | 1421 | USA | 21-may |
|  | [CY040629](http://www.ncbi.nlm.nih.gov/entrez/viewer.fcgi?val=CY040629) | 1719 | USA | 28-Apr |  | [CY044863](http://www.ncbi.nlm.nih.gov/entrez/viewer.fcgi?val=CY044863) | 1421 | USA | 31-may |
| 182 | [CY043267](http://www.ncbi.nlm.nih.gov/entrez/viewer.fcgi?val=CY043267) | 1719 | USA | 19-may |  | [CY052292](http://www.ncbi.nlm.nih.gov/entrez/viewer.fcgi?val=CY052292) | 1421 | USA | 09-jun |
|  | [CY040701](http://www.ncbi.nlm.nih.gov/entrez/viewer.fcgi?val=CY040701) | 1725 | USA | 28-Apr |  | [CY052252](http://www.ncbi.nlm.nih.gov/entrez/viewer.fcgi?val=CY052252) | 1421 | USA | 14-jul |
| 183 | [CY040717](http://www.ncbi.nlm.nih.gov/entrez/viewer.fcgi?val=CY040717) | 1716 | USA | 28-Apr | 120 | [GQ323480](http://www.ncbi.nlm.nih.gov/entrez/viewer.fcgi?val=GQ323480) | 1410 | USA | 27-Apr |
| 184 | [GQ221823](http://www.ncbi.nlm.nih.gov/entrez/viewer.fcgi?val=GQ221823) | 1701 | USA | 28-Apr |  | [GQ323549](http://www.ncbi.nlm.nih.gov/entrez/viewer.fcgi?val=GQ323549) | 1410 | USA | 04-may |
| 185 | [CY041090](http://www.ncbi.nlm.nih.gov/entrez/viewer.fcgi?val=CY041090) | 1732 | USA | 28-Apr | 121 | [GQ338393](http://www.ncbi.nlm.nih.gov/entrez/viewer.fcgi?val=GQ338393) | 1410 | USA | 27-Apr |
| 186 | [CY047318](http://www.ncbi.nlm.nih.gov/entrez/viewer.fcgi?val=CY047318) | 1732 | USA | 28-Apr |  | [GQ323543](http://www.ncbi.nlm.nih.gov/entrez/viewer.fcgi?val=GQ323543) | 1410 | USA | 28-Apr |
|  | [CY043211](http://www.ncbi.nlm.nih.gov/entrez/viewer.fcgi?val=CY043211) | 1732 | USA | 13-may |  | [GQ338371](http://www.ncbi.nlm.nih.gov/entrez/viewer.fcgi?val=GQ338371) | 1410 | USA | 28-Apr |
|  | [CY043227](http://www.ncbi.nlm.nih.gov/entrez/viewer.fcgi?val=CY043227) | 1732 | USA | 17-may |  | [GQ377086](http://www.ncbi.nlm.nih.gov/entrez/viewer.fcgi?val=GQ377086) | 1410 | USA | 29-Apr |
|  | [CY041106](http://www.ncbi.nlm.nih.gov/entrez/viewer.fcgi?val=CY041106) | 1731 | USA | 28-Apr |  | [GQ377074](http://www.ncbi.nlm.nih.gov/entrez/viewer.fcgi?val=GQ377074) | 1410 | USA | 30-Apr |
| 187 | [CY041114](http://www.ncbi.nlm.nih.gov/entrez/viewer.fcgi?val=CY041114) | 1721 | USA | 28-Apr |  | [GQ221696](http://www.ncbi.nlm.nih.gov/entrez/viewer.fcgi?val=GQ221696) | 1410 | China | 17-may |
| 188 | [CY044893](http://www.ncbi.nlm.nih.gov/entrez/viewer.fcgi?val=CY044893) | 1721 | USA | 20-may | 122 | [GQ338413](http://www.ncbi.nlm.nih.gov/entrez/viewer.fcgi?val=GQ338413) | 1410 | USA | 27-Apr |
|  | [CY045207](http://www.ncbi.nlm.nih.gov/entrez/viewer.fcgi?val=CY045207) | 1721 | USA | 08-jun | 123 | [GQ338415](http://www.ncbi.nlm.nih.gov/entrez/viewer.fcgi?val=GQ338415) | 1410 | USA | 27-Apr |
|  | [CY041146](http://www.ncbi.nlm.nih.gov/entrez/viewer.fcgi?val=CY041146) | 1731 | USA | 28-Apr |  | [GQ377036](http://www.ncbi.nlm.nih.gov/entrez/viewer.fcgi?val=GQ377036) | 1410 | USA | 30-Apr |
| 189 | [CY041154](http://www.ncbi.nlm.nih.gov/entrez/viewer.fcgi?val=CY041154) | 1738 | USA | 28-Apr |  | [GQ377033](http://www.ncbi.nlm.nih.gov/entrez/viewer.fcgi?val=GQ377033) | 1410 | USA | May |
| 190 | [CY041202](http://www.ncbi.nlm.nih.gov/entrez/viewer.fcgi?val=CY041202) | 1736 | USA | 28-Apr |  | [CY046253](http://www.ncbi.nlm.nih.gov/entrez/viewer.fcgi?val=CY046253) | 1410 | USA | 04-may |
| 191 | [GQ232037](http://www.ncbi.nlm.nih.gov/entrez/viewer.fcgi?val=GQ232037) | 1701 | USA | 28-Apr |  | [GQ323572](http://www.ncbi.nlm.nih.gov/entrez/viewer.fcgi?val=GQ323572) | 1410 | USA | 04-may |
| 192 | [GQ232044](http://www.ncbi.nlm.nih.gov/entrez/viewer.fcgi?val=GQ232044) | 1701 | USA | 28-Apr |  | [GQ323548](http://www.ncbi.nlm.nih.gov/entrez/viewer.fcgi?val=GQ323548) | 1410 | USA | 04-may |
| 193 | [GQ232057](http://www.ncbi.nlm.nih.gov/entrez/viewer.fcgi?val=GQ232057) | 1701 | USA | 28-Apr |  | [GQ338362](http://www.ncbi.nlm.nih.gov/entrez/viewer.fcgi?val=GQ338362) | 1410 | USA | 07-may |
| 194 | [CY041498](http://www.ncbi.nlm.nih.gov/entrez/viewer.fcgi?val=CY041498) | 1733 | USA | 28-Apr |  | [CY046565](http://www.ncbi.nlm.nih.gov/entrez/viewer.fcgi?val=CY046565) | 1410 | USA | 09-may |
| 195 | [CY041506](http://www.ncbi.nlm.nih.gov/entrez/viewer.fcgi?val=CY041506) | 1729 | USA | 28-Apr |  | [GQ290107](http://www.ncbi.nlm.nih.gov/entrez/viewer.fcgi?val=GQ290107) | 1410 | China | 20-may |
| 196 | [CY041194](http://www.ncbi.nlm.nih.gov/entrez/viewer.fcgi?val=CY041194) | 1729 | USA | 29-Apr |  | [GQ225383](http://www.ncbi.nlm.nih.gov/entrez/viewer.fcgi?val=GQ225383) | 1410 | China | 20-may |
|  | [CY041178](http://www.ncbi.nlm.nih.gov/entrez/viewer.fcgi?val=CY041178) | 1729 | USA | 01-may |  | [GQ223415](http://www.ncbi.nlm.nih.gov/entrez/viewer.fcgi?val=GQ223415) | 1410 | China | 21-may |
|  | [GQ265533](http://www.ncbi.nlm.nih.gov/entrez/viewer.fcgi?val=GQ265533) | 1551 | Spain | 28-Apr |  | [CY046781](http://www.ncbi.nlm.nih.gov/entrez/viewer.fcgi?val=CY046781) | 1410 | USA | 26-may |
| 197 | [CY041830](http://www.ncbi.nlm.nih.gov/entrez/viewer.fcgi?val=CY041830) | 1732 | USA | 28-Apr |  | [GQ367490](http://www.ncbi.nlm.nih.gov/entrez/viewer.fcgi?val=GQ367490) | 1410 | Brazil | 01-jun |
| 198 | [GQ338355](http://www.ncbi.nlm.nih.gov/entrez/viewer.fcgi?val=GQ338355) | 1701 | USA | 28-Apr |  | [GQ334357](http://www.ncbi.nlm.nih.gov/entrez/viewer.fcgi?val=GQ334357) | 1410 | Japan | 16-jun |
| 199 | [GQ338394](http://www.ncbi.nlm.nih.gov/entrez/viewer.fcgi?val=GQ338394) | 1701 | USA | 28-Apr |  | [GQ365457](http://www.ncbi.nlm.nih.gov/entrez/viewer.fcgi?val=GQ365457) | 1410 | Japan | 16-jun |
| 200 | [GQ396535](http://www.ncbi.nlm.nih.gov/entrez/viewer.fcgi?val=GQ396535) | 949 | Spain | 28-Apr | 124 | [GQ377059](http://www.ncbi.nlm.nih.gov/entrez/viewer.fcgi?val=GQ377059) | 1410 | USA | 27-Apr |
| 201 | [GQ396570](http://www.ncbi.nlm.nih.gov/entrez/viewer.fcgi?val=GQ396570) | 949 | Spain | 28-Apr | 125 | [GQ402231](http://www.ncbi.nlm.nih.gov/entrez/viewer.fcgi?val=GQ402231) | 1422 | Mexico | 27-Apr |
|  | [GQ396574](http://www.ncbi.nlm.nih.gov/entrez/viewer.fcgi?val=GQ396574) | 949 | Spain | 28-Apr | 126 | [GQ465701](http://www.ncbi.nlm.nih.gov/entrez/viewer.fcgi?val=GQ465701) | 1422 | Canada | 27-Apr |
|  | [GQ402192](http://www.ncbi.nlm.nih.gov/entrez/viewer.fcgi?val=GQ402192) | 1743 | Mexico | 28-Apr |  | [GQ465697](http://www.ncbi.nlm.nih.gov/entrez/viewer.fcgi?val=GQ465697) | 1422 | Canada | 28-Apr |
| 202 | [GQ402193](http://www.ncbi.nlm.nih.gov/entrez/viewer.fcgi?val=GQ402193) | 1743 | Mexico | 29-Apr |  | [GQ402232](http://www.ncbi.nlm.nih.gov/entrez/viewer.fcgi?val=GQ402232) | 1422 | Mexico | 28-Apr |
|  | [GQ465680](http://www.ncbi.nlm.nih.gov/entrez/viewer.fcgi?val=GQ465680) | 1743 | Canada | 08-may |  | [GQ402233](http://www.ncbi.nlm.nih.gov/entrez/viewer.fcgi?val=GQ402233) | 1422 | Mexico | 29-Apr |
|  | [GQ402199](http://www.ncbi.nlm.nih.gov/entrez/viewer.fcgi?val=GQ402199) | 1743 | Canada | 17-may |  | [GQ402240](http://www.ncbi.nlm.nih.gov/entrez/viewer.fcgi?val=GQ402240) | 1422 | Canada | 20-may |
|  | [GQ465673](http://www.ncbi.nlm.nih.gov/entrez/viewer.fcgi?val=GQ465673) | 1743 | Canada | 28-Apr |  | [GQ402243](http://www.ncbi.nlm.nih.gov/entrez/viewer.fcgi?val=GQ402243) | 1422 | Canada | 21-may |
| 203 | [CY046331](http://www.ncbi.nlm.nih.gov/entrez/viewer.fcgi?val=CY046331) | 1732 | USA | 28-Apr |  | [GQ402244](http://www.ncbi.nlm.nih.gov/entrez/viewer.fcgi?val=GQ402244) | 1422 | Canada | 28-may |
| 204 | [CY046379](http://www.ncbi.nlm.nih.gov/entrez/viewer.fcgi?val=CY046379) | 1732 | USA | 02-may |  | [GQ402246](http://www.ncbi.nlm.nih.gov/entrez/viewer.fcgi?val=GQ402246) | 1422 | Canada | 30-may |
|  | [CY046883](http://www.ncbi.nlm.nih.gov/entrez/viewer.fcgi?val=CY046883) | 1732 | USA | 03-may |  | [GU290049](http://www.ncbi.nlm.nih.gov/entrez/viewer.fcgi?val=GU290049) | 1422 | Czech Republic | 27-jun |
|  | [CY046211](http://www.ncbi.nlm.nih.gov/entrez/viewer.fcgi?val=CY046211) | 1732 | USA | 03-may | 127 | [CY050478](http://www.ncbi.nlm.nih.gov/entrez/viewer.fcgi?val=CY050478) | 1423 | USA | 27-Apr |
|  | [CY046915](http://www.ncbi.nlm.nih.gov/entrez/viewer.fcgi?val=CY046915) | 1732 | USA | 04-may | 128 | [CY050614](http://www.ncbi.nlm.nih.gov/entrez/viewer.fcgi?val=CY050614) | 1404 | USA | 27-Apr |
|  | [CY047326](http://www.ncbi.nlm.nih.gov/entrez/viewer.fcgi?val=CY047326) | 1727 | USA | 28-Apr | 129 | [CY050662](http://www.ncbi.nlm.nih.gov/entrez/viewer.fcgi?val=CY050662) | 1422 | USA | 27-Apr |
| 205 | [CY050067](http://www.ncbi.nlm.nih.gov/entrez/viewer.fcgi?val=CY050067) | 1698 | USA | 28-Apr | 130 | [GQ160529](http://www.ncbi.nlm.nih.gov/entrez/viewer.fcgi?val=GQ160529) | 1410 | USA | 28-Apr |
| 206 | [CY053071](http://www.ncbi.nlm.nih.gov/entrez/viewer.fcgi?val=CY053071) | 1734 | USA | 28-Apr |  | [GQ225375](http://www.ncbi.nlm.nih.gov/entrez/viewer.fcgi?val=GQ225375) | 1410 | China | 15-may |
| 207 | [FJ982430](http://www.ncbi.nlm.nih.gov/entrez/viewer.fcgi?val=FJ982430) | 1699 | Denmark | 29-Apr |  | [GQ232095](http://www.ncbi.nlm.nih.gov/entrez/viewer.fcgi?val=GQ232095) | 1410 | China | 23-may |
| 208 | [GQ150335](http://www.ncbi.nlm.nih.gov/entrez/viewer.fcgi?val=GQ150335) | 1688 | New Zealand | 29-Apr |  | [AB514229](http://www.ncbi.nlm.nih.gov/entrez/viewer.fcgi?val=AB514229) | 1410 | Japan | 03-jun |
| 209 | [CY040031](http://www.ncbi.nlm.nih.gov/entrez/viewer.fcgi?val=CY040031) | 1736 | USA | 29-Apr |  | [GU014795](http://www.ncbi.nlm.nih.gov/entrez/viewer.fcgi?val=GU014795) | 1410 | Japan | 18-jun |
| 210 | [GQ200270](http://www.ncbi.nlm.nih.gov/entrez/viewer.fcgi?val=GQ200270) | 1701 | USA | 29-Apr | 131 | [GQ160585](http://www.ncbi.nlm.nih.gov/entrez/viewer.fcgi?val=GQ160585) | 1410 | USA | 28-Apr |
| 211 | [GQ214151](http://www.ncbi.nlm.nih.gov/entrez/viewer.fcgi?val=GQ214151) | 1758 | France | 29-Apr | 132 | [GQ168896](http://www.ncbi.nlm.nih.gov/entrez/viewer.fcgi?val=GQ168896) | 1234 | Spain | 28-Apr |
| 212 | [CY040830](http://www.ncbi.nlm.nih.gov/entrez/viewer.fcgi?val=CY040830) | 1724 | USA | 29-Apr |  | [GQ168898](http://www.ncbi.nlm.nih.gov/entrez/viewer.fcgi?val=GQ168898) | 1234 | Spain | 29-Apr |
| 213 | [CY044901](http://www.ncbi.nlm.nih.gov/entrez/viewer.fcgi?val=CY044901) | 1724 | USA | 21-may | 133 | [GQ168897](http://www.ncbi.nlm.nih.gov/entrez/viewer.fcgi?val=GQ168897) | 1237 | Spain | 28-Apr |
|  | [CY040854](http://www.ncbi.nlm.nih.gov/entrez/viewer.fcgi?val=CY040854) | 1719 | USA | 29-Apr | 134 | [CY040719](http://www.ncbi.nlm.nih.gov/entrez/viewer.fcgi?val=CY040719) | 1412 | USA | 28-Apr |
| 214 | [CY040862](http://www.ncbi.nlm.nih.gov/entrez/viewer.fcgi?val=CY040862) | 1710 | USA | 29-Apr | 135 | [GQ221827](http://www.ncbi.nlm.nih.gov/entrez/viewer.fcgi?val=GQ221827) | 1410 | USA | 28-Apr |
| 215 | [CY041162](http://www.ncbi.nlm.nih.gov/entrez/viewer.fcgi?val=CY041162) | 1731 | USA | 29-Apr | 136 | [CY041108](http://www.ncbi.nlm.nih.gov/entrez/viewer.fcgi?val=CY041108) | 1412 | USA | 28-Apr |
| 216 | [GQ351290](http://www.ncbi.nlm.nih.gov/entrez/viewer.fcgi?val=GQ351290) | 1731 | Italy | 14-jun | 137 | [CY041116](http://www.ncbi.nlm.nih.gov/entrez/viewer.fcgi?val=CY041116) | 1413 | USA | 28-Apr |
|  | [CY041482](http://www.ncbi.nlm.nih.gov/entrez/viewer.fcgi?val=CY041482) | 1706 | USA | 29-Apr |  | [CY041832](http://www.ncbi.nlm.nih.gov/entrez/viewer.fcgi?val=CY041832) | 1413 | USA | 28-Apr |
| 217 | [GQ265535](http://www.ncbi.nlm.nih.gov/entrez/viewer.fcgi?val=GQ265535) | 1547 | Spain | 29-Apr |  | [CY043125](http://www.ncbi.nlm.nih.gov/entrez/viewer.fcgi?val=CY043125) | 1413 | USA | 08-may |
| 218 | [GQ265536](http://www.ncbi.nlm.nih.gov/entrez/viewer.fcgi?val=GQ265536) | 1547 | Spain | 29-Apr |  | [CY043133](http://www.ncbi.nlm.nih.gov/entrez/viewer.fcgi?val=CY043133) | 1413 | USA | 14-may |
| 219 | [GQ338375](http://www.ncbi.nlm.nih.gov/entrez/viewer.fcgi?val=GQ338375) | 1701 | USA | 29-Apr |  | [CY041792](http://www.ncbi.nlm.nih.gov/entrez/viewer.fcgi?val=CY041792) | 1413 | USA | 14-may |
| 220 | [GQ365658](http://www.ncbi.nlm.nih.gov/entrez/viewer.fcgi?val=GQ365658) | 1704 | Germany | 29-Apr |  | [CY043141](http://www.ncbi.nlm.nih.gov/entrez/viewer.fcgi?val=CY043141) | 1413 | USA | 19-may |
| 221 | [GQ396542](http://www.ncbi.nlm.nih.gov/entrez/viewer.fcgi?val=GQ396542) | 948 | Spain | 29-Apr |  | [CY044138](http://www.ncbi.nlm.nih.gov/entrez/viewer.fcgi?val=CY044138) | 1413 | USA | 19-may |
| 222 | [GQ396590](http://www.ncbi.nlm.nih.gov/entrez/viewer.fcgi?val=GQ396590) | 948 | Spain | 29-Apr |  | [CY044935](http://www.ncbi.nlm.nih.gov/entrez/viewer.fcgi?val=CY044935) | 1413 | USA | 20-may |
| 223 | [GQ402189](http://www.ncbi.nlm.nih.gov/entrez/viewer.fcgi?val=GQ402189) | 1743 | Mexico | 29-Apr | 138 | [CY041148](http://www.ncbi.nlm.nih.gov/entrez/viewer.fcgi?val=CY041148) | 1412 | USA | 28-Apr |
| 224 | [CY046299](http://www.ncbi.nlm.nih.gov/entrez/viewer.fcgi?val=CY046299) | 1734 | USA | 29-Apr |  | [CY046365](http://www.ncbi.nlm.nih.gov/entrez/viewer.fcgi?val=CY046365) | 1412 | USA | 04-may |
| 225 | [CY046411](http://www.ncbi.nlm.nih.gov/entrez/viewer.fcgi?val=CY046411) | 1734 | USA | 30-Apr |  | [CY051401](http://www.ncbi.nlm.nih.gov/entrez/viewer.fcgi?val=CY051401) | 1412 | USA | 10-jun |
|  | [CY046419](http://www.ncbi.nlm.nih.gov/entrez/viewer.fcgi?val=CY046419) | 1734 | USA | 30-Apr |  | [CY051113](http://www.ncbi.nlm.nih.gov/entrez/viewer.fcgi?val=CY051113) | 1412 | USA | 12-jun |
|  | [CY046355](http://www.ncbi.nlm.nih.gov/entrez/viewer.fcgi?val=CY046355) | 1734 | USA | 30-Apr | 139 | [CY041156](http://www.ncbi.nlm.nih.gov/entrez/viewer.fcgi?val=CY041156) | 1420 | USA | 28-Apr |
|  | [CY046395](http://www.ncbi.nlm.nih.gov/entrez/viewer.fcgi?val=CY046395) | 1734 | USA | 02-may |  | [CY053081](http://www.ncbi.nlm.nih.gov/entrez/viewer.fcgi?val=CY053081) | 1420 | USA | 01-may |
|  | [CY046339](http://www.ncbi.nlm.nih.gov/entrez/viewer.fcgi?val=CY046339) | 1734 | USA | 02-may |  | [CY053089](http://www.ncbi.nlm.nih.gov/entrez/viewer.fcgi?val=CY053089) | 1420 | USA | 01-may |
|  | [CY046347](http://www.ncbi.nlm.nih.gov/entrez/viewer.fcgi?val=CY046347) | 1734 | USA | 03-may |  | [CY050374](http://www.ncbi.nlm.nih.gov/entrez/viewer.fcgi?val=CY050374) | 1420 | USA | 13-may |
|  | [CY046227](http://www.ncbi.nlm.nih.gov/entrez/viewer.fcgi?val=CY046227) | 1734 | USA | 03-may |  | [CY046773](http://www.ncbi.nlm.nih.gov/entrez/viewer.fcgi?val=CY046773) | 1420 | USA | 19-may |
|  | [CY046219](http://www.ncbi.nlm.nih.gov/entrez/viewer.fcgi?val=CY046219) | 1734 | USA | 04-may |  | [CY053240](http://www.ncbi.nlm.nih.gov/entrez/viewer.fcgi?val=CY053240) | 1420 | USA | 27-may |
|  | [CY046531](http://www.ncbi.nlm.nih.gov/entrez/viewer.fcgi?val=CY046531) | 1734 | USA | 04-may |  | [CY053248](http://www.ncbi.nlm.nih.gov/entrez/viewer.fcgi?val=CY053248) | 1420 | USA | 27-may |
|  | [CY046867](http://www.ncbi.nlm.nih.gov/entrez/viewer.fcgi?val=CY046867) | 1734 | USA | 04-may |  | [CY045007](http://www.ncbi.nlm.nih.gov/entrez/viewer.fcgi?val=CY045007) | 1420 | USA | 28-may |
|  | [CY050206](http://www.ncbi.nlm.nih.gov/entrez/viewer.fcgi?val=CY050206) | 1734 | Mexico | 08-may |  | [CY050953](http://www.ncbi.nlm.nih.gov/entrez/viewer.fcgi?val=CY050953) | 1420 | USA | 01-jun |
|  | [CY050238](http://www.ncbi.nlm.nih.gov/entrez/viewer.fcgi?val=CY050238) | 1734 | Mexico | 09-may |  | [CY052124](http://www.ncbi.nlm.nih.gov/entrez/viewer.fcgi?val=CY052124) | 1420 | USA | 09-jun |
|  | [CY050847](http://www.ncbi.nlm.nih.gov/entrez/viewer.fcgi?val=CY050847) | 1734 | Mexico | 09-may | 140 | [CY041500](http://www.ncbi.nlm.nih.gov/entrez/viewer.fcgi?val=CY041500) | 1422 | USA | 28-Apr |
|  | [CY050863](http://www.ncbi.nlm.nih.gov/entrez/viewer.fcgi?val=CY050863) | 1734 | Mexico | 09-may | 141 | [GQ338333](http://www.ncbi.nlm.nih.gov/entrez/viewer.fcgi?val=GQ338333) | 1410 | USA | 28-Apr |
|  | [CY046547](http://www.ncbi.nlm.nih.gov/entrez/viewer.fcgi?val=CY046547) | 1734 | USA | 10-may | 141 | [GQ323547](http://www.ncbi.nlm.nih.gov/entrez/viewer.fcgi?val=GQ323547) | 1410 | USA | 30-Apr |
|  | [CY046651](http://www.ncbi.nlm.nih.gov/entrez/viewer.fcgi?val=CY046651) | 1734 | USA | 14-may | 142 | [GQ338401](http://www.ncbi.nlm.nih.gov/entrez/viewer.fcgi?val=GQ338401) | 1410 | USA | 28-Apr |
|  | [CY046659](http://www.ncbi.nlm.nih.gov/entrez/viewer.fcgi?val=CY046659) | 1734 | USA | 14-may |  | [GQ323493](http://www.ncbi.nlm.nih.gov/entrez/viewer.fcgi?val=GQ323493) | 1410 | USA | 07-may |
|  | [CY046667](http://www.ncbi.nlm.nih.gov/entrez/viewer.fcgi?val=CY046667) | 1734 | USA | 14-may |  | [GQ323529](http://www.ncbi.nlm.nih.gov/entrez/viewer.fcgi?val=GQ323529) | 1410 | USA | 23-may |
|  | [CY046619](http://www.ncbi.nlm.nih.gov/entrez/viewer.fcgi?val=CY046619) | 1734 | USA | 16-may |  | [GQ455034](http://www.ncbi.nlm.nih.gov/entrez/viewer.fcgi?val=GQ455034) | 1410 | China | 17-jun |
|  | [CY046739](http://www.ncbi.nlm.nih.gov/entrez/viewer.fcgi?val=CY046739) | 1734 | USA | 19-may | 143 | [CY046333](http://www.ncbi.nlm.nih.gov/entrez/viewer.fcgi?val=CY046333) | 1416 | USA | 28-Apr |
|  | [CY046755](http://www.ncbi.nlm.nih.gov/entrez/viewer.fcgi?val=CY046755) | 1734 | USA | 19-may | 144 | [CY047320](http://www.ncbi.nlm.nih.gov/entrez/viewer.fcgi?val=CY047320) | 1414 | USA | 28-Apr |
|  | [CY053230](http://www.ncbi.nlm.nih.gov/entrez/viewer.fcgi?val=CY053230) | 1734 | USA | 23-may |  | [CY044943](http://www.ncbi.nlm.nih.gov/entrez/viewer.fcgi?val=CY044943) | 1414 | USA | 26-may |
|  | [CY053254](http://www.ncbi.nlm.nih.gov/entrez/viewer.fcgi?val=CY053254) | 1734 | USA | 27-may | 145 | [CY053073](http://www.ncbi.nlm.nih.gov/entrez/viewer.fcgi?val=CY053073) | 1420 | USA | 28-Apr |
|  | [CY053309](http://www.ncbi.nlm.nih.gov/entrez/viewer.fcgi?val=CY053309) | 1734 | USA | 28-may | 146 | [FJ982431](http://www.ncbi.nlm.nih.gov/entrez/viewer.fcgi?val=FJ982431) | 1401 | Denmark | 29-Apr |
|  | [CY046795](http://www.ncbi.nlm.nih.gov/entrez/viewer.fcgi?val=CY046795) | 1734 | USA | 28-may | 147 | [GQ122098](http://www.ncbi.nlm.nih.gov/entrez/viewer.fcgi?val=GQ122098) | 1371 | New Zealand | 29-Apr |
|  | [CY051279](http://www.ncbi.nlm.nih.gov/entrez/viewer.fcgi?val=CY051279) | 1734 | USA | 02-jun | 148 | [GQ150333](http://www.ncbi.nlm.nih.gov/entrez/viewer.fcgi?val=GQ150333) | 1396 | New Zealand | 29-Apr |
|  | [CY050991](http://www.ncbi.nlm.nih.gov/entrez/viewer.fcgi?val=CY050991) | 1734 | USA | 05-jun | 149 | [CY040033](http://www.ncbi.nlm.nih.gov/entrez/viewer.fcgi?val=CY040033) | 1413 | USA | 29-Apr |
|  | [CY051311](http://www.ncbi.nlm.nih.gov/entrez/viewer.fcgi?val=CY051311) | 1734 | USA | 08-jun | 150 | [GQ183609](http://www.ncbi.nlm.nih.gov/entrez/viewer.fcgi?val=GQ183609) | 1244 | Spain | 29-Apr |
|  | [CY051319](http://www.ncbi.nlm.nih.gov/entrez/viewer.fcgi?val=CY051319) | 1734 | USA | 08-jun | 150 | [GQ183610](http://www.ncbi.nlm.nih.gov/entrez/viewer.fcgi?val=GQ183610) | 1244 | Spain | 29-Apr |
|  | [CY051327](http://www.ncbi.nlm.nih.gov/entrez/viewer.fcgi?val=CY051327) | 1734 | USA | 08-jun | 151 | [GQ214152](http://www.ncbi.nlm.nih.gov/entrez/viewer.fcgi?val=GQ214152) | 1428 | France | 29-Apr |
|  | [CY051503](http://www.ncbi.nlm.nih.gov/entrez/viewer.fcgi?val=CY051503) | 1734 | USA | 08-jun | 152 | [CY041196](http://www.ncbi.nlm.nih.gov/entrez/viewer.fcgi?val=CY041196) | 1418 | USA | 29-Apr |
|  | [CY051343](http://www.ncbi.nlm.nih.gov/entrez/viewer.fcgi?val=CY051343) | 1734 | USA | 09-jun |  | [CY045055](http://www.ncbi.nlm.nih.gov/entrez/viewer.fcgi?val=CY045055) | 1418 | USA | 09-jun |
|  | [CY051351](http://www.ncbi.nlm.nih.gov/entrez/viewer.fcgi?val=CY051351) | 1734 | USA | 09-jun | 153 | [CY041484](http://www.ncbi.nlm.nih.gov/entrez/viewer.fcgi?val=CY041484) | 1412 | USA | 29-Apr |
|  | [CY051359](http://www.ncbi.nlm.nih.gov/entrez/viewer.fcgi?val=CY051359) | 1734 | USA | 10-jun | 154 | [GQ323524](http://www.ncbi.nlm.nih.gov/entrez/viewer.fcgi?val=GQ323524) | 1410 | USA | 29-Apr |
|  | [CY051383](http://www.ncbi.nlm.nih.gov/entrez/viewer.fcgi?val=CY051383) | 1734 | USA | 10-jun | 155 | [GQ323544](http://www.ncbi.nlm.nih.gov/entrez/viewer.fcgi?val=GQ323544) | 1410 | USA | 29-Apr |
|  | [CY051415](http://www.ncbi.nlm.nih.gov/entrez/viewer.fcgi?val=CY051415) | 1734 | USA | 15-jun | 156 | [GQ338341](http://www.ncbi.nlm.nih.gov/entrez/viewer.fcgi?val=GQ338341) | 1410 | USA | 29-Apr |
|  | [CY051431](http://www.ncbi.nlm.nih.gov/entrez/viewer.fcgi?val=CY051431) | 1734 | USA | 15-jun | 157 | [GQ338418](http://www.ncbi.nlm.nih.gov/entrez/viewer.fcgi?val=GQ338418) | 1410 | USA | 29-Apr |
|  | [CY051159](http://www.ncbi.nlm.nih.gov/entrez/viewer.fcgi?val=CY051159) | 1734 | USA | 16-jun | 158 | [GQ365660](http://www.ncbi.nlm.nih.gov/entrez/viewer.fcgi?val=GQ365660) | 1380 | Germany | 29-Apr |
|  | [CY051463](http://www.ncbi.nlm.nih.gov/entrez/viewer.fcgi?val=CY051463) | 1734 | USA | 16-jun | 159 | [GQ465698](http://www.ncbi.nlm.nih.gov/entrez/viewer.fcgi?val=GQ465698) | 1422 | Canada | 29-Apr |
|  | [CY051447](http://www.ncbi.nlm.nih.gov/entrez/viewer.fcgi?val=CY051447) | 1734 | USA | 18-jun |  | [GQ465699](http://www.ncbi.nlm.nih.gov/entrez/viewer.fcgi?val=GQ465699) | 1422 | Canada | 29-Apr |
|  | [CY051199](http://www.ncbi.nlm.nih.gov/entrez/viewer.fcgi?val=CY051199) | 1734 | USA | 22-jun |  | [GQ465707](http://www.ncbi.nlm.nih.gov/entrez/viewer.fcgi?val=GQ465707) | 1422 | Canada | 30-Apr |
|  | [CY051663](http://www.ncbi.nlm.nih.gov/entrez/viewer.fcgi?val=CY051663) | 1734 | USA | 25-jul | 160 | [CY046301](http://www.ncbi.nlm.nih.gov/entrez/viewer.fcgi?val=CY046301) | 1412 | USA | 29-Apr |
|  | [CY046307](http://www.ncbi.nlm.nih.gov/entrez/viewer.fcgi?val=CY046307) | 1734 | USA | 29-Apr |  | [CY046341](http://www.ncbi.nlm.nih.gov/entrez/viewer.fcgi?val=CY046341) | 1412 | USA | 02-may |
| 226 | [CY039527](http://www.ncbi.nlm.nih.gov/entrez/viewer.fcgi?val=CY039527) | 1777 | Netherlands | 29-Apr |  | [CY046373](http://www.ncbi.nlm.nih.gov/entrez/viewer.fcgi?val=CY046373) | 1412 | USA | 03-may |
| 227 | [CY047310](http://www.ncbi.nlm.nih.gov/entrez/viewer.fcgi?val=CY047310) | 1734 | USA | 29-Apr |  | [CY046749](http://www.ncbi.nlm.nih.gov/entrez/viewer.fcgi?val=CY046749) | 1412 | USA | 22-may |
| 228 | [CY053095](http://www.ncbi.nlm.nih.gov/entrez/viewer.fcgi?val=CY053095) | 1734 | USA | 01-may |  | [CY051049](http://www.ncbi.nlm.nih.gov/entrez/viewer.fcgi?val=CY051049) | 1412 | USA | 04-jun |
|  | [CY053103](http://www.ncbi.nlm.nih.gov/entrez/viewer.fcgi?val=CY053103) | 1734 | USA | 01-may |  | [CY051057](http://www.ncbi.nlm.nih.gov/entrez/viewer.fcgi?val=CY051057) | 1412 | USA | 08-jun |
|  | [CY053142](http://www.ncbi.nlm.nih.gov/entrez/viewer.fcgi?val=CY053142) | 1734 | USA | 04-may |  | [CY051065](http://www.ncbi.nlm.nih.gov/entrez/viewer.fcgi?val=CY051065) | 1412 | USA | 08-jun |
|  | [CY053150](http://www.ncbi.nlm.nih.gov/entrez/viewer.fcgi?val=CY053150) | 1734 | USA | 04-may |  | [CY051073](http://www.ncbi.nlm.nih.gov/entrez/viewer.fcgi?val=CY051073) | 1412 | USA | 08-jun |
|  | [CY050222](http://www.ncbi.nlm.nih.gov/entrez/viewer.fcgi?val=CY050222) | 1734 | Mexico | 08-may |  | [CY051337](http://www.ncbi.nlm.nih.gov/entrez/viewer.fcgi?val=CY051337) | 1412 | USA | 09-jun |
|  | [CY051879](http://www.ncbi.nlm.nih.gov/entrez/viewer.fcgi?val=CY051879) | 1734 | USA | 11-jun |  | [CY051353](http://www.ncbi.nlm.nih.gov/entrez/viewer.fcgi?val=CY051353) | 1412 | USA | 09-jun |
|  | [CY051543](http://www.ncbi.nlm.nih.gov/entrez/viewer.fcgi?val=CY051543) | 1734 | USA | 17-jun |  | [CY051361](http://www.ncbi.nlm.nih.gov/entrez/viewer.fcgi?val=CY051361) | 1412 | USA | 10-jun |
|  | [CY050548](http://www.ncbi.nlm.nih.gov/entrez/viewer.fcgi?val=CY050548) | 1723 | USA | 29-Apr |  | [CY051081](http://www.ncbi.nlm.nih.gov/entrez/viewer.fcgi?val=CY051081) | 1412 | USA | 11-jun |
| 229 | [CY050764](http://www.ncbi.nlm.nih.gov/entrez/viewer.fcgi?val=CY050764) | 1724 | USA | 29-Apr |  | [CY051105](http://www.ncbi.nlm.nih.gov/entrez/viewer.fcgi?val=CY051105) | 1412 | USA | 13-jun |
| 230 | [GQ122105](http://www.ncbi.nlm.nih.gov/entrez/viewer.fcgi?val=GQ122105) | 1186 | Sweden | 30-Apr | 161 | [CY046309](http://www.ncbi.nlm.nih.gov/entrez/viewer.fcgi?val=CY046309) | 1414 | USA | 29-Apr |
| 231 | [GQ166194](http://www.ncbi.nlm.nih.gov/entrez/viewer.fcgi?val=GQ166194) | 1553 | Spain | 30-Apr |  | [CY046461](http://www.ncbi.nlm.nih.gov/entrez/viewer.fcgi?val=CY046461) | 1414 | USA | 01-may |
| 232 | [GQ168606](http://www.ncbi.nlm.nih.gov/entrez/viewer.fcgi?val=GQ168606) | 1742 | Hong Kong | 30-Apr |  | [CY046189](http://www.ncbi.nlm.nih.gov/entrez/viewer.fcgi?val=CY046189) | 1414 | USA | 01-may |
| 233 | [GQ214138](http://www.ncbi.nlm.nih.gov/entrez/viewer.fcgi?val=GQ214138) | 1720 | France | 30-Apr |  | [CY046293](http://www.ncbi.nlm.nih.gov/entrez/viewer.fcgi?val=CY046293) | 1414 | USA | 01-may |
| 234 | [GQ214144](http://www.ncbi.nlm.nih.gov/entrez/viewer.fcgi?val=GQ214144) | 1749 | France | 30-Apr |  | [CY046381](http://www.ncbi.nlm.nih.gov/entrez/viewer.fcgi?val=CY046381) | 1414 | USA | 02-may |
| 235 | [CY040693](http://www.ncbi.nlm.nih.gov/entrez/viewer.fcgi?val=CY040693) | 1715 | USA | 30-Apr |  | [CY046229](http://www.ncbi.nlm.nih.gov/entrez/viewer.fcgi?val=CY046229) | 1414 | USA | 03-may |
| 236 | [GQ232009](http://www.ncbi.nlm.nih.gov/entrez/viewer.fcgi?val=GQ232009) | 1701 | USA | 30-Apr |  | [CY046213](http://www.ncbi.nlm.nih.gov/entrez/viewer.fcgi?val=CY046213) | 1414 | USA | 03-may |
| 237 | [GQ377064](http://www.ncbi.nlm.nih.gov/entrez/viewer.fcgi?val=GQ377064) | 1701 | USA | 06-may |  | [CY046221](http://www.ncbi.nlm.nih.gov/entrez/viewer.fcgi?val=CY046221) | 1414 | USA | 04-may |
| 238 | [CY041621](http://www.ncbi.nlm.nih.gov/entrez/viewer.fcgi?val=CY041621) | 1736 | USA | 30-Apr |  | [CY046869](http://www.ncbi.nlm.nih.gov/entrez/viewer.fcgi?val=CY046869) | 1414 | USA | 04-may |
| 239 | [GQ323443](http://www.ncbi.nlm.nih.gov/entrez/viewer.fcgi?val=GQ323443) | 1701 | USA | 30-Apr |  | [CY046877](http://www.ncbi.nlm.nih.gov/entrez/viewer.fcgi?val=CY046877) | 1414 | USA | 04-may |
| 240 | [GQ323451](http://www.ncbi.nlm.nih.gov/entrez/viewer.fcgi?val=GQ323451) | 1701 | USA | 30-Apr |  | [CY050446](http://www.ncbi.nlm.nih.gov/entrez/viewer.fcgi?val=CY050446) | 1414 | USA | 10-may |
| 241 | [GQ232007](http://www.ncbi.nlm.nih.gov/entrez/viewer.fcgi?val=GQ232007) | 1701 | USA | 04-may |  | [CY046757](http://www.ncbi.nlm.nih.gov/entrez/viewer.fcgi?val=CY046757) | 1414 | USA | 19-may |
|  | [GQ323551](http://www.ncbi.nlm.nih.gov/entrez/viewer.fcgi?val=GQ323551) | 1701 | USA | 07-may | 162 | [CY039528](http://www.ncbi.nlm.nih.gov/entrez/viewer.fcgi?val=CY039528) | 1458 | Netherlands | 29-Apr |
|  | [GQ323446](http://www.ncbi.nlm.nih.gov/entrez/viewer.fcgi?val=GQ323446) | 1701 | USA | 09-may | 163 | [CY047312](http://www.ncbi.nlm.nih.gov/entrez/viewer.fcgi?val=CY047312) | 1414 | USA | 29-Apr |
|  | [GQ368664](http://www.ncbi.nlm.nih.gov/entrez/viewer.fcgi?val=GQ368664) | 1701 | Brazil | 27-may | 164 | [CY050550](http://www.ncbi.nlm.nih.gov/entrez/viewer.fcgi?val=CY050550) | 1424 | USA | 29-Apr |
|  | [CY052349](http://www.ncbi.nlm.nih.gov/entrez/viewer.fcgi?val=CY052349) | 1701 | Brazil | 01-jun | 165 | [CY050766](http://www.ncbi.nlm.nih.gov/entrez/viewer.fcgi?val=CY050766) | 1423 | USA | 29-Apr |
|  | [GU014802](http://www.ncbi.nlm.nih.gov/entrez/viewer.fcgi?val=GU014802) | 1701 | Japan | 15-jun | 166 | [GQ122104](http://www.ncbi.nlm.nih.gov/entrez/viewer.fcgi?val=GQ122104) | 1358 | Sweden | 30-Apr |
|  | [CY052348](http://www.ncbi.nlm.nih.gov/entrez/viewer.fcgi?val=CY052348) | 1701 | Brazil | 29-jul | 167 | [GQ166189](http://www.ncbi.nlm.nih.gov/entrez/viewer.fcgi?val=GQ166189) | 1246 | Spain | 30-Apr |
|  | [GQ915025](http://www.ncbi.nlm.nih.gov/entrez/viewer.fcgi?val=GQ915025) | 1701 | Brazil | 9-Aug | 168 | [GQ214140](http://www.ncbi.nlm.nih.gov/entrez/viewer.fcgi?val=GQ214140) | 1428 | France | 30-Apr |
|  | [GQ377045](http://www.ncbi.nlm.nih.gov/entrez/viewer.fcgi?val=GQ377045) | 1701 | USA | 30-Apr |  | [GQ214146](http://www.ncbi.nlm.nih.gov/entrez/viewer.fcgi?val=GQ214146) | 1428 | France | 30-Apr |
| 242 | [GQ377101](http://www.ncbi.nlm.nih.gov/entrez/viewer.fcgi?val=GQ377101) | 1701 | USA | 30-Apr | 169 | [GQ323515](http://www.ncbi.nlm.nih.gov/entrez/viewer.fcgi?val=GQ323515) | 1410 | USA | 30-Apr |
|  | [GQ465681](http://www.ncbi.nlm.nih.gov/entrez/viewer.fcgi?val=GQ465681) | 1743 | Canada | 30-Apr |  | [GQ377063](http://www.ncbi.nlm.nih.gov/entrez/viewer.fcgi?val=GQ377063) | 1410 | USA | 06-may |
| 243 | [GQ402198](http://www.ncbi.nlm.nih.gov/entrez/viewer.fcgi?val=GQ402198) | 1743 | Canada | 06-may | 170 | [GQ323562](http://www.ncbi.nlm.nih.gov/entrez/viewer.fcgi?val=GQ323562) | 1410 | USA | 30-Apr |
|  | [GQ465682](http://www.ncbi.nlm.nih.gov/entrez/viewer.fcgi?val=GQ465682) | 1743 | Canada | 07-may |  | [GQ433898](http://www.ncbi.nlm.nih.gov/entrez/viewer.fcgi?val=GQ433898) | 1410 | China | 15-jun |
|  | [GQ465683](http://www.ncbi.nlm.nih.gov/entrez/viewer.fcgi?val=GQ465683) | 1743 | Canada | 07-may | 171 | [GQ377044](http://www.ncbi.nlm.nih.gov/entrez/viewer.fcgi?val=GQ377044) | 1410 | USA | 30-Apr |
|  | [GQ476145](http://www.ncbi.nlm.nih.gov/entrez/viewer.fcgi?val=GQ476145) | 1698 | USA | 30-Apr | 172 | [GQ465704](http://www.ncbi.nlm.nih.gov/entrez/viewer.fcgi?val=GQ465704) | 1422 | Canada | 30-Apr |
| 244 | [GQ894862](http://www.ncbi.nlm.nih.gov/entrez/viewer.fcgi?val=GQ894862) | 1701 | USA | 30-Apr |  | [GQ402238](http://www.ncbi.nlm.nih.gov/entrez/viewer.fcgi?val=GQ402238) | 1422 | Canada | 06-may |
| 245 | [GQ259909](http://www.ncbi.nlm.nih.gov/entrez/viewer.fcgi?val=GQ259909) | 1701 | China | 13-jun |  | [GQ465705](http://www.ncbi.nlm.nih.gov/entrez/viewer.fcgi?val=GQ465705) | 1422 | Canada | 07-may |
|  | [CY046259](http://www.ncbi.nlm.nih.gov/entrez/viewer.fcgi?val=CY046259) | 1701 | USA | 30-Apr |  | [GQ465706](http://www.ncbi.nlm.nih.gov/entrez/viewer.fcgi?val=GQ465706) | 1422 | Canada | 07-may |
| 246 | [CY046387](http://www.ncbi.nlm.nih.gov/entrez/viewer.fcgi?val=CY046387) | 1733 | USA | 30-Apr | 173 | [GQ476146](http://www.ncbi.nlm.nih.gov/entrez/viewer.fcgi?val=GQ476146) | 1413 | USA | 30-Apr |
| 247 | [CY046451](http://www.ncbi.nlm.nih.gov/entrez/viewer.fcgi?val=CY046451) | 1736 | USA | 30-Apr | 174 | [CY046261](http://www.ncbi.nlm.nih.gov/entrez/viewer.fcgi?val=CY046261) | 1413 | USA | 30-Apr |
| 248 | [CY046475](http://www.ncbi.nlm.nih.gov/entrez/viewer.fcgi?val=CY046475) | 1734 | USA | 30-Apr | 175 | [CY046357](http://www.ncbi.nlm.nih.gov/entrez/viewer.fcgi?val=CY046357) | 1417 | USA | 30-Apr |
| 249 | [CY046515](http://www.ncbi.nlm.nih.gov/entrez/viewer.fcgi?val=CY046515) | 1707 | USA | 30-Apr |  | [CY046429](http://www.ncbi.nlm.nih.gov/entrez/viewer.fcgi?val=CY046429) | 1417 | USA | 01-may |
| 250 | [CY046251](http://www.ncbi.nlm.nih.gov/entrez/viewer.fcgi?val=CY046251) | 1707 | USA | 04-may |  | [CY046885](http://www.ncbi.nlm.nih.gov/entrez/viewer.fcgi?val=CY046885) | 1417 | USA | 03-may |
|  | [CY046843](http://www.ncbi.nlm.nih.gov/entrez/viewer.fcgi?val=CY046843) | 1734 | USA | 30-Apr | 176 | [CY046389](http://www.ncbi.nlm.nih.gov/entrez/viewer.fcgi?val=CY046389) | 1414 | USA | 30-Apr |
| 251 | [CY047350](http://www.ncbi.nlm.nih.gov/entrez/viewer.fcgi?val=CY047350) | 1727 | USA | 30-Apr | 177 | [CY046413](http://www.ncbi.nlm.nih.gov/entrez/viewer.fcgi?val=CY046413) | 1423 | USA | 30-Apr |
| 252 | [CY049828](http://www.ncbi.nlm.nih.gov/entrez/viewer.fcgi?val=CY049828) | 1698 | USA | 30-Apr | 178 | [CY046421](http://www.ncbi.nlm.nih.gov/entrez/viewer.fcgi?val=CY046421) | 1420 | USA | 30-Apr |
| 253 | [CY039986](http://www.ncbi.nlm.nih.gov/entrez/viewer.fcgi?val=CY039986) | 1770 | Thailand | May |  | [CY046317](http://www.ncbi.nlm.nih.gov/entrez/viewer.fcgi?val=CY046317) | 1420 | USA | 01-may |
| 254 | [GQ149765](http://www.ncbi.nlm.nih.gov/entrez/viewer.fcgi?val=GQ149765) | 909 | Italy | May |  | [CY046397](http://www.ncbi.nlm.nih.gov/entrez/viewer.fcgi?val=CY046397) | 1420 | USA | 02-may |
| 255 | [GQ144465](http://www.ncbi.nlm.nih.gov/entrez/viewer.fcgi?val=GQ144465) | 1547 | Spain | May |  | [CY046405](http://www.ncbi.nlm.nih.gov/entrez/viewer.fcgi?val=CY046405) | 1420 | USA | 04-may |
| 256 | [GQ152374](http://www.ncbi.nlm.nih.gov/entrez/viewer.fcgi?val=GQ152374) | 1547 | Spain | May |  | [CY046469](http://www.ncbi.nlm.nih.gov/entrez/viewer.fcgi?val=CY046469) | 1420 | USA | 04-may |
| 257 | [GQ258462](http://www.ncbi.nlm.nih.gov/entrez/viewer.fcgi?val=GQ258462) | 1701 | Australia | May |  | [CY046525](http://www.ncbi.nlm.nih.gov/entrez/viewer.fcgi?val=CY046525) | 1420 | USA | 04-may |
| 258 | [GQ377035](http://www.ncbi.nlm.nih.gov/entrez/viewer.fcgi?val=GQ377035) | 1701 | USA | May |  | [CY046533](http://www.ncbi.nlm.nih.gov/entrez/viewer.fcgi?val=CY046533) | 1420 | USA | 04-may |
| 259 | [CY052093](http://www.ncbi.nlm.nih.gov/entrez/viewer.fcgi?val=CY052093) | 970 | Ecuador | May |  | [CY050216](http://www.ncbi.nlm.nih.gov/entrez/viewer.fcgi?val=CY050216) | 1420 | Mexico | 08-may |
| 260 | [CY053654](http://www.ncbi.nlm.nih.gov/entrez/viewer.fcgi?val=CY053654) | 1763 | Russia | May |  | [CY050330](http://www.ncbi.nlm.nih.gov/entrez/viewer.fcgi?val=CY050330) | 1420 | USA | 12-may |
| 261 | [GQ166195](http://www.ncbi.nlm.nih.gov/entrez/viewer.fcgi?val=GQ166195) | 1541 | Spain | 01-may |  | [CY046669](http://www.ncbi.nlm.nih.gov/entrez/viewer.fcgi?val=CY046669) | 1420 | USA | 14-may |
| 262 | [GQ166196](http://www.ncbi.nlm.nih.gov/entrez/viewer.fcgi?val=GQ166196) | 1550 | Spain | 01-may |  | [CY050382](http://www.ncbi.nlm.nih.gov/entrez/viewer.fcgi?val=CY050382) | 1420 | USA | 15-may |
| 263 | [GQ166197](http://www.ncbi.nlm.nih.gov/entrez/viewer.fcgi?val=GQ166197) | 1544 | Spain | 01-may |  | [CY050390](http://www.ncbi.nlm.nih.gov/entrez/viewer.fcgi?val=CY050390) | 1420 | USA | 15-may |
| 264 | [GQ166198](http://www.ncbi.nlm.nih.gov/entrez/viewer.fcgi?val=GQ166198) | 860 | Spain | 01-may |  | [CY046629](http://www.ncbi.nlm.nih.gov/entrez/viewer.fcgi?val=CY046629) | 1420 | USA | 15-may |
| 265 | [CY040457](http://www.ncbi.nlm.nih.gov/entrez/viewer.fcgi?val=CY040457) | 1718 | USA | 01-may |  | [CY050398](http://www.ncbi.nlm.nih.gov/entrez/viewer.fcgi?val=CY050398) | 1420 | USA | 17-may |
| 266 | [GQ214156](http://www.ncbi.nlm.nih.gov/entrez/viewer.fcgi?val=GQ214156) | 1738 | France | 01-may |  | [CY050414](http://www.ncbi.nlm.nih.gov/entrez/viewer.fcgi?val=CY050414) | 1420 | USA | 19-may |
| 267 | [GQ231987](http://www.ncbi.nlm.nih.gov/entrez/viewer.fcgi?val=GQ231987) | 1701 | USA | 01-may |  | [CY050406](http://www.ncbi.nlm.nih.gov/entrez/viewer.fcgi?val=CY050406) | 1420 | USA | 23-may |
| 268 | [GQ232067](http://www.ncbi.nlm.nih.gov/entrez/viewer.fcgi?val=GQ232067) | 1701 | USA | 01-may |  | [CY050897](http://www.ncbi.nlm.nih.gov/entrez/viewer.fcgi?val=CY050897) | 1420 | USA | 27-may |
| 269 | [GQ323473](http://www.ncbi.nlm.nih.gov/entrez/viewer.fcgi?val=GQ323473) | 1701 | USA | 01-may |  | [CY050905](http://www.ncbi.nlm.nih.gov/entrez/viewer.fcgi?val=CY050905) | 1420 | USA | 28-may |
|  | [GQ249333](http://www.ncbi.nlm.nih.gov/entrez/viewer.fcgi?val=GQ249333) | 1701 | France | 01-may |  | [CY050937](http://www.ncbi.nlm.nih.gov/entrez/viewer.fcgi?val=CY050937) | 1420 | USA | 29-may |
| 270 | [CY041637](http://www.ncbi.nlm.nih.gov/entrez/viewer.fcgi?val=CY041637) | 1736 | USA | 01-may |  | [CY051257](http://www.ncbi.nlm.nih.gov/entrez/viewer.fcgi?val=CY051257) | 1420 | USA | 29-may |
| 271 | [GQ323560](http://www.ncbi.nlm.nih.gov/entrez/viewer.fcgi?val=GQ323560) | 1701 | USA | 01-may |  | [CY050969](http://www.ncbi.nlm.nih.gov/entrez/viewer.fcgi?val=CY050969) | 1420 | USA | 01-jun |
| 272 | [GQ338378](http://www.ncbi.nlm.nih.gov/entrez/viewer.fcgi?val=GQ338378) | 1701 | USA | 01-may |  | [CY050985](http://www.ncbi.nlm.nih.gov/entrez/viewer.fcgi?val=CY050985) | 1420 | USA | 02-jun |
| 273 | [GQ377085](http://www.ncbi.nlm.nih.gov/entrez/viewer.fcgi?val=GQ377085) | 1701 | USA | 01-may |  | [CY051265](http://www.ncbi.nlm.nih.gov/entrez/viewer.fcgi?val=CY051265) | 1420 | USA | 02-jun |
| 274 | [GQ457517](http://www.ncbi.nlm.nih.gov/entrez/viewer.fcgi?val=GQ457517) | 1701 | USA | 01-may |  | [CY051281](http://www.ncbi.nlm.nih.gov/entrez/viewer.fcgi?val=CY051281) | 1420 | USA | 02-jun |
| 275 | [GQ465679](http://www.ncbi.nlm.nih.gov/entrez/viewer.fcgi?val=GQ465679) | 1743 | Canada | 01-may |  | [CY051025](http://www.ncbi.nlm.nih.gov/entrez/viewer.fcgi?val=CY051025) | 1420 | USA | 04-jun |
| 276 | [CY046187](http://www.ncbi.nlm.nih.gov/entrez/viewer.fcgi?val=CY046187) | 1733 | USA | 01-may |  | [CY051305](http://www.ncbi.nlm.nih.gov/entrez/viewer.fcgi?val=CY051305) | 1420 | USA | 08-jun |
| 277 | [CY046235](http://www.ncbi.nlm.nih.gov/entrez/viewer.fcgi?val=CY046235) | 1733 | USA | 02-may |  | [CY051321](http://www.ncbi.nlm.nih.gov/entrez/viewer.fcgi?val=CY051321) | 1420 | USA | 08-jun |
|  | [CY046291](http://www.ncbi.nlm.nih.gov/entrez/viewer.fcgi?val=CY046291) | 1733 | USA | 01-may |  | [CY051241](http://www.ncbi.nlm.nih.gov/entrez/viewer.fcgi?val=CY051241) | 1420 | USA | 09-jun |
| 278 | [CY046459](http://www.ncbi.nlm.nih.gov/entrez/viewer.fcgi?val=CY046459) | 1733 | USA | 01-may |  | [CY051513](http://www.ncbi.nlm.nih.gov/entrez/viewer.fcgi?val=CY051513) | 1420 | USA | 09-jun |
|  | [CY046435](http://www.ncbi.nlm.nih.gov/entrez/viewer.fcgi?val=CY046435) | 1733 | USA | 02-may |  | [CY051369](http://www.ncbi.nlm.nih.gov/entrez/viewer.fcgi?val=CY051369) | 1420 | USA | 10-jun |
|  | [CY046467](http://www.ncbi.nlm.nih.gov/entrez/viewer.fcgi?val=CY046467) | 1733 | USA | 04-may |  | [CY051385](http://www.ncbi.nlm.nih.gov/entrez/viewer.fcgi?val=CY051385) | 1420 | USA | 10-jun |
|  | [CY050444](http://www.ncbi.nlm.nih.gov/entrez/viewer.fcgi?val=CY050444) | 1733 | USA | 10-may |  | [CY051097](http://www.ncbi.nlm.nih.gov/entrez/viewer.fcgi?val=CY051097) | 1420 | USA | 13-jun |
|  | [CY050388](http://www.ncbi.nlm.nih.gov/entrez/viewer.fcgi?val=CY050388) | 1733 | USA | 15-may |  | [CY051161](http://www.ncbi.nlm.nih.gov/entrez/viewer.fcgi?val=CY051161) | 1420 | USA | 16-jun |
|  | [CY050396](http://www.ncbi.nlm.nih.gov/entrez/viewer.fcgi?val=CY050396) | 1733 | USA | 17-may | 179 | [CY046453](http://www.ncbi.nlm.nih.gov/entrez/viewer.fcgi?val=CY046453) | 1413 | USA | 30-Apr |
|  | [CY046723](http://www.ncbi.nlm.nih.gov/entrez/viewer.fcgi?val=CY046723) | 1733 | USA | 20-may |  | [CY046237](http://www.ncbi.nlm.nih.gov/entrez/viewer.fcgi?val=CY046237) | 1413 | USA | 02-may |
|  | [CY050404](http://www.ncbi.nlm.nih.gov/entrez/viewer.fcgi?val=CY050404) | 1733 | USA | 23-may |  | [CY046437](http://www.ncbi.nlm.nih.gov/entrez/viewer.fcgi?val=CY046437) | 1413 | USA | 02-may |
|  | [CY050428](http://www.ncbi.nlm.nih.gov/entrez/viewer.fcgi?val=CY050428) | 1733 | USA | 25-may |  | [CY046861](http://www.ncbi.nlm.nih.gov/entrez/viewer.fcgi?val=CY046861) | 1413 | USA | 04-may |
|  | [CY050436](http://www.ncbi.nlm.nih.gov/entrez/viewer.fcgi?val=CY050436) | 1733 | USA | 27-may |  | [CY046573](http://www.ncbi.nlm.nih.gov/entrez/viewer.fcgi?val=CY046573) | 1413 | USA | 08-may |
|  | [CY050895](http://www.ncbi.nlm.nih.gov/entrez/viewer.fcgi?val=CY050895) | 1733 | USA | 27-may | 180 | [CY046477](http://www.ncbi.nlm.nih.gov/entrez/viewer.fcgi?val=CY046477) | 1418 | USA | 30-Apr |
|  | [CY050927](http://www.ncbi.nlm.nih.gov/entrez/viewer.fcgi?val=CY050927) | 1733 | USA | 29-may |  | [CY046325](http://www.ncbi.nlm.nih.gov/entrez/viewer.fcgi?val=CY046325) | 1418 | USA | 04-may |
|  | [CY050943](http://www.ncbi.nlm.nih.gov/entrez/viewer.fcgi?val=CY050943) | 1733 | USA | 01-jun |  | [CY050200](http://www.ncbi.nlm.nih.gov/entrez/viewer.fcgi?val=CY050200) | 1418 | Mexico | 07-may |
|  | [CY051303](http://www.ncbi.nlm.nih.gov/entrez/viewer.fcgi?val=CY051303) | 1733 | USA | 08-jun |  | [CY046725](http://www.ncbi.nlm.nih.gov/entrez/viewer.fcgi?val=CY046725) | 1418 | USA | 20-may |
|  | [CY046315](http://www.ncbi.nlm.nih.gov/entrez/viewer.fcgi?val=CY046315) | 1715 | USA | 01-may |  | [CY050977](http://www.ncbi.nlm.nih.gov/entrez/viewer.fcgi?val=CY050977) | 1418 | USA | 02-jun |
| 279 | [CY046427](http://www.ncbi.nlm.nih.gov/entrez/viewer.fcgi?val=CY046427) | 1720 | USA | 01-may |  | [CY051425](http://www.ncbi.nlm.nih.gov/entrez/viewer.fcgi?val=CY051425) | 1418 | USA | 15-jun |
| 280 | [CY046491](http://www.ncbi.nlm.nih.gov/entrez/viewer.fcgi?val=CY046491) | 1720 | USA | 01-may | 181 | [CY046517](http://www.ncbi.nlm.nih.gov/entrez/viewer.fcgi?val=CY046517) | 1416 | USA | 30-Apr |
| 281 | [CY046267](http://www.ncbi.nlm.nih.gov/entrez/viewer.fcgi?val=CY046267) | 1720 | USA | 02-may |  | [CY046269](http://www.ncbi.nlm.nih.gov/entrez/viewer.fcgi?val=CY046269) | 1416 | USA | 02-may |
|  | [CY046691](http://www.ncbi.nlm.nih.gov/entrez/viewer.fcgi?val=CY046691) | 1720 | USA | 16-may |  | [CY046277](http://www.ncbi.nlm.nih.gov/entrez/viewer.fcgi?val=CY046277) | 1416 | USA | 03-may |
|  | [CY046971](http://www.ncbi.nlm.nih.gov/entrez/viewer.fcgi?val=CY046971) | 1733 | USA | 01-may |  | [CY046485](http://www.ncbi.nlm.nih.gov/entrez/viewer.fcgi?val=CY046485) | 1416 | USA | 04-may |
| 282 | [CY053079](http://www.ncbi.nlm.nih.gov/entrez/viewer.fcgi?val=CY053079) | 1734 | USA | 01-may |  | [CY046509](http://www.ncbi.nlm.nih.gov/entrez/viewer.fcgi?val=CY046509) | 1416 | USA | 04-may |
| 283 | [CY053087](http://www.ncbi.nlm.nih.gov/entrez/viewer.fcgi?val=CY053087) | 1734 | USA | 01-may |  | [CY046597](http://www.ncbi.nlm.nih.gov/entrez/viewer.fcgi?val=CY046597) | 1416 | USA | 10-may |
|  | [GQ131023](http://www.ncbi.nlm.nih.gov/entrez/viewer.fcgi?val=GQ131023) | 1701 | South Korea | 02-may |  | [CY046765](http://www.ncbi.nlm.nih.gov/entrez/viewer.fcgi?val=CY046765) | 1416 | USA | 22-may |
| 284 | [CY041597](http://www.ncbi.nlm.nih.gov/entrez/viewer.fcgi?val=CY041597) | 1728 | USA | 02-may | 182 | [CY046845](http://www.ncbi.nlm.nih.gov/entrez/viewer.fcgi?val=CY046845) | 1420 | USA | 30-Apr |
| 285 | [CY041806](http://www.ncbi.nlm.nih.gov/entrez/viewer.fcgi?val=CY041806) | 1733 | USA | 02-may | 183 | [CY039988](http://www.ncbi.nlm.nih.gov/entrez/viewer.fcgi?val=CY039988) | 1410 | Thailand | May |
| 286 | [CY047334](http://www.ncbi.nlm.nih.gov/entrez/viewer.fcgi?val=CY047334) | 1733 | USA | 06-may | 184 | [GQ144464](http://www.ncbi.nlm.nih.gov/entrez/viewer.fcgi?val=GQ144464) | 1238 | Spain | May |
|  | [CY044128](http://www.ncbi.nlm.nih.gov/entrez/viewer.fcgi?val=CY044128) | 1733 | USA | 17-may | 185 | [GQ152373](http://www.ncbi.nlm.nih.gov/entrez/viewer.fcgi?val=GQ152373) | 1243 | Spain | May |
|  | [CY044869](http://www.ncbi.nlm.nih.gov/entrez/viewer.fcgi?val=CY044869) | 1733 | USA | 20-may | 186 | [GQ258463](http://www.ncbi.nlm.nih.gov/entrez/viewer.fcgi?val=GQ258463) | 1396 | Australia | May |
|  | [CY043259](http://www.ncbi.nlm.nih.gov/entrez/viewer.fcgi?val=CY043259) | 1733 | USA | 20-may | 187 | [CY053655](http://www.ncbi.nlm.nih.gov/entrez/viewer.fcgi?val=CY053655) | 1453 | Russia | May |
|  | [CY045045](http://www.ncbi.nlm.nih.gov/entrez/viewer.fcgi?val=CY045045) | 1733 | USA | 10-jun | 188 | [GQ166191](http://www.ncbi.nlm.nih.gov/entrez/viewer.fcgi?val=GQ166191) | 1239 | Spain | 01-may |
|  | [GQ365666](http://www.ncbi.nlm.nih.gov/entrez/viewer.fcgi?val=GQ365666) | 1701 | Germany | 02-may | 189 | [GQ166192](http://www.ncbi.nlm.nih.gov/entrez/viewer.fcgi?val=GQ166192) | 1232 | Spain | 01-may |
| 287 | [GQ894905](http://www.ncbi.nlm.nih.gov/entrez/viewer.fcgi?val=GQ894905) | 1701 | USA | 02-may | 190 | [GQ166193](http://www.ncbi.nlm.nih.gov/entrez/viewer.fcgi?val=GQ166193) | 1211 | Spain | 01-may |
| 288 | [CY046195](http://www.ncbi.nlm.nih.gov/entrez/viewer.fcgi?val=CY046195) | 1732 | USA | 02-may | 191 | [GQ169710](http://www.ncbi.nlm.nih.gov/entrez/viewer.fcgi?val=GQ169710) | 805 | Ireland | 01-may |
| 289 | [CY046203](http://www.ncbi.nlm.nih.gov/entrez/viewer.fcgi?val=CY046203) | 1732 | USA | 02-may | 192 | [CY040458](http://www.ncbi.nlm.nih.gov/entrez/viewer.fcgi?val=CY040458) | 1397 | USA | 01-may |
| 290 | [CY046891](http://www.ncbi.nlm.nih.gov/entrez/viewer.fcgi?val=CY046891) | 1732 | USA | 04-may | 193 | [GQ254710](http://www.ncbi.nlm.nih.gov/entrez/viewer.fcgi?val=GQ254710) | 1410 | France | 01-may |
|  | [CY046851](http://www.ncbi.nlm.nih.gov/entrez/viewer.fcgi?val=CY046851) | 1734 | USA | 02-may | 194 | [CY041639](http://www.ncbi.nlm.nih.gov/entrez/viewer.fcgi?val=CY041639) | 1412 | USA | 01-may |
| 291 | [CY049859](http://www.ncbi.nlm.nih.gov/entrez/viewer.fcgi?val=CY049859) | 1698 | USA | 02-may | 195 | [GQ323472](http://www.ncbi.nlm.nih.gov/entrez/viewer.fcgi?val=GQ323472) | 1410 | USA | 01-may |
| 292 | [CY049923](http://www.ncbi.nlm.nih.gov/entrez/viewer.fcgi?val=CY049923) | 1698 | USA | 14-jun | 196 | [GQ323559](http://www.ncbi.nlm.nih.gov/entrez/viewer.fcgi?val=GQ323559) | 1410 | USA | 01-may |
|  | [CY049867](http://www.ncbi.nlm.nih.gov/entrez/viewer.fcgi?val=CY049867) | 1701 | USA | 02-may | 197 | [GQ323570](http://www.ncbi.nlm.nih.gov/entrez/viewer.fcgi?val=GQ323570) | 1410 | USA | 01-may |
| 293 | [CY049875](http://www.ncbi.nlm.nih.gov/entrez/viewer.fcgi?val=CY049875) | 1698 | USA | 02-may | 198 | [GQ338380](http://www.ncbi.nlm.nih.gov/entrez/viewer.fcgi?val=GQ338380) | 1410 | USA | 01-may |
| 294 | [CY049843](http://www.ncbi.nlm.nih.gov/entrez/viewer.fcgi?val=CY049843) | 1698 | USA | 04-may | 199 | [CY046493](http://www.ncbi.nlm.nih.gov/entrez/viewer.fcgi?val=CY046493) | 1418 | USA | 01-may |
|  | [CY054296](http://www.ncbi.nlm.nih.gov/entrez/viewer.fcgi?val=CY054296) | 1719 | Mexico | 02-may |  | [CY046501](http://www.ncbi.nlm.nih.gov/entrez/viewer.fcgi?val=CY046501) | 1418 | USA | 04-may |
| 295 | [GQ251035](http://www.ncbi.nlm.nih.gov/entrez/viewer.fcgi?val=GQ251035) | 1747 | Italy | 03-may |  | [CY046557](http://www.ncbi.nlm.nih.gov/entrez/viewer.fcgi?val=CY046557) | 1418 | USA | 06-may |
| 296 | [CY046243](http://www.ncbi.nlm.nih.gov/entrez/viewer.fcgi?val=CY046243) | 1734 | USA | 03-may |  | [CY046581](http://www.ncbi.nlm.nih.gov/entrez/viewer.fcgi?val=CY046581) | 1418 | USA | 08-may |
| 297 | [CY046275](http://www.ncbi.nlm.nih.gov/entrez/viewer.fcgi?val=CY046275) | 1719 | USA | 03-may |  | [CY046549](http://www.ncbi.nlm.nih.gov/entrez/viewer.fcgi?val=CY046549) | 1418 | USA | 10-may |
| 298 | [CY046371](http://www.ncbi.nlm.nih.gov/entrez/viewer.fcgi?val=CY046371) | 1731 | USA | 03-may |  | [CY046693](http://www.ncbi.nlm.nih.gov/entrez/viewer.fcgi?val=CY046693) | 1418 | USA | 16-may |
| 299 | [GQ232073](http://www.ncbi.nlm.nih.gov/entrez/viewer.fcgi?val=GQ232073) | 1701 | USA | 04-may |  | [CY046717](http://www.ncbi.nlm.nih.gov/entrez/viewer.fcgi?val=CY046717) | 1418 | USA | 19-may |
| 300 | [CY041645](http://www.ncbi.nlm.nih.gov/entrez/viewer.fcgi?val=CY041645) | 1735 | USA | 04-may |  | [CY046837](http://www.ncbi.nlm.nih.gov/entrez/viewer.fcgi?val=CY046837) | 1418 | USA | 27-may |
| 301 | [GQ323464](http://www.ncbi.nlm.nih.gov/entrez/viewer.fcgi?val=GQ323464) | 1701 | USA | 04-may |  | [CY051121](http://www.ncbi.nlm.nih.gov/entrez/viewer.fcgi?val=CY051121) | 1418 | USA | 14-jun |
| 302 | [GQ323470](http://www.ncbi.nlm.nih.gov/entrez/viewer.fcgi?val=GQ323470) | 1701 | USA | 04-may |  | [CY051129](http://www.ncbi.nlm.nih.gov/entrez/viewer.fcgi?val=CY051129) | 1418 | USA | 14-jun |
| 303 | [CY041782](http://www.ncbi.nlm.nih.gov/entrez/viewer.fcgi?val=CY041782) | 1732 | USA | 04-may | 200 | [CY046973](http://www.ncbi.nlm.nih.gov/entrez/viewer.fcgi?val=CY046973) | 1419 | USA | 01-may |
| 304 | [GQ365674](http://www.ncbi.nlm.nih.gov/entrez/viewer.fcgi?val=GQ365674) | 1725 | Germany | 04-may |  | [CY043173](http://www.ncbi.nlm.nih.gov/entrez/viewer.fcgi?val=CY043173) | 1419 | USA | 04-may |
| 305 | [CY043171](http://www.ncbi.nlm.nih.gov/entrez/viewer.fcgi?val=CY043171) | 1734 | USA | 04-may | 201 | [CY047344](http://www.ncbi.nlm.nih.gov/entrez/viewer.fcgi?val=CY047344) | 1421 | USA | 01-may |
| 306 | [CY043203](http://www.ncbi.nlm.nih.gov/entrez/viewer.fcgi?val=CY043203) | 1734 | USA | 04-may | 202 | [CY053097](http://www.ncbi.nlm.nih.gov/entrez/viewer.fcgi?val=CY053097) | 1420 | USA | 01-may |
| 307 | [CY043299](http://www.ncbi.nlm.nih.gov/entrez/viewer.fcgi?val=CY043299) | 1734 | USA | 12-may | 203 | [CY053105](http://www.ncbi.nlm.nih.gov/entrez/viewer.fcgi?val=CY053105) | 1420 | USA | 01-may |
|  | [CY043187](http://www.ncbi.nlm.nih.gov/entrez/viewer.fcgi?val=CY043187) | 1734 | USA | 16-may | 204 | [GQ132185](http://www.ncbi.nlm.nih.gov/entrez/viewer.fcgi?val=GQ132185) | 1410 | South Korea | 02-may |
|  | [CY044989](http://www.ncbi.nlm.nih.gov/entrez/viewer.fcgi?val=CY044989) | 1734 | USA | 01-jun | 205 | [CY041076](http://www.ncbi.nlm.nih.gov/entrez/viewer.fcgi?val=CY041076) | 1419 | USA | 02-may |
|  | [CY052274](http://www.ncbi.nlm.nih.gov/entrez/viewer.fcgi?val=CY052274) | 1734 | USA | 15-jun |  | [CY041808](http://www.ncbi.nlm.nih.gov/entrez/viewer.fcgi?val=CY041808) | 1419 | USA | 02-may |
|  | [CY052455](http://www.ncbi.nlm.nih.gov/entrez/viewer.fcgi?val=CY052455) | 1734 | USA | 12-sep |  | [CY041824](http://www.ncbi.nlm.nih.gov/entrez/viewer.fcgi?val=CY041824) | 1419 | USA | 06-may |
|  | [CY052471](http://www.ncbi.nlm.nih.gov/entrez/viewer.fcgi?val=CY052471) | 1734 | USA | 12-sep |  | [CY043189](http://www.ncbi.nlm.nih.gov/entrez/viewer.fcgi?val=CY043189) | 1419 | USA | 16-may |
|  | [CY045482](http://www.ncbi.nlm.nih.gov/entrez/viewer.fcgi?val=CY045482) | 1742 | Germany | 04-may |  | [CY044130](http://www.ncbi.nlm.nih.gov/entrez/viewer.fcgi?val=CY044130) | 1419 | USA | 17-may |
| 308 | [CY046323](http://www.ncbi.nlm.nih.gov/entrez/viewer.fcgi?val=CY046323) | 1734 | USA | 04-may |  | [CY045031](http://www.ncbi.nlm.nih.gov/entrez/viewer.fcgi?val=CY045031) | 1419 | USA | 31-may |
| 309 | [CY046363](http://www.ncbi.nlm.nih.gov/entrez/viewer.fcgi?val=CY046363) | 1734 | USA | 04-may |  | [CY044991](http://www.ncbi.nlm.nih.gov/entrez/viewer.fcgi?val=CY044991) | 1419 | USA | 01-jun |
| 310 | [CY046443](http://www.ncbi.nlm.nih.gov/entrez/viewer.fcgi?val=CY046443) | 1734 | USA | 04-may |  | [CY045023](http://www.ncbi.nlm.nih.gov/entrez/viewer.fcgi?val=CY045023) | 1419 | USA | 02-jun |
|  | [CY046771](http://www.ncbi.nlm.nih.gov/entrez/viewer.fcgi?val=CY046771) | 1734 | USA | 19-may |  | [CY045217](http://www.ncbi.nlm.nih.gov/entrez/viewer.fcgi?val=CY045217) | 1419 | USA | 10-jun |
|  | [CY051039](http://www.ncbi.nlm.nih.gov/entrez/viewer.fcgi?val=CY051039) | 1734 | USA | 04-jun |  | [CY051153](http://www.ncbi.nlm.nih.gov/entrez/viewer.fcgi?val=CY051153) | 1419 | USA | 14-jun |
|  | [CY046403](http://www.ncbi.nlm.nih.gov/entrez/viewer.fcgi?val=CY046403) | 1735 | USA | 04-may | 206 | [CY041599](http://www.ncbi.nlm.nih.gov/entrez/viewer.fcgi?val=CY041599) | 1414 | USA | 02-may |
| 311 | [CY046483](http://www.ncbi.nlm.nih.gov/entrez/viewer.fcgi?val=CY046483) | 1720 | USA | 04-may | 207 | [GQ338342](http://www.ncbi.nlm.nih.gov/entrez/viewer.fcgi?val=GQ338342) | 1410 | USA | 02-may |
| 312 | [CY046499](http://www.ncbi.nlm.nih.gov/entrez/viewer.fcgi?val=CY046499) | 1721 | USA | 04-may | 208 | [GQ365668](http://www.ncbi.nlm.nih.gov/entrez/viewer.fcgi?val=GQ365668) | 1395 | Germany | 02-may |
| 313 | [CY046507](http://www.ncbi.nlm.nih.gov/entrez/viewer.fcgi?val=CY046507) | 1721 | USA | 04-may | 209 | [CY046197](http://www.ncbi.nlm.nih.gov/entrez/viewer.fcgi?val=CY046197) | 1419 | USA | 02-may |
|  | [CY046579](http://www.ncbi.nlm.nih.gov/entrez/viewer.fcgi?val=CY046579) | 1721 | USA | 08-may | 210 | [CY046205](http://www.ncbi.nlm.nih.gov/entrez/viewer.fcgi?val=CY046205) | 1413 | USA | 02-may |
|  | [CY046595](http://www.ncbi.nlm.nih.gov/entrez/viewer.fcgi?val=CY046595) | 1721 | USA | 10-may | 211 | [CY046853](http://www.ncbi.nlm.nih.gov/entrez/viewer.fcgi?val=CY046853) | 1419 | USA | 02-may |
|  | [CY046683](http://www.ncbi.nlm.nih.gov/entrez/viewer.fcgi?val=CY046683) | 1721 | USA | 14-may | 212 | [CY054297](http://www.ncbi.nlm.nih.gov/entrez/viewer.fcgi?val=CY054297) | 1413 | Mexico | 02-may |
|  | [CY046715](http://www.ncbi.nlm.nih.gov/entrez/viewer.fcgi?val=CY046715) | 1721 | USA | 19-may | 213 | [GQ251037](http://www.ncbi.nlm.nih.gov/entrez/viewer.fcgi?val=GQ251037) | 1439 | Italy | 03-may |
|  | [CY051135](http://www.ncbi.nlm.nih.gov/entrez/viewer.fcgi?val=CY051135) | 1721 | USA | 15-jun |  | [GQ351320](http://www.ncbi.nlm.nih.gov/entrez/viewer.fcgi?val=GQ351320) | 1439 | Italy | 06-jun |
|  | [CY051143](http://www.ncbi.nlm.nih.gov/entrez/viewer.fcgi?val=CY051143) | 1721 | USA | 16-jun | 214 | [GQ323532](http://www.ncbi.nlm.nih.gov/entrez/viewer.fcgi?val=GQ323532) | 1410 | USA | 03-may |
|  | [CY051215](http://www.ncbi.nlm.nih.gov/entrez/viewer.fcgi?val=CY051215) | 1721 | USA | 30-jun | 215 | [CY046245](http://www.ncbi.nlm.nih.gov/entrez/viewer.fcgi?val=CY046245) | 1413 | USA | 03-may |
|  | [CY046523](http://www.ncbi.nlm.nih.gov/entrez/viewer.fcgi?val=CY046523) | 1734 | USA | 04-may | 216 | [CY046285](http://www.ncbi.nlm.nih.gov/entrez/viewer.fcgi?val=CY046285) | 1418 | USA | 03-may |
| 314 | [CY051295](http://www.ncbi.nlm.nih.gov/entrez/viewer.fcgi?val=CY051295) | 1734 | USA | 08-jun | 217 | [CY046349](http://www.ncbi.nlm.nih.gov/entrez/viewer.fcgi?val=CY046349) | 1417 | USA | 03-may |
|  | [CY046859](http://www.ncbi.nlm.nih.gov/entrez/viewer.fcgi?val=CY046859) | 1731 | USA | 04-may | 218 | [GQ323497](http://www.ncbi.nlm.nih.gov/entrez/viewer.fcgi?val=GQ323497) | 1410 | USA | 04-may |
| 315 | [CY046875](http://www.ncbi.nlm.nih.gov/entrez/viewer.fcgi?val=CY046875) | 1726 | USA | 04-may | 219 | [GQ365676](http://www.ncbi.nlm.nih.gov/entrez/viewer.fcgi?val=GQ365676) | 1402 | Germany | 04-may |
| 316 | [CY050142](http://www.ncbi.nlm.nih.gov/entrez/viewer.fcgi?val=CY050142) | 1738 | USA | 04-may | 220 | [GQ377048](http://www.ncbi.nlm.nih.gov/entrez/viewer.fcgi?val=GQ377048) | 1410 | USA | 04-may |
| 317 | [GQ323520](http://www.ncbi.nlm.nih.gov/entrez/viewer.fcgi?val=GQ323520) | 1701 | USA | 05-may | 221 | [GQ402236](http://www.ncbi.nlm.nih.gov/entrez/viewer.fcgi?val=GQ402236) | 1422 | Canada | 04-may |
| 318 | [GQ457501](http://www.ncbi.nlm.nih.gov/entrez/viewer.fcgi?val=GQ457501) | 1701 | USA | 05-may | 222 | [CY045484](http://www.ncbi.nlm.nih.gov/entrez/viewer.fcgi?val=CY045484) | 1437 | Germany | 04-may |
| 319 | [GQ476008](http://www.ncbi.nlm.nih.gov/entrez/viewer.fcgi?val=GQ476008) | 1698 | USA | 05-may | 223 | [CY046445](http://www.ncbi.nlm.nih.gov/entrez/viewer.fcgi?val=CY046445) | 1420 | USA | 04-may |
| 320 | [CY049820](http://www.ncbi.nlm.nih.gov/entrez/viewer.fcgi?val=CY049820) | 1701 | USA | 05-may | 224 | [CY046893](http://www.ncbi.nlm.nih.gov/entrez/viewer.fcgi?val=CY046893) | 1413 | USA | 04-may |
| 321 | [GQ169382](http://www.ncbi.nlm.nih.gov/entrez/viewer.fcgi?val=GQ169382) | 1741 | Thailand | 06-may | 225 | [CY046917](http://www.ncbi.nlm.nih.gov/entrez/viewer.fcgi?val=CY046917) | 1416 | USA | 04-may |
| 322 | [GQ179933](http://www.ncbi.nlm.nih.gov/entrez/viewer.fcgi?val=GQ179933) | 906 | Thailand | 06-may | 226 | [CY050144](http://www.ncbi.nlm.nih.gov/entrez/viewer.fcgi?val=CY050144) | 1418 | USA | 04-may |
| 323 | [CY041750](http://www.ncbi.nlm.nih.gov/entrez/viewer.fcgi?val=CY041750) | 1727 | USA | 06-may | 227 | [CY053144](http://www.ncbi.nlm.nih.gov/entrez/viewer.fcgi?val=CY053144) | 1420 | USA | 04-may |
| 324 | [CY041814](http://www.ncbi.nlm.nih.gov/entrez/viewer.fcgi?val=CY041814) | 1733 | USA | 06-may | 227 | [CY053152](http://www.ncbi.nlm.nih.gov/entrez/viewer.fcgi?val=CY053152) | 1420 | USA | 04-may |
| 325 | [CY041822](http://www.ncbi.nlm.nih.gov/entrez/viewer.fcgi?val=CY041822) | 1733 | USA | 06-may | 228 | [GQ219779](http://www.ncbi.nlm.nih.gov/entrez/viewer.fcgi?val=GQ219779) | 1400 | Poland | 05-may |
| 326 | [GQ329088](http://www.ncbi.nlm.nih.gov/entrez/viewer.fcgi?val=GQ329088) | 1701 | France | 06-may | 229 | [GQ323519](http://www.ncbi.nlm.nih.gov/entrez/viewer.fcgi?val=GQ323519) | 1410 | USA | 05-may |
| 327 | [GQ329106](http://www.ncbi.nlm.nih.gov/entrez/viewer.fcgi?val=GQ329106) | 1701 | France | 06-may | 230 | [GQ323553](http://www.ncbi.nlm.nih.gov/entrez/viewer.fcgi?val=GQ323553) | 1410 | USA | 05-may |
| 328 | [CY043195](http://www.ncbi.nlm.nih.gov/entrez/viewer.fcgi?val=CY043195) | 1727 | USA | 06-may | 231 | [GQ476009](http://www.ncbi.nlm.nih.gov/entrez/viewer.fcgi?val=GQ476009) | 1413 | USA | 05-may |
| 329 | [CY043283](http://www.ncbi.nlm.nih.gov/entrez/viewer.fcgi?val=CY043283) | 1727 | USA | 13-may | 232 | [GQ169381](http://www.ncbi.nlm.nih.gov/entrez/viewer.fcgi?val=GQ169381) | 1419 | Thailand | 06-may |
| 329 | [CY045490](http://www.ncbi.nlm.nih.gov/entrez/viewer.fcgi?val=CY045490) | 1759 | Germany | 06-may | 233 | [GQ179932](http://www.ncbi.nlm.nih.gov/entrez/viewer.fcgi?val=GQ179932) | 1382 | Thailand | 06-may |
| 330 | [CY046555](http://www.ncbi.nlm.nih.gov/entrez/viewer.fcgi?val=CY046555) | 1712 | USA | 06-may | 234 | [CY041816](http://www.ncbi.nlm.nih.gov/entrez/viewer.fcgi?val=CY041816) | 1419 | USA | 06-may |
| 331 | [GQ160611](http://www.ncbi.nlm.nih.gov/entrez/viewer.fcgi?val=GQ160611) | 1687 | Australia | 07-may | 235 | [CY043197](http://www.ncbi.nlm.nih.gov/entrez/viewer.fcgi?val=CY043197) | 1419 | USA | 06-may |
| 332 | [GQ200596](http://www.ncbi.nlm.nih.gov/entrez/viewer.fcgi?val=GQ200596) | 1449 | Mexico | 07-may | 236 | [CY045492](http://www.ncbi.nlm.nih.gov/entrez/viewer.fcgi?val=CY045492) | 1412 | Germany | 06-may |
| 333 | [GQ202695](http://www.ncbi.nlm.nih.gov/entrez/viewer.fcgi?val=GQ202695) | 1679 | Norway | 07-may | 237 | [GQ160610](http://www.ncbi.nlm.nih.gov/entrez/viewer.fcgi?val=GQ160610) | 1396 | Australia | 07-may |
| 334 | [GQ402195](http://www.ncbi.nlm.nih.gov/entrez/viewer.fcgi?val=GQ402195) | 1743 | Canada | 07-may | 238 | [CY040874](http://www.ncbi.nlm.nih.gov/entrez/viewer.fcgi?val=CY040874) | 1417 | Norway | 07-may |
| 335 | [CY046603](http://www.ncbi.nlm.nih.gov/entrez/viewer.fcgi?val=CY046603) | 1730 | USA | 07-may | 239 | [GQ323507](http://www.ncbi.nlm.nih.gov/entrez/viewer.fcgi?val=GQ323507) | 1387 | USA | 07-may |
| 336 | [CY050198](http://www.ncbi.nlm.nih.gov/entrez/viewer.fcgi?val=CY050198) | 1733 | Mexico | 07-may | 240 | [GQ402235](http://www.ncbi.nlm.nih.gov/entrez/viewer.fcgi?val=GQ402235) | 1422 | Canada | 07-may |
| 337 | [CY040880](http://www.ncbi.nlm.nih.gov/entrez/viewer.fcgi?val=CY040880) | 1683 | Norway | 08-may | 241 | [CY046605](http://www.ncbi.nlm.nih.gov/entrez/viewer.fcgi?val=CY046605) | 1420 | USA | 07-may |
| 338 | [GQ227545](http://www.ncbi.nlm.nih.gov/entrez/viewer.fcgi?val=GQ227545) | 1763 | Sweden | 08-may | 242 | [GQ166204](http://www.ncbi.nlm.nih.gov/entrez/viewer.fcgi?val=GQ166204) | 1410 | Japan | 08-may |
| 339 | [CY041766](http://www.ncbi.nlm.nih.gov/entrez/viewer.fcgi?val=CY041766) | 1732 | USA | 08-may | 242 | [GQ166205](http://www.ncbi.nlm.nih.gov/entrez/viewer.fcgi?val=GQ166205) | 1410 | Japan | 08-may |
| 340 | [CY044941](http://www.ncbi.nlm.nih.gov/entrez/viewer.fcgi?val=CY044941) | 1732 | USA | 26-may | 243 | [CY040882](http://www.ncbi.nlm.nih.gov/entrez/viewer.fcgi?val=CY040882) | 1417 | Norway | 08-may |
|  | [GQ396552](http://www.ncbi.nlm.nih.gov/entrez/viewer.fcgi?val=GQ396552) | 949 | Spain | 08-may |  | [CY045039](http://www.ncbi.nlm.nih.gov/entrez/viewer.fcgi?val=CY045039) | 1417 | USA | 09-jun |
| 341 | [GQ402197](http://www.ncbi.nlm.nih.gov/entrez/viewer.fcgi?val=GQ402197) | 1743 | Canada | 08-may | 244 | [GQ227546](http://www.ncbi.nlm.nih.gov/entrez/viewer.fcgi?val=GQ227546) | 1447 | Sweden | 08-may |
| 342 | [CY043123](http://www.ncbi.nlm.nih.gov/entrez/viewer.fcgi?val=CY043123) | 1736 | USA | 08-may | 245 | [GQ323540](http://www.ncbi.nlm.nih.gov/entrez/viewer.fcgi?val=GQ323540) | 1410 | USA | 08-may |
| 343 | [CY046539](http://www.ncbi.nlm.nih.gov/entrez/viewer.fcgi?val=CY046539) | 1734 | USA | 08-may | 246 | [GQ402237](http://www.ncbi.nlm.nih.gov/entrez/viewer.fcgi?val=GQ402237) | 1422 | Canada | 08-may |
| 344 | [CY051647](http://www.ncbi.nlm.nih.gov/entrez/viewer.fcgi?val=CY051647) | 1734 | USA | 30-jun | 247 | [GQ465703](http://www.ncbi.nlm.nih.gov/entrez/viewer.fcgi?val=GQ465703) | 1422 | Canada | 08-may |
|  | [CY046571](http://www.ncbi.nlm.nih.gov/entrez/viewer.fcgi?val=CY046571) | 1720 | USA | 08-may | 248 | [CY046541](http://www.ncbi.nlm.nih.gov/entrez/viewer.fcgi?val=CY046541) | 1419 | USA | 08-may |
| 345 | [CY046587](http://www.ncbi.nlm.nih.gov/entrez/viewer.fcgi?val=CY046587) | 1720 | USA | 08-may | 249 | [CY046589](http://www.ncbi.nlm.nih.gov/entrez/viewer.fcgi?val=CY046589) | 1418 | USA | 08-may |
| 346 | [CY050214](http://www.ncbi.nlm.nih.gov/entrez/viewer.fcgi?val=CY050214) | 1738 | Mexico | 08-may | 250 | [CY050208](http://www.ncbi.nlm.nih.gov/entrez/viewer.fcgi?val=CY050208) | 1420 | Mexico | 08-may |
| 347 | [CY050879](http://www.ncbi.nlm.nih.gov/entrez/viewer.fcgi?val=CY050879) | 1739 | Mexico | 08-may | 251 | [CY050224](http://www.ncbi.nlm.nih.gov/entrez/viewer.fcgi?val=CY050224) | 1414 | Mexico | 08-may |
| 348 | [CY045946](http://www.ncbi.nlm.nih.gov/entrez/viewer.fcgi?val=CY045946) | 1740 | Canada | 09-may | 252 | [CY050881](http://www.ncbi.nlm.nih.gov/entrez/viewer.fcgi?val=CY050881) | 1420 | Mexico | 08-may |
| 349 | [CY046563](http://www.ncbi.nlm.nih.gov/entrez/viewer.fcgi?val=CY046563) | 1721 | USA | 09-may | 253 | [CY045948](http://www.ncbi.nlm.nih.gov/entrez/viewer.fcgi?val=CY045948) | 1435 | Canada | 09-may |
| 350 | [CY046611](http://www.ncbi.nlm.nih.gov/entrez/viewer.fcgi?val=CY046611) | 1726 | USA | 09-may | 254 | [CY046613](http://www.ncbi.nlm.nih.gov/entrez/viewer.fcgi?val=CY046613) | 1411 | USA | 09-may |
| 351 | [CY050174](http://www.ncbi.nlm.nih.gov/entrez/viewer.fcgi?val=CY050174) | 1733 | Mexico | 09-may | 255 | [CY050176](http://www.ncbi.nlm.nih.gov/entrez/viewer.fcgi?val=CY050176) | 1417 | Mexico | 09-may |
| 352 | [CY050230](http://www.ncbi.nlm.nih.gov/entrez/viewer.fcgi?val=CY050230) | 1734 | Mexico | 09-may | 256 | [CY050232](http://www.ncbi.nlm.nih.gov/entrez/viewer.fcgi?val=CY050232) | 1419 | Mexico | 09-may |
| 353 | [CY050246](http://www.ncbi.nlm.nih.gov/entrez/viewer.fcgi?val=CY050246) | 1734 | Mexico | 09-may | 257 | [CY050240](http://www.ncbi.nlm.nih.gov/entrez/viewer.fcgi?val=CY050240) | 1424 | Mexico | 09-may |
| 354 | [CY050254](http://www.ncbi.nlm.nih.gov/entrez/viewer.fcgi?val=CY050254) | 1733 | Mexico | 09-may | 258 | [CY050248](http://www.ncbi.nlm.nih.gov/entrez/viewer.fcgi?val=CY050248) | 1420 | Mexico | 09-may |
| 355 | [CY050772](http://www.ncbi.nlm.nih.gov/entrez/viewer.fcgi?val=CY050772) | 1707 | USA | 09-may | 259 | [CY050256](http://www.ncbi.nlm.nih.gov/entrez/viewer.fcgi?val=CY050256) | 1420 | Mexico | 09-may |
| 356 | [CY050871](http://www.ncbi.nlm.nih.gov/entrez/viewer.fcgi?val=CY050871) | 1733 | Mexico | 09-may | 260 | [CY050774](http://www.ncbi.nlm.nih.gov/entrez/viewer.fcgi?val=CY050774) | 1422 | USA | 09-may |
| 357 | [GQ179930](http://www.ncbi.nlm.nih.gov/entrez/viewer.fcgi?val=GQ179930) | 935 | Thailand | 10-may | 261 | [CY050849](http://www.ncbi.nlm.nih.gov/entrez/viewer.fcgi?val=CY050849) | 1412 | Mexico | 09-may |
| 358 | [GQ166223](http://www.ncbi.nlm.nih.gov/entrez/viewer.fcgi?val=GQ166223) | 1701 | China | 10-may | 262 | [CY050865](http://www.ncbi.nlm.nih.gov/entrez/viewer.fcgi?val=CY050865) | 1420 | Mexico | 09-may |
| 359 | [GQ183633](http://www.ncbi.nlm.nih.gov/entrez/viewer.fcgi?val=GQ183633) | 1726 | Finland | 10-may | 263 | [CY050873](http://www.ncbi.nlm.nih.gov/entrez/viewer.fcgi?val=CY050873) | 1412 | Mexico | 09-may |
| 360 | [GQ200287](http://www.ncbi.nlm.nih.gov/entrez/viewer.fcgi?val=GQ200287) | 1701 | China | 10-may | 264 | [GQ179931](http://www.ncbi.nlm.nih.gov/entrez/viewer.fcgi?val=GQ179931) | 1382 | Thailand | 10-may |
| 361 | [CY050166](http://www.ncbi.nlm.nih.gov/entrez/viewer.fcgi?val=CY050166) | 1733 | Mexico | 10-may | 265 | [GQ166224](http://www.ncbi.nlm.nih.gov/entrez/viewer.fcgi?val=GQ166224) | 1410 | China | 10-may |
| 362 | [CY050887](http://www.ncbi.nlm.nih.gov/entrez/viewer.fcgi?val=CY050887) | 1733 | Mexico | 10-may | 266 | [CY050889](http://www.ncbi.nlm.nih.gov/entrez/viewer.fcgi?val=CY050889) | 1420 | Mexico | 10-may |
| 363 | [CY041758](http://www.ncbi.nlm.nih.gov/entrez/viewer.fcgi?val=CY041758) | 1722 | USA | 11-may | 267 | [GQ323448](http://www.ncbi.nlm.nih.gov/entrez/viewer.fcgi?val=GQ323448) | 1410 | USA | 11-may |
| 364 | [CY043163](http://www.ncbi.nlm.nih.gov/entrez/viewer.fcgi?val=CY043163) | 1734 | USA | 11-may | 268 | [GQ323456](http://www.ncbi.nlm.nih.gov/entrez/viewer.fcgi?val=GQ323456) | 1410 | USA | 11-may |
| 365 | [CY043219](http://www.ncbi.nlm.nih.gov/entrez/viewer.fcgi?val=CY043219) | 1719 | USA | 11-may | 269 | [GQ323569](http://www.ncbi.nlm.nih.gov/entrez/viewer.fcgi?val=GQ323569) | 1410 | USA | 11-may |
| 366 | [GQ323574](http://www.ncbi.nlm.nih.gov/entrez/viewer.fcgi?val=GQ323574) | 1701 | USA | 12-may | 270 | [CY041760](http://www.ncbi.nlm.nih.gov/entrez/viewer.fcgi?val=CY041760) | 1414 | USA | 11-may |
| 367 | [GQ475870](http://www.ncbi.nlm.nih.gov/entrez/viewer.fcgi?val=GQ475870) | 1698 | USA | 12-may | 271 | [CY043165](http://www.ncbi.nlm.nih.gov/entrez/viewer.fcgi?val=CY043165) | 1421 | USA | 11-may |
| 368 | [GQ475927](http://www.ncbi.nlm.nih.gov/entrez/viewer.fcgi?val=GQ475927) | 1698 | USA | 12-may | 272 | [GQ323453](http://www.ncbi.nlm.nih.gov/entrez/viewer.fcgi?val=GQ323453) | 1410 | USA | 12-may |
| 369 | [CY050328](http://www.ncbi.nlm.nih.gov/entrez/viewer.fcgi?val=CY050328) | 1733 | USA | 12-may | 273 | [GQ323575](http://www.ncbi.nlm.nih.gov/entrez/viewer.fcgi?val=GQ323575) | 1410 | USA | 12-may |
| 370 | [CY050855](http://www.ncbi.nlm.nih.gov/entrez/viewer.fcgi?val=CY050855) | 1733 | Mexico | 12-may | 274 | [CY043157](http://www.ncbi.nlm.nih.gov/entrez/viewer.fcgi?val=CY043157) | 1418 | USA | 12-may |
| 371 | [CY046675](http://www.ncbi.nlm.nih.gov/entrez/viewer.fcgi?val=CY046675) | 1721 | USA | 13-may | 275 | [GQ475871](http://www.ncbi.nlm.nih.gov/entrez/viewer.fcgi?val=GQ475871) | 1413 | USA | 12-may |
| 372 | [CY050372](http://www.ncbi.nlm.nih.gov/entrez/viewer.fcgi?val=CY050372) | 1733 | USA | 13-may |  | [GQ475928](http://www.ncbi.nlm.nih.gov/entrez/viewer.fcgi?val=GQ475928) | 1413 | USA | 12-may |
| 373 | [CY050756](http://www.ncbi.nlm.nih.gov/entrez/viewer.fcgi?val=CY050756) | 1721 | USA | 13-may | 276 | [CY050857](http://www.ncbi.nlm.nih.gov/entrez/viewer.fcgi?val=CY050857) | 1414 | Mexico | 12-may |
| 374 | [CY051903](http://www.ncbi.nlm.nih.gov/entrez/viewer.fcgi?val=CY051903) | 1734 | USA | 13-may | 277 | [CY046677](http://www.ncbi.nlm.nih.gov/entrez/viewer.fcgi?val=CY046677) | 1419 | USA | 13-may |
| 375 | [CY053063](http://www.ncbi.nlm.nih.gov/entrez/viewer.fcgi?val=CY053063) | 1734 | USA | 13-may | 278 | [CY050758](http://www.ncbi.nlm.nih.gov/entrez/viewer.fcgi?val=CY050758) | 1404 | USA | 13-may |
|  | [CY052983](http://www.ncbi.nlm.nih.gov/entrez/viewer.fcgi?val=CY052983) | 1734 | USA | 18-may | 279 | [CY051905](http://www.ncbi.nlm.nih.gov/entrez/viewer.fcgi?val=CY051905) | 1420 | USA | 13-may |
|  | [CY051911](http://www.ncbi.nlm.nih.gov/entrez/viewer.fcgi?val=CY051911) | 1734 | USA | 18-may |  | [CY053057](http://www.ncbi.nlm.nih.gov/entrez/viewer.fcgi?val=CY053057) | 1420 | USA | 13-may |
|  | [CY052967](http://www.ncbi.nlm.nih.gov/entrez/viewer.fcgi?val=CY052967) | 1734 | USA | 18-may |  | [CY053065](http://www.ncbi.nlm.nih.gov/entrez/viewer.fcgi?val=CY053065) | 1420 | USA | 13-may |
|  | [CY052991](http://www.ncbi.nlm.nih.gov/entrez/viewer.fcgi?val=CY052991) | 1734 | USA | 18-may |  | [CY051913](http://www.ncbi.nlm.nih.gov/entrez/viewer.fcgi?val=CY051913) | 1420 | USA | 18-may |
|  | [CY052999](http://www.ncbi.nlm.nih.gov/entrez/viewer.fcgi?val=CY052999) | 1734 | USA | 19-may |  | [CY052969](http://www.ncbi.nlm.nih.gov/entrez/viewer.fcgi?val=CY052969) | 1420 | USA | 18-may |
|  | [CY053007](http://www.ncbi.nlm.nih.gov/entrez/viewer.fcgi?val=CY053007) | 1734 | USA | 19-may |  | [CY052985](http://www.ncbi.nlm.nih.gov/entrez/viewer.fcgi?val=CY052985) | 1420 | USA | 18-may |
|  | [CY053015](http://www.ncbi.nlm.nih.gov/entrez/viewer.fcgi?val=CY053015) | 1734 | USA | 20-may |  | [CY052993](http://www.ncbi.nlm.nih.gov/entrez/viewer.fcgi?val=CY052993) | 1420 | USA | 18-may |
|  | [CY053023](http://www.ncbi.nlm.nih.gov/entrez/viewer.fcgi?val=CY053023) | 1734 | USA | 20-may |  | [CY053001](http://www.ncbi.nlm.nih.gov/entrez/viewer.fcgi?val=CY053001) | 1420 | USA | 19-may |
|  | [CY053031](http://www.ncbi.nlm.nih.gov/entrez/viewer.fcgi?val=CY053031) | 1734 | USA | 21-may |  | [CY053009](http://www.ncbi.nlm.nih.gov/entrez/viewer.fcgi?val=CY053009) | 1420 | USA | 19-may |
|  | [CY052975](http://www.ncbi.nlm.nih.gov/entrez/viewer.fcgi?val=CY052975) | 1734 | USA | 21-may |  | [CY053017](http://www.ncbi.nlm.nih.gov/entrez/viewer.fcgi?val=CY053017) | 1420 | USA | 20-may |
|  | [CY051919](http://www.ncbi.nlm.nih.gov/entrez/viewer.fcgi?val=CY051919) | 1734 | USA | 21-may |  | [CY053025](http://www.ncbi.nlm.nih.gov/entrez/viewer.fcgi?val=CY053025) | 1420 | USA | 20-may |
|  | [CY052298](http://www.ncbi.nlm.nih.gov/entrez/viewer.fcgi?val=CY052298) | 1734 | USA | 10-jun |  | [CY053033](http://www.ncbi.nlm.nih.gov/entrez/viewer.fcgi?val=CY053033) | 1420 | USA | 21-may |
|  | [CY052959](http://www.ncbi.nlm.nih.gov/entrez/viewer.fcgi?val=CY052959) | 1734 | USA | 13-may |  | [CY053041](http://www.ncbi.nlm.nih.gov/entrez/viewer.fcgi?val=CY053041) | 1420 | USA | 21-may |
| 376 | [CY053055](http://www.ncbi.nlm.nih.gov/entrez/viewer.fcgi?val=CY053055) | 1724 | USA | 13-may |  | [CY053049](http://www.ncbi.nlm.nih.gov/entrez/viewer.fcgi?val=CY053049) | 1420 | USA | 21-may |
| 377 | [CY053047](http://www.ncbi.nlm.nih.gov/entrez/viewer.fcgi?val=CY053047) | 1724 | USA | 21-may |  | [CY052977](http://www.ncbi.nlm.nih.gov/entrez/viewer.fcgi?val=CY052977) | 1420 | USA | 21-may |
|  | [GQ200598](http://www.ncbi.nlm.nih.gov/entrez/viewer.fcgi?val=GQ200598) | 1062 | Turkey | 14-may |  | [CY051921](http://www.ncbi.nlm.nih.gov/entrez/viewer.fcgi?val=CY051921) | 1420 | USA | 21-may |
| 378 | [CY041790](http://www.ncbi.nlm.nih.gov/entrez/viewer.fcgi?val=CY041790) | 1733 | USA | 14-may |  | [CY052300](http://www.ncbi.nlm.nih.gov/entrez/viewer.fcgi?val=CY052300) | 1420 | USA | 10-jun |
| 379 | [CY043131](http://www.ncbi.nlm.nih.gov/entrez/viewer.fcgi?val=CY043131) | 1732 | USA | 14-may | 280 | [CY052961](http://www.ncbi.nlm.nih.gov/entrez/viewer.fcgi?val=CY052961) | 1420 | USA | 13-may |
| 380 | [GQ476111](http://www.ncbi.nlm.nih.gov/entrez/viewer.fcgi?val=GQ476111) | 1698 | USA | 14-may | 281 | [GQ200599](http://www.ncbi.nlm.nih.gov/entrez/viewer.fcgi?val=GQ200599) | 1146 | Turkey | 14-may |
| 381 | [GQ476114](http://www.ncbi.nlm.nih.gov/entrez/viewer.fcgi?val=GQ476114) | 1698 | USA | 14-may | 282 | [GQ476112](http://www.ncbi.nlm.nih.gov/entrez/viewer.fcgi?val=GQ476112) | 1413 | USA | 14-may |
| 382 | [GQ896363](http://www.ncbi.nlm.nih.gov/entrez/viewer.fcgi?val=GQ896363) | 1698 | USA | 14-may |  | [GQ476115](http://www.ncbi.nlm.nih.gov/entrez/viewer.fcgi?val=GQ476115) | 1413 | USA | 14-may |
| 383 | [CY044973](http://www.ncbi.nlm.nih.gov/entrez/viewer.fcgi?val=CY044973) | 1721 | USA | 15-may |  | [GQ896364](http://www.ncbi.nlm.nih.gov/entrez/viewer.fcgi?val=GQ896364) | 1413 | USA | 14-may |
| 384 | [CY046627](http://www.ncbi.nlm.nih.gov/entrez/viewer.fcgi?val=CY046627) | 1727 | USA | 15-may | 283 | [CY046653](http://www.ncbi.nlm.nih.gov/entrez/viewer.fcgi?val=CY046653) | 1419 | USA | 14-may |
| 385 | [CY048925](http://www.ncbi.nlm.nih.gov/entrez/viewer.fcgi?val=CY048925) | 1734 | Malaysia | 15-may |  | [CY046645](http://www.ncbi.nlm.nih.gov/entrez/viewer.fcgi?val=CY046645) | 1419 | USA | 16-may |
| 386 | [CY050380](http://www.ncbi.nlm.nih.gov/entrez/viewer.fcgi?val=CY050380) | 1725 | USA | 15-may |  | [CY046829](http://www.ncbi.nlm.nih.gov/entrez/viewer.fcgi?val=CY046829) | 1419 | USA | 28-may |
| 387 | [GQ183617](http://www.ncbi.nlm.nih.gov/entrez/viewer.fcgi?val=GQ183617) | 1728 | China | 16-may |  | [CY051273](http://www.ncbi.nlm.nih.gov/entrez/viewer.fcgi?val=CY051273) | 1419 | USA | 02-jun |
| 388 | [GQ183625](http://www.ncbi.nlm.nih.gov/entrez/viewer.fcgi?val=GQ183625) | 1726 | China | 16-may |  | [CY051233](http://www.ncbi.nlm.nih.gov/entrez/viewer.fcgi?val=CY051233) | 1419 | USA | 03-jun |
| 389 | [GQ219578](http://www.ncbi.nlm.nih.gov/entrez/viewer.fcgi?val=GQ219578) | 1701 | Japan | 16-may |  | [CY051289](http://www.ncbi.nlm.nih.gov/entrez/viewer.fcgi?val=CY051289) | 1419 | USA | 04-jun |
| 390 | [GQ219579](http://www.ncbi.nlm.nih.gov/entrez/viewer.fcgi?val=GQ219579) | 1701 | Japan | 16-may |  | [CY051329](http://www.ncbi.nlm.nih.gov/entrez/viewer.fcgi?val=CY051329) | 1419 | USA | 08-jun |
| 391 | [CY041774](http://www.ncbi.nlm.nih.gov/entrez/viewer.fcgi?val=CY041774) | 1733 | USA | 16-may |  | [CY051497](http://www.ncbi.nlm.nih.gov/entrez/viewer.fcgi?val=CY051497) | 1419 | USA | 08-jun |
| 392 | [CY043235](http://www.ncbi.nlm.nih.gov/entrez/viewer.fcgi?val=CY043235) | 1733 | USA | 16-may |  | [CY051505](http://www.ncbi.nlm.nih.gov/entrez/viewer.fcgi?val=CY051505) | 1419 | USA | 08-jun |
| 393 | [CY044981](http://www.ncbi.nlm.nih.gov/entrez/viewer.fcgi?val=CY044981) | 1721 | USA | 16-may |  | [CY051345](http://www.ncbi.nlm.nih.gov/entrez/viewer.fcgi?val=CY051345) | 1419 | USA | 09-jun |
| 394 | [CY046635](http://www.ncbi.nlm.nih.gov/entrez/viewer.fcgi?val=CY046635) | 1728 | USA | 16-may |  | [CY051377](http://www.ncbi.nlm.nih.gov/entrez/viewer.fcgi?val=CY051377) | 1419 | USA | 10-jun |
| 395 | [CY046643](http://www.ncbi.nlm.nih.gov/entrez/viewer.fcgi?val=CY046643) | 1733 | USA | 16-may |  | [CY051001](http://www.ncbi.nlm.nih.gov/entrez/viewer.fcgi?val=CY051001) | 1419 | USA | 13-jun |
| 396 | [CY048933](http://www.ncbi.nlm.nih.gov/entrez/viewer.fcgi?val=CY048933) | 1734 | Malaysia | 16-may |  | [CY051433](http://www.ncbi.nlm.nih.gov/entrez/viewer.fcgi?val=CY051433) | 1419 | USA | 15-jun |
| 397 | [CY051984](http://www.ncbi.nlm.nih.gov/entrez/viewer.fcgi?val=CY051984) | 1744 | Norway | 16-may |  | [CY051457](http://www.ncbi.nlm.nih.gov/entrez/viewer.fcgi?val=CY051457) | 1419 | USA | 18-jun |
| 398 | [GQ200282](http://www.ncbi.nlm.nih.gov/entrez/viewer.fcgi?val=GQ200282) | 1040 | Chile | 17-may |  | [CY051177](http://www.ncbi.nlm.nih.gov/entrez/viewer.fcgi?val=CY051177) | 1419 | USA | 20-jun |
| 399 | [GQ200284](http://www.ncbi.nlm.nih.gov/entrez/viewer.fcgi?val=GQ200284) | 1040 | Chile | 18-may |  | [CY051201](http://www.ncbi.nlm.nih.gov/entrez/viewer.fcgi?val=CY051201) | 1419 | USA | 22-jun |
|  | [GQ200280](http://www.ncbi.nlm.nih.gov/entrez/viewer.fcgi?val=GQ200280) | 1040 | Chile | 18-may |  | [CY051481](http://www.ncbi.nlm.nih.gov/entrez/viewer.fcgi?val=CY051481) | 1419 | USA | 23-jun |
|  | [GQ221694](http://www.ncbi.nlm.nih.gov/entrez/viewer.fcgi?val=GQ221694) | 1701 | China | 17-may |  | [CY051489](http://www.ncbi.nlm.nih.gov/entrez/viewer.fcgi?val=CY051489) | 1419 | USA | 30-jun |
| 400 | [CY041798](http://www.ncbi.nlm.nih.gov/entrez/viewer.fcgi?val=CY041798) | 1731 | USA | 17-may | 284 | [CY046661](http://www.ncbi.nlm.nih.gov/entrez/viewer.fcgi?val=CY046661) | 1421 | USA | 14-may |
| 401 | [GQ329100](http://www.ncbi.nlm.nih.gov/entrez/viewer.fcgi?val=GQ329100) | 1701 | France | 17-may |  | [CY046813](http://www.ncbi.nlm.nih.gov/entrez/viewer.fcgi?val=CY046813) | 1421 | USA | 27-may |
| 402 | [CY045085](http://www.ncbi.nlm.nih.gov/entrez/viewer.fcgi?val=CY045085) | 1734 | USA | 17-may |  | [CY050438](http://www.ncbi.nlm.nih.gov/entrez/viewer.fcgi?val=CY050438) | 1421 | USA | 27-may |
| 403 | [GQ223440](http://www.ncbi.nlm.nih.gov/entrez/viewer.fcgi?val=GQ223440) | 1739 | China | 18-may |  | [CY050945](http://www.ncbi.nlm.nih.gov/entrez/viewer.fcgi?val=CY050945) | 1421 | USA | 01-jun |
| 404 | [GQ268003](http://www.ncbi.nlm.nih.gov/entrez/viewer.fcgi?val=GQ268003) | 1710 | China | 18-may |  | [CY051417](http://www.ncbi.nlm.nih.gov/entrez/viewer.fcgi?val=CY051417) | 1421 | USA | 15-jun |
| 405 | [GQ377069](http://www.ncbi.nlm.nih.gov/entrez/viewer.fcgi?val=GQ377069) | 1701 | USA | 18-may |  | [CY051169](http://www.ncbi.nlm.nih.gov/entrez/viewer.fcgi?val=CY051169) | 1421 | USA | 18-jun |
| 406 | [GQ896360](http://www.ncbi.nlm.nih.gov/entrez/viewer.fcgi?val=GQ896360) | 1698 | USA | 18-may | 285 | [CY046685](http://www.ncbi.nlm.nih.gov/entrez/viewer.fcgi?val=CY046685) | 1419 | USA | 14-may |
| 407 | [GQ896370](http://www.ncbi.nlm.nih.gov/entrez/viewer.fcgi?val=GQ896370) | 1698 | USA | 18-may | 286 | [CY044975](http://www.ncbi.nlm.nih.gov/entrez/viewer.fcgi?val=CY044975) | 1416 | USA | 15-may |
|  | [GQ200286](http://www.ncbi.nlm.nih.gov/entrez/viewer.fcgi?val=GQ200286) | 1040 | Chile | 19-may |  | [CY044959](http://www.ncbi.nlm.nih.gov/entrez/viewer.fcgi?val=CY044959) | 1416 | USA | 24-may |
| 408 | [GQ243751](http://www.ncbi.nlm.nih.gov/entrez/viewer.fcgi?val=GQ243751) | 1687 | Australia | 19-may | 287 | [CY048927](http://www.ncbi.nlm.nih.gov/entrez/viewer.fcgi?val=CY048927) | 1413 | Malaysia | 15-may |
| 409 | [GQ243757](http://www.ncbi.nlm.nih.gov/entrez/viewer.fcgi?val=GQ243757) | 1687 | Australia | 29-may | 288 | [GQ183619](http://www.ncbi.nlm.nih.gov/entrez/viewer.fcgi?val=GQ183619) | 1416 | China | 16-may |
|  | [GQ323486](http://www.ncbi.nlm.nih.gov/entrez/viewer.fcgi?val=GQ323486) | 1701 | USA | 19-may |  | [GQ183627](http://www.ncbi.nlm.nih.gov/entrez/viewer.fcgi?val=GQ183627) | 1416 | China | 16-may |
| 410 | [GQ323489](http://www.ncbi.nlm.nih.gov/entrez/viewer.fcgi?val=GQ323489) | 1701 | USA | 19-may | 289 | [GQ220734](http://www.ncbi.nlm.nih.gov/entrez/viewer.fcgi?val=GQ220734) | 1410 | Japan | 16-may |
|  | [GQ396555](http://www.ncbi.nlm.nih.gov/entrez/viewer.fcgi?val=GQ396555) | 948 | Spain | 19-may |  | [GQ220735](http://www.ncbi.nlm.nih.gov/entrez/viewer.fcgi?val=GQ220735) | 1410 | Japan | 16-may |
| 411 | [CY044064](http://www.ncbi.nlm.nih.gov/entrez/viewer.fcgi?val=CY044064) | 1734 | USA | 19-may |  | [GQ220736](http://www.ncbi.nlm.nih.gov/entrez/viewer.fcgi?val=GQ220736) | 1410 | Japan | 16-may |
| 412 | [CY044196](http://www.ncbi.nlm.nih.gov/entrez/viewer.fcgi?val=CY044196) | 1701 | Taiwan | 19-may |  | [GQ220732](http://www.ncbi.nlm.nih.gov/entrez/viewer.fcgi?val=GQ220732) | 1410 | Japan | 17-may |
| 413 | [CY044917](http://www.ncbi.nlm.nih.gov/entrez/viewer.fcgi?val=CY044917) | 1733 | USA | 19-may |  | [GQ220737](http://www.ncbi.nlm.nih.gov/entrez/viewer.fcgi?val=GQ220737) | 1410 | Japan | 17-may |
| 414 | [CY045143](http://www.ncbi.nlm.nih.gov/entrez/viewer.fcgi?val=CY045143) | 1734 | USA | 19-may |  | [GQ261273](http://www.ncbi.nlm.nih.gov/entrez/viewer.fcgi?val=GQ261273) | 1410 | Japan | 18-may |
| 415 | [CY047358](http://www.ncbi.nlm.nih.gov/entrez/viewer.fcgi?val=CY047358) | 1718 | USA | 19-may |  | [GQ287622](http://www.ncbi.nlm.nih.gov/entrez/viewer.fcgi?val=GQ287622) | 1410 | Japan | 19-may |
| 416 | [CY050412](http://www.ncbi.nlm.nih.gov/entrez/viewer.fcgi?val=CY050412) | 1726 | USA | 19-may |  | [AB514228](http://www.ncbi.nlm.nih.gov/entrez/viewer.fcgi?val=AB514228) | 1410 | Japan | 21-may |
| 417 | [GQ225381](http://www.ncbi.nlm.nih.gov/entrez/viewer.fcgi?val=GQ225381) | 1701 | China | 20-may |  | [GQ261274](http://www.ncbi.nlm.nih.gov/entrez/viewer.fcgi?val=GQ261274) | 1410 | Japan | 21-may |
| 418 | [GQ290106](http://www.ncbi.nlm.nih.gov/entrez/viewer.fcgi?val=GQ290106) | 1701 | China | 20-may |  | [GQ261276](http://www.ncbi.nlm.nih.gov/entrez/viewer.fcgi?val=GQ261276) | 1410 | Japan | 21-may |
|  | [GQ243753](http://www.ncbi.nlm.nih.gov/entrez/viewer.fcgi?val=GQ243753) | 1687 | Philippines | 20-may |  | [GQ504753](http://www.ncbi.nlm.nih.gov/entrez/viewer.fcgi?val=GQ504753) | 1410 | China | 18-jun |
| 419 | [GQ246478](http://www.ncbi.nlm.nih.gov/entrez/viewer.fcgi?val=GQ246478) | 916 | Italy | 20-may | 290 | [CY043237](http://www.ncbi.nlm.nih.gov/entrez/viewer.fcgi?val=CY043237) | 1420 | USA | 16-may |
| 420 | [GQ402200](http://www.ncbi.nlm.nih.gov/entrez/viewer.fcgi?val=GQ402200) | 1743 | Canada | 20-may | 291 | [CY043293](http://www.ncbi.nlm.nih.gov/entrez/viewer.fcgi?val=CY043293) | 1420 | USA | 16-may |
| 421 | [GQ414764](http://www.ncbi.nlm.nih.gov/entrez/viewer.fcgi?val=GQ414764) | 1701 | Brazil | 20-may | 292 | [CY044983](http://www.ncbi.nlm.nih.gov/entrez/viewer.fcgi?val=CY044983) | 1415 | USA | 16-may |
| 422 | [CY044048](http://www.ncbi.nlm.nih.gov/entrez/viewer.fcgi?val=CY044048) | 1734 | USA | 20-may | 293 | [CY046621](http://www.ncbi.nlm.nih.gov/entrez/viewer.fcgi?val=CY046621) | 1417 | USA | 16-may |
| 423 | [CY044056](http://www.ncbi.nlm.nih.gov/entrez/viewer.fcgi?val=CY044056) | 1733 | USA | 20-may | 294 | [CY046637](http://www.ncbi.nlm.nih.gov/entrez/viewer.fcgi?val=CY046637) | 1412 | USA | 16-may |
| 424 | [CY044933](http://www.ncbi.nlm.nih.gov/entrez/viewer.fcgi?val=CY044933) | 1733 | USA | 20-may |  | [CY046901](http://www.ncbi.nlm.nih.gov/entrez/viewer.fcgi?val=CY046901) | 1412 | USA | 28-may |
| 425 | [CY046707](http://www.ncbi.nlm.nih.gov/entrez/viewer.fcgi?val=CY046707) | 1724 | USA | 20-may | 295 | [CY048935](http://www.ncbi.nlm.nih.gov/entrez/viewer.fcgi?val=CY048935) | 1413 | Malaysia | 16-may |
| 426 | [CY046731](http://www.ncbi.nlm.nih.gov/entrez/viewer.fcgi?val=CY046731) | 1720 | USA | 20-may | 296 | [GQ200281](http://www.ncbi.nlm.nih.gov/entrez/viewer.fcgi?val=GQ200281) | 1293 | Chile | 17-may |
| 427 | [CY053174](http://www.ncbi.nlm.nih.gov/entrez/viewer.fcgi?val=CY053174) | 1734 | USA | 20-may |  | [GQ200283](http://www.ncbi.nlm.nih.gov/entrez/viewer.fcgi?val=GQ200283) | 1293 | Chile | 18-may |
| 428 | [CY053182](http://www.ncbi.nlm.nih.gov/entrez/viewer.fcgi?val=CY053182) | 1734 | USA | 20-may |  | [GQ200279](http://www.ncbi.nlm.nih.gov/entrez/viewer.fcgi?val=GQ200279) | 1293 | Chile | 18-may |
| 429 | [CY053135](http://www.ncbi.nlm.nih.gov/entrez/viewer.fcgi?val=CY053135) | 1734 | USA | 23-may | 297 | [GQ220731](http://www.ncbi.nlm.nih.gov/entrez/viewer.fcgi?val=GQ220731) | 1410 | Japan | 17-may |
| 430 | [CY053127](http://www.ncbi.nlm.nih.gov/entrez/viewer.fcgi?val=CY053127) | 1734 | USA | 23-may | 298 | [CY041800](http://www.ncbi.nlm.nih.gov/entrez/viewer.fcgi?val=CY041800) | 1414 | USA | 17-may |
|  | [GQ219586](http://www.ncbi.nlm.nih.gov/entrez/viewer.fcgi?val=GQ219586) | 1752 | Russia | 21-may | 299 | [GQ402239](http://www.ncbi.nlm.nih.gov/entrez/viewer.fcgi?val=GQ402239) | 1422 | Canada | 17-may |
| 431 | [GQ494354](http://www.ncbi.nlm.nih.gov/entrez/viewer.fcgi?val=GQ494354) | 1752 | Russia | 20-jun | 300 | [CY043229](http://www.ncbi.nlm.nih.gov/entrez/viewer.fcgi?val=CY043229) | 1418 | USA | 17-may |
|  | [GQ392022](http://www.ncbi.nlm.nih.gov/entrez/viewer.fcgi?val=GQ392022) | 1752 | Russia | 09-jul |  | [CY045177](http://www.ncbi.nlm.nih.gov/entrez/viewer.fcgi?val=CY045177) | 1418 | USA | 01-jun |
|  | [GU433033](http://www.ncbi.nlm.nih.gov/entrez/viewer.fcgi?val=GU433033) | 1752 | Russia | 21-jul | 301 | [CY045087](http://www.ncbi.nlm.nih.gov/entrez/viewer.fcgi?val=CY045087) | 1414 | USA | 17-may |
|  | [GU211227](http://www.ncbi.nlm.nih.gov/entrez/viewer.fcgi?val=GU211227) | 1752 | Russia | 22-sep | 302 | [GQ223445](http://www.ncbi.nlm.nih.gov/entrez/viewer.fcgi?val=GQ223445) | 1425 | China | 18-may |
|  | [GQ223408](http://www.ncbi.nlm.nih.gov/entrez/viewer.fcgi?val=GQ223408) | 1701 | China | 21-may | 303 | [CY044090](http://www.ncbi.nlm.nih.gov/entrez/viewer.fcgi?val=CY044090) | 1417 | USA | 18-may |
| 432 | [GQ232099](http://www.ncbi.nlm.nih.gov/entrez/viewer.fcgi?val=GQ232099) | 1701 | Italy | 21-may |  | [CY045185](http://www.ncbi.nlm.nih.gov/entrez/viewer.fcgi?val=CY045185) | 1417 | USA | 31-may |
| 433 | [GQ243761](http://www.ncbi.nlm.nih.gov/entrez/viewer.fcgi?val=GQ243761) | 1687 | Australia | 21-may |  | [CY044098](http://www.ncbi.nlm.nih.gov/entrez/viewer.fcgi?val=CY044098) | 1417 | USA | 03-jun |
| 434 | [GQ243759](http://www.ncbi.nlm.nih.gov/entrez/viewer.fcgi?val=GQ243759) | 1687 | Philippines | 25-may | 304 | [CY044911](http://www.ncbi.nlm.nih.gov/entrez/viewer.fcgi?val=CY044911) | 1416 | USA | 18-may |
|  | [GQ261275](http://www.ncbi.nlm.nih.gov/entrez/viewer.fcgi?val=GQ261275) | 1701 | Japan | 21-may |  | [CY044066](http://www.ncbi.nlm.nih.gov/entrez/viewer.fcgi?val=CY044066) | 1416 | USA | 19-may |
| 435 | [GQ267839](http://www.ncbi.nlm.nih.gov/entrez/viewer.fcgi?val=GQ267839) | 1701 | Japan | 21-may | 305 | [GQ896361](http://www.ncbi.nlm.nih.gov/entrez/viewer.fcgi?val=GQ896361) | 1413 | USA | 18-may |
| 436 | [GQ402203](http://www.ncbi.nlm.nih.gov/entrez/viewer.fcgi?val=GQ402203) | 1743 | Canada | 21-may | 306 | [GQ896371](http://www.ncbi.nlm.nih.gov/entrez/viewer.fcgi?val=GQ896371) | 1413 | USA | 18-may |
| 437 | [CY044877](http://www.ncbi.nlm.nih.gov/entrez/viewer.fcgi?val=CY044877) | 1734 | USA | 21-may | 307 | [GQ200285](http://www.ncbi.nlm.nih.gov/entrez/viewer.fcgi?val=GQ200285) | 1293 | Chile | 19-may |
| 438 | [CY046963](http://www.ncbi.nlm.nih.gov/entrez/viewer.fcgi?val=CY046963) | 1734 | USA | 21-may | 308 | [GQ243752](http://www.ncbi.nlm.nih.gov/entrez/viewer.fcgi?val=GQ243752) | 1403 | Australia | 19-may |
|  | [CY044965](http://www.ncbi.nlm.nih.gov/entrez/viewer.fcgi?val=CY044965) | 1720 | USA | 21-may | 309 | [GQ323488](http://www.ncbi.nlm.nih.gov/entrez/viewer.fcgi?val=GQ323488) | 1410 | USA | 19-may |
| 439 | [CY053039](http://www.ncbi.nlm.nih.gov/entrez/viewer.fcgi?val=CY053039) | 1734 | USA | 21-may | 310 | [CY045145](http://www.ncbi.nlm.nih.gov/entrez/viewer.fcgi?val=CY045145) | 1414 | USA | 19-may |
| 440 | [CY053190](http://www.ncbi.nlm.nih.gov/entrez/viewer.fcgi?val=CY053190) | 1734 | USA | 21-may | 311 | [CY046741](http://www.ncbi.nlm.nih.gov/entrez/viewer.fcgi?val=CY046741) | 1420 | USA | 19-may |
| 441 | [CY053198](http://www.ncbi.nlm.nih.gov/entrez/viewer.fcgi?val=CY053198) | 1734 | USA | 21-may | 312 | [CY047360](http://www.ncbi.nlm.nih.gov/entrez/viewer.fcgi?val=CY047360) | 1418 | USA | 19-may |
|  | [GQ225365](http://www.ncbi.nlm.nih.gov/entrez/viewer.fcgi?val=GQ225365) | 1701 | China | 22-may | 313 | [GQ243754](http://www.ncbi.nlm.nih.gov/entrez/viewer.fcgi?val=GQ243754) | 1397 | Philippines | 20-may |
| 442 | [GQ255897](http://www.ncbi.nlm.nih.gov/entrez/viewer.fcgi?val=GQ255897) | 1701 | Russia | 22-may | 314 | [CY043149](http://www.ncbi.nlm.nih.gov/entrez/viewer.fcgi?val=CY043149) | 1413 | USA | 20-may |
| 443 | [CY041960](http://www.ncbi.nlm.nih.gov/entrez/viewer.fcgi?val=CY041960) | 1701 | Dominican Republic | 24-may | 315 | [CY044058](http://www.ncbi.nlm.nih.gov/entrez/viewer.fcgi?val=CY044058) | 1417 | USA | 20-may |
|  | [CY041983](http://www.ncbi.nlm.nih.gov/entrez/viewer.fcgi?val=CY041983) | 1701 | Dominican Republic | 24-may | 316 | [CY044895](http://www.ncbi.nlm.nih.gov/entrez/viewer.fcgi?val=CY044895) | 1411 | USA | 20-may |
|  | [CY043102](http://www.ncbi.nlm.nih.gov/entrez/viewer.fcgi?val=CY043102) | 1701 | Dominican Republic | 26-may | 317 | [CY046709](http://www.ncbi.nlm.nih.gov/entrez/viewer.fcgi?val=CY046709) | 1416 | USA | 20-may |
|  | [CY043110](http://www.ncbi.nlm.nih.gov/entrez/viewer.fcgi?val=CY043110) | 1701 | Dominican Republic | 26-may | 318 | [CY046733](http://www.ncbi.nlm.nih.gov/entrez/viewer.fcgi?val=CY046733) | 1414 | USA | 20-may |
|  | [GQ329093](http://www.ncbi.nlm.nih.gov/entrez/viewer.fcgi?val=GQ329093) | 1701 | France | 02-jun | 319 | [CY053176](http://www.ncbi.nlm.nih.gov/entrez/viewer.fcgi?val=CY053176) | 1421 | USA | 20-may |
|  | [CY049971](http://www.ncbi.nlm.nih.gov/entrez/viewer.fcgi?val=CY049971) | 1701 | Dominican Republic | 17-jun |  | [CY053192](http://www.ncbi.nlm.nih.gov/entrez/viewer.fcgi?val=CY053192) | 1421 | USA | 21-may |
|  | [GQ261277](http://www.ncbi.nlm.nih.gov/entrez/viewer.fcgi?val=GQ261277) | 1701 | Japan | 22-may |  | [CY053200](http://www.ncbi.nlm.nih.gov/entrez/viewer.fcgi?val=CY053200) | 1421 | USA | 21-may |
| 444 | [GQ329082](http://www.ncbi.nlm.nih.gov/entrez/viewer.fcgi?val=GQ329082) | 1701 | France | 22-may |  | [CY053271](http://www.ncbi.nlm.nih.gov/entrez/viewer.fcgi?val=CY053271) | 1421 | USA | 30-may |
| 445 | [GQ402202](http://www.ncbi.nlm.nih.gov/entrez/viewer.fcgi?val=GQ402202) | 1743 | Canada | 22-may | 320 | [CY053184](http://www.ncbi.nlm.nih.gov/entrez/viewer.fcgi?val=CY053184) | 1420 | USA | 20-may |
| 446 | [GQ402187](http://www.ncbi.nlm.nih.gov/entrez/viewer.fcgi?val=GQ402187) | 1743 | Canada | 24-may | 321 | [GQ219585](http://www.ncbi.nlm.nih.gov/entrez/viewer.fcgi?val=GQ219585) | 1433 | Russia | 21-may |
|  | [GQ465672](http://www.ncbi.nlm.nih.gov/entrez/viewer.fcgi?val=GQ465672) | 1743 | Canada | 01-jul | 322 | [GQ243762](http://www.ncbi.nlm.nih.gov/entrez/viewer.fcgi?val=GQ243762) | 1396 | Australia | 21-may |
|  | [CY044212](http://www.ncbi.nlm.nih.gov/entrez/viewer.fcgi?val=CY044212) | 1704 | Taiwan | 22-may | 323 | [GQ184628](http://www.ncbi.nlm.nih.gov/entrez/viewer.fcgi?val=GQ184628) | 1410 | Russia | 21-may |
| 447 | [CY046747](http://www.ncbi.nlm.nih.gov/entrez/viewer.fcgi?val=CY046747) | 1721 | USA | 22-may | 324 | [CY044903](http://www.ncbi.nlm.nih.gov/entrez/viewer.fcgi?val=CY044903) | 1420 | USA | 21-may |
| 448 | [CY046763](http://www.ncbi.nlm.nih.gov/entrez/viewer.fcgi?val=CY046763) | 1721 | USA | 22-may | 325 | [CY044967](http://www.ncbi.nlm.nih.gov/entrez/viewer.fcgi?val=CY044967) | 1419 | USA | 21-may |
| 449 | [GQ896366](http://www.ncbi.nlm.nih.gov/entrez/viewer.fcgi?val=GQ896366) | 1698 | USA | 22-may |  | [CY045095](http://www.ncbi.nlm.nih.gov/entrez/viewer.fcgi?val=CY045095) | 1419 | USA | 22-may |
| 450 | [GQ896376](http://www.ncbi.nlm.nih.gov/entrez/viewer.fcgi?val=GQ896376) | 1698 | USA | 22-may |  | [CY045193](http://www.ncbi.nlm.nih.gov/entrez/viewer.fcgi?val=CY045193) | 1419 | USA | 10-jun |
| 451 | [CY053206](http://www.ncbi.nlm.nih.gov/entrez/viewer.fcgi?val=CY053206) | 1734 | USA | 22-may | 326 | [GQ255898](http://www.ncbi.nlm.nih.gov/entrez/viewer.fcgi?val=GQ255898) | 1410 | Russia | 22-may |
| 452 | [CY053214](http://www.ncbi.nlm.nih.gov/entrez/viewer.fcgi?val=CY053214) | 1734 | USA | 22-may | 327 | [GQ261278](http://www.ncbi.nlm.nih.gov/entrez/viewer.fcgi?val=GQ261278) | 1410 | Japan | 22-may |
|  | [GQ225349](http://www.ncbi.nlm.nih.gov/entrez/viewer.fcgi?val=GQ225349) | 1701 | China | 23-may | 328 | [GQ402242](http://www.ncbi.nlm.nih.gov/entrez/viewer.fcgi?val=GQ402242) | 1422 | Canada | 22-may |
| 453 | [GQ232093](http://www.ncbi.nlm.nih.gov/entrez/viewer.fcgi?val=GQ232093) | 1701 | China | 23-may | 329 | [GQ896367](http://www.ncbi.nlm.nih.gov/entrez/viewer.fcgi?val=GQ896367) | 1413 | USA | 22-may |
| 454 | [GQ243749](http://www.ncbi.nlm.nih.gov/entrez/viewer.fcgi?val=GQ243749) | 1687 | Philippines | 23-may | 330 | [GQ896377](http://www.ncbi.nlm.nih.gov/entrez/viewer.fcgi?val=GQ896377) | 1413 | USA | 22-may |
| 455 | [GQ329066](http://www.ncbi.nlm.nih.gov/entrez/viewer.fcgi?val=GQ329066) | 1702 | France | 23-may | 331 | [CY053208](http://www.ncbi.nlm.nih.gov/entrez/viewer.fcgi?val=CY053208) | 1420 | USA | 22-may |
| 456 | [GQ338335](http://www.ncbi.nlm.nih.gov/entrez/viewer.fcgi?val=GQ338335) | 1701 | USA | 23-may |  | [CY053216](http://www.ncbi.nlm.nih.gov/entrez/viewer.fcgi?val=CY053216) | 1420 | USA | 22-may |
| 457 | [CY049907](http://www.ncbi.nlm.nih.gov/entrez/viewer.fcgi?val=CY049907) | 1698 | Dominican Republic | 23-may | 332 | [GQ225351](http://www.ncbi.nlm.nih.gov/entrez/viewer.fcgi?val=GQ225351) | 1410 | China | 23-may |
| 458 | [CY049915](http://www.ncbi.nlm.nih.gov/entrez/viewer.fcgi?val=CY049915) | 1707 | Dominican Republic | 23-may |  | [GQ334348](http://www.ncbi.nlm.nih.gov/entrez/viewer.fcgi?val=GQ334348) | 1410 | Japan | 02-jun |
| 459 | [CY050420](http://www.ncbi.nlm.nih.gov/entrez/viewer.fcgi?val=CY050420) | 1733 | USA | 23-may |  | [GU014777](http://www.ncbi.nlm.nih.gov/entrez/viewer.fcgi?val=GU014777) | 1410 | Japan | 02-jun |
| 460 | [CY053222](http://www.ncbi.nlm.nih.gov/entrez/viewer.fcgi?val=CY053222) | 1734 | USA | 23-may |  | [GU014809](http://www.ncbi.nlm.nih.gov/entrez/viewer.fcgi?val=GU014809) | 1410 | Japan | 03-jun |
| 461 | [CY051239](http://www.ncbi.nlm.nih.gov/entrez/viewer.fcgi?val=CY051239) | 1734 | USA | 09-jun |  | [GQ368668](http://www.ncbi.nlm.nih.gov/entrez/viewer.fcgi?val=GQ368668) | 1410 | Brazil | 05-jun |
|  | [GQ323579](http://www.ncbi.nlm.nih.gov/entrez/viewer.fcgi?val=GQ323579) | 1701 | USA | 24-may |  | [GQ287624](http://www.ncbi.nlm.nih.gov/entrez/viewer.fcgi?val=GQ287624) | 1410 | Japan | 06-jun |
| 462 | [CY044957](http://www.ncbi.nlm.nih.gov/entrez/viewer.fcgi?val=CY044957) | 1721 | USA | 24-may |  | [GQ287628](http://www.ncbi.nlm.nih.gov/entrez/viewer.fcgi?val=GQ287628) | 1410 | Japan | 09-jun |
| 463 | [CY045938](http://www.ncbi.nlm.nih.gov/entrez/viewer.fcgi?val=CY045938) | 1741 | Canada | 24-may |  | [GQ365427](http://www.ncbi.nlm.nih.gov/entrez/viewer.fcgi?val=GQ365427) | 1410 | Japan | 09-jun |
| 464 | [CY046699](http://www.ncbi.nlm.nih.gov/entrez/viewer.fcgi?val=CY046699) | 1726 | USA | 24-may |  | [GQ365412](http://www.ncbi.nlm.nih.gov/entrez/viewer.fcgi?val=GQ365412) | 1410 | Japan | 11-jun |
| 465 | [CY052047](http://www.ncbi.nlm.nih.gov/entrez/viewer.fcgi?val=CY052047) | 1269 | Brazil | 24-may |  | [GU014793](http://www.ncbi.nlm.nih.gov/entrez/viewer.fcgi?val=GU014793) | 1410 | Japan | 12-jun |
| 466 | [GQ243760](http://www.ncbi.nlm.nih.gov/entrez/viewer.fcgi?val=GQ243760) | 1687 | Philippines | 25-may | 333 | [GQ243750](http://www.ncbi.nlm.nih.gov/entrez/viewer.fcgi?val=GQ243750) | 1396 | Philippines | 23-may |
| 467 | [GQ293441](http://www.ncbi.nlm.nih.gov/entrez/viewer.fcgi?val=GQ293441) | 1546 | Spain | 25-may |  | [GQ243758](http://www.ncbi.nlm.nih.gov/entrez/viewer.fcgi?val=GQ243758) | 1396 | Australia | 29-may |
| 468 | [CY043275](http://www.ncbi.nlm.nih.gov/entrez/viewer.fcgi?val=CY043275) | 1733 | USA | 25-may | 334 | [CY049909](http://www.ncbi.nlm.nih.gov/entrez/viewer.fcgi?val=CY049909) | 1410 | Dominican Republic | 23-may |
| 469 | [CY044925](http://www.ncbi.nlm.nih.gov/entrez/viewer.fcgi?val=CY044925) | 1733 | USA | 25-may |  | [CY049917](http://www.ncbi.nlm.nih.gov/entrez/viewer.fcgi?val=CY049917) | 1410 | Dominican Republic | 23-may |
| 470 | [GQ243755](http://www.ncbi.nlm.nih.gov/entrez/viewer.fcgi?val=GQ243755) | 1687 | Australia | 26-may | 335 | [CY050422](http://www.ncbi.nlm.nih.gov/entrez/viewer.fcgi?val=CY050422) | 1420 | USA | 23-may |
| 471 | [GQ247726](http://www.ncbi.nlm.nih.gov/entrez/viewer.fcgi?val=GQ247726) | 1752 | Russia | 26-may | 336 | [CY053129](http://www.ncbi.nlm.nih.gov/entrez/viewer.fcgi?val=CY053129) | 1420 | USA | 23-may |
| 472 | [GQ375284](http://www.ncbi.nlm.nih.gov/entrez/viewer.fcgi?val=GQ375284) | 1752 | Russia | 26-may |  | [CY053137](http://www.ncbi.nlm.nih.gov/entrez/viewer.fcgi?val=CY053137) | 1420 | USA | 23-may |
|  | [GQ255900](http://www.ncbi.nlm.nih.gov/entrez/viewer.fcgi?val=GQ255900) | 1701 | Russia | 26-may | 337 | [CY053224](http://www.ncbi.nlm.nih.gov/entrez/viewer.fcgi?val=CY053224) | 1421 | USA | 23-may |
| 473 | [CY044228](http://www.ncbi.nlm.nih.gov/entrez/viewer.fcgi?val=CY044228) | 1701 | Taiwan | 30-may |  | [CY053232](http://www.ncbi.nlm.nih.gov/entrez/viewer.fcgi?val=CY053232) | 1421 | USA | 23-may |
|  | [GQ283488](http://www.ncbi.nlm.nih.gov/entrez/viewer.fcgi?val=GQ283488) | 1743 | Finland | 26-may |  | [CY053256](http://www.ncbi.nlm.nih.gov/entrez/viewer.fcgi?val=CY053256) | 1421 | USA | 27-may |
| 474 | [GQ385300](http://www.ncbi.nlm.nih.gov/entrez/viewer.fcgi?val=GQ385300) | 1753 | Canada | 26-may |  | [CY053311](http://www.ncbi.nlm.nih.gov/entrez/viewer.fcgi?val=CY053311) | 1421 | USA | 28-may |
| 475 | [CY044072](http://www.ncbi.nlm.nih.gov/entrez/viewer.fcgi?val=CY044072) | 1734 | USA | 26-may |  | [CY053263](http://www.ncbi.nlm.nih.gov/entrez/viewer.fcgi?val=CY053263) | 1421 | USA | 28-may |
| 476 | [CY044997](http://www.ncbi.nlm.nih.gov/entrez/viewer.fcgi?val=CY044997) | 1734 | USA | 02-jun | 338 | [CY041985](http://www.ncbi.nlm.nih.gov/entrez/viewer.fcgi?val=CY041985) | 1410 | Dominican Republic | 24-may |
|  | [CY052615](http://www.ncbi.nlm.nih.gov/entrez/viewer.fcgi?val=CY052615) | 1734 | USA | 30-Aug |  | [CY043104](http://www.ncbi.nlm.nih.gov/entrez/viewer.fcgi?val=CY043104) | 1410 | Dominican Republic | 26-may |
|  | [CY051783](http://www.ncbi.nlm.nih.gov/entrez/viewer.fcgi?val=CY051783) | 1734 | USA | 06-sep |  | [CY043112](http://www.ncbi.nlm.nih.gov/entrez/viewer.fcgi?val=CY043112) | 1410 | Dominican Republic | 26-may |
|  | [CY052695](http://www.ncbi.nlm.nih.gov/entrez/viewer.fcgi?val=CY052695) | 1734 | USA | 08-sep |  | [CY049941](http://www.ncbi.nlm.nih.gov/entrez/viewer.fcgi?val=CY049941) | 1410 | Dominican Republic | 25-jun |
|  | [CY052503](http://www.ncbi.nlm.nih.gov/entrez/viewer.fcgi?val=CY052503) | 1734 | USA | 10-sep | 339 | [GQ402227](http://www.ncbi.nlm.nih.gov/entrez/viewer.fcgi?val=GQ402227) | 1422 | Canada | 24-may |
|  | [CY045151](http://www.ncbi.nlm.nih.gov/entrez/viewer.fcgi?val=CY045151) | 1721 | USA | 26-may | 340 | [GQ402241](http://www.ncbi.nlm.nih.gov/entrez/viewer.fcgi?val=GQ402241) | 1422 | Canada | 24-may |
| 477 | [CY046779](http://www.ncbi.nlm.nih.gov/entrez/viewer.fcgi?val=CY046779) | 1721 | USA | 26-may |  | [GQ402245](http://www.ncbi.nlm.nih.gov/entrez/viewer.fcgi?val=GQ402245) | 1422 | Canada | 29-may |
| 478 | [CY046955](http://www.ncbi.nlm.nih.gov/entrez/viewer.fcgi?val=CY046955) | 1716 | USA | 26-may | 341 | [CY045940](http://www.ncbi.nlm.nih.gov/entrez/viewer.fcgi?val=CY045940) | 1431 | Canada | 24-may |
| 479 | [GU014804](http://www.ncbi.nlm.nih.gov/entrez/viewer.fcgi?val=GU014804) | 1701 | Japan | 26-may | 342 | [GQ214167](http://www.ncbi.nlm.nih.gov/entrez/viewer.fcgi?val=GQ214167) | 838 | Ireland | 25-may |
| 480 | [CY050748](http://www.ncbi.nlm.nih.gov/entrez/viewer.fcgi?val=CY050748) | 1727 | USA | 26-may | 343 | [GQ303551](http://www.ncbi.nlm.nih.gov/entrez/viewer.fcgi?val=GQ303551) | 1226 | Spain | 25-may |
| 481 | [CY051247](http://www.ncbi.nlm.nih.gov/entrez/viewer.fcgi?val=CY051247) | 1734 | USA | 26-may | 344 | [CY043277](http://www.ncbi.nlm.nih.gov/entrez/viewer.fcgi?val=CY043277) | 1421 | USA | 25-may |
| 482 | [GQ232085](http://www.ncbi.nlm.nih.gov/entrez/viewer.fcgi?val=GQ232085) | 1701 | China | 27-may | 345 | [CY044927](http://www.ncbi.nlm.nih.gov/entrez/viewer.fcgi?val=CY044927) | 1413 | USA | 25-may |
| 483 | [GQ223435](http://www.ncbi.nlm.nih.gov/entrez/viewer.fcgi?val=GQ223435) | 1701 | China | 29-may | 346 | [CY050430](http://www.ncbi.nlm.nih.gov/entrez/viewer.fcgi?val=CY050430) | 1420 | USA | 25-may |
|  | [GQ377095](http://www.ncbi.nlm.nih.gov/entrez/viewer.fcgi?val=GQ377095) | 1701 | USA | 29-may | 347 | [GQ243756](http://www.ncbi.nlm.nih.gov/entrez/viewer.fcgi?val=GQ243756) | 1396 | Australia | 26-may |
|  | [CY046062](http://www.ncbi.nlm.nih.gov/entrez/viewer.fcgi?val=CY046062) | 1701 | Italy | 16-jun | 348 | [GQ247727](http://www.ncbi.nlm.nih.gov/entrez/viewer.fcgi?val=GQ247727) | 1434 | Russia | 26-may |
|  | [CY046787](http://www.ncbi.nlm.nih.gov/entrez/viewer.fcgi?val=CY046787) | 1734 | USA | 27-may |  | [GQ375286](http://www.ncbi.nlm.nih.gov/entrez/viewer.fcgi?val=GQ375286) | 1434 | Russia | 26-may |
| 484 | [CY046811](http://www.ncbi.nlm.nih.gov/entrez/viewer.fcgi?val=CY046811) | 1734 | USA | 27-may |  | [GU433027](http://www.ncbi.nlm.nih.gov/entrez/viewer.fcgi?val=GU433027) | 1434 | Russia | 19-jul |
| 485 | [CY046835](http://www.ncbi.nlm.nih.gov/entrez/viewer.fcgi?val=CY046835) | 1721 | USA | 27-may |  | [GU433035](http://www.ncbi.nlm.nih.gov/entrez/viewer.fcgi?val=GU433035) | 1434 | Russia | 21-jul |
| 486 | [CY046827](http://www.ncbi.nlm.nih.gov/entrez/viewer.fcgi?val=CY046827) | 1721 | USA | 28-may | 349 | [GQ281077](http://www.ncbi.nlm.nih.gov/entrez/viewer.fcgi?val=GQ281077) | 811 | Ireland | 26-may |
|  | [CY049931](http://www.ncbi.nlm.nih.gov/entrez/viewer.fcgi?val=CY049931) | 1698 | USA | 27-may | 350 | [GQ283487](http://www.ncbi.nlm.nih.gov/entrez/viewer.fcgi?val=GQ283487) | 1434 | Finland | 26-may |
| 487 | [CY051985](http://www.ncbi.nlm.nih.gov/entrez/viewer.fcgi?val=CY051985) | 1744 | Norway | 27-may | 351 | [GQ359769](http://www.ncbi.nlm.nih.gov/entrez/viewer.fcgi?val=GQ359769) | 1426 | Sweden | 26-may |
| 488 | [CY053238](http://www.ncbi.nlm.nih.gov/entrez/viewer.fcgi?val=CY053238) | 1734 | USA | 27-may | 352 | [GQ385302](http://www.ncbi.nlm.nih.gov/entrez/viewer.fcgi?val=GQ385302) | 1447 | Canada | 26-may |
| 489 | [CY053246](http://www.ncbi.nlm.nih.gov/entrez/viewer.fcgi?val=CY053246) | 1734 | USA | 27-may | 353 | [CY044074](http://www.ncbi.nlm.nih.gov/entrez/viewer.fcgi?val=CY044074) | 1414 | USA | 26-may |
| 490 | [GQ265537](http://www.ncbi.nlm.nih.gov/entrez/viewer.fcgi?val=GQ265537) | 1553 | Spain | 28-may | 354 | [CY045153](http://www.ncbi.nlm.nih.gov/entrez/viewer.fcgi?val=CY045153) | 1416 | USA | 26-may |
| 491 | [GQ283493](http://www.ncbi.nlm.nih.gov/entrez/viewer.fcgi?val=GQ283493) | 1743 | Finland | 28-may | 355 | [CY050750](http://www.ncbi.nlm.nih.gov/entrez/viewer.fcgi?val=CY050750) | 1415 | USA | 26-may |
| 492 | [GQ288372](http://www.ncbi.nlm.nih.gov/entrez/viewer.fcgi?val=GQ288372) | 1763 | China | 28-may | 356 | [CY051249](http://www.ncbi.nlm.nih.gov/entrez/viewer.fcgi?val=CY051249) | 1419 | USA | 26-may |
| 493 | [GQ368665](http://www.ncbi.nlm.nih.gov/entrez/viewer.fcgi?val=GQ368665) | 1701 | Brazil | 28-may |  | [CY051441](http://www.ncbi.nlm.nih.gov/entrez/viewer.fcgi?val=CY051441) | 1419 | USA | 17-jun |
| 494 | [GQ402204](http://www.ncbi.nlm.nih.gov/entrez/viewer.fcgi?val=GQ402204) | 1743 | Canada | 28-may | 357 | [CY052071](http://www.ncbi.nlm.nih.gov/entrez/viewer.fcgi?val=CY052071) | 1089 | Italy | 26-may |
| 495 | [GQ411908](http://www.ncbi.nlm.nih.gov/entrez/viewer.fcgi?val=GQ411908) | 1701 | China | 28-may | 358 | [GQ232087](http://www.ncbi.nlm.nih.gov/entrez/viewer.fcgi?val=GQ232087) | 1410 | China | 27-may |
| 496 | [CY044220](http://www.ncbi.nlm.nih.gov/entrez/viewer.fcgi?val=CY044220) | 1704 | Taiwan | 28-may |  | [GQ250162](http://www.ncbi.nlm.nih.gov/entrez/viewer.fcgi?val=GQ250162) | 1410 | China | 29-may |
| 497 | [CY045005](http://www.ncbi.nlm.nih.gov/entrez/viewer.fcgi?val=CY045005) | 1734 | USA | 28-may |  | [GQ223436](http://www.ncbi.nlm.nih.gov/entrez/viewer.fcgi?val=GQ223436) | 1410 | China | 29-may |
| 498 | [CY046899](http://www.ncbi.nlm.nih.gov/entrez/viewer.fcgi?val=CY046899) | 1732 | USA | 28-may |  | [GU134716](http://www.ncbi.nlm.nih.gov/entrez/viewer.fcgi?val=GU134716) | 1410 | Italy | Jul |
| 499 | [CY046907](http://www.ncbi.nlm.nih.gov/entrez/viewer.fcgi?val=CY046907) | 1733 | USA | 28-may | 359 | [CY046789](http://www.ncbi.nlm.nih.gov/entrez/viewer.fcgi?val=CY046789) | 1420 | USA | 27-may |
| 500 | [CY050150](http://www.ncbi.nlm.nih.gov/entrez/viewer.fcgi?val=CY050150) | 1738 | USA | 28-may | 360 | [CY053902](http://www.ncbi.nlm.nih.gov/entrez/viewer.fcgi?val=CY053902) | 1391 | Argentina | 27-may |
| 501 | [CY050903](http://www.ncbi.nlm.nih.gov/entrez/viewer.fcgi?val=CY050903) | 1733 | USA | 28-may | 361 | [GQ283492](http://www.ncbi.nlm.nih.gov/entrez/viewer.fcgi?val=GQ283492) | 1434 | Finland | 28-may |
| 502 | [CY050911](http://www.ncbi.nlm.nih.gov/entrez/viewer.fcgi?val=CY050911) | 1733 | USA | 28-may | 362 | [GQ288374](http://www.ncbi.nlm.nih.gov/entrez/viewer.fcgi?val=GQ288374) | 1451 | China | 28-may |
| 503 | [CY053261](http://www.ncbi.nlm.nih.gov/entrez/viewer.fcgi?val=CY053261) | 1734 | USA | 28-may | 363 | [CY044222](http://www.ncbi.nlm.nih.gov/entrez/viewer.fcgi?val=CY044222) | 1410 | Taiwan | 28-may |
| 504 | [GQ250161](http://www.ncbi.nlm.nih.gov/entrez/viewer.fcgi?val=GQ250161) | 1701 | China | 29-may | 364 | [GQ387438](http://www.ncbi.nlm.nih.gov/entrez/viewer.fcgi?val=GQ387438) | 1237 | Spain | 28-may |
| 505 | [GQ359765](http://www.ncbi.nlm.nih.gov/entrez/viewer.fcgi?val=GQ359765) | 1756 | Sweden | 29-may | 365 | [CY046797](http://www.ncbi.nlm.nih.gov/entrez/viewer.fcgi?val=CY046797) | 1420 | USA | 28-may |
| 506 | [GQ414766](http://www.ncbi.nlm.nih.gov/entrez/viewer.fcgi?val=GQ414766) | 1701 | Brazil | 29-may |  | [CY046909](http://www.ncbi.nlm.nih.gov/entrez/viewer.fcgi?val=CY046909) | 1416 | USA | 28-may |
| 507 | [CY044080](http://www.ncbi.nlm.nih.gov/entrez/viewer.fcgi?val=CY044080) | 1732 | USA | 29-may | 366 | [CY050152](http://www.ncbi.nlm.nih.gov/entrez/viewer.fcgi?val=CY050152) | 1421 | USA | 28-may |
| 508 | [CY046946](http://www.ncbi.nlm.nih.gov/entrez/viewer.fcgi?val=CY046946) | 1744 | Norway | 29-may | 367 | [CY050913](http://www.ncbi.nlm.nih.gov/entrez/viewer.fcgi?val=CY050913) | 1420 | USA | 28-may |
| 509 | [CY049068](http://www.ncbi.nlm.nih.gov/entrez/viewer.fcgi?val=CY049068) | 1743 | Singapore | 29-may | 368 | [GQ281078](http://www.ncbi.nlm.nih.gov/entrez/viewer.fcgi?val=GQ281078) | 825 | Ireland | 29-may |
| 510 | [CY050919](http://www.ncbi.nlm.nih.gov/entrez/viewer.fcgi?val=CY050919) | 1717 | USA | 29-may | 369 | [GQ359766](http://www.ncbi.nlm.nih.gov/entrez/viewer.fcgi?val=GQ359766) | 1439 | Sweden | 29-may |
| 511 | [CY050935](http://www.ncbi.nlm.nih.gov/entrez/viewer.fcgi?val=CY050935) | 1733 | USA | 29-may | 370 | [GQ365366](http://www.ncbi.nlm.nih.gov/entrez/viewer.fcgi?val=GQ365366) | 1431 | Sweden | 29-may |
| 512 | [CY051255](http://www.ncbi.nlm.nih.gov/entrez/viewer.fcgi?val=CY051255) | 1733 | USA | 29-may | 371 | [CY044082](http://www.ncbi.nlm.nih.gov/entrez/viewer.fcgi?val=CY044082) | 1414 | USA | 29-may |
| 513 | [GQ360060](http://www.ncbi.nlm.nih.gov/entrez/viewer.fcgi?val=GQ360060) | 1751 | Sweden | 30-may | 372 | [CY046947](http://www.ncbi.nlm.nih.gov/entrez/viewer.fcgi?val=CY046947) | 1428 | Norway | 29-may |
| 514 | [GQ402206](http://www.ncbi.nlm.nih.gov/entrez/viewer.fcgi?val=GQ402206) | 1743 | Canada | 30-may | 373 | [GQ293078](http://www.ncbi.nlm.nih.gov/entrez/viewer.fcgi?val=GQ293078) | 1428 | China | 31-may |
| 515 | [CY045962](http://www.ncbi.nlm.nih.gov/entrez/viewer.fcgi?val=CY045962) | 1785 | Canada | 30-may | 374 | [CY049070](http://www.ncbi.nlm.nih.gov/entrez/viewer.fcgi?val=CY049070) | 1411 | Singapore | 29-may |
| 516 | [CY046803](http://www.ncbi.nlm.nih.gov/entrez/viewer.fcgi?val=CY046803) | 1735 | USA | 30-may | 375 | [CY050921](http://www.ncbi.nlm.nih.gov/entrez/viewer.fcgi?val=CY050921) | 1420 | USA | 29-may |
| 517 | [CY046949](http://www.ncbi.nlm.nih.gov/entrez/viewer.fcgi?val=CY046949) | 1744 | Norway | 30-may | 376 | [CY050929](http://www.ncbi.nlm.nih.gov/entrez/viewer.fcgi?val=CY050929) | 1420 | USA | 29-may |
| 518 | [CY053269](http://www.ncbi.nlm.nih.gov/entrez/viewer.fcgi?val=CY053269) | 1734 | USA | 30-may | 377 | [CY052072](http://www.ncbi.nlm.nih.gov/entrez/viewer.fcgi?val=CY052072) | 1089 | Italy | 29-may |
| 519 | [CY053277](http://www.ncbi.nlm.nih.gov/entrez/viewer.fcgi?val=CY053277) | 1726 | USA | 30-may |  | [CY052077](http://www.ncbi.nlm.nih.gov/entrez/viewer.fcgi?val=CY052077) | 1089 | Italy | 02-jun |
| 520 | [GQ253498](http://www.ncbi.nlm.nih.gov/entrez/viewer.fcgi?val=GQ253498) | 1701 | China | 31-may | 378 | [GQ360062](http://www.ncbi.nlm.nih.gov/entrez/viewer.fcgi?val=GQ360062) | 1428 | Sweden | 30-may |
| 521 | [GQ293077](http://www.ncbi.nlm.nih.gov/entrez/viewer.fcgi?val=GQ293077) | 1747 | China | 31-may | 379 | [GQ360064](http://www.ncbi.nlm.nih.gov/entrez/viewer.fcgi?val=GQ360064) | 1434 | Sweden | 30-may |
| 522 | [GQ377107](http://www.ncbi.nlm.nih.gov/entrez/viewer.fcgi?val=GQ377107) | 1701 | USA | 31-may | 380 | [CY044230](http://www.ncbi.nlm.nih.gov/entrez/viewer.fcgi?val=CY044230) | 1410 | Taiwan | 30-may |
| 523 | [CY044861](http://www.ncbi.nlm.nih.gov/entrez/viewer.fcgi?val=CY044861) | 1734 | USA | 31-may | 381 | [CY045964](http://www.ncbi.nlm.nih.gov/entrez/viewer.fcgi?val=CY045964) | 1438 | Canada | 30-may |
| 524 | [CY045183](http://www.ncbi.nlm.nih.gov/entrez/viewer.fcgi?val=CY045183) | 1732 | USA | 31-may | 382 | [CY046805](http://www.ncbi.nlm.nih.gov/entrez/viewer.fcgi?val=CY046805) | 1420 | USA | 30-may |
| 525 | [GQ330653](http://www.ncbi.nlm.nih.gov/entrez/viewer.fcgi?val=GQ330653) | 916 | Italy | Jun | 383 | [CY046821](http://www.ncbi.nlm.nih.gov/entrez/viewer.fcgi?val=CY046821) | 1413 | USA | 30-may |
| 526 | [GQ330654](http://www.ncbi.nlm.nih.gov/entrez/viewer.fcgi?val=GQ330654) | 908 | Italy | Jun | 384 | [CY046950](http://www.ncbi.nlm.nih.gov/entrez/viewer.fcgi?val=CY046950) | 1108 | Norway | 30-may |
| 527 | [GQ330655](http://www.ncbi.nlm.nih.gov/entrez/viewer.fcgi?val=GQ330655) | 913 | Italy | Jun | 385 | [CY053279](http://www.ncbi.nlm.nih.gov/entrez/viewer.fcgi?val=CY053279) | 1421 | USA | 30-may |
| 528 | [GQ359758](http://www.ncbi.nlm.nih.gov/entrez/viewer.fcgi?val=GQ359758) | 1795 | China | Jun | 386 | [GQ253504](http://www.ncbi.nlm.nih.gov/entrez/viewer.fcgi?val=GQ253504) | 1410 | China | 31-may |
| 529 | [CY045226](http://www.ncbi.nlm.nih.gov/entrez/viewer.fcgi?val=CY045226) | 1767 | Taiwan | Jun | 387 | [GQ359760](http://www.ncbi.nlm.nih.gov/entrez/viewer.fcgi?val=GQ359760) | 1462 | China | Jun |
| 530 | [CY045234](http://www.ncbi.nlm.nih.gov/entrez/viewer.fcgi?val=CY045234) | 1748 | Taiwan | Jun | 388 | [CY045228](http://www.ncbi.nlm.nih.gov/entrez/viewer.fcgi?val=CY045228) | 1438 | Taiwan | Jun |
| 531 | [CY045242](http://www.ncbi.nlm.nih.gov/entrez/viewer.fcgi?val=CY045242) | 1735 | Taiwan | Jun | 389 | [CY045236](http://www.ncbi.nlm.nih.gov/entrez/viewer.fcgi?val=CY045236) | 1455 | Taiwan | Jun |
| 532 | [GU369647](http://www.ncbi.nlm.nih.gov/entrez/viewer.fcgi?val=GU369647) | 1754 | Turkey | Jun | 390 | [CY045244](http://www.ncbi.nlm.nih.gov/entrez/viewer.fcgi?val=CY045244) | 1438 | Taiwan | Jun |
| 533 | [GQ338381](http://www.ncbi.nlm.nih.gov/entrez/viewer.fcgi?val=GQ338381) | 1701 | USA | 01-jun | 391 | [CY051473](http://www.ncbi.nlm.nih.gov/entrez/viewer.fcgi?val=CY051473) | 1418 | USA | Jun |
| 534 | [GQ356787](http://www.ncbi.nlm.nih.gov/entrez/viewer.fcgi?val=GQ356787) | 1701 | Brazil | 01-jun | 392 | [GQ281080](http://www.ncbi.nlm.nih.gov/entrez/viewer.fcgi?val=GQ281080) | 841 | Ireland | 01-jun |
| 535 | [GQ365368](http://www.ncbi.nlm.nih.gov/entrez/viewer.fcgi?val=GQ365368) | 1740 | Sweden | 01-jun | 393 | [GQ281081](http://www.ncbi.nlm.nih.gov/entrez/viewer.fcgi?val=GQ281081) | 839 | Ireland | 01-jun |
| 536 | [CY045175](http://www.ncbi.nlm.nih.gov/entrez/viewer.fcgi?val=CY045175) | 1734 | USA | 01-jun | 394 | [GQ338384](http://www.ncbi.nlm.nih.gov/entrez/viewer.fcgi?val=GQ338384) | 1410 | USA | 01-jun |
| 537 | [CY047366](http://www.ncbi.nlm.nih.gov/entrez/viewer.fcgi?val=CY047366) | 1734 | USA | 01-jun | 395 | [GQ365370](http://www.ncbi.nlm.nih.gov/entrez/viewer.fcgi?val=GQ365370) | 1332 | Sweden | 01-jun |
| 538 | [CY045013](http://www.ncbi.nlm.nih.gov/entrez/viewer.fcgi?val=CY045013) | 1734 | USA | 02-jun | 396 | [CY047368](http://www.ncbi.nlm.nih.gov/entrez/viewer.fcgi?val=CY047368) | 1422 | USA | 01-jun |
| 538 | [CY047374](http://www.ncbi.nlm.nih.gov/entrez/viewer.fcgi?val=CY047374) | 1732 | USA | 01-jun | 397 | [CY050961](http://www.ncbi.nlm.nih.gov/entrez/viewer.fcgi?val=CY050961) | 1420 | USA | 01-jun |
| 539 | [CY050951](http://www.ncbi.nlm.nih.gov/entrez/viewer.fcgi?val=CY050951) | 1733 | USA | 01-jun | 398 | [CY053287](http://www.ncbi.nlm.nih.gov/entrez/viewer.fcgi?val=CY053287) | 1420 | USA | 01-jun |
| 540 | [CY050959](http://www.ncbi.nlm.nih.gov/entrez/viewer.fcgi?val=CY050959) | 1736 | USA | 01-jun |  | [CY053303](http://www.ncbi.nlm.nih.gov/entrez/viewer.fcgi?val=CY053303) | 1420 | USA | 02-jun |
| 541 | [CY050967](http://www.ncbi.nlm.nih.gov/entrez/viewer.fcgi?val=CY050967) | 1736 | USA | 01-jun | 399 | [CY052076](http://www.ncbi.nlm.nih.gov/entrez/viewer.fcgi?val=CY052076) | 1089 | Italy | 01-jun |
| 542 | [CY053285](http://www.ncbi.nlm.nih.gov/entrez/viewer.fcgi?val=CY053285) | 1734 | USA | 01-jun | 400 | [GQ475991](http://www.ncbi.nlm.nih.gov/entrez/viewer.fcgi?val=GQ475991) | 1413 | USA | 02-jun |
| 543 | [GQ287625](http://www.ncbi.nlm.nih.gov/entrez/viewer.fcgi?val=GQ287625) | 1701 | Japan | 02-jun | 401 | [CY044999](http://www.ncbi.nlm.nih.gov/entrez/viewer.fcgi?val=CY044999) | 1419 | USA | 02-jun |
| 544 | [GQ329076](http://www.ncbi.nlm.nih.gov/entrez/viewer.fcgi?val=GQ329076) | 1701 | France | 02-jun | 402 | [CY045015](http://www.ncbi.nlm.nih.gov/entrez/viewer.fcgi?val=CY045015) | 1419 | USA | 02-jun |
| 545 | [GQ475990](http://www.ncbi.nlm.nih.gov/entrez/viewer.fcgi?val=GQ475990) | 1698 | USA | 02-jun | 403 | [CY049078](http://www.ncbi.nlm.nih.gov/entrez/viewer.fcgi?val=CY049078) | 1408 | Singapore | 02-jun |
| 546 | [GQ476058](http://www.ncbi.nlm.nih.gov/entrez/viewer.fcgi?val=GQ476058) | 1698 | USA | 02-jun | 404 | [CY051225](http://www.ncbi.nlm.nih.gov/entrez/viewer.fcgi?val=CY051225) | 1417 | USA | 02-jun |
| 547 | [CY049076](http://www.ncbi.nlm.nih.gov/entrez/viewer.fcgi?val=CY049076) | 1743 | Singapore | 02-jun | 405 | [CY053295](http://www.ncbi.nlm.nih.gov/entrez/viewer.fcgi?val=CY053295) | 1413 | USA | 02-jun |
| 548 | [CY050975](http://www.ncbi.nlm.nih.gov/entrez/viewer.fcgi?val=CY050975) | 1734 | USA | 02-jun | 406 | [GQ281082](http://www.ncbi.nlm.nih.gov/entrez/viewer.fcgi?val=GQ281082) | 825 | Ireland | 03-jun |
| 549 | [CY050983](http://www.ncbi.nlm.nih.gov/entrez/viewer.fcgi?val=CY050983) | 1734 | USA | 02-jun |  | [GQ281084](http://www.ncbi.nlm.nih.gov/entrez/viewer.fcgi?val=GQ281084) | 825 | Ireland | 04-jun |
| 550 | [CY051223](http://www.ncbi.nlm.nih.gov/entrez/viewer.fcgi?val=CY051223) | 1721 | USA | 02-jun | 407 | [GQ330646](http://www.ncbi.nlm.nih.gov/entrez/viewer.fcgi?val=GQ330646) | 1433 | Russia | 03-jun |
| 551 | [CY051263](http://www.ncbi.nlm.nih.gov/entrez/viewer.fcgi?val=CY051263) | 1734 | USA | 02-jun | 408 | [GQ396735](http://www.ncbi.nlm.nih.gov/entrez/viewer.fcgi?val=GQ396735) | 1410 | USA | 03-jun |
|  | [CY051271](http://www.ncbi.nlm.nih.gov/entrez/viewer.fcgi?val=CY051271) | 1734 | USA | 02-jun | 409 | [GQ411899](http://www.ncbi.nlm.nih.gov/entrez/viewer.fcgi?val=GQ411899) | 1434 | Canada | 03-jun |
| 552 | [CY051287](http://www.ncbi.nlm.nih.gov/entrez/viewer.fcgi?val=CY051287) | 1734 | USA | 04-jun | 410 | [CY045497](http://www.ncbi.nlm.nih.gov/entrez/viewer.fcgi?val=CY045497) | 1441 | Germany | 03-jun |
|  | [CY051063](http://www.ncbi.nlm.nih.gov/entrez/viewer.fcgi?val=CY051063) | 1734 | USA | 08-jun | 411 | [CY043336](http://www.ncbi.nlm.nih.gov/entrez/viewer.fcgi?val=CY043336) | 1423 | Denmark | 04-jun |
|  | [CY051367](http://www.ncbi.nlm.nih.gov/entrez/viewer.fcgi?val=CY051367) | 1734 | USA | 10-jun | 412 | [CY043344](http://www.ncbi.nlm.nih.gov/entrez/viewer.fcgi?val=CY043344) | 1422 | Denmark | 04-jun |
|  | [CY051079](http://www.ncbi.nlm.nih.gov/entrez/viewer.fcgi?val=CY051079) | 1734 | USA | 11-jun | 413 | [CY051017](http://www.ncbi.nlm.nih.gov/entrez/viewer.fcgi?val=CY051017) | 1419 | USA | 04-jun |
|  | [CY053293](http://www.ncbi.nlm.nih.gov/entrez/viewer.fcgi?val=CY053293) | 1734 | USA | 02-jun | 414 | [CY051033](http://www.ncbi.nlm.nih.gov/entrez/viewer.fcgi?val=CY051033) | 1420 | USA | 04-jun |
| 553 | [CY053301](http://www.ncbi.nlm.nih.gov/entrez/viewer.fcgi?val=CY053301) | 1734 | USA | 02-jun | 415 | [CY051041](http://www.ncbi.nlm.nih.gov/entrez/viewer.fcgi?val=CY051041) | 1421 | USA | 04-jun |
|  | [GQ259997](http://www.ncbi.nlm.nih.gov/entrez/viewer.fcgi?val=GQ259997) | 906 | Italy | 03-jun | 416 | [CY044106](http://www.ncbi.nlm.nih.gov/entrez/viewer.fcgi?val=CY044106) | 1414 | USA | 05-jun |
| 554 | [GQ265538](http://www.ncbi.nlm.nih.gov/entrez/viewer.fcgi?val=GQ265538) | 1550 | Spain | 03-jun | 417 | [CY050993](http://www.ncbi.nlm.nih.gov/entrez/viewer.fcgi?val=CY050993) | 1420 | USA | 05-jun |
| 555 | [GQ330645](http://www.ncbi.nlm.nih.gov/entrez/viewer.fcgi?val=GQ330645) | 1752 | Russia | 03-jun | 418 | [CY052140](http://www.ncbi.nlm.nih.gov/entrez/viewer.fcgi?val=CY052140) | 1420 | USA | 05-jun |
| 556 | [GQ411897](http://www.ncbi.nlm.nih.gov/entrez/viewer.fcgi?val=GQ411897) | 1766 | Canada | 03-jun | 418 | [CY045063](http://www.ncbi.nlm.nih.gov/entrez/viewer.fcgi?val=CY045063) | 1420 | USA | 13-jun |
| 557 | [CY044096](http://www.ncbi.nlm.nih.gov/entrez/viewer.fcgi?val=CY044096) | 1732 | USA | 03-jun | 419 | [CY052080](http://www.ncbi.nlm.nih.gov/entrez/viewer.fcgi?val=CY052080) | 1071 | Italy | 05-jun |
| 558 | [AB514226](http://www.ncbi.nlm.nih.gov/entrez/viewer.fcgi?val=AB514226) | 1701 | Japan | 03-jun | 420 | [CY053898](http://www.ncbi.nlm.nih.gov/entrez/viewer.fcgi?val=CY053898) | 1418 | Argentina | 05-jun |
| 559 | [CY045495](http://www.ncbi.nlm.nih.gov/entrez/viewer.fcgi?val=CY045495) | 1752 | Germany | 03-jun | 421 | [GQ365363](http://www.ncbi.nlm.nih.gov/entrez/viewer.fcgi?val=GQ365363) | 1446 | Sweden | 06-jun |
| 560 | [GU014808](http://www.ncbi.nlm.nih.gov/entrez/viewer.fcgi?val=GU014808) | 1701 | Japan | 03-jun | 422 | [GQ369275](http://www.ncbi.nlm.nih.gov/entrez/viewer.fcgi?val=GQ369275) | 1434 | Sweden | 06-jun |
| 561 | [CY051231](http://www.ncbi.nlm.nih.gov/entrez/viewer.fcgi?val=CY051231) | 1721 | USA | 03-jun | 423 | [GQ411905](http://www.ncbi.nlm.nih.gov/entrez/viewer.fcgi?val=GQ411905) | 1410 | China | 06-jun |
| 562 | [CY043334](http://www.ncbi.nlm.nih.gov/entrez/viewer.fcgi?val=CY043334) | 1733 | Denmark | 04-jun | 424 | [GU012634](http://www.ncbi.nlm.nih.gov/entrez/viewer.fcgi?val=GU012634) | 1410 | Poland | 06-jun |
| 563 | [CY043342](http://www.ncbi.nlm.nih.gov/entrez/viewer.fcgi?val=CY043342) | 1733 | Denmark | 04-jun | 425 | [GQ287620](http://www.ncbi.nlm.nih.gov/entrez/viewer.fcgi?val=GQ287620) | 1410 | Japan | 07-jun |
| 564 | [CY043350](http://www.ncbi.nlm.nih.gov/entrez/viewer.fcgi?val=CY043350) | 1733 | Denmark | 09-jun | 426 | [GQ365685](http://www.ncbi.nlm.nih.gov/entrez/viewer.fcgi?val=GQ365685) | 1449 | Sweden | 08-jun |
|  | [CY051015](http://www.ncbi.nlm.nih.gov/entrez/viewer.fcgi?val=CY051015) | 1734 | USA | 04-jun | 427 | [CY045209](http://www.ncbi.nlm.nih.gov/entrez/viewer.fcgi?val=CY045209) | 1418 | USA | 08-jun |
| 565 | [CY051023](http://www.ncbi.nlm.nih.gov/entrez/viewer.fcgi?val=CY051023) | 1734 | USA | 04-jun | 428 | [GU012635](http://www.ncbi.nlm.nih.gov/entrez/viewer.fcgi?val=GU012635) | 1410 | Poland | 08-jun |
| 566 | [CY051335](http://www.ncbi.nlm.nih.gov/entrez/viewer.fcgi?val=CY051335) | 1734 | USA | 09-jun |  | [CY051297](http://www.ncbi.nlm.nih.gov/entrez/viewer.fcgi?val=CY051297) | 1421 | USA | 08-jun |
|  | [CY051031](http://www.ncbi.nlm.nih.gov/entrez/viewer.fcgi?val=CY051031) | 1734 | USA | 04-jun | 429 | [CY051313](http://www.ncbi.nlm.nih.gov/entrez/viewer.fcgi?val=CY051313) | 1420 | USA | 08-jun |
| 567 | [CY051047](http://www.ncbi.nlm.nih.gov/entrez/viewer.fcgi?val=CY051047) | 1715 | USA | 04-jun | 430 | [GQ365680](http://www.ncbi.nlm.nih.gov/entrez/viewer.fcgi?val=GQ365680) | 1442 | Sweden | 09-jun |
| 568 | [GQ259998](http://www.ncbi.nlm.nih.gov/entrez/viewer.fcgi?val=GQ259998) | 903 | Italy | 05-jun | 431 | [GQ365682](http://www.ncbi.nlm.nih.gov/entrez/viewer.fcgi?val=GQ365682) | 1449 | Sweden | 09-jun |
| 569 | [GQ259999](http://www.ncbi.nlm.nih.gov/entrez/viewer.fcgi?val=GQ259999) | 910 | Italy | 05-jun | 432 | [GQ365688](http://www.ncbi.nlm.nih.gov/entrez/viewer.fcgi?val=GQ365688) | 1384 | Sweden | 09-jun |
| 570 | [GQ260000](http://www.ncbi.nlm.nih.gov/entrez/viewer.fcgi?val=GQ260000) | 891 | Italy | 05-jun | 433 | [GQ367304](http://www.ncbi.nlm.nih.gov/entrez/viewer.fcgi?val=GQ367304) | 1447 | Sweden | 09-jun |
| 571 | [GQ260001](http://www.ncbi.nlm.nih.gov/entrez/viewer.fcgi?val=GQ260001) | 903 | Italy | 05-jun | 434 | [CY043352](http://www.ncbi.nlm.nih.gov/entrez/viewer.fcgi?val=CY043352) | 1424 | Denmark | 09-jun |
| 572 | [GQ280797](http://www.ncbi.nlm.nih.gov/entrez/viewer.fcgi?val=GQ280797) | 1701 | USA | 05-jun | 435 | [CY045972](http://www.ncbi.nlm.nih.gov/entrez/viewer.fcgi?val=CY045972) | 1448 | Canada | 09-jun |
| 573 | [CY053896](http://www.ncbi.nlm.nih.gov/entrez/viewer.fcgi?val=CY053896) | 1720 | Argentina | 05-jun | 436 | [GU012636](http://www.ncbi.nlm.nih.gov/entrez/viewer.fcgi?val=GU012636) | 1410 | Poland | 09-jun |
| 574 | [GQ351319](http://www.ncbi.nlm.nih.gov/entrez/viewer.fcgi?val=GQ351319) | 1747 | Italy | 06-jun | 437 | [CY049885](http://www.ncbi.nlm.nih.gov/entrez/viewer.fcgi?val=CY049885) | 1410 | El Salvador | 09-jun |
| 575 | [GQ411907](http://www.ncbi.nlm.nih.gov/entrez/viewer.fcgi?val=GQ411907) | 1701 | China | 06-jun | 438 | [CY045047](http://www.ncbi.nlm.nih.gov/entrez/viewer.fcgi?val=CY045047) | 1419 | USA | 10-jun |
| 576 | [GQ287619](http://www.ncbi.nlm.nih.gov/entrez/viewer.fcgi?val=GQ287619) | 1701 | Japan | 07-jun | 439 | [CY045201](http://www.ncbi.nlm.nih.gov/entrez/viewer.fcgi?val=CY045201) | 1419 | USA | 10-jun |
| 577 | [GQ334330](http://www.ncbi.nlm.nih.gov/entrez/viewer.fcgi?val=GQ334330) | 1701 | Japan | 07-jun | 440 | [CY051393](http://www.ncbi.nlm.nih.gov/entrez/viewer.fcgi?val=CY051393) | 1414 | USA | 10-jun |
|  | [GQ334338](http://www.ncbi.nlm.nih.gov/entrez/viewer.fcgi?val=GQ334338) | 1701 | Japan | 07-jun | 441 | [CY052268](http://www.ncbi.nlm.nih.gov/entrez/viewer.fcgi?val=CY052268) | 1420 | USA | 10-jun |
|  | [GQ365436](http://www.ncbi.nlm.nih.gov/entrez/viewer.fcgi?val=GQ365436) | 1701 | Japan | 13-jun | 442 | [GQ351316](http://www.ncbi.nlm.nih.gov/entrez/viewer.fcgi?val=GQ351316) | 1410 | Hong Kong | 11-jun |
|  | [CY053119](http://www.ncbi.nlm.nih.gov/entrez/viewer.fcgi?val=CY053119) | 1734 | USA | 07-jun | 443 | [GQ369279](http://www.ncbi.nlm.nih.gov/entrez/viewer.fcgi?val=GQ369279) | 1438 | Sweden | 11-jun |
| 578 | [CY053111](http://www.ncbi.nlm.nih.gov/entrez/viewer.fcgi?val=CY053111) | 1734 | USA | 11-jun | 444 | [CY051409](http://www.ncbi.nlm.nih.gov/entrez/viewer.fcgi?val=CY051409) | 1419 | USA | 11-jun |
|  | [GQ301888](http://www.ncbi.nlm.nih.gov/entrez/viewer.fcgi?val=GQ301888) | 976 | Italy | 08-jun | 445 | [CY051881](http://www.ncbi.nlm.nih.gov/entrez/viewer.fcgi?val=CY051881) | 1420 | USA | 11-jun |
| 579 | [GQ421199](http://www.ncbi.nlm.nih.gov/entrez/viewer.fcgi?val=GQ421199) | 1701 | Italy | 08-jun | 446 | [CY053975](http://www.ncbi.nlm.nih.gov/entrez/viewer.fcgi?val=CY053975) | 1410 | Argentina | 11-jun |
| 580 | [CY051055](http://www.ncbi.nlm.nih.gov/entrez/viewer.fcgi?val=CY051055) | 1732 | USA | 08-jun | 447 | [CY049893](http://www.ncbi.nlm.nih.gov/entrez/viewer.fcgi?val=CY049893) | 1410 | El Salvador | 12-jun |
| 581 | [CY051071](http://www.ncbi.nlm.nih.gov/entrez/viewer.fcgi?val=CY051071) | 1732 | USA | 08-jun | 448 | [CY050160](http://www.ncbi.nlm.nih.gov/entrez/viewer.fcgi?val=CY050160) | 1420 | USA | 12-jun |
| 582 | [CY051495](http://www.ncbi.nlm.nih.gov/entrez/viewer.fcgi?val=CY051495) | 1734 | USA | 08-jun | 449 | [CY051841](http://www.ncbi.nlm.nih.gov/entrez/viewer.fcgi?val=CY051841) | 1420 | USA | 12-jun |
| 583 | [CY045037](http://www.ncbi.nlm.nih.gov/entrez/viewer.fcgi?val=CY045037) | 1720 | USA | 09-jun | 450 | [CY051857](http://www.ncbi.nlm.nih.gov/entrez/viewer.fcgi?val=CY051857) | 1419 | USA | 12-jun |
| 584 | [CY045053](http://www.ncbi.nlm.nih.gov/entrez/viewer.fcgi?val=CY045053) | 1721 | USA | 09-jun | 451 | [CY052196](http://www.ncbi.nlm.nih.gov/entrez/viewer.fcgi?val=CY052196) | 1420 | USA | 12-jun |
| 585 | [CY045970](http://www.ncbi.nlm.nih.gov/entrez/viewer.fcgi?val=CY045970) | 1783 | Canada | 09-jun |  | [CY052385](http://www.ncbi.nlm.nih.gov/entrez/viewer.fcgi?val=CY052385) | 1420 | USA | 16-jun |
| 586 | [CY049883](http://www.ncbi.nlm.nih.gov/entrez/viewer.fcgi?val=CY049883) | 1695 | El Salvador | 09-jun |  | [CY052244](http://www.ncbi.nlm.nih.gov/entrez/viewer.fcgi?val=CY052244) | 1420 | USA | 20-jun |
| 587 | [CY051511](http://www.ncbi.nlm.nih.gov/entrez/viewer.fcgi?val=CY051511) | 1734 | USA | 09-jun |  | [CY052332](http://www.ncbi.nlm.nih.gov/entrez/viewer.fcgi?val=CY052332) | 1420 | USA | 21-jun |
| 588 | [CY052122](http://www.ncbi.nlm.nih.gov/entrez/viewer.fcgi?val=CY052122) | 1734 | USA | 09-jun |  | [CY052188](http://www.ncbi.nlm.nih.gov/entrez/viewer.fcgi?val=CY052188) | 1420 | USA | 28-jun |
| 589 | [CY044112](http://www.ncbi.nlm.nih.gov/entrez/viewer.fcgi?val=CY044112) | 1733 | USA | 10-jun |  | [CY053160](http://www.ncbi.nlm.nih.gov/entrez/viewer.fcgi?val=CY053160) | 1420 | USA | 07-jul |
| 590 | [CY045191](http://www.ncbi.nlm.nih.gov/entrez/viewer.fcgi?val=CY045191) | 1721 | USA | 10-jun | 452 | [CY052084](http://www.ncbi.nlm.nih.gov/entrez/viewer.fcgi?val=CY052084) | 1071 | Italy | 12-jun |
| 591 | [CY045199](http://www.ncbi.nlm.nih.gov/entrez/viewer.fcgi?val=CY045199) | 1721 | USA | 10-jun | 453 | [GQ463202](http://www.ncbi.nlm.nih.gov/entrez/viewer.fcgi?val=GQ463202) | 1410 | China | 13-jun |
| 592 | [CY051375](http://www.ncbi.nlm.nih.gov/entrez/viewer.fcgi?val=CY051375) | 1724 | USA | 10-jun | 454 | [GQ866953](http://www.ncbi.nlm.nih.gov/entrez/viewer.fcgi?val=GQ866953) | 1431 | Thailand | 13-jun |
| 593 | [CY051391](http://www.ncbi.nlm.nih.gov/entrez/viewer.fcgi?val=CY051391) | 1732 | USA | 10-jun | 455 | [GQ894817](http://www.ncbi.nlm.nih.gov/entrez/viewer.fcgi?val=GQ894817) | 1410 | USA | 13-jun |
| 594 | [CY051399](http://www.ncbi.nlm.nih.gov/entrez/viewer.fcgi?val=CY051399) | 1734 | USA | 10-jun | 456 | [GU014781](http://www.ncbi.nlm.nih.gov/entrez/viewer.fcgi?val=GU014781) | 1410 | Japan | 13-jun |
| 595 | [CY052266](http://www.ncbi.nlm.nih.gov/entrez/viewer.fcgi?val=CY052266) | 1734 | USA | 10-jun | 457 | [CY047384](http://www.ncbi.nlm.nih.gov/entrez/viewer.fcgi?val=CY047384) | 1412 | USA | 13-jun |
| 596 | [GQ286175](http://www.ncbi.nlm.nih.gov/entrez/viewer.fcgi?val=GQ286175) | 1795 | Chile | 11-jun | 458 | [GQ457464](http://www.ncbi.nlm.nih.gov/entrez/viewer.fcgi?val=GQ457464) | 1410 | USA | 14-jun |
| 597 | [GQ351314](http://www.ncbi.nlm.nih.gov/entrez/viewer.fcgi?val=GQ351314) | 1701 | Hong Kong | 11-jun | 459 | [CY049126](http://www.ncbi.nlm.nih.gov/entrez/viewer.fcgi?val=CY049126) | 1370 | Singapore | 14-jun |
| 598 | [GQ365446](http://www.ncbi.nlm.nih.gov/entrez/viewer.fcgi?val=GQ365446) | 1701 | Japan | 11-jun | 460 | [CY052393](http://www.ncbi.nlm.nih.gov/entrez/viewer.fcgi?val=CY052393) | 1420 | USA | 14-jun |
| 599 | [GQ421200](http://www.ncbi.nlm.nih.gov/entrez/viewer.fcgi?val=GQ421200) | 1701 | Italy | 11-jun | 461 | [GQ303552](http://www.ncbi.nlm.nih.gov/entrez/viewer.fcgi?val=GQ303552) | 1228 | Spain | 15-jun |
| 600 | [CY051407](http://www.ncbi.nlm.nih.gov/entrez/viewer.fcgi?val=CY051407) | 1734 | USA | 11-jun | 462 | [GQ328767](http://www.ncbi.nlm.nih.gov/entrez/viewer.fcgi?val=GQ328767) | 1410 | Greece | 15-jun |
| 601 | [CY051167](http://www.ncbi.nlm.nih.gov/entrez/viewer.fcgi?val=CY051167) | 1734 | USA | 18-jun |  | [GU014773](http://www.ncbi.nlm.nih.gov/entrez/viewer.fcgi?val=GU014773) | 1410 | Japan | 15-jun |
|  | [CY052351](http://www.ncbi.nlm.nih.gov/entrez/viewer.fcgi?val=CY052351) | 1371 | Brazil | 11-jun |  | [GU014803](http://www.ncbi.nlm.nih.gov/entrez/viewer.fcgi?val=GU014803) | 1410 | Japan | 15-jun |
| 602 | [CY053974](http://www.ncbi.nlm.nih.gov/entrez/viewer.fcgi?val=CY053974) | 1283 | Argentina | 11-jun | 463 | [CY047108](http://www.ncbi.nlm.nih.gov/entrez/viewer.fcgi?val=CY047108) | 1234 | Spain | 15-jun |
| 603 | [CY054283](http://www.ncbi.nlm.nih.gov/entrez/viewer.fcgi?val=CY054283) | 1701 | Brazil | 11-jun | 464 | [CY049134](http://www.ncbi.nlm.nih.gov/entrez/viewer.fcgi?val=CY049134) | 1405 | Singapore | 15-jun |
| 604 | [CY045077](http://www.ncbi.nlm.nih.gov/entrez/viewer.fcgi?val=CY045077) | 1733 | USA | 12-jun | 465 | [CY051009](http://www.ncbi.nlm.nih.gov/entrez/viewer.fcgi?val=CY051009) | 1419 | USA | 15-jun |
| 605 | [GQ894841](http://www.ncbi.nlm.nih.gov/entrez/viewer.fcgi?val=GQ894841) | 1701 | USA | 12-jun | 466 | [CY051089](http://www.ncbi.nlm.nih.gov/entrez/viewer.fcgi?val=CY051089) | 1412 | USA | 15-jun |
| 606 | [CY049891](http://www.ncbi.nlm.nih.gov/entrez/viewer.fcgi?val=CY049891) | 1701 | El Salvador | 12-jun | 467 | [CY051137](http://www.ncbi.nlm.nih.gov/entrez/viewer.fcgi?val=CY051137) | 1418 | USA | 15-jun |
| 607 | [CY050158](http://www.ncbi.nlm.nih.gov/entrez/viewer.fcgi?val=CY050158) | 1734 | USA | 12-jun | 468 | [CY052108](http://www.ncbi.nlm.nih.gov/entrez/viewer.fcgi?val=CY052108) | 1412 | USA | 15-jun |
| 608 | [CY051111](http://www.ncbi.nlm.nih.gov/entrez/viewer.fcgi?val=CY051111) | 1724 | USA | 12-jun | 469 | [GQ303553](http://www.ncbi.nlm.nih.gov/entrez/viewer.fcgi?val=GQ303553) | 1224 | Spain | 16-jun |
| 609 | [CY051839](http://www.ncbi.nlm.nih.gov/entrez/viewer.fcgi?val=CY051839) | 1724 | USA | 12-jun | 470 | [CY044173](http://www.ncbi.nlm.nih.gov/entrez/viewer.fcgi?val=CY044173) | 1410 | USA | 16-jun |
| 610 | [CY051855](http://www.ncbi.nlm.nih.gov/entrez/viewer.fcgi?val=CY051855) | 1734 | USA | 12-jun | 471 | [GQ902803](http://www.ncbi.nlm.nih.gov/entrez/viewer.fcgi?val=GQ902803) | 1464 | Thailand | 16-jun |
| 611 | [CY052194](http://www.ncbi.nlm.nih.gov/entrez/viewer.fcgi?val=CY052194) | 1734 | USA | 12-jun | 472 | [CY050088](http://www.ncbi.nlm.nih.gov/entrez/viewer.fcgi?val=CY050088) | 1410 | Colombia | 16-jun |
| 612 | [CY052383](http://www.ncbi.nlm.nih.gov/entrez/viewer.fcgi?val=CY052383) | 1734 | USA | 16-jun |  | [CY044149](http://www.ncbi.nlm.nih.gov/entrez/viewer.fcgi?val=CY044149) | 1410 | Colombia | 25-jun |
|  | [CY052242](http://www.ncbi.nlm.nih.gov/entrez/viewer.fcgi?val=CY052242) | 1734 | USA | 20-jun | 473 | [CY051145](http://www.ncbi.nlm.nih.gov/entrez/viewer.fcgi?val=CY051145) | 1417 | USA | 16-jun |
|  | [CY052330](http://www.ncbi.nlm.nih.gov/entrez/viewer.fcgi?val=CY052330) | 1734 | USA | 21-jun | 474 | [CY051465](http://www.ncbi.nlm.nih.gov/entrez/viewer.fcgi?val=CY051465) | 1419 | USA | 16-jun |
|  | [CY053158](http://www.ncbi.nlm.nih.gov/entrez/viewer.fcgi?val=CY053158) | 1734 | USA | 07-jul | 475 | [CY052212](http://www.ncbi.nlm.nih.gov/entrez/viewer.fcgi?val=CY052212) | 1420 | USA | 16-jun |
|  | [CY045061](http://www.ncbi.nlm.nih.gov/entrez/viewer.fcgi?val=CY045061) | 1738 | USA | 13-jun | 476 | [GQ392031](http://www.ncbi.nlm.nih.gov/entrez/viewer.fcgi?val=GQ392031) | 1412 | Italy | 17-jun |
| 613 | [GU014780](http://www.ncbi.nlm.nih.gov/entrez/viewer.fcgi?val=GU014780) | 1701 | Japan | 13-jun | 477 | [CY045980](http://www.ncbi.nlm.nih.gov/entrez/viewer.fcgi?val=CY045980) | 1458 | Canada | 17-jun |
| 614 | [CY047382](http://www.ncbi.nlm.nih.gov/entrez/viewer.fcgi?val=CY047382) | 1721 | USA | 13-jun | 478 | [GQ866961](http://www.ncbi.nlm.nih.gov/entrez/viewer.fcgi?val=GQ866961) | 1451 | Thailand | 17-jun |
| 615 | [CY050999](http://www.ncbi.nlm.nih.gov/entrez/viewer.fcgi?val=CY050999) | 1734 | USA | 13-jun | 479 | [CY049973](http://www.ncbi.nlm.nih.gov/entrez/viewer.fcgi?val=CY049973) | 1410 | Dominican Republic | 17-jun |
| 616 | [CY051095](http://www.ncbi.nlm.nih.gov/entrez/viewer.fcgi?val=CY051095) | 1725 | USA | 13-jun | 480 | [CY051545](http://www.ncbi.nlm.nih.gov/entrez/viewer.fcgi?val=CY051545) | 1420 | USA | 17-jun |
| 617 | [CY051103](http://www.ncbi.nlm.nih.gov/entrez/viewer.fcgi?val=CY051103) | 1713 | USA | 13-jun | 481 | [CY053937](http://www.ncbi.nlm.nih.gov/entrez/viewer.fcgi?val=CY053937) | 1266 | Argentina | 17-jun |
| 618 | [GQ457479](http://www.ncbi.nlm.nih.gov/entrez/viewer.fcgi?val=GQ457479) | 1701 | USA | 14-jun | 482 | [GQ303554](http://www.ncbi.nlm.nih.gov/entrez/viewer.fcgi?val=GQ303554) | 1230 | Spain | 18-jun |
| 619 | [GQ527163](http://www.ncbi.nlm.nih.gov/entrez/viewer.fcgi?val=GQ527163) | 1721 | Singapore | 14-jun | 483 | [GQ902835](http://www.ncbi.nlm.nih.gov/entrez/viewer.fcgi?val=GQ902835) | 1471 | Thailand | 18-jun |
| 620 | [GQ527164](http://www.ncbi.nlm.nih.gov/entrez/viewer.fcgi?val=GQ527164) | 1721 | Singapore | 14-jun | 484 | [CY051449](http://www.ncbi.nlm.nih.gov/entrez/viewer.fcgi?val=CY051449) | 1418 | USA | 18-jun |
| 621 | [GQ527165](http://www.ncbi.nlm.nih.gov/entrez/viewer.fcgi?val=GQ527165) | 1721 | Singapore | 14-jun | 485 | [CY053930](http://www.ncbi.nlm.nih.gov/entrez/viewer.fcgi?val=CY053930) | 1410 | Argentina | 18-jun |
| 622 | [GQ527166](http://www.ncbi.nlm.nih.gov/entrez/viewer.fcgi?val=GQ527166) | 1721 | Singapore | 14-jun | 486 | [CY045071](http://www.ncbi.nlm.nih.gov/entrez/viewer.fcgi?val=CY045071) | 1420 | USA | 19-jun |
| 623 | [CY049124](http://www.ncbi.nlm.nih.gov/entrez/viewer.fcgi?val=CY049124) | 1689 | Singapore | 14-jun | 487 | [CY049086](http://www.ncbi.nlm.nih.gov/entrez/viewer.fcgi?val=CY049086) | 1391 | Singapore | 19-jun |
| 624 | [CY051119](http://www.ncbi.nlm.nih.gov/entrez/viewer.fcgi?val=CY051119) | 1721 | USA | 14-jun | 488 | [CY049366](http://www.ncbi.nlm.nih.gov/entrez/viewer.fcgi?val=CY049366) | 1429 | Singapore | 19-jun |
| 625 | [CY051127](http://www.ncbi.nlm.nih.gov/entrez/viewer.fcgi?val=CY051127) | 1721 | USA | 14-jun |  | [CY049062](http://www.ncbi.nlm.nih.gov/entrez/viewer.fcgi?val=CY049062) | 1429 | Singapore | 20-jun |
| 626 | [CY051151](http://www.ncbi.nlm.nih.gov/entrez/viewer.fcgi?val=CY051151) | 1734 | USA | 14-jun | 489 | [CY051849](http://www.ncbi.nlm.nih.gov/entrez/viewer.fcgi?val=CY051849) | 1419 | USA | 19-jun |
| 627 | [GQ293442](http://www.ncbi.nlm.nih.gov/entrez/viewer.fcgi?val=GQ293442) | 1550 | Spain | 15-jun | 490 | [CY052236](http://www.ncbi.nlm.nih.gov/entrez/viewer.fcgi?val=CY052236) | 1420 | USA | 19-jun |
| 628 | [GQ457514](http://www.ncbi.nlm.nih.gov/entrez/viewer.fcgi?val=GQ457514) | 1701 | USA | 15-jun | 491 | [CY052085](http://www.ncbi.nlm.nih.gov/entrez/viewer.fcgi?val=CY052085) | 1410 | Italy | 19-jun |
| 629 | [GU014772](http://www.ncbi.nlm.nih.gov/entrez/viewer.fcgi?val=GU014772) | 1701 | Japan | 15-jun | 492 | [CY053917](http://www.ncbi.nlm.nih.gov/entrez/viewer.fcgi?val=CY053917) | 1324 | Argentina | 19-jun |
| 630 | [GU014798](http://www.ncbi.nlm.nih.gov/entrez/viewer.fcgi?val=GU014798) | 1701 | Japan | 15-jun | 493 | [GQ494353](http://www.ncbi.nlm.nih.gov/entrez/viewer.fcgi?val=GQ494353) | 1433 | Russia | 20-jun |
| 631 | [CY049132](http://www.ncbi.nlm.nih.gov/entrez/viewer.fcgi?val=CY049132) | 1701 | Singapore | 15-jun | 494 | [CY049142](http://www.ncbi.nlm.nih.gov/entrez/viewer.fcgi?val=CY049142) | 1420 | Singapore | 20-jun |
| 632 | [CY051007](http://www.ncbi.nlm.nih.gov/entrez/viewer.fcgi?val=CY051007) | 1734 | USA | 15-jun | 495 | [CY051193](http://www.ncbi.nlm.nih.gov/entrez/viewer.fcgi?val=CY051193) | 1419 | USA | 20-jun |
| 633 | [CY051087](http://www.ncbi.nlm.nih.gov/entrez/viewer.fcgi?val=CY051087) | 1725 | USA | 15-jun | 496 | [CY051185](http://www.ncbi.nlm.nih.gov/entrez/viewer.fcgi?val=CY051185) | 1420 | USA | 21-jun |
| 634 | [CY051423](http://www.ncbi.nlm.nih.gov/entrez/viewer.fcgi?val=CY051423) | 1734 | USA | 15-jun | 496 | [CY051649](http://www.ncbi.nlm.nih.gov/entrez/viewer.fcgi?val=CY051649) | 1420 | USA | 30-jun |
| 635 | [CY052106](http://www.ncbi.nlm.nih.gov/entrez/viewer.fcgi?val=CY052106) | 1735 | USA | 15-jun | 497 | [CY054278](http://www.ncbi.nlm.nih.gov/entrez/viewer.fcgi?val=CY054278) | 1284 | Argentina | 21-jun |
| 636 | [GQ293443](http://www.ncbi.nlm.nih.gov/entrez/viewer.fcgi?val=GQ293443) | 1542 | Spain | 16-jun | 498 | [GQ396511](http://www.ncbi.nlm.nih.gov/entrez/viewer.fcgi?val=GQ396511) | 1332 | China | 22-jun |
| 637 | [GQ334355](http://www.ncbi.nlm.nih.gov/entrez/viewer.fcgi?val=GQ334355) | 1701 | Japan | 16-jun | 499 | [GU014765](http://www.ncbi.nlm.nih.gov/entrez/viewer.fcgi?val=GU014765) | 1410 | Japan | 22-jun |
| 638 | [GQ365455](http://www.ncbi.nlm.nih.gov/entrez/viewer.fcgi?val=GQ365455) | 1701 | Japan | 16-jun | 500 | [CY049094](http://www.ncbi.nlm.nih.gov/entrez/viewer.fcgi?val=CY049094) | 1390 | Singapore | 22-jun |
|  | [CY044171](http://www.ncbi.nlm.nih.gov/entrez/viewer.fcgi?val=CY044171) | 1701 | USA | 16-jun | 501 | [CY049358](http://www.ncbi.nlm.nih.gov/entrez/viewer.fcgi?val=CY049358) | 1398 | Singapore | 22-jun |
| 639 | [GQ902801](http://www.ncbi.nlm.nih.gov/entrez/viewer.fcgi?val=GQ902801) | 1773 | Thailand | 16-jun | 502 | [CY049374](http://www.ncbi.nlm.nih.gov/entrez/viewer.fcgi?val=CY049374) | 1420 | Singapore | 22-jun |
| 640 | [CY049836](http://www.ncbi.nlm.nih.gov/entrez/viewer.fcgi?val=CY049836) | 1698 | Colombia | 16-jun | 503 | [CY049853](http://www.ncbi.nlm.nih.gov/entrez/viewer.fcgi?val=CY049853) | 1410 | Bolivia | 22-jun |
| 641 | [CY052210](http://www.ncbi.nlm.nih.gov/entrez/viewer.fcgi?val=CY052210) | 1734 | USA | 16-jun | 504 | [CY053940](http://www.ncbi.nlm.nih.gov/entrez/viewer.fcgi?val=CY053940) | 1410 | Argentina | 22-jun |
| 642 | [GQ392029](http://www.ncbi.nlm.nih.gov/entrez/viewer.fcgi?val=GQ392029) | 1731 | Italy | 17-jun | 505 | [CY053948](http://www.ncbi.nlm.nih.gov/entrez/viewer.fcgi?val=CY053948) | 903 | Argentina | 22-jun |
| 643 | [CY044120](http://www.ncbi.nlm.nih.gov/entrez/viewer.fcgi?val=CY044120) | 1734 | USA | 17-jun | 506 | [CY053972](http://www.ncbi.nlm.nih.gov/entrez/viewer.fcgi?val=CY053972) | 1383 | Argentina | 22-jun |
| 644 | [CY045978](http://www.ncbi.nlm.nih.gov/entrez/viewer.fcgi?val=CY045978) | 1784 | Canada | 17-jun | 507 | [GQ387440](http://www.ncbi.nlm.nih.gov/entrez/viewer.fcgi?val=GQ387440) | 1234 | Spain | 23-jun |
| 645 | [GQ866959](http://www.ncbi.nlm.nih.gov/entrez/viewer.fcgi?val=GQ866959) | 1706 | Thailand | 17-jun | 508 | [GQ387441](http://www.ncbi.nlm.nih.gov/entrez/viewer.fcgi?val=GQ387441) | 1235 | Spain | 23-jun |
| 646 | [GU014750](http://www.ncbi.nlm.nih.gov/entrez/viewer.fcgi?val=GU014750) | 1701 | Japan | 17-jun | 509 | [GQ906584](http://www.ncbi.nlm.nih.gov/entrez/viewer.fcgi?val=GQ906584) | 1447 | Sweden | 23-jun |
| 647 | [CY051439](http://www.ncbi.nlm.nih.gov/entrez/viewer.fcgi?val=CY051439) | 1734 | USA | 17-jun | 510 | [CY049294](http://www.ncbi.nlm.nih.gov/entrez/viewer.fcgi?val=CY049294) | 1410 | Singapore | 23-jun |
| 648 | [CY051871](http://www.ncbi.nlm.nih.gov/entrez/viewer.fcgi?val=CY051871) | 1734 | USA | 17-jun | 511 | [CY049302](http://www.ncbi.nlm.nih.gov/entrez/viewer.fcgi?val=CY049302) | 1405 | Singapore | 23-jun |
| 649 | [CY052098](http://www.ncbi.nlm.nih.gov/entrez/viewer.fcgi?val=CY052098) | 1721 | USA | 17-jun | 512 | [CY049310](http://www.ncbi.nlm.nih.gov/entrez/viewer.fcgi?val=CY049310) | 1396 | Singapore | 23-jun |
| 650 | [CY053933](http://www.ncbi.nlm.nih.gov/entrez/viewer.fcgi?val=CY053933) | 1478 | Argentina | 17-jun |  | [CY049166](http://www.ncbi.nlm.nih.gov/entrez/viewer.fcgi?val=CY049166) | 1396 | Singapore | 26-jun |
| 651 | [GQ280264](http://www.ncbi.nlm.nih.gov/entrez/viewer.fcgi?val=GQ280264) | 1701 | China | 18-jun | 513 | [CY053951](http://www.ncbi.nlm.nih.gov/entrez/viewer.fcgi?val=CY053951) | 1412 | Argentina | 23-jun |
| 652 | [GQ293444](http://www.ncbi.nlm.nih.gov/entrez/viewer.fcgi?val=GQ293444) | 1540 | Spain | 18-jun | 514 | [CY049334](http://www.ncbi.nlm.nih.gov/entrez/viewer.fcgi?val=CY049334) | 1399 | Singapore | 24-jun |
| 653 | [GQ365428](http://www.ncbi.nlm.nih.gov/entrez/viewer.fcgi?val=GQ365428) | 1701 | Japan | 18-jun | 515 | [CY052148](http://www.ncbi.nlm.nih.gov/entrez/viewer.fcgi?val=CY052148) | 1420 | USA | 24-jun |
| 654 | [GQ374889](http://www.ncbi.nlm.nih.gov/entrez/viewer.fcgi?val=GQ374889) | 959 | Italy | 18-jun | 516 | [CY053943](http://www.ncbi.nlm.nih.gov/entrez/viewer.fcgi?val=CY053943) | 1333 | Argentina | 24-jun |
| 655 | [CY043118](http://www.ncbi.nlm.nih.gov/entrez/viewer.fcgi?val=CY043118) | 1701 | USA | 18-jun | 517 | [CY053954](http://www.ncbi.nlm.nih.gov/entrez/viewer.fcgi?val=CY053954) | 1422 | Argentina | 24-jun |
| 656 | [CY044179](http://www.ncbi.nlm.nih.gov/entrez/viewer.fcgi?val=CY044179) | 1704 | USA | 18-jun | 518 | [GQ355295](http://www.ncbi.nlm.nih.gov/entrez/viewer.fcgi?val=GQ355295) | 1235 | Spain | 25-jun |
| 657 | [CY044187](http://www.ncbi.nlm.nih.gov/entrez/viewer.fcgi?val=CY044187) | 1701 | USA | 18-jun |  | [GQ379836](http://www.ncbi.nlm.nih.gov/entrez/viewer.fcgi?val=GQ379836) | 1235 | Spain | 01-jul |
| 658 | [CY046923](http://www.ncbi.nlm.nih.gov/entrez/viewer.fcgi?val=CY046923) | 1034 | Chile | 18-jun |  | [GQ379841](http://www.ncbi.nlm.nih.gov/entrez/viewer.fcgi?val=GQ379841) | 1235 | Spain | 02-jul |
| 659 | [GQ902833](http://www.ncbi.nlm.nih.gov/entrez/viewer.fcgi?val=GQ902833) | 1784 | Thailand | 18-jun |  | [GQ387395](http://www.ncbi.nlm.nih.gov/entrez/viewer.fcgi?val=GQ387395) | 1235 | Spain | 04-jul |
| 660 | [GU014778](http://www.ncbi.nlm.nih.gov/entrez/viewer.fcgi?val=GU014778) | 1701 | Japan | 18-jun |  | [GU014789](http://www.ncbi.nlm.nih.gov/entrez/viewer.fcgi?val=GU014789) | 1410 | Japan | 25-jun |
| 661 | [CY051455](http://www.ncbi.nlm.nih.gov/entrez/viewer.fcgi?val=CY051455) | 1734 | USA | 18-jun | 519 | [CY049102](http://www.ncbi.nlm.nih.gov/entrez/viewer.fcgi?val=CY049102) | 1413 | Singapore | 25-jun |
| 662 | [CY053928](http://www.ncbi.nlm.nih.gov/entrez/viewer.fcgi?val=CY053928) | 1701 | Argentina | 18-jun | 520 | [CY049150](http://www.ncbi.nlm.nih.gov/entrez/viewer.fcgi?val=CY049150) | 1414 | Singapore | 25-jun |
| 663 | [GQ359771](http://www.ncbi.nlm.nih.gov/entrez/viewer.fcgi?val=GQ359771) | 1707 | Chile | 19-jun | 521 | [CY049158](http://www.ncbi.nlm.nih.gov/entrez/viewer.fcgi?val=CY049158) | 1388 | Singapore | 25-jun |
| 664 | [CY049084](http://www.ncbi.nlm.nih.gov/entrez/viewer.fcgi?val=CY049084) | 1743 | Singapore | 19-jun |  | [CY049437](http://www.ncbi.nlm.nih.gov/entrez/viewer.fcgi?val=CY049437) | 1388 | Singapore | 04-jul |
| 665 | [CY049060](http://www.ncbi.nlm.nih.gov/entrez/viewer.fcgi?val=CY049060) | 1743 | Singapore | 20-jun |  | [CY049485](http://www.ncbi.nlm.nih.gov/entrez/viewer.fcgi?val=CY049485) | 1388 | Singapore | 09-jul |
|  | [CY049396](http://www.ncbi.nlm.nih.gov/entrez/viewer.fcgi?val=CY049396) | 1743 | Singapore | 25-jun | 522 | [CY049318](http://www.ncbi.nlm.nih.gov/entrez/viewer.fcgi?val=CY049318) | 1400 | Singapore | 25-jun |
|  | [CY049404](http://www.ncbi.nlm.nih.gov/entrez/viewer.fcgi?val=CY049404) | 1743 | Singapore | 26-jun | 523 | [CY049326](http://www.ncbi.nlm.nih.gov/entrez/viewer.fcgi?val=CY049326) | 1392 | Singapore | 25-jun |
|  | [CY049340](http://www.ncbi.nlm.nih.gov/entrez/viewer.fcgi?val=CY049340) | 1743 | Singapore | 26-jun | 524 | [CY049382](http://www.ncbi.nlm.nih.gov/entrez/viewer.fcgi?val=CY049382) | 1400 | Singapore | 25-jun |
|  | [CY049108](http://www.ncbi.nlm.nih.gov/entrez/viewer.fcgi?val=CY049108) | 1743 | Singapore | 26-jun | 525 | [CY049390](http://www.ncbi.nlm.nih.gov/entrez/viewer.fcgi?val=CY049390) | 1404 | Singapore | 25-jun |
|  | [CY049220](http://www.ncbi.nlm.nih.gov/entrez/viewer.fcgi?val=CY049220) | 1743 | Singapore | 27-jun | 526 | [CY049398](http://www.ncbi.nlm.nih.gov/entrez/viewer.fcgi?val=CY049398) | 1406 | Singapore | 25-jun |
|  | [CY049523](http://www.ncbi.nlm.nih.gov/entrez/viewer.fcgi?val=CY049523) | 1743 | Singapore | 13-jul | 527 | [CY049981](http://www.ncbi.nlm.nih.gov/entrez/viewer.fcgi?val=CY049981) | 1410 | Dominican Republic | 25-jun |
|  | [CY049364](http://www.ncbi.nlm.nih.gov/entrez/viewer.fcgi?val=CY049364) | 1743 | Singapore | 19-jun | 528 | [CY050184](http://www.ncbi.nlm.nih.gov/entrez/viewer.fcgi?val=CY050184) | 1420 | USA | 25-jun |
| 666 | [CY049987](http://www.ncbi.nlm.nih.gov/entrez/viewer.fcgi?val=CY049987) | 1698 | Mexico | 19-jun | 529 | [CY053969](http://www.ncbi.nlm.nih.gov/entrez/viewer.fcgi?val=CY053969) | 1411 | Argentina | 25-jun |
| 667 | [CY051559](http://www.ncbi.nlm.nih.gov/entrez/viewer.fcgi?val=CY051559) | 1734 | USA | 19-jun | 530 | [GQ355296](http://www.ncbi.nlm.nih.gov/entrez/viewer.fcgi?val=GQ355296) | 1233 | Spain | 26-jun |
| 668 | [CY051847](http://www.ncbi.nlm.nih.gov/entrez/viewer.fcgi?val=CY051847) | 1734 | USA | 19-jun |  | [GQ387396](http://www.ncbi.nlm.nih.gov/entrez/viewer.fcgi?val=GQ387396) | 1233 | Spain | 05-jul |
| 669 | [CY053916](http://www.ncbi.nlm.nih.gov/entrez/viewer.fcgi?val=CY053916) | 1500 | Argentina | 19-jun | 531 | [GQ355297](http://www.ncbi.nlm.nih.gov/entrez/viewer.fcgi?val=GQ355297) | 1233 | Spain | 26-jun |
| 670 | [CY046061](http://www.ncbi.nlm.nih.gov/entrez/viewer.fcgi?val=CY046061) | 1701 | Italy | 20-jun | 532 | [CY044157](http://www.ncbi.nlm.nih.gov/entrez/viewer.fcgi?val=CY044157) | 1410 | Nicaragua | 26-jun |
| 671 | [CY049140](http://www.ncbi.nlm.nih.gov/entrez/viewer.fcgi?val=CY049140) | 1696 | Singapore | 20-jun | 533 | [CY049110](http://www.ncbi.nlm.nih.gov/entrez/viewer.fcgi?val=CY049110) | 1413 | Singapore | 26-jun |
| 672 | [CY051175](http://www.ncbi.nlm.nih.gov/entrez/viewer.fcgi?val=CY051175) | 1734 | USA | 20-jun | 534 | [CY049174](http://www.ncbi.nlm.nih.gov/entrez/viewer.fcgi?val=CY049174) | 1387 | Singapore | 26-jun |
| 673 | [CY051191](http://www.ncbi.nlm.nih.gov/entrez/viewer.fcgi?val=CY051191) | 1734 | USA | 20-jun | 536 | [CY049182](http://www.ncbi.nlm.nih.gov/entrez/viewer.fcgi?val=CY049182) | 1390 | Singapore | 26-jun |
| 674 | [CY051183](http://www.ncbi.nlm.nih.gov/entrez/viewer.fcgi?val=CY051183) | 1734 | USA | 21-jun | 537 | [CY049190](http://www.ncbi.nlm.nih.gov/entrez/viewer.fcgi?val=CY049190) | 1387 | Singapore | 26-jun |
| 675 | [CY053945](http://www.ncbi.nlm.nih.gov/entrez/viewer.fcgi?val=CY053945) | 1734 | Argentina | 21-jun | 538 | [CY049342](http://www.ncbi.nlm.nih.gov/entrez/viewer.fcgi?val=CY049342) | 1413 | Singapore | 26-jun |
| 676 | [GQ387429](http://www.ncbi.nlm.nih.gov/entrez/viewer.fcgi?val=GQ387429) | 1542 | Spain | 22-jun | 539 | [CY049350](http://www.ncbi.nlm.nih.gov/entrez/viewer.fcgi?val=CY049350) | 1400 | Singapore | 26-jun |
| 677 | [GU014764](http://www.ncbi.nlm.nih.gov/entrez/viewer.fcgi?val=GU014764) | 1701 | Japan | 22-jun | 540 | [CY049406](http://www.ncbi.nlm.nih.gov/entrez/viewer.fcgi?val=CY049406) | 1403 | Singapore | 26-jun |
| 678 | [GU014810](http://www.ncbi.nlm.nih.gov/entrez/viewer.fcgi?val=GU014810) | 1701 | Japan | 22-jun | 541 | [CY052116](http://www.ncbi.nlm.nih.gov/entrez/viewer.fcgi?val=CY052116) | 1420 | USA | 26-jun |
| 679 | [CY049092](http://www.ncbi.nlm.nih.gov/entrez/viewer.fcgi?val=CY049092) | 1742 | Singapore | 22-jun | 542 | [CY053957](http://www.ncbi.nlm.nih.gov/entrez/viewer.fcgi?val=CY053957) | 1410 | Argentina | 26-jun |
| 680 | [CY049356](http://www.ncbi.nlm.nih.gov/entrez/viewer.fcgi?val=CY049356) | 1741 | Singapore | 22-jun | 543 | [CY049198](http://www.ncbi.nlm.nih.gov/entrez/viewer.fcgi?val=CY049198) | 1392 | Singapore | 27-jun |
| 681 | [CY049292](http://www.ncbi.nlm.nih.gov/entrez/viewer.fcgi?val=CY049292) | 1741 | Singapore | 23-jun | 544 | [CY049206](http://www.ncbi.nlm.nih.gov/entrez/viewer.fcgi?val=CY049206) | 1394 | Singapore | 27-jun |
|  | [CY049300](http://www.ncbi.nlm.nih.gov/entrez/viewer.fcgi?val=CY049300) | 1741 | Singapore | 23-jun | 545 | [CY049214](http://www.ncbi.nlm.nih.gov/entrez/viewer.fcgi?val=CY049214) | 1399 | Singapore | 27-jun |
|  | [CY049204](http://www.ncbi.nlm.nih.gov/entrez/viewer.fcgi?val=CY049204) | 1741 | Singapore | 27-jun |  | [CY049222](http://www.ncbi.nlm.nih.gov/entrez/viewer.fcgi?val=CY049222) | 1399 | Singapore | 27-jun |
|  | [CY049228](http://www.ncbi.nlm.nih.gov/entrez/viewer.fcgi?val=CY049228) | 1741 | Singapore | 04-jul |  | [CY049517](http://www.ncbi.nlm.nih.gov/entrez/viewer.fcgi?val=CY049517) | 1399 | Singapore | 13-jul |
|  | [CY049372](http://www.ncbi.nlm.nih.gov/entrez/viewer.fcgi?val=CY049372) | 1743 | Singapore | 22-jun |  | [CY049581](http://www.ncbi.nlm.nih.gov/entrez/viewer.fcgi?val=CY049581) | 1399 | Singapore | 14-jul |
| 682 | [CY049851](http://www.ncbi.nlm.nih.gov/entrez/viewer.fcgi?val=CY049851) | 1695 | Bolivia | 22-jun | 546 | [CY051209](http://www.ncbi.nlm.nih.gov/entrez/viewer.fcgi?val=CY051209) | 1420 | USA | 27-jun |
| 683 | [CY053939](http://www.ncbi.nlm.nih.gov/entrez/viewer.fcgi?val=CY053939) | 1719 | Argentina | 22-jun | 547 | [CY053960](http://www.ncbi.nlm.nih.gov/entrez/viewer.fcgi?val=CY053960) | 1420 | Argentina | 27-jun |
| 684 | [CY053947](http://www.ncbi.nlm.nih.gov/entrez/viewer.fcgi?val=CY053947) | 1701 | Argentina | 22-jun | 548 | [CY046953](http://www.ncbi.nlm.nih.gov/entrez/viewer.fcgi?val=CY046953) | 1428 | Norway | 28-jun |
| 685 | [CY053971](http://www.ncbi.nlm.nih.gov/entrez/viewer.fcgi?val=CY053971) | 1701 | Argentina | 22-jun | 549 | [CY052087](http://www.ncbi.nlm.nih.gov/entrez/viewer.fcgi?val=CY052087) | 1410 | Italy | 28-jun |
| 686 | [GQ365418](http://www.ncbi.nlm.nih.gov/entrez/viewer.fcgi?val=GQ365418) | 1701 | Japan | 23-jun | 550 | [GQ355298](http://www.ncbi.nlm.nih.gov/entrez/viewer.fcgi?val=GQ355298) | 1234 | Spain | 29-jun |
| 687 | [GQ387430](http://www.ncbi.nlm.nih.gov/entrez/viewer.fcgi?val=GQ387430) | 1523 | Spain | 23-jun | 551 | [GQ355299](http://www.ncbi.nlm.nih.gov/entrez/viewer.fcgi?val=GQ355299) | 1234 | Spain | 29-jun |
| 688 | [GU014800](http://www.ncbi.nlm.nih.gov/entrez/viewer.fcgi?val=GU014800) | 1701 | Japan | 23-jun | 552 | [GQ355300](http://www.ncbi.nlm.nih.gov/entrez/viewer.fcgi?val=GQ355300) | 1234 | Spain | 29-jun |
| 689 | [CY049308](http://www.ncbi.nlm.nih.gov/entrez/viewer.fcgi?val=CY049308) | 1740 | Singapore | 23-jun | 553 | [GQ355301](http://www.ncbi.nlm.nih.gov/entrez/viewer.fcgi?val=GQ355301) | 1233 | Spain | 29-jun |
| 690 | [CY049196](http://www.ncbi.nlm.nih.gov/entrez/viewer.fcgi?val=CY049196) | 1740 | Singapore | 27-jun | 554 | [GQ355302](http://www.ncbi.nlm.nih.gov/entrez/viewer.fcgi?val=GQ355302) | 1233 | Spain | 29-jun |
|  | [CY049236](http://www.ncbi.nlm.nih.gov/entrez/viewer.fcgi?val=CY049236) | 1740 | Singapore | 04-jul |  | [GQ379837](http://www.ncbi.nlm.nih.gov/entrez/viewer.fcgi?val=GQ379837) | 1233 | Spain | 01-jul |
|  | [CY049451](http://www.ncbi.nlm.nih.gov/entrez/viewer.fcgi?val=CY049451) | 1740 | Singapore | 05-jul |  | [GQ379839](http://www.ncbi.nlm.nih.gov/entrez/viewer.fcgi?val=GQ379839) | 1233 | Spain | 02-jul |
|  | [CY049547](http://www.ncbi.nlm.nih.gov/entrez/viewer.fcgi?val=CY049547) | 1740 | Singapore | 12-jul | 555 | [GQ375278](http://www.ncbi.nlm.nih.gov/entrez/viewer.fcgi?val=GQ375278) | 805 | Ireland | 29-jun |
|  | [CY051479](http://www.ncbi.nlm.nih.gov/entrez/viewer.fcgi?val=CY051479) | 1734 | USA | 23-jun | 556 | [CY043080](http://www.ncbi.nlm.nih.gov/entrez/viewer.fcgi?val=CY043080) | 1410 | Japan | 29-jun |
| 691 | [CY051551](http://www.ncbi.nlm.nih.gov/entrez/viewer.fcgi?val=CY051551) | 1734 | USA | 23-jun |  | [CY050077](http://www.ncbi.nlm.nih.gov/entrez/viewer.fcgi?val=CY050077) | 1410 | Japan | 10-jul |
| 692 | [CY053950](http://www.ncbi.nlm.nih.gov/entrez/viewer.fcgi?val=CY053950) | 1701 | Argentina | 23-jun | 557 | [GQ463958](http://www.ncbi.nlm.nih.gov/entrez/viewer.fcgi?val=GQ463958) | 1422 | China | 29-jun |
| 693 | [GQ374891](http://www.ncbi.nlm.nih.gov/entrez/viewer.fcgi?val=GQ374891) | 916 | Italy | 24-jun | 558 | [CY053908](http://www.ncbi.nlm.nih.gov/entrez/viewer.fcgi?val=CY053908) | 1291 | Argentina | 29-jun |
| 694 | [CY046925](http://www.ncbi.nlm.nih.gov/entrez/viewer.fcgi?val=CY046925) | 998 | Chile | 24-jun | 559 | [GQ375279](http://www.ncbi.nlm.nih.gov/entrez/viewer.fcgi?val=GQ375279) | 825 | Ireland | 30-jun |
| 695 | [CY049332](http://www.ncbi.nlm.nih.gov/entrez/viewer.fcgi?val=CY049332) | 1743 | Singapore | 24-jun | 560 | [GQ379825](http://www.ncbi.nlm.nih.gov/entrez/viewer.fcgi?val=GQ379825) | 1233 | Spain | 30-jun |
| 696 | [CY049324](http://www.ncbi.nlm.nih.gov/entrez/viewer.fcgi?val=CY049324) | 1743 | Singapore | 25-jun | 561 | [GQ379826](http://www.ncbi.nlm.nih.gov/entrez/viewer.fcgi?val=GQ379826) | 1230 | Spain | 30-jun |
|  | [CY051471](http://www.ncbi.nlm.nih.gov/entrez/viewer.fcgi?val=CY051471) | 1734 | USA | 24-jun | 562 | [CY049413](http://www.ncbi.nlm.nih.gov/entrez/viewer.fcgi?val=CY049413) | 1284 | Singapore | 30-jun |
| 697 | [CY052146](http://www.ncbi.nlm.nih.gov/entrez/viewer.fcgi?val=CY052146) | 1724 | USA | 24-jun | 563 | [CY049965](http://www.ncbi.nlm.nih.gov/entrez/viewer.fcgi?val=CY049965) | 1410 | Dominican Republic | 30-jun |
| 698 | [CY053953](http://www.ncbi.nlm.nih.gov/entrez/viewer.fcgi?val=CY053953) | 1701 | Argentina | 24-jun |  | [CY049949](http://www.ncbi.nlm.nih.gov/entrez/viewer.fcgi?val=CY049949) | 1410 | Dominican Republic | 02-jul |
| 699 | [GQ355303](http://www.ncbi.nlm.nih.gov/entrez/viewer.fcgi?val=GQ355303) | 1540 | Spain | 25-jun |  | [CY049957](http://www.ncbi.nlm.nih.gov/entrez/viewer.fcgi?val=CY049957) | 1410 | Dominican Republic | 02-jul |
| 700 | [GQ426223](http://www.ncbi.nlm.nih.gov/entrez/viewer.fcgi?val=GQ426223) | 1034 | Italy | 25-jun | 564 | [CY051217](http://www.ncbi.nlm.nih.gov/entrez/viewer.fcgi?val=CY051217) | 1417 | USA | 30-jun |
| 701 | [CY044147](http://www.ncbi.nlm.nih.gov/entrez/viewer.fcgi?val=CY044147) | 1701 | Colombia | 25-jun | 565 | [CY052204](http://www.ncbi.nlm.nih.gov/entrez/viewer.fcgi?val=CY052204) | 1420 | USA | 30-jun |
| 702 | [GU014788](http://www.ncbi.nlm.nih.gov/entrez/viewer.fcgi?val=GU014788) | 1701 | Japan | 25-jun | 566 | [CY053963](http://www.ncbi.nlm.nih.gov/entrez/viewer.fcgi?val=CY053963) | 1430 | Argentina | 30-jun |
| 703 | [CY049100](http://www.ncbi.nlm.nih.gov/entrez/viewer.fcgi?val=CY049100) | 1741 | Singapore | 25-jun | 567 | [GQ866936](http://www.ncbi.nlm.nih.gov/entrez/viewer.fcgi?val=GQ866936) | 1417 | Thailand | Jul |
| 704 | [CY049148](http://www.ncbi.nlm.nih.gov/entrez/viewer.fcgi?val=CY049148) | 1741 | Singapore | 25-jun | 568 | [GQ866937](http://www.ncbi.nlm.nih.gov/entrez/viewer.fcgi?val=GQ866937) | 1397 | Thailand | Jul |
| 705 | [CY049156](http://www.ncbi.nlm.nih.gov/entrez/viewer.fcgi?val=CY049156) | 1739 | Singapore | 25-jun | 569 | [CY047746](http://www.ncbi.nlm.nih.gov/entrez/viewer.fcgi?val=CY047746) | 1438 | Taiwan | Jul |
| 706 | [CY049316](http://www.ncbi.nlm.nih.gov/entrez/viewer.fcgi?val=CY049316) | 1742 | Singapore | 25-jun | 570 | [GU123901](http://www.ncbi.nlm.nih.gov/entrez/viewer.fcgi?val=GU123901) | 1410 | Italy | Jul |
| 707 | [CY049380](http://www.ncbi.nlm.nih.gov/entrez/viewer.fcgi?val=CY049380) | 1741 | Singapore | 25-jun | 571 | [GU123902](http://www.ncbi.nlm.nih.gov/entrez/viewer.fcgi?val=GU123902) | 1347 | Italy | Jul |
| 708 | [CY049388](http://www.ncbi.nlm.nih.gov/entrez/viewer.fcgi?val=CY049388) | 1742 | Singapore | 25-jun | 572 | [GU123903](http://www.ncbi.nlm.nih.gov/entrez/viewer.fcgi?val=GU123903) | 1373 | Italy | Jul |
| 709 | [CY049939](http://www.ncbi.nlm.nih.gov/entrez/viewer.fcgi?val=CY049939) | 1698 | Dominican Republic | 25-jun | 573 | [GU123904](http://www.ncbi.nlm.nih.gov/entrez/viewer.fcgi?val=GU123904) | 1404 | Italy | Jul |
| 710 | [CY049979](http://www.ncbi.nlm.nih.gov/entrez/viewer.fcgi?val=CY049979) | 1695 | Dominican Republic | 25-jun | 574 | [GU123905](http://www.ncbi.nlm.nih.gov/entrez/viewer.fcgi?val=GU123905) | 1402 | Italy | Jul |
| 711 | [CY050182](http://www.ncbi.nlm.nih.gov/entrez/viewer.fcgi?val=CY050182) | 1733 | USA | 25-jun | 575 | [GU123906](http://www.ncbi.nlm.nih.gov/entrez/viewer.fcgi?val=GU123906) | 1301 | Italy | Jul |
| 712 | [CY052154](http://www.ncbi.nlm.nih.gov/entrez/viewer.fcgi?val=CY052154) | 1734 | USA | 25-jun | 576 | [GU123907](http://www.ncbi.nlm.nih.gov/entrez/viewer.fcgi?val=GU123907) | 1400 | Italy | Jul |
| 713 | [GQ355304](http://www.ncbi.nlm.nih.gov/entrez/viewer.fcgi?val=GQ355304) | 1553 | Spain | 26-jun | 577 | [GU123909](http://www.ncbi.nlm.nih.gov/entrez/viewer.fcgi?val=GU123909) | 1409 | Italy | Jul |
| 714 | [GQ355305](http://www.ncbi.nlm.nih.gov/entrez/viewer.fcgi?val=GQ355305) | 1518 | Spain | 26-jun | 578 | [GU123910](http://www.ncbi.nlm.nih.gov/entrez/viewer.fcgi?val=GU123910) | 1409 | Italy | Jul |
| 715 | [CY046928](http://www.ncbi.nlm.nih.gov/entrez/viewer.fcgi?val=CY046928) | 923 | Chile | 26-jun | 579 | [GU123912](http://www.ncbi.nlm.nih.gov/entrez/viewer.fcgi?val=GU123912) | 1398 | Italy | Jul |
| 716 | [CY049164](http://www.ncbi.nlm.nih.gov/entrez/viewer.fcgi?val=CY049164) | 1739 | Singapore | 26-jun | 580 | [GU123913](http://www.ncbi.nlm.nih.gov/entrez/viewer.fcgi?val=GU123913) | 1337 | Italy | Jul |
| 717 | [CY049172](http://www.ncbi.nlm.nih.gov/entrez/viewer.fcgi?val=CY049172) | 1739 | Singapore | 26-jun | 581 | [GU123916](http://www.ncbi.nlm.nih.gov/entrez/viewer.fcgi?val=GU123916) | 1201 | Italy | Jul |
|  | [CY049180](http://www.ncbi.nlm.nih.gov/entrez/viewer.fcgi?val=CY049180) | 1739 | Singapore | 26-jun | 582 | [GU123917](http://www.ncbi.nlm.nih.gov/entrez/viewer.fcgi?val=GU123917) | 1217 | Italy | Jul |
|  | [CY049188](http://www.ncbi.nlm.nih.gov/entrez/viewer.fcgi?val=CY049188) | 1739 | Singapore | 26-jun | 583 | [GU123918](http://www.ncbi.nlm.nih.gov/entrez/viewer.fcgi?val=GU123918) | 1374 | Italy | Jul |
|  | [CY049435](http://www.ncbi.nlm.nih.gov/entrez/viewer.fcgi?val=CY049435) | 1739 | Singapore | 04-jul | 584 | [GU123919](http://www.ncbi.nlm.nih.gov/entrez/viewer.fcgi?val=GU123919) | 1410 | Italy | Jul |
|  | [CY049443](http://www.ncbi.nlm.nih.gov/entrez/viewer.fcgi?val=CY049443) | 1739 | Singapore | 04-jul | 585 | [GU123920](http://www.ncbi.nlm.nih.gov/entrez/viewer.fcgi?val=GU123920) | 1385 | Italy | Jul |
|  | [CY049260](http://www.ncbi.nlm.nih.gov/entrez/viewer.fcgi?val=CY049260) | 1739 | Singapore | 06-jul | 586 | [GU123921](http://www.ncbi.nlm.nih.gov/entrez/viewer.fcgi?val=GU123921) | 1396 | Italy | Jul |
|  | [CY049531](http://www.ncbi.nlm.nih.gov/entrez/viewer.fcgi?val=CY049531) | 1739 | Singapore | 10-jul | 587 | [GU123922](http://www.ncbi.nlm.nih.gov/entrez/viewer.fcgi?val=GU123922) | 1400 | Italy | Jul |
|  | [CY049587](http://www.ncbi.nlm.nih.gov/entrez/viewer.fcgi?val=CY049587) | 1739 | Singapore | 14-jul | 588 | [GU123923](http://www.ncbi.nlm.nih.gov/entrez/viewer.fcgi?val=GU123923) | 1391 | Italy | Jul |
|  | [CY049348](http://www.ncbi.nlm.nih.gov/entrez/viewer.fcgi?val=CY049348) | 1743 | Singapore | 26-jun | 589 | [GU123924](http://www.ncbi.nlm.nih.gov/entrez/viewer.fcgi?val=GU123924) | 1365 | Italy | Jul |
| 718 | [CY052114](http://www.ncbi.nlm.nih.gov/entrez/viewer.fcgi?val=CY052114) | 1734 | USA | 26-jun | 590 | [GU134712](http://www.ncbi.nlm.nih.gov/entrez/viewer.fcgi?val=GU134712) | 1341 | Italy | Jul |
| 719 | [CY049212](http://www.ncbi.nlm.nih.gov/entrez/viewer.fcgi?val=CY049212) | 1740 | Singapore | 27-jun | 591 | [GU134713](http://www.ncbi.nlm.nih.gov/entrez/viewer.fcgi?val=GU134713) | 1382 | Italy | Jul |
| 720 | [CY051207](http://www.ncbi.nlm.nih.gov/entrez/viewer.fcgi?val=CY051207) | 1734 | USA | 27-jun | 592 | [GU134714](http://www.ncbi.nlm.nih.gov/entrez/viewer.fcgi?val=GU134714) | 1399 | Italy | Jul |
| 721 | [CY053959](http://www.ncbi.nlm.nih.gov/entrez/viewer.fcgi?val=CY053959) | 1701 | Argentina | 27-jun | 593 | [GU134715](http://www.ncbi.nlm.nih.gov/entrez/viewer.fcgi?val=GU134715) | 1405 | Italy | Jul |
| 722 | [CY046952](http://www.ncbi.nlm.nih.gov/entrez/viewer.fcgi?val=CY046952) | 1744 | Norway | 28-jun | 594 | [GU134717](http://www.ncbi.nlm.nih.gov/entrez/viewer.fcgi?val=GU134717) | 1410 | Italy | Jul |
| 723 | [CY052186](http://www.ncbi.nlm.nih.gov/entrez/viewer.fcgi?val=CY052186) | 1733 | USA | 28-jun | 595 | [GU134719](http://www.ncbi.nlm.nih.gov/entrez/viewer.fcgi?val=GU134719) | 1285 | Italy | Jul |
| 724 | [GQ355306](http://www.ncbi.nlm.nih.gov/entrez/viewer.fcgi?val=GQ355306) | 1541 | Spain | 29-jun | 596 | [GU134722](http://www.ncbi.nlm.nih.gov/entrez/viewer.fcgi?val=GU134722) | 1363 | Italy | Jul |
| 725 | [GQ355307](http://www.ncbi.nlm.nih.gov/entrez/viewer.fcgi?val=GQ355307) | 1543 | Spain | 29-jun | 597 | [GU134723](http://www.ncbi.nlm.nih.gov/entrez/viewer.fcgi?val=GU134723) | 1288 | Italy | Jul |
| 726 | [GQ355308](http://www.ncbi.nlm.nih.gov/entrez/viewer.fcgi?val=GQ355308) | 1329 | Spain | 29-jun | 598 | [GU134724](http://www.ncbi.nlm.nih.gov/entrez/viewer.fcgi?val=GU134724) | 1405 | Italy | Jul |
| 727 | [GQ355309](http://www.ncbi.nlm.nih.gov/entrez/viewer.fcgi?val=GQ355309) | 1545 | Spain | 29-jun | 599 | [GU134725](http://www.ncbi.nlm.nih.gov/entrez/viewer.fcgi?val=GU134725) | 1302 | Italy | Jul |
| 728 | [GQ355310](http://www.ncbi.nlm.nih.gov/entrez/viewer.fcgi?val=GQ355310) | 1549 | Spain | 29-jun | 600 | [GU134726](http://www.ncbi.nlm.nih.gov/entrez/viewer.fcgi?val=GU134726) | 1360 | Italy | Jul |
| 729 | [CY043078](http://www.ncbi.nlm.nih.gov/entrez/viewer.fcgi?val=CY043078) | 1701 | Japan | 29-jun | 601 | [GU134727](http://www.ncbi.nlm.nih.gov/entrez/viewer.fcgi?val=GU134727) | 1400 | Italy | Jul |
| 730 | [GQ457519](http://www.ncbi.nlm.nih.gov/entrez/viewer.fcgi?val=GQ457519) | 1701 | China | 29-jun | 602 | [GU134728](http://www.ncbi.nlm.nih.gov/entrez/viewer.fcgi?val=GU134728) | 1369 | Italy | Jul |
| 731 | [CY052399](http://www.ncbi.nlm.nih.gov/entrez/viewer.fcgi?val=CY052399) | 1734 | USA | 29-jun | 603 | [GU134729](http://www.ncbi.nlm.nih.gov/entrez/viewer.fcgi?val=GU134729) | 1299 | Italy | Jul |
| 732 | [CY053907](http://www.ncbi.nlm.nih.gov/entrez/viewer.fcgi?val=CY053907) | 1704 | Argentina | 29-jun | 604 | [GU134730](http://www.ncbi.nlm.nih.gov/entrez/viewer.fcgi?val=GU134730) | 921 | Italy | Jul |
| 733 | [GQ374892](http://www.ncbi.nlm.nih.gov/entrez/viewer.fcgi?val=GQ374892) | 926 | Italy | 30-jun | 605 | [CY050365](http://www.ncbi.nlm.nih.gov/entrez/viewer.fcgi?val=CY050365) | 1363 | Singapore | Jul |
| 734 | [GQ392017](http://www.ncbi.nlm.nih.gov/entrez/viewer.fcgi?val=GQ392017) | 1721 | Singapore | 30-jun | 606 | [GQ379827](http://www.ncbi.nlm.nih.gov/entrez/viewer.fcgi?val=GQ379827) | 1235 | Spain | 01-jul |
| 735 | [CY046930](http://www.ncbi.nlm.nih.gov/entrez/viewer.fcgi?val=CY046930) | 998 | Chile | 30-jun | 607 | [GQ379828](http://www.ncbi.nlm.nih.gov/entrez/viewer.fcgi?val=GQ379828) | 1233 | Spain | 01-jul |
| 736 | [CY049963](http://www.ncbi.nlm.nih.gov/entrez/viewer.fcgi?val=CY049963) | 1698 | Dominican Republic | 30-jun | 608 | [GQ379829](http://www.ncbi.nlm.nih.gov/entrez/viewer.fcgi?val=GQ379829) | 1232 | Spain | 01-jul |
| 737 | [CY051487](http://www.ncbi.nlm.nih.gov/entrez/viewer.fcgi?val=CY051487) | 1734 | USA | 30-jun | 609 | [GQ379830](http://www.ncbi.nlm.nih.gov/entrez/viewer.fcgi?val=GQ379830) | 1233 | Spain | 01-jul |
| 738 | [CY052045](http://www.ncbi.nlm.nih.gov/entrez/viewer.fcgi?val=CY052045) | 1701 | Brazil | 30-jun | 610 | [GQ379831](http://www.ncbi.nlm.nih.gov/entrez/viewer.fcgi?val=GQ379831) | 1233 | Spain | 01-jul |
| 739 | [CY052202](http://www.ncbi.nlm.nih.gov/entrez/viewer.fcgi?val=CY052202) | 1734 | USA | 30-jun | 611 | [GQ379832](http://www.ncbi.nlm.nih.gov/entrez/viewer.fcgi?val=GQ379832) | 1234 | Spain | 01-jul |
| 740 | [CY053962](http://www.ncbi.nlm.nih.gov/entrez/viewer.fcgi?val=CY053962) | 1701 | Argentina | 30-jun | 612 | [GQ379833](http://www.ncbi.nlm.nih.gov/entrez/viewer.fcgi?val=GQ379833) | 1226 | Spain | 01-jul |
| 741 | [GQ496602](http://www.ncbi.nlm.nih.gov/entrez/viewer.fcgi?val=GQ496602) | 1314 | China | Jul | 613 | [GQ379834](http://www.ncbi.nlm.nih.gov/entrez/viewer.fcgi?val=GQ379834) | 1233 | Spain | 01-jul |
| 742 | [GQ866928](http://www.ncbi.nlm.nih.gov/entrez/viewer.fcgi?val=GQ866928) | 1741 | Thailand | Jul |  | [GQ379835](http://www.ncbi.nlm.nih.gov/entrez/viewer.fcgi?val=GQ379835) | 1233 | Spain | 01-jul |
| 743 | [GQ866929](http://www.ncbi.nlm.nih.gov/entrez/viewer.fcgi?val=GQ866929) | 1712 | Thailand | Jul | 614 | [GQ379838](http://www.ncbi.nlm.nih.gov/entrez/viewer.fcgi?val=GQ379838) | 1234 | Spain | 01-jul |
| 744 | [CY047744](http://www.ncbi.nlm.nih.gov/entrez/viewer.fcgi?val=CY047744) | 1744 | Taiwan | Jul | 615 | [GQ421202](http://www.ncbi.nlm.nih.gov/entrez/viewer.fcgi?val=GQ421202) | 1410 | Italy | 01-jul |
| 745 | [CY050363](http://www.ncbi.nlm.nih.gov/entrez/viewer.fcgi?val=CY050363) | 1701 | Singapore | Jul | 616 | [GQ465696](http://www.ncbi.nlm.nih.gov/entrez/viewer.fcgi?val=GQ465696) | 1422 | Canada | 01-jul |
| 746 | [CY052094](http://www.ncbi.nlm.nih.gov/entrez/viewer.fcgi?val=CY052094) | 1017 | Ecuador | Jul | 617 | [GQ387385](http://www.ncbi.nlm.nih.gov/entrez/viewer.fcgi?val=GQ387385) | 1231 | Spain | 01-jul |
| 747 | [CY052095](http://www.ncbi.nlm.nih.gov/entrez/viewer.fcgi?val=CY052095) | 1030 | Ecuador | Jul | 618 | [GQ387386](http://www.ncbi.nlm.nih.gov/entrez/viewer.fcgi?val=GQ387386) | 1233 | Spain | 01-jul |
| 748 | [CY052096](http://www.ncbi.nlm.nih.gov/entrez/viewer.fcgi?val=CY052096) | 956 | Ecuador | Jul | 619 | [GQ387387](http://www.ncbi.nlm.nih.gov/entrez/viewer.fcgi?val=GQ387387) | 1236 | Spain | 01-jul |
| 749 | [CY052097](http://www.ncbi.nlm.nih.gov/entrez/viewer.fcgi?val=CY052097) | 957 | Ecuador | Jul | 620 | [CY047111](http://www.ncbi.nlm.nih.gov/entrez/viewer.fcgi?val=CY047111) | 1229 | Spain | 01-jul |
| 750 | [GU369646](http://www.ncbi.nlm.nih.gov/entrez/viewer.fcgi?val=GU369646) | 1701 | Turkey | Jul | 621 | [GU065292](http://www.ncbi.nlm.nih.gov/entrez/viewer.fcgi?val=GU065292) | 1410 | Poland | 01-jul |
| 751 | [GU369648](http://www.ncbi.nlm.nih.gov/entrez/viewer.fcgi?val=GU369648) | 1701 | Turkey | Jul | 622 | [CY051569](http://www.ncbi.nlm.nih.gov/entrez/viewer.fcgi?val=CY051569) | 1420 | USA | 01-jul |
| 752 | [GU369653](http://www.ncbi.nlm.nih.gov/entrez/viewer.fcgi?val=GU369653) | 1356 | Turkey | Jul | 623 | [CY053914](http://www.ncbi.nlm.nih.gov/entrez/viewer.fcgi?val=CY053914) | 1218 | Argentina | 01-jul |
| 753 | [GU369659](http://www.ncbi.nlm.nih.gov/entrez/viewer.fcgi?val=GU369659) | 1647 | Turkey | Jul | 624 | [CY053920](http://www.ncbi.nlm.nih.gov/entrez/viewer.fcgi?val=CY053920) | 1412 | Argentina | 01-jul |
| 754 | [GQ421201](http://www.ncbi.nlm.nih.gov/entrez/viewer.fcgi?val=GQ421201) | 1701 | Italy | 01-jul | 625 | [GQ379840](http://www.ncbi.nlm.nih.gov/entrez/viewer.fcgi?val=GQ379840) | 1231 | Spain | 02-jul |
| 755 | [GU014782](http://www.ncbi.nlm.nih.gov/entrez/viewer.fcgi?val=GU014782) | 1701 | Japan | 01-jul | 626 | [GQ379842](http://www.ncbi.nlm.nih.gov/entrez/viewer.fcgi?val=GQ379842) | 1233 | Spain | 02-jul |
| 756 | [CY047110](http://www.ncbi.nlm.nih.gov/entrez/viewer.fcgi?val=CY047110) | 1537 | Spain | 01-jul |  | [GQ387394](http://www.ncbi.nlm.nih.gov/entrez/viewer.fcgi?val=GQ387394) | 1233 | Spain | 04-jul |
| 757 | [CY051567](http://www.ncbi.nlm.nih.gov/entrez/viewer.fcgi?val=CY051567) | 1734 | USA | 01-jul | 627 | [GQ379843](http://www.ncbi.nlm.nih.gov/entrez/viewer.fcgi?val=GQ379843) | 1231 | Spain | 02-jul |
| 758 | [CY051575](http://www.ncbi.nlm.nih.gov/entrez/viewer.fcgi?val=CY051575) | 1734 | USA | 01-jul | 628 | [GQ379844](http://www.ncbi.nlm.nih.gov/entrez/viewer.fcgi?val=GQ379844) | 1227 | Spain | 03-jul |
| 759 | [CY053919](http://www.ncbi.nlm.nih.gov/entrez/viewer.fcgi?val=CY053919) | 1701 | Argentina | 01-jul | 629 | [GQ379845](http://www.ncbi.nlm.nih.gov/entrez/viewer.fcgi?val=GQ379845) | 1235 | Spain | 03-jul |
| 760 | [CY049947](http://www.ncbi.nlm.nih.gov/entrez/viewer.fcgi?val=CY049947) | 1698 | Dominican Republic | 02-jul |  | [GQ379846](http://www.ncbi.nlm.nih.gov/entrez/viewer.fcgi?val=GQ379846) | 1235 | Spain | 03-jul |
| 761 | [CY049955](http://www.ncbi.nlm.nih.gov/entrez/viewer.fcgi?val=CY049955) | 1698 | Dominican Republic | 02-jul | 630 | [GQ379847](http://www.ncbi.nlm.nih.gov/entrez/viewer.fcgi?val=GQ379847) | 1226 | Spain | 03-jul |
| 762 | [CY052046](http://www.ncbi.nlm.nih.gov/entrez/viewer.fcgi?val=CY052046) | 1760 | Brazil | 02-jul | 631 | [GQ379848](http://www.ncbi.nlm.nih.gov/entrez/viewer.fcgi?val=GQ379848) | 1234 | Spain | 03-jul |
| 763 | [GQ414768](http://www.ncbi.nlm.nih.gov/entrez/viewer.fcgi?val=GQ414768) | 1701 | Brazil | 03-jul | 632 | [GQ379849](http://www.ncbi.nlm.nih.gov/entrez/viewer.fcgi?val=GQ379849) | 1232 | Spain | 03-jul |
| 764 | [GQ502906](http://www.ncbi.nlm.nih.gov/entrez/viewer.fcgi?val=GQ502906) | 1761 | Canada | 03-jul | 633 | [GQ379850](http://www.ncbi.nlm.nih.gov/entrez/viewer.fcgi?val=GQ379850) | 1231 | Spain | 03-jul |
| 765 | [GU014786](http://www.ncbi.nlm.nih.gov/entrez/viewer.fcgi?val=GU014786) | 1701 | Japan | 03-jul |  | [GQ379854](http://www.ncbi.nlm.nih.gov/entrez/viewer.fcgi?val=GQ379854) | 1231 | Spain | 03-jul |
| 766 | [CY048994](http://www.ncbi.nlm.nih.gov/entrez/viewer.fcgi?val=CY048994) | 1560 | Spain | 03-jul | 634 | [GQ379851](http://www.ncbi.nlm.nih.gov/entrez/viewer.fcgi?val=GQ379851) | 1232 | Spain | 03-jul |
| 767 | [CY049244](http://www.ncbi.nlm.nih.gov/entrez/viewer.fcgi?val=CY049244) | 1740 | Singapore | 04-jul | 635 | [GQ379852](http://www.ncbi.nlm.nih.gov/entrez/viewer.fcgi?val=GQ379852) | 1232 | Spain | 03-jul |
| 768 | [CY049252](http://www.ncbi.nlm.nih.gov/entrez/viewer.fcgi?val=CY049252) | 1741 | Singapore | 04-jul | 636 | [GQ379853](http://www.ncbi.nlm.nih.gov/entrez/viewer.fcgi?val=GQ379853) | 1231 | Spain | 03-jul |
| 769 | [CY049419](http://www.ncbi.nlm.nih.gov/entrez/viewer.fcgi?val=CY049419) | 1739 | Singapore | 04-jul | 637 | [GQ379855](http://www.ncbi.nlm.nih.gov/entrez/viewer.fcgi?val=GQ379855) | 1229 | Spain | 03-jul |
| 770 | [CY049427](http://www.ncbi.nlm.nih.gov/entrez/viewer.fcgi?val=CY049427) | 1738 | Singapore | 04-jul | 638 | [GQ379856](http://www.ncbi.nlm.nih.gov/entrez/viewer.fcgi?val=GQ379856) | 1233 | Spain | 03-jul |
| 771 | [AB530161](http://www.ncbi.nlm.nih.gov/entrez/viewer.fcgi?val=AB530161) | 1701 | Japan | 05-jul | 639 | [GQ379857](http://www.ncbi.nlm.nih.gov/entrez/viewer.fcgi?val=GQ379857) | 1232 | Spain | 03-jul |
| 772 | [CY049116](http://www.ncbi.nlm.nih.gov/entrez/viewer.fcgi?val=CY049116) | 1743 | Singapore | 05-jul |  | [GQ379861](http://www.ncbi.nlm.nih.gov/entrez/viewer.fcgi?val=GQ379861) | 1232 | Spain | 03-jul |
| 773 | [CY049459](http://www.ncbi.nlm.nih.gov/entrez/viewer.fcgi?val=CY049459) | 1740 | Singapore | 05-jul |  | [GQ387399](http://www.ncbi.nlm.nih.gov/entrez/viewer.fcgi?val=GQ387399) | 1232 | Spain | 06-jul |
| 774 | [GQ422377](http://www.ncbi.nlm.nih.gov/entrez/viewer.fcgi?val=GQ422377) | 947 | Italy | 06-jul |  | [GQ387404](http://www.ncbi.nlm.nih.gov/entrez/viewer.fcgi?val=GQ387404) | 1232 | Spain | 07-jul |
| 775 | [CY045511](http://www.ncbi.nlm.nih.gov/entrez/viewer.fcgi?val=CY045511) | 1766 | Canada | 06-jul | 640 | [GQ379858](http://www.ncbi.nlm.nih.gov/entrez/viewer.fcgi?val=GQ379858) | 1236 | Spain | 03-jul |
| 776 | [CY049268](http://www.ncbi.nlm.nih.gov/entrez/viewer.fcgi?val=CY049268) | 1741 | Singapore | 06-jul | 641 | [GQ379859](http://www.ncbi.nlm.nih.gov/entrez/viewer.fcgi?val=GQ379859) | 1236 | Spain | 03-jul |
| 777 | [CY049276](http://www.ncbi.nlm.nih.gov/entrez/viewer.fcgi?val=CY049276) | 1743 | Singapore | 06-jul | 642 | [GQ379860](http://www.ncbi.nlm.nih.gov/entrez/viewer.fcgi?val=GQ379860) | 1237 | Spain | 03-jul |
| 778 | [CY049284](http://www.ncbi.nlm.nih.gov/entrez/viewer.fcgi?val=CY049284) | 1741 | Singapore | 06-jul | 643 | [GQ379862](http://www.ncbi.nlm.nih.gov/entrez/viewer.fcgi?val=GQ379862) | 1231 | Spain | 03-jul |
| 779 | [CY045954](http://www.ncbi.nlm.nih.gov/entrez/viewer.fcgi?val=CY045954) | 1765 | Canada | 07-jul | 644 | [GQ502908](http://www.ncbi.nlm.nih.gov/entrez/viewer.fcgi?val=GQ502908) | 1458 | Canada | 03-jul |
| 780 | [CY046934](http://www.ncbi.nlm.nih.gov/entrez/viewer.fcgi?val=CY046934) | 928 | Chile | 07-jul | 645 | [CY048995](http://www.ncbi.nlm.nih.gov/entrez/viewer.fcgi?val=CY048995) | 1256 | Spain | 03-jul |
| 781 | [CY046936](http://www.ncbi.nlm.nih.gov/entrez/viewer.fcgi?val=CY046936) | 872 | Chile | 07-jul | 646 | [GQ387388](http://www.ncbi.nlm.nih.gov/entrez/viewer.fcgi?val=GQ387388) | 1236 | Spain | 04-jul |
| 782 | [CY046938](http://www.ncbi.nlm.nih.gov/entrez/viewer.fcgi?val=CY046938) | 1059 | Chile | 07-jul | 647 | [GQ387389](http://www.ncbi.nlm.nih.gov/entrez/viewer.fcgi?val=GQ387389) | 932 | Spain | 04-jul |
| 783 | [GU014748](http://www.ncbi.nlm.nih.gov/entrez/viewer.fcgi?val=GU014748) | 1701 | Japan | 07-jul | 648 | [GQ387390](http://www.ncbi.nlm.nih.gov/entrez/viewer.fcgi?val=GQ387390) | 1234 | Spain | 04-jul |
| 784 | [GU014766](http://www.ncbi.nlm.nih.gov/entrez/viewer.fcgi?val=GU014766) | 1701 | Japan | 07-jul | 649 | [GQ387391](http://www.ncbi.nlm.nih.gov/entrez/viewer.fcgi?val=GQ387391) | 1233 | Spain | 04-jul |
| 785 | [CY047090](http://www.ncbi.nlm.nih.gov/entrez/viewer.fcgi?val=CY047090) | 1558 | Spain | 07-jul | 650 | [GQ387392](http://www.ncbi.nlm.nih.gov/entrez/viewer.fcgi?val=GQ387392) | 1230 | Spain | 04-jul |
| 786 | [GQ422380](http://www.ncbi.nlm.nih.gov/entrez/viewer.fcgi?val=GQ422380) | 950 | Italy | 08-jul | 651 | [GQ387393](http://www.ncbi.nlm.nih.gov/entrez/viewer.fcgi?val=GQ387393) | 1234 | Spain | 04-jul |
| 787 | [GU014756](http://www.ncbi.nlm.nih.gov/entrez/viewer.fcgi?val=GU014756) | 1701 | Myanmar | 08-jul | 652 | [GU112751](http://www.ncbi.nlm.nih.gov/entrez/viewer.fcgi?val=GU112751) | 1410 | Poland | 04-jul |
| 788 | [CY050190](http://www.ncbi.nlm.nih.gov/entrez/viewer.fcgi?val=CY050190) | 1733 | USA | 08-jul |  | [GU112752](http://www.ncbi.nlm.nih.gov/entrez/viewer.fcgi?val=GU112752) | 1410 | Poland | 09-jul |
| 789 | [CY051591](http://www.ncbi.nlm.nih.gov/entrez/viewer.fcgi?val=CY051591) | 1734 | USA | 08-jul | 653 | [CY049230](http://www.ncbi.nlm.nih.gov/entrez/viewer.fcgi?val=CY049230) | 1399 | Singapore | 04-jul |
| 790 | [CY051599](http://www.ncbi.nlm.nih.gov/entrez/viewer.fcgi?val=CY051599) | 1734 | USA | 08-jul |  | [CY049565](http://www.ncbi.nlm.nih.gov/entrez/viewer.fcgi?val=CY049565) | 1399 | Singapore | 13-jul |
| 791 | [CY051607](http://www.ncbi.nlm.nih.gov/entrez/viewer.fcgi?val=CY051607) | 1734 | USA | 08-jul | 654 | [CY049238](http://www.ncbi.nlm.nih.gov/entrez/viewer.fcgi?val=CY049238) | 1393 | Singapore | 04-jul |
| 792 | [CY049483](http://www.ncbi.nlm.nih.gov/entrez/viewer.fcgi?val=CY049483) | 1740 | Singapore | 09-jul | 655 | [CY049246](http://www.ncbi.nlm.nih.gov/entrez/viewer.fcgi?val=CY049246) | 1390 | Singapore | 04-jul |
| 793 | [CY049491](http://www.ncbi.nlm.nih.gov/entrez/viewer.fcgi?val=CY049491) | 1743 | Singapore | 09-jul | 656 | [CY049254](http://www.ncbi.nlm.nih.gov/entrez/viewer.fcgi?val=CY049254) | 1416 | Singapore | 04-jul |
| 794 | [CY049539](http://www.ncbi.nlm.nih.gov/entrez/viewer.fcgi?val=CY049539) | 1740 | Singapore | 09-jul | 657 | [CY049421](http://www.ncbi.nlm.nih.gov/entrez/viewer.fcgi?val=CY049421) | 1411 | Singapore | 04-jul |
| 795 | [CY051615](http://www.ncbi.nlm.nih.gov/entrez/viewer.fcgi?val=CY051615) | 1734 | USA | 09-jul | 658 | [CY049429](http://www.ncbi.nlm.nih.gov/entrez/viewer.fcgi?val=CY049429) | 1386 | Singapore | 04-jul |
| 796 | [CY049899](http://www.ncbi.nlm.nih.gov/entrez/viewer.fcgi?val=CY049899) | 1704 | Russia | 10-jul | 659 | [CY049445](http://www.ncbi.nlm.nih.gov/entrez/viewer.fcgi?val=CY049445) | 1388 | Singapore | 04-jul |
| 797 | [CY050075](http://www.ncbi.nlm.nih.gov/entrez/viewer.fcgi?val=CY050075) | 1701 | Japan | 10-jul | 660 | [GQ387397](http://www.ncbi.nlm.nih.gov/entrez/viewer.fcgi?val=GQ387397) | 1230 | Spain | 05-jul |
| 798 | [CY050083](http://www.ncbi.nlm.nih.gov/entrez/viewer.fcgi?val=CY050083) | 1701 | Taiwan | 10-jul |  | [CY047113](http://www.ncbi.nlm.nih.gov/entrez/viewer.fcgi?val=CY047113) | 1230 | Spain | 09-jul |
| 799 | [GU183799](http://www.ncbi.nlm.nih.gov/entrez/viewer.fcgi?val=GU183799) | 1784 | Thailand | 10-jul | 661 | [CY049118](http://www.ncbi.nlm.nih.gov/entrez/viewer.fcgi?val=CY049118) | 1424 | Singapore | 05-jul |
| 800 | [GU014752](http://www.ncbi.nlm.nih.gov/entrez/viewer.fcgi?val=GU014752) | 1701 | Japan | 11-jul | 662 | [CY049453](http://www.ncbi.nlm.nih.gov/entrez/viewer.fcgi?val=CY049453) | 1388 | Singapore | 05-jul |
| 801 | [GQ421203](http://www.ncbi.nlm.nih.gov/entrez/viewer.fcgi?val=GQ421203) | 1701 | Italy | 12-jul | 663 | [CY049461](http://www.ncbi.nlm.nih.gov/entrez/viewer.fcgi?val=CY049461) | 1412 | Singapore | 05-jul |
| 802 | [CY047105](http://www.ncbi.nlm.nih.gov/entrez/viewer.fcgi?val=CY047105) | 1524 | Spain | 12-jul | 664 | [GQ387398](http://www.ncbi.nlm.nih.gov/entrez/viewer.fcgi?val=GQ387398) | 1231 | Spain | 06-jul |
| 803 | [CY049467](http://www.ncbi.nlm.nih.gov/entrez/viewer.fcgi?val=CY049467) | 1741 | Singapore | 12-jul | 665 | [GQ387400](http://www.ncbi.nlm.nih.gov/entrez/viewer.fcgi?val=GQ387400) | 1231 | Spain | 06-jul |
| 804 | [CY049475](http://www.ncbi.nlm.nih.gov/entrez/viewer.fcgi?val=CY049475) | 1740 | Singapore | 12-jul | 666 | [GQ387402](http://www.ncbi.nlm.nih.gov/entrez/viewer.fcgi?val=GQ387402) | 1231 | Spain | 06-jul |
| 805 | [GU183807](http://www.ncbi.nlm.nih.gov/entrez/viewer.fcgi?val=GU183807) | 1784 | Thailand | 12-jul | 667 | [GQ387403](http://www.ncbi.nlm.nih.gov/entrez/viewer.fcgi?val=GQ387403) | 1234 | Spain | 06-jul |
| 806 | [CY052346](http://www.ncbi.nlm.nih.gov/entrez/viewer.fcgi?val=CY052346) | 1701 | Brazil | 12-jul | 668 | [GQ387406](http://www.ncbi.nlm.nih.gov/entrez/viewer.fcgi?val=GQ387406) | 1060 | Spain | 06-jul |
| 807 | [CY052423](http://www.ncbi.nlm.nih.gov/entrez/viewer.fcgi?val=CY052423) | 1733 | USA | 12-jul | 669 | [GQ387401](http://www.ncbi.nlm.nih.gov/entrez/viewer.fcgi?val=GQ387401) | 1232 | Spain | 06-jul |
| 808 | [GQ422378](http://www.ncbi.nlm.nih.gov/entrez/viewer.fcgi?val=GQ422378) | 937 | Italy | 13-jul | 670 | [CY045513](http://www.ncbi.nlm.nih.gov/entrez/viewer.fcgi?val=CY045513) | 1411 | Canada | 06-jul |
| 809 | [GQ422379](http://www.ncbi.nlm.nih.gov/entrez/viewer.fcgi?val=GQ422379) | 936 | Italy | 13-jul | 671 | [CY049262](http://www.ncbi.nlm.nih.gov/entrez/viewer.fcgi?val=CY049262) | 1399 | Singapore | 06-jul |
| 810 | [GU014770](http://www.ncbi.nlm.nih.gov/entrez/viewer.fcgi?val=GU014770) | 1701 | Japan | 13-jul | 672 | [CY049270](http://www.ncbi.nlm.nih.gov/entrez/viewer.fcgi?val=CY049270) | 1399 | Singapore | 06-jul |
| 811 | [CY049499](http://www.ncbi.nlm.nih.gov/entrez/viewer.fcgi?val=CY049499) | 1741 | Singapore | 13-jul | 673 | [CY049278](http://www.ncbi.nlm.nih.gov/entrez/viewer.fcgi?val=CY049278) | 1412 | Singapore | 06-jul |
| 812 | [CY049507](http://www.ncbi.nlm.nih.gov/entrez/viewer.fcgi?val=CY049507) | 1743 | Singapore | 13-jul | 674 | [CY049286](http://www.ncbi.nlm.nih.gov/entrez/viewer.fcgi?val=CY049286) | 1418 | Singapore | 06-jul |
| 813 | [CY049515](http://www.ncbi.nlm.nih.gov/entrez/viewer.fcgi?val=CY049515) | 1740 | Singapore | 13-jul | 675 | [GQ387405](http://www.ncbi.nlm.nih.gov/entrez/viewer.fcgi?val=GQ387405) | 1230 | Spain | 07-jul |
| 814 | [CY049555](http://www.ncbi.nlm.nih.gov/entrez/viewer.fcgi?val=CY049555) | 1740 | Singapore | 13-jul | 676 | [CY045956](http://www.ncbi.nlm.nih.gov/entrez/viewer.fcgi?val=CY045956) | 1443 | Canada | 07-jul |
| 815 | [CY049563](http://www.ncbi.nlm.nih.gov/entrez/viewer.fcgi?val=CY049563) | 1740 | Singapore | 13-jul | 677 | [GU014749](http://www.ncbi.nlm.nih.gov/entrez/viewer.fcgi?val=GU014749) | 1410 | Japan | 07-jul |
| 816 | [CY052407](http://www.ncbi.nlm.nih.gov/entrez/viewer.fcgi?val=CY052407) | 1734 | USA | 13-jul | 678 | [GU014767](http://www.ncbi.nlm.nih.gov/entrez/viewer.fcgi?val=GU014767) | 1410 | Japan | 07-jul |
| 817 | [GQ499336](http://www.ncbi.nlm.nih.gov/entrez/viewer.fcgi?val=GQ499336) | 1701 | USA | 14-jul | 679 | [CY047091](http://www.ncbi.nlm.nih.gov/entrez/viewer.fcgi?val=CY047091) | 1245 | Spain | 07-jul |
| 818 | [GU014758](http://www.ncbi.nlm.nih.gov/entrez/viewer.fcgi?val=GU014758) | 1701 | Myanmar | 14-jul | 680 | [GQ497278](http://www.ncbi.nlm.nih.gov/entrez/viewer.fcgi?val=GQ497278) | 1410 | Poland | 07-jul |
| 819 | [CY047118](http://www.ncbi.nlm.nih.gov/entrez/viewer.fcgi?val=CY047118) | 1438 | Spain | 14-jul | 681 | [GU014757](http://www.ncbi.nlm.nih.gov/entrez/viewer.fcgi?val=GU014757) | 1410 | Myanmar | 08-jul |
| 820 | [CY049571](http://www.ncbi.nlm.nih.gov/entrez/viewer.fcgi?val=CY049571) | 1743 | Singapore | 14-jul | 682 | [CY047093](http://www.ncbi.nlm.nih.gov/entrez/viewer.fcgi?val=CY047093) | 1240 | Spain | 08-jul |
| 821 | [CY049579](http://www.ncbi.nlm.nih.gov/entrez/viewer.fcgi?val=CY049579) | 1740 | Singapore | 14-jul | 683 | [CY047095](http://www.ncbi.nlm.nih.gov/entrez/viewer.fcgi?val=CY047095) | 1243 | Spain | 08-jul |
| 822 | [CY049595](http://www.ncbi.nlm.nih.gov/entrez/viewer.fcgi?val=CY049595) | 1740 | Singapore | 14-jul | 684 | [CY047097](http://www.ncbi.nlm.nih.gov/entrez/viewer.fcgi?val=CY047097) | 1233 | Spain | 08-jul |
| 823 | [CY051623](http://www.ncbi.nlm.nih.gov/entrez/viewer.fcgi?val=CY051623) | 1734 | USA | 14-jul | 685 | [CY047099](http://www.ncbi.nlm.nih.gov/entrez/viewer.fcgi?val=CY047099) | 1249 | Spain | 08-jul |
| 824 | [CY051631](http://www.ncbi.nlm.nih.gov/entrez/viewer.fcgi?val=CY051631) | 1734 | USA | 14-jul | 686 | [CY047101](http://www.ncbi.nlm.nih.gov/entrez/viewer.fcgi?val=CY047101) | 1209 | Spain | 08-jul |
| 825 | [GQ422381](http://www.ncbi.nlm.nih.gov/entrez/viewer.fcgi?val=GQ422381) | 943 | Italy | 15-jul | 687 | [CY047103](http://www.ncbi.nlm.nih.gov/entrez/viewer.fcgi?val=CY047103) | 1231 | Spain | 08-jul |
| 826 | [CY047123](http://www.ncbi.nlm.nih.gov/entrez/viewer.fcgi?val=CY047123) | 1558 | Spain | 15-jul | 688 | [CY050192](http://www.ncbi.nlm.nih.gov/entrez/viewer.fcgi?val=CY050192) | 1413 | USA | 08-jul |
| 827 | [CY047125](http://www.ncbi.nlm.nih.gov/entrez/viewer.fcgi?val=CY047125) | 1569 | Spain | 15-jul | 689 | [CY051593](http://www.ncbi.nlm.nih.gov/entrez/viewer.fcgi?val=CY051593) | 1420 | USA | 08-jul |
| 828 | [CY047128](http://www.ncbi.nlm.nih.gov/entrez/viewer.fcgi?val=CY047128) | 1570 | Spain | 15-jul | 690 | [CY051601](http://www.ncbi.nlm.nih.gov/entrez/viewer.fcgi?val=CY051601) | 1420 | USA | 08-jul |
| 829 | [CY049603](http://www.ncbi.nlm.nih.gov/entrez/viewer.fcgi?val=CY049603) | 1741 | Singapore | 15-jul | 691 | [CY051609](http://www.ncbi.nlm.nih.gov/entrez/viewer.fcgi?val=CY051609) | 1420 | USA | 08-jul |
| 830 | [CY049611](http://www.ncbi.nlm.nih.gov/entrez/viewer.fcgi?val=CY049611) | 1683 | Singapore | 15-jul | 692 | [GQ392023](http://www.ncbi.nlm.nih.gov/entrez/viewer.fcgi?val=GQ392023) | 1433 | Russia | 09-jul |
| 831 | [GU290055](http://www.ncbi.nlm.nih.gov/entrez/viewer.fcgi?val=GU290055) | 1743 | Czech Republic | 15-jul | 693 | [CY049493](http://www.ncbi.nlm.nih.gov/entrez/viewer.fcgi?val=CY049493) | 1391 | Singapore | 09-jul |
| 832 | [CY047131](http://www.ncbi.nlm.nih.gov/entrez/viewer.fcgi?val=CY047131) | 1551 | Spain | 16-jul | 694 | [CY049541](http://www.ncbi.nlm.nih.gov/entrez/viewer.fcgi?val=CY049541) | 1390 | Singapore | 09-jul |
| 833 | [CY047134](http://www.ncbi.nlm.nih.gov/entrez/viewer.fcgi?val=CY047134) | 1560 | Spain | 16-jul | 695 | [GQ497279](http://www.ncbi.nlm.nih.gov/entrez/viewer.fcgi?val=GQ497279) | 1410 | Poland | 10-jul |
| 834 | [GU183815](http://www.ncbi.nlm.nih.gov/entrez/viewer.fcgi?val=GU183815) | 1780 | Thailand | 16-jul | 696 | [CY047115](http://www.ncbi.nlm.nih.gov/entrez/viewer.fcgi?val=CY047115) | 1229 | Spain | 10-jul |
| 835 | [CY046064](http://www.ncbi.nlm.nih.gov/entrez/viewer.fcgi?val=CY046064) | 1701 | Italy | 17-jul | 697 | [CY047116](http://www.ncbi.nlm.nih.gov/entrez/viewer.fcgi?val=CY047116) | 1233 | Spain | 10-jul |
| 836 | [CY046065](http://www.ncbi.nlm.nih.gov/entrez/viewer.fcgi?val=CY046065) | 1701 | Italy | 17-jul | 698 | [CY049533](http://www.ncbi.nlm.nih.gov/entrez/viewer.fcgi?val=CY049533) | 1387 | Singapore | 10-jul |
| 837 | [GU014806](http://www.ncbi.nlm.nih.gov/entrez/viewer.fcgi?val=GU014806) | 1701 | Japan | 18-jul | 699 | [CY049901](http://www.ncbi.nlm.nih.gov/entrez/viewer.fcgi?val=CY049901) | 1409 | Russia | 10-jul |
| 838 | [CY052048](http://www.ncbi.nlm.nih.gov/entrez/viewer.fcgi?val=CY052048) | 1701 | Brazil | 18-jul | 700 | [GU183801](http://www.ncbi.nlm.nih.gov/entrez/viewer.fcgi?val=GU183801) | 1462 | Thailand | 10-jul |
| 839 | [GU433025](http://www.ncbi.nlm.nih.gov/entrez/viewer.fcgi?val=GU433025) | 1752 | Russia | 19-jul | 701 | [GU014753](http://www.ncbi.nlm.nih.gov/entrez/viewer.fcgi?val=GU014753) | 1410 | Japan | 11-jul |
| 840 | [CY047137](http://www.ncbi.nlm.nih.gov/entrez/viewer.fcgi?val=CY047137) | 1557 | Spain | 20-jul | 702 | [GU065290](http://www.ncbi.nlm.nih.gov/entrez/viewer.fcgi?val=GU065290) | 1410 | Poland | 11-jul |
| 841 | [CY047143](http://www.ncbi.nlm.nih.gov/entrez/viewer.fcgi?val=CY047143) | 1554 | Spain | 20-jul | 703 | [GQ421204](http://www.ncbi.nlm.nih.gov/entrez/viewer.fcgi?val=GQ421204) | 1410 | Italy | 12-jul |
| 842 | [CY047145](http://www.ncbi.nlm.nih.gov/entrez/viewer.fcgi?val=CY047145) | 1540 | Spain | 20-jul | 704 | [CY047106](http://www.ncbi.nlm.nih.gov/entrez/viewer.fcgi?val=CY047106) | 1254 | Spain | 12-jul |
| 843 | [CY052049](http://www.ncbi.nlm.nih.gov/entrez/viewer.fcgi?val=CY052049) | 1701 | Brazil | 20-jul | 705 | [CY049469](http://www.ncbi.nlm.nih.gov/entrez/viewer.fcgi?val=CY049469) | 1398 | Singapore | 12-jul |
| 844 | [CY052050](http://www.ncbi.nlm.nih.gov/entrez/viewer.fcgi?val=CY052050) | 1701 | Brazil | 20-jul | 706 | [CY049477](http://www.ncbi.nlm.nih.gov/entrez/viewer.fcgi?val=CY049477) | 1397 | Singapore | 12-jul |
| 845 | [CY053904](http://www.ncbi.nlm.nih.gov/entrez/viewer.fcgi?val=CY053904) | 1701 | Argentina | 20-jul | 707 | [CY049549](http://www.ncbi.nlm.nih.gov/entrez/viewer.fcgi?val=CY049549) | 1396 | Singapore | 12-jul |
| 846 | [CY046066](http://www.ncbi.nlm.nih.gov/entrez/viewer.fcgi?val=CY046066) | 1701 | Italy | 21-jul | 708 | [GU183809](http://www.ncbi.nlm.nih.gov/entrez/viewer.fcgi?val=GU183809) | 1414 | Thailand | 12-jul |
| 847 | [AB530461](http://www.ncbi.nlm.nih.gov/entrez/viewer.fcgi?val=AB530461) | 1701 | Japan | 23-jul | 709 | [CY052425](http://www.ncbi.nlm.nih.gov/entrez/viewer.fcgi?val=CY052425) | 1420 | USA | 12-jul |
|  | [CY047150](http://www.ncbi.nlm.nih.gov/entrez/viewer.fcgi?val=CY047150) | 1535 | Spain | 21-jul | 710 | [CY049501](http://www.ncbi.nlm.nih.gov/entrez/viewer.fcgi?val=CY049501) | 1400 | Singapore | 13-jul |
| 848 | [GQ496142](http://www.ncbi.nlm.nih.gov/entrez/viewer.fcgi?val=GQ496142) | 1752 | Russia | 22-jul | 711 | [CY049509](http://www.ncbi.nlm.nih.gov/entrez/viewer.fcgi?val=CY049509) | 1393 | Singapore | 13-jul |
| 849 | [GQ496149](http://www.ncbi.nlm.nih.gov/entrez/viewer.fcgi?val=GQ496149) | 1752 | Russia | 24-jul | 712 | [CY049525](http://www.ncbi.nlm.nih.gov/entrez/viewer.fcgi?val=CY049525) | 1397 | Singapore | 13-jul |
|  | [GU014760](http://www.ncbi.nlm.nih.gov/entrez/viewer.fcgi?val=GU014760) | 1701 | Myanmar | 22-jul | 713 | [CY049557](http://www.ncbi.nlm.nih.gov/entrez/viewer.fcgi?val=CY049557) | 1393 | Singapore | 13-jul |
| 850 | [GU014762](http://www.ncbi.nlm.nih.gov/entrez/viewer.fcgi?val=GU014762) | 1701 | Myanmar | 22-jul | 714 | [CY052409](http://www.ncbi.nlm.nih.gov/entrez/viewer.fcgi?val=CY052409) | 1420 | USA | 13-jul |
|  | [CY047140](http://www.ncbi.nlm.nih.gov/entrez/viewer.fcgi?val=CY047140) | 1536 | Spain | 22-jul | 715 | [GQ499337](http://www.ncbi.nlm.nih.gov/entrez/viewer.fcgi?val=GQ499337) | 1410 | USA | 14-jul |
| 851 | [AB530462](http://www.ncbi.nlm.nih.gov/entrez/viewer.fcgi?val=AB530462) | 1701 | Japan | 23-jul | 716 | [CY047119](http://www.ncbi.nlm.nih.gov/entrez/viewer.fcgi?val=CY047119) | 1244 | Spain | 14-jul |
| 852 | [AB530463](http://www.ncbi.nlm.nih.gov/entrez/viewer.fcgi?val=AB530463) | 1701 | Japan | 23-jul | 717 | [CY049573](http://www.ncbi.nlm.nih.gov/entrez/viewer.fcgi?val=CY049573) | 1399 | Singapore | 14-jul |
| 853 | [GU117765](http://www.ncbi.nlm.nih.gov/entrez/viewer.fcgi?val=GU117765) | 1701 | Japan | Jul | 718 | [CY049589](http://www.ncbi.nlm.nih.gov/entrez/viewer.fcgi?val=CY049589) | 1416 | Singapore | 14-jul |
|  | [AB530465](http://www.ncbi.nlm.nih.gov/entrez/viewer.fcgi?val=AB530465) | 1701 | Japan | 24-jul | 719 | [CY049597](http://www.ncbi.nlm.nih.gov/entrez/viewer.fcgi?val=CY049597) | 1414 | Singapore | 14-jul |
|  | [AB535738](http://www.ncbi.nlm.nih.gov/entrez/viewer.fcgi?val=AB535738) | 1701 | Japan | 29-jul | 720 | [CY051625](http://www.ncbi.nlm.nih.gov/entrez/viewer.fcgi?val=CY051625) | 1420 | USA | 14-jul |
|  | [AB535739](http://www.ncbi.nlm.nih.gov/entrez/viewer.fcgi?val=AB535739) | 1701 | Japan | 30-jul | 721 | [CY051633](http://www.ncbi.nlm.nih.gov/entrez/viewer.fcgi?val=CY051633) | 1420 | USA | 14-jul |
|  | [AB530474](http://www.ncbi.nlm.nih.gov/entrez/viewer.fcgi?val=AB530474) | 1701 | Japan | 30-jul | 722 | [CY052089](http://www.ncbi.nlm.nih.gov/entrez/viewer.fcgi?val=CY052089) | 1089 | Italy | 14-jul |
|  | [AB530477](http://www.ncbi.nlm.nih.gov/entrez/viewer.fcgi?val=AB530477) | 1701 | Japan | 3-Aug | 723 | [CY047121](http://www.ncbi.nlm.nih.gov/entrez/viewer.fcgi?val=CY047121) | 1245 | Spain | 15-jul |
|  | [AB530478](http://www.ncbi.nlm.nih.gov/entrez/viewer.fcgi?val=AB530478) | 1701 | Japan | 3-Aug | 724 | [CY047126](http://www.ncbi.nlm.nih.gov/entrez/viewer.fcgi?val=CY047126) | 1250 | Spain | 15-jul |
|  | [AB530479](http://www.ncbi.nlm.nih.gov/entrez/viewer.fcgi?val=AB530479) | 1701 | Japan | 3-Aug | 725 | [CY047129](http://www.ncbi.nlm.nih.gov/entrez/viewer.fcgi?val=CY047129) | 1251 | Spain | 15-jul |
|  | [AB530480](http://www.ncbi.nlm.nih.gov/entrez/viewer.fcgi?val=AB530480) | 1701 | Japan | 3-Aug | 726 | [CY049605](http://www.ncbi.nlm.nih.gov/entrez/viewer.fcgi?val=CY049605) | 1399 | Singapore | 15-jul |
|  | [AB530481](http://www.ncbi.nlm.nih.gov/entrez/viewer.fcgi?val=AB530481) | 1701 | Japan | 3-Aug | 727 | [CY049613](http://www.ncbi.nlm.nih.gov/entrez/viewer.fcgi?val=CY049613) | 1398 | Singapore | 15-jul |
|  | [AB530482](http://www.ncbi.nlm.nih.gov/entrez/viewer.fcgi?val=AB530482) | 1701 | Japan | 5-Aug | 728 | [GU290057](http://www.ncbi.nlm.nih.gov/entrez/viewer.fcgi?val=GU290057) | 1422 | Czech Republic | 15-jul |
|  | [AB535740](http://www.ncbi.nlm.nih.gov/entrez/viewer.fcgi?val=AB535740) | 1701 | Japan | 5-Aug | 729 | [CY047132](http://www.ncbi.nlm.nih.gov/entrez/viewer.fcgi?val=CY047132) | 1250 | Spain | 16-jul |
|  | [AB530483](http://www.ncbi.nlm.nih.gov/entrez/viewer.fcgi?val=AB530483) | 1701 | Japan | 10-Aug | 730 | [CY049717](http://www.ncbi.nlm.nih.gov/entrez/viewer.fcgi?val=CY049717) | 1250 | Spain | 13-oct |
|  | [AB530484](http://www.ncbi.nlm.nih.gov/entrez/viewer.fcgi?val=AB530484) | 1701 | Japan | 10-Aug | 731 | [CY047135](http://www.ncbi.nlm.nih.gov/entrez/viewer.fcgi?val=CY047135) | 1244 | Spain | 16-jul |
|  | [AB530485](http://www.ncbi.nlm.nih.gov/entrez/viewer.fcgi?val=AB530485) | 1701 | Japan | 17-Aug | 731 | [CY047075](http://www.ncbi.nlm.nih.gov/entrez/viewer.fcgi?val=CY047075) | 1244 | Spain | 29-jul |
|  | [AB530487](http://www.ncbi.nlm.nih.gov/entrez/viewer.fcgi?val=AB530487) | 1701 | Japan | 20-Aug | 732 | [GU183817](http://www.ncbi.nlm.nih.gov/entrez/viewer.fcgi?val=GU183817) | 1448 | Thailand | 16-jul |
|  | [CY052347](http://www.ncbi.nlm.nih.gov/entrez/viewer.fcgi?val=CY052347) | 1701 | Brazil | 23-jul | 733 | [GU014807](http://www.ncbi.nlm.nih.gov/entrez/viewer.fcgi?val=GU014807) | 1410 | Japan | 18-jul |
| 854 | [CY054279](http://www.ncbi.nlm.nih.gov/entrez/viewer.fcgi?val=CY054279) | 1701 | Brazil | 23-jul | 734 | [CY047138](http://www.ncbi.nlm.nih.gov/entrez/viewer.fcgi?val=CY047138) | 1256 | Spain | 20-jul |
| 855 | [CY047155](http://www.ncbi.nlm.nih.gov/entrez/viewer.fcgi?val=CY047155) | 1569 | Spain | 24-jul | 735 | [CY047144](http://www.ncbi.nlm.nih.gov/entrez/viewer.fcgi?val=CY047144) | 1255 | Spain | 20-jul |
| 856 | [AB530464](http://www.ncbi.nlm.nih.gov/entrez/viewer.fcgi?val=AB530464) | 1701 | Japan | 24-jul | 736 | [CY047146](http://www.ncbi.nlm.nih.gov/entrez/viewer.fcgi?val=CY047146) | 1220 | Spain | 20-jul |
| 857 | [AB530468](http://www.ncbi.nlm.nih.gov/entrez/viewer.fcgi?val=AB530468) | 1701 | Japan | 25-jul | 737 | [CY052377](http://www.ncbi.nlm.nih.gov/entrez/viewer.fcgi?val=CY052377) | 1420 | USA | 20-jul |
|  | [AB530471](http://www.ncbi.nlm.nih.gov/entrez/viewer.fcgi?val=AB530471) | 1701 | Japan | 29-jul | 738 | [CY052090](http://www.ncbi.nlm.nih.gov/entrez/viewer.fcgi?val=CY052090) | 1071 | Italy | 20-jul |
|  | [AB530475](http://www.ncbi.nlm.nih.gov/entrez/viewer.fcgi?val=AB530475) | 1701 | Japan | 30-jul | 739 | [CY053905](http://www.ncbi.nlm.nih.gov/entrez/viewer.fcgi?val=CY053905) | 1417 | Argentina | 20-jul |
|  | [AB535747](http://www.ncbi.nlm.nih.gov/entrez/viewer.fcgi?val=AB535747) | 1701 | Japan | 27-oct | 740 | [GU014755](http://www.ncbi.nlm.nih.gov/entrez/viewer.fcgi?val=GU014755) | 1410 | Japan | 21-jul |
|  | [AB530466](http://www.ncbi.nlm.nih.gov/entrez/viewer.fcgi?val=AB530466) | 1701 | Japan | 24-jul |  | [GU290191](http://www.ncbi.nlm.nih.gov/entrez/viewer.fcgi?val=GU290191) | 1410 | USA | 24-jul |
| 858 | [AB530467](http://www.ncbi.nlm.nih.gov/entrez/viewer.fcgi?val=AB530467) | 1701 | Japan | 24-jul | 741 | [CY047148](http://www.ncbi.nlm.nih.gov/entrez/viewer.fcgi?val=CY047148) | 1231 | Spain | 21-jul |
| 859 | [CY051655](http://www.ncbi.nlm.nih.gov/entrez/viewer.fcgi?val=CY051655) | 1734 | USA | 24-jul | 742 | [CY047151](http://www.ncbi.nlm.nih.gov/entrez/viewer.fcgi?val=CY047151) | 1230 | Spain | 21-jul |
| 860 | [CY051823](http://www.ncbi.nlm.nih.gov/entrez/viewer.fcgi?val=CY051823) | 1734 | USA | 24-jul | 743 | [GQ496143](http://www.ncbi.nlm.nih.gov/entrez/viewer.fcgi?val=GQ496143) | 1433 | Russia | 22-jul |
| 861 | [GU290188](http://www.ncbi.nlm.nih.gov/entrez/viewer.fcgi?val=GU290188) | 1701 | USA | 24-jul |  | [GQ496148](http://www.ncbi.nlm.nih.gov/entrez/viewer.fcgi?val=GQ496148) | 1433 | Russia | 24-jul |
| 862 | [GU290190](http://www.ncbi.nlm.nih.gov/entrez/viewer.fcgi?val=GU290190) | 1701 | USA | 24-jul | 744 | [CY047141](http://www.ncbi.nlm.nih.gov/entrez/viewer.fcgi?val=CY047141) | 1246 | Spain | 22-jul |
| 863 | [GU292341](http://www.ncbi.nlm.nih.gov/entrez/viewer.fcgi?val=GU292341) | 1717 | Finland | 24-jul | 745 | [CY047153](http://www.ncbi.nlm.nih.gov/entrez/viewer.fcgi?val=CY047153) | 1231 | Spain | 23-jul |
| 864 | [CY047071](http://www.ncbi.nlm.nih.gov/entrez/viewer.fcgi?val=CY047071) | 1554 | Spain | 26-jul | 746 | [CY047156](http://www.ncbi.nlm.nih.gov/entrez/viewer.fcgi?val=CY047156) | 1252 | Spain | 24-jul |
| 865 | [GU057010](http://www.ncbi.nlm.nih.gov/entrez/viewer.fcgi?val=GU057010) | 1701 | China | 26-jul | 747 | [CY051657](http://www.ncbi.nlm.nih.gov/entrez/viewer.fcgi?val=CY051657) | 1420 | USA | 24-jul |
| 866 | [GU057012](http://www.ncbi.nlm.nih.gov/entrez/viewer.fcgi?val=GU057012) | 1701 | China | 26-jul | 748 | [CY051665](http://www.ncbi.nlm.nih.gov/entrez/viewer.fcgi?val=CY051665) | 1420 | USA | 25-jul |
|  | [GU057013](http://www.ncbi.nlm.nih.gov/entrez/viewer.fcgi?val=GU057013) | 1701 | China | 26-jul | 749 | [CY047072](http://www.ncbi.nlm.nih.gov/entrez/viewer.fcgi?val=CY047072) | 1251 | Spain | 26-jul |
|  | [GU057015](http://www.ncbi.nlm.nih.gov/entrez/viewer.fcgi?val=GU057015) | 1701 | China | 26-jul | 750 | [GU071976](http://www.ncbi.nlm.nih.gov/entrez/viewer.fcgi?val=GU071976) | 1410 | Iran | 27-jul |
|  | [GU057016](http://www.ncbi.nlm.nih.gov/entrez/viewer.fcgi?val=GU057016) | 1701 | China | 26-jul | 751 | [GQ499335](http://www.ncbi.nlm.nih.gov/entrez/viewer.fcgi?val=GQ499335) | 1410 | USA | 28-jul |
|  | [GU057017](http://www.ncbi.nlm.nih.gov/entrez/viewer.fcgi?val=GU057017) | 1701 | China | 26-jul | 752 | [CY047159](http://www.ncbi.nlm.nih.gov/entrez/viewer.fcgi?val=CY047159) | 1243 | Spain | 28-jul |
|  | [GU057018](http://www.ncbi.nlm.nih.gov/entrez/viewer.fcgi?val=GU057018) | 1701 | China | 26-jul | 753 | [CY047162](http://www.ncbi.nlm.nih.gov/entrez/viewer.fcgi?val=CY047162) | 1227 | Spain | 28-jul |
|  | [GU057020](http://www.ncbi.nlm.nih.gov/entrez/viewer.fcgi?val=GU057020) | 1701 | China | 26-jul | 754 | [CY047168](http://www.ncbi.nlm.nih.gov/entrez/viewer.fcgi?val=CY047168) | 1252 | Spain | 28-jul |
|  | [GU057021](http://www.ncbi.nlm.nih.gov/entrez/viewer.fcgi?val=GU057021) | 1701 | China | 26-jul | 755 | [CY047171](http://www.ncbi.nlm.nih.gov/entrez/viewer.fcgi?val=CY047171) | 1242 | Spain | 28-jul |
|  | [GU057011](http://www.ncbi.nlm.nih.gov/entrez/viewer.fcgi?val=GU057011) | 1701 | China | 26-jul | 756 | [CY047174](http://www.ncbi.nlm.nih.gov/entrez/viewer.fcgi?val=CY047174) | 1246 | Spain | 28-jul |
| 867 | [GU057014](http://www.ncbi.nlm.nih.gov/entrez/viewer.fcgi?val=GU057014) | 1701 | China | 26-jul | 757 | [CY047186](http://www.ncbi.nlm.nih.gov/entrez/viewer.fcgi?val=CY047186) | 1245 | Spain | 28-jul |
| 868 | [GU057019](http://www.ncbi.nlm.nih.gov/entrez/viewer.fcgi?val=GU057019) | 1701 | China | 26-jul | 758 | [CY051673](http://www.ncbi.nlm.nih.gov/entrez/viewer.fcgi?val=CY051673) | 1420 | USA | 28-jul |
| 869 | [GQ915076](http://www.ncbi.nlm.nih.gov/entrez/viewer.fcgi?val=GQ915076) | 1701 | Singapore | 27-jul | 759 | [CY047183](http://www.ncbi.nlm.nih.gov/entrez/viewer.fcgi?val=CY047183) | 1245 | Spain | 29-jul |
| 870 | [AB530469](http://www.ncbi.nlm.nih.gov/entrez/viewer.fcgi?val=AB530469) | 1701 | Japan | 27-jul | 760 | [GU071975](http://www.ncbi.nlm.nih.gov/entrez/viewer.fcgi?val=GU071975) | 1410 | Iran | 29-jul |
| 871 | [AB530470](http://www.ncbi.nlm.nih.gov/entrez/viewer.fcgi?val=AB530470) | 1701 | Japan | 27-jul | 761 | [CY052091](http://www.ncbi.nlm.nih.gov/entrez/viewer.fcgi?val=CY052091) | 1071 | Italy | 29-jul |
| 872 | [CY051986](http://www.ncbi.nlm.nih.gov/entrez/viewer.fcgi?val=CY051986) | 1744 | Norway | 27-jul | 762 | [CY047165](http://www.ncbi.nlm.nih.gov/entrez/viewer.fcgi?val=CY047165) | 1240 | Spain | 30-jul |
| 873 | [CY051987](http://www.ncbi.nlm.nih.gov/entrez/viewer.fcgi?val=CY051987) | 1744 | Norway | 29-jul | 763 | [CY047177](http://www.ncbi.nlm.nih.gov/entrez/viewer.fcgi?val=CY047177) | 1245 | Spain | 30-jul |
|  | [GU226572](http://www.ncbi.nlm.nih.gov/entrez/viewer.fcgi?val=GU226572) | 1701 | Iran | 27-jul | 764 | [CY047180](http://www.ncbi.nlm.nih.gov/entrez/viewer.fcgi?val=CY047180) | 1251 | Spain | 30-jul |
| 874 | [CY052367](http://www.ncbi.nlm.nih.gov/entrez/viewer.fcgi?val=CY052367) | 1734 | USA | 27-jul | 765 | [CY047189](http://www.ncbi.nlm.nih.gov/entrez/viewer.fcgi?val=CY047189) | 1247 | Spain | 30-jul |
| 875 | [CY053922](http://www.ncbi.nlm.nih.gov/entrez/viewer.fcgi?val=CY053922) | 1702 | Argentina | 27-jul | 766 | [GU071974](http://www.ncbi.nlm.nih.gov/entrez/viewer.fcgi?val=GU071974) | 1410 | Iran | 30-jul |
| 876 | [GQ499334](http://www.ncbi.nlm.nih.gov/entrez/viewer.fcgi?val=GQ499334) | 1701 | USA | 28-jul | 767 | [CY047078](http://www.ncbi.nlm.nih.gov/entrez/viewer.fcgi?val=CY047078) | 824 | Spain | 31-jul |
| 877 | [CY047158](http://www.ncbi.nlm.nih.gov/entrez/viewer.fcgi?val=CY047158) | 1554 | Spain | 28-jul | 768 | [CY049621](http://www.ncbi.nlm.nih.gov/entrez/viewer.fcgi?val=CY049621) | 1381 | Singapore | 31-jul |
| 878 | [CY047161](http://www.ncbi.nlm.nih.gov/entrez/viewer.fcgi?val=CY047161) | 1559 | Spain | 28-jul | 769 | [CY049629](http://www.ncbi.nlm.nih.gov/entrez/viewer.fcgi?val=CY049629) | 1388 | Singapore | 31-jul |
| 879 | [CY047167](http://www.ncbi.nlm.nih.gov/entrez/viewer.fcgi?val=CY047167) | 1558 | Spain | 28-jul | 770 | [GQ866938](http://www.ncbi.nlm.nih.gov/entrez/viewer.fcgi?val=GQ866938) | 1371 | Thailand | Aug |
| 880 | [CY047170](http://www.ncbi.nlm.nih.gov/entrez/viewer.fcgi?val=CY047170) | 1563 | Spain | 28-jul | 771 | [GQ866939](http://www.ncbi.nlm.nih.gov/entrez/viewer.fcgi?val=GQ866939) | 1413 | Thailand | Aug |
| 881 | [CY047173](http://www.ncbi.nlm.nih.gov/entrez/viewer.fcgi?val=CY047173) | 1561 | Spain | 28-jul | 772 | [GU134731](http://www.ncbi.nlm.nih.gov/entrez/viewer.fcgi?val=GU134731) | 1365 | Italy | Aug |
| 882 | [CY047185](http://www.ncbi.nlm.nih.gov/entrez/viewer.fcgi?val=CY047185) | 1570 | Spain | 28-jul | 773 | [GU134732](http://www.ncbi.nlm.nih.gov/entrez/viewer.fcgi?val=GU134732) | 1331 | Italy | Aug |
| 883 | [CY051671](http://www.ncbi.nlm.nih.gov/entrez/viewer.fcgi?val=CY051671) | 1734 | USA | 28-jul | 774 | [GU134733](http://www.ncbi.nlm.nih.gov/entrez/viewer.fcgi?val=GU134733) | 1358 | Italy | Aug |
| 884 | [CY054280](http://www.ncbi.nlm.nih.gov/entrez/viewer.fcgi?val=CY054280) | 1701 | Brazil | 28-jul | 775 | [GU134734](http://www.ncbi.nlm.nih.gov/entrez/viewer.fcgi?val=GU134734) | 1353 | Italy | Aug |
| 885 | [CY047074](http://www.ncbi.nlm.nih.gov/entrez/viewer.fcgi?val=CY047074) | 1554 | Spain | 29-jul | 776 | [CY050341](http://www.ncbi.nlm.nih.gov/entrez/viewer.fcgi?val=CY050341) | 1398 | Singapore | Aug |
| 886 | [CY047182](http://www.ncbi.nlm.nih.gov/entrez/viewer.fcgi?val=CY047182) | 1560 | Spain | 29-jul | 777 | [CY050349](http://www.ncbi.nlm.nih.gov/entrez/viewer.fcgi?val=CY050349) | 1399 | Singapore | Aug |
| 887 | [GU226571](http://www.ncbi.nlm.nih.gov/entrez/viewer.fcgi?val=GU226571) | 1701 | Iran | 29-jul | 778 | [CY050357](http://www.ncbi.nlm.nih.gov/entrez/viewer.fcgi?val=CY050357) | 1399 | Singapore | Aug |
| 888 | [CY052282](http://www.ncbi.nlm.nih.gov/entrez/viewer.fcgi?val=CY052282) | 1734 | USA | 29-jul | 779 | [CY047081](http://www.ncbi.nlm.nih.gov/entrez/viewer.fcgi?val=CY047081) | 1245 | Spain | 3-Aug |
| 889 | [CY047164](http://www.ncbi.nlm.nih.gov/entrez/viewer.fcgi?val=CY047164) | 1558 | Spain | 30-jul | 780 | [CY047084](http://www.ncbi.nlm.nih.gov/entrez/viewer.fcgi?val=CY047084) | 1253 | Spain | 3-Aug |
| 890 | [CY049027](http://www.ncbi.nlm.nih.gov/entrez/viewer.fcgi?val=CY049027) | 1558 | Spain | 16-sep | 781 | [CY047087](http://www.ncbi.nlm.nih.gov/entrez/viewer.fcgi?val=CY047087) | 1225 | Spain | 4-Aug |
|  | [CY047176](http://www.ncbi.nlm.nih.gov/entrez/viewer.fcgi?val=CY047176) | 1561 | Spain | 30-jul | 782 | [CY047195](http://www.ncbi.nlm.nih.gov/entrez/viewer.fcgi?val=CY047195) | 1249 | Spain | 4-Aug |
| 891 | [CY047179](http://www.ncbi.nlm.nih.gov/entrez/viewer.fcgi?val=CY047179) | 1560 | Spain | 30-jul | 783 | [CY047192](http://www.ncbi.nlm.nih.gov/entrez/viewer.fcgi?val=CY047192) | 1246 | Spain | 5-Aug |
| 892 | [CY047188](http://www.ncbi.nlm.nih.gov/entrez/viewer.fcgi?val=CY047188) | 1439 | Spain | 30-jul | 784 | [CY047198](http://www.ncbi.nlm.nih.gov/entrez/viewer.fcgi?val=CY047198) | 1248 | Spain | 5-Aug |
| 893 | [AB530472](http://www.ncbi.nlm.nih.gov/entrez/viewer.fcgi?val=AB530472) | 1701 | Japan | 30-jul | 785 | [CY047204](http://www.ncbi.nlm.nih.gov/entrez/viewer.fcgi?val=CY047204) | 1245 | Spain | 5-Aug |
| 894 | [AB530473](http://www.ncbi.nlm.nih.gov/entrez/viewer.fcgi?val=AB530473) | 1701 | Japan | 30-jul | 785 | [CY049055](http://www.ncbi.nlm.nih.gov/entrez/viewer.fcgi?val=CY049055) | 1245 | Spain | 30-sep |
| 895 | [AB530476](http://www.ncbi.nlm.nih.gov/entrez/viewer.fcgi?val=AB530476) | 1701 | Japan | 30-jul | 786 | [CY047207](http://www.ncbi.nlm.nih.gov/entrez/viewer.fcgi?val=CY047207) | 1250 | Spain | 5-Aug |
| 896 | [CY051679](http://www.ncbi.nlm.nih.gov/entrez/viewer.fcgi?val=CY051679) | 1734 | USA | 30-jul | 787 | [CY047216](http://www.ncbi.nlm.nih.gov/entrez/viewer.fcgi?val=CY047216) | 1242 | Spain | 5-Aug |
| 897 | [GU226570](http://www.ncbi.nlm.nih.gov/entrez/viewer.fcgi?val=GU226570) | 1701 | Iran | 30-jul | 788 | [CY049637](http://www.ncbi.nlm.nih.gov/entrez/viewer.fcgi?val=CY049637) | 1414 | Singapore | 5-Aug |
| 898 | [CY047077](http://www.ncbi.nlm.nih.gov/entrez/viewer.fcgi?val=CY047077) | 1552 | Spain | 31-jul | 789 | [CY049645](http://www.ncbi.nlm.nih.gov/entrez/viewer.fcgi?val=CY049645) | 1383 | Singapore | 5-Aug |
| 899 | [CY049619](http://www.ncbi.nlm.nih.gov/entrez/viewer.fcgi?val=CY049619) | 1690 | Singapore | 31-jul | 790 | [CY047201](http://www.ncbi.nlm.nih.gov/entrez/viewer.fcgi?val=CY047201) | 1248 | Spain | 6-Aug |
| 900 | [CY049627](http://www.ncbi.nlm.nih.gov/entrez/viewer.fcgi?val=CY049627) | 1741 | Singapore | 31-jul | 791 | [CY047210](http://www.ncbi.nlm.nih.gov/entrez/viewer.fcgi?val=CY047210) | 1245 | Spain | 6-Aug |
| 901 | [GQ866930](http://www.ncbi.nlm.nih.gov/entrez/viewer.fcgi?val=GQ866930) | 1636 | Thailand | Aug |  | [CY047282](http://www.ncbi.nlm.nih.gov/entrez/viewer.fcgi?val=CY047282) | 1245 | Spain | 24-Aug |
| 902 | [GQ866931](http://www.ncbi.nlm.nih.gov/entrez/viewer.fcgi?val=GQ866931) | 1714 | Thailand | Aug |  | [CY048998](http://www.ncbi.nlm.nih.gov/entrez/viewer.fcgi?val=CY048998) | 1245 | Spain | 26-Aug |
| 903 | [CY050339](http://www.ncbi.nlm.nih.gov/entrez/viewer.fcgi?val=CY050339) | 1701 | Singapore | Aug |  | [CY049043](http://www.ncbi.nlm.nih.gov/entrez/viewer.fcgi?val=CY049043) | 1245 | Spain | 23-sep |
| 904 | [CY050347](http://www.ncbi.nlm.nih.gov/entrez/viewer.fcgi?val=CY050347) | 1701 | Singapore | Aug | 792 | [CY047213](http://www.ncbi.nlm.nih.gov/entrez/viewer.fcgi?val=CY047213) | 1250 | Spain | 6-Aug |
| 905 | [CY050355](http://www.ncbi.nlm.nih.gov/entrez/viewer.fcgi?val=CY050355) | 1701 | Singapore | Aug | 793 | [CY049661](http://www.ncbi.nlm.nih.gov/entrez/viewer.fcgi?val=CY049661) | 1390 | Singapore | 6-Aug |
| 906 | [CY052066](http://www.ncbi.nlm.nih.gov/entrez/viewer.fcgi?val=CY052066) | 827 | Belarus | Aug | 794 | [CY049669](http://www.ncbi.nlm.nih.gov/entrez/viewer.fcgi?val=CY049669) | 1400 | Singapore | 6-Aug |
| 907 | [CY052068](http://www.ncbi.nlm.nih.gov/entrez/viewer.fcgi?val=CY052068) | 950 | Belarus | Aug | 795 | [CY049677](http://www.ncbi.nlm.nih.gov/entrez/viewer.fcgi?val=CY049677) | 1387 | Singapore | 6-Aug |
| 908 | [GU369650](http://www.ncbi.nlm.nih.gov/entrez/viewer.fcgi?val=GU369650) | 1770 | Turkey | Aug | 796 | [CY049685](http://www.ncbi.nlm.nih.gov/entrez/viewer.fcgi?val=CY049685) | 1387 | Singapore | 6-Aug |
| 909 | [GU369652](http://www.ncbi.nlm.nih.gov/entrez/viewer.fcgi?val=GU369652) | 1783 | Turkey | Aug | 797 | [CY049693](http://www.ncbi.nlm.nih.gov/entrez/viewer.fcgi?val=CY049693) | 1388 | Singapore | 6-Aug |
| 910 | [GU369655](http://www.ncbi.nlm.nih.gov/entrez/viewer.fcgi?val=GU369655) | 1782 | Turkey | Aug | 798 | [CY049701](http://www.ncbi.nlm.nih.gov/entrez/viewer.fcgi?val=CY049701) | 1386 | Singapore | 6-Aug |
| 911 | [GU369657](http://www.ncbi.nlm.nih.gov/entrez/viewer.fcgi?val=GU369657) | 1748 | Turkey | Aug | 799 | [CY047219](http://www.ncbi.nlm.nih.gov/entrez/viewer.fcgi?val=CY047219) | 1241 | Spain | 7-Aug |
| 912 | [GU369661](http://www.ncbi.nlm.nih.gov/entrez/viewer.fcgi?val=GU369661) | 1600 | Turkey | Aug | 800 | [CY047228](http://www.ncbi.nlm.nih.gov/entrez/viewer.fcgi?val=CY047228) | 1246 | Spain | 10-Aug |
| 913 | [GU369662](http://www.ncbi.nlm.nih.gov/entrez/viewer.fcgi?val=GU369662) | 1776 | Turkey | Aug | 801 | [CY047231](http://www.ncbi.nlm.nih.gov/entrez/viewer.fcgi?val=CY047231) | 1251 | Spain | 10-Aug |
| 914 | [GU369666](http://www.ncbi.nlm.nih.gov/entrez/viewer.fcgi?val=GU369666) | 1782 | Turkey | Aug | 801 | [CY052041](http://www.ncbi.nlm.nih.gov/entrez/viewer.fcgi?val=CY052041) | 1251 | Spain | 16-nov |
| 915 | [GQ915018](http://www.ncbi.nlm.nih.gov/entrez/viewer.fcgi?val=GQ915018) | 1701 | Brazil | 1-Aug | 802 | [CY053786](http://www.ncbi.nlm.nih.gov/entrez/viewer.fcgi?val=CY053786) | 1428 | Norway | 10-Aug |
| 916 | [GQ915019](http://www.ncbi.nlm.nih.gov/entrez/viewer.fcgi?val=GQ915019) | 1701 | Brazil | 1-Aug | 803 | [CY052000](http://www.ncbi.nlm.nih.gov/entrez/viewer.fcgi?val=CY052000) | 1428 | Norway | 08-sep |
| 917 | [GQ915020](http://www.ncbi.nlm.nih.gov/entrez/viewer.fcgi?val=GQ915020) | 1701 | Brazil | 1-Aug | 804 | [CY047225](http://www.ncbi.nlm.nih.gov/entrez/viewer.fcgi?val=CY047225) | 1245 | Spain | 11-Aug |
| 918 | [GQ915022](http://www.ncbi.nlm.nih.gov/entrez/viewer.fcgi?val=GQ915022) | 1701 | Brazil | 1-Aug | 804 | [CY053380](http://www.ncbi.nlm.nih.gov/entrez/viewer.fcgi?val=CY053380) | 1245 | Spain | 23-nov |
| 919 | [GQ915021](http://www.ncbi.nlm.nih.gov/entrez/viewer.fcgi?val=GQ915021) | 1701 | Brazil | 2-Aug | 805 | [CY047222](http://www.ncbi.nlm.nih.gov/entrez/viewer.fcgi?val=CY047222) | 1244 | Spain | 12-Aug |
| 920 | [CY047080](http://www.ncbi.nlm.nih.gov/entrez/viewer.fcgi?val=CY047080) | 1219 | Spain | 3-Aug | 806 | [CY047233](http://www.ncbi.nlm.nih.gov/entrez/viewer.fcgi?val=CY047233) | 1254 | Spain | 13-Aug |
| 921 | [CY047083](http://www.ncbi.nlm.nih.gov/entrez/viewer.fcgi?val=CY047083) | 1479 | Spain | 3-Aug | 807 | [CY047236](http://www.ncbi.nlm.nih.gov/entrez/viewer.fcgi?val=CY047236) | 1253 | Spain | 13-Aug |
| 922 | [CY047194](http://www.ncbi.nlm.nih.gov/entrez/viewer.fcgi?val=CY047194) | 1546 | Spain | 4-Aug | 808 | [CY047238](http://www.ncbi.nlm.nih.gov/entrez/viewer.fcgi?val=CY047238) | 1250 | Spain | 13-Aug |
| 923 | [GQ915023](http://www.ncbi.nlm.nih.gov/entrez/viewer.fcgi?val=GQ915023) | 1701 | Brazil | 5-Aug | 809 | [CY047241](http://www.ncbi.nlm.nih.gov/entrez/viewer.fcgi?val=CY047241) | 1253 | Spain | 13-Aug |
| 924 | [GQ915024](http://www.ncbi.nlm.nih.gov/entrez/viewer.fcgi?val=GQ915024) | 1701 | Brazil | 5-Aug | 810 | [CY047244](http://www.ncbi.nlm.nih.gov/entrez/viewer.fcgi?val=CY047244) | 1250 | Spain | 13-Aug |
| 925 | [CY047191](http://www.ncbi.nlm.nih.gov/entrez/viewer.fcgi?val=CY047191) | 1546 | Spain | 5-Aug | 811 | [CY047249](http://www.ncbi.nlm.nih.gov/entrez/viewer.fcgi?val=CY047249) | 1244 | Spain | 14-Aug |
| 926 | [CY047197](http://www.ncbi.nlm.nih.gov/entrez/viewer.fcgi?val=CY047197) | 1546 | Spain | 5-Aug | 812 | [CY051697](http://www.ncbi.nlm.nih.gov/entrez/viewer.fcgi?val=CY051697) | 1421 | USA | 14-Aug |
| 927 | [CY047203](http://www.ncbi.nlm.nih.gov/entrez/viewer.fcgi?val=CY047203) | 1441 | Spain | 5-Aug | 813 | [CY052849](http://www.ncbi.nlm.nih.gov/entrez/viewer.fcgi?val=CY052849) | 1420 | USA | 15-Aug |
| 928 | [CY047206](http://www.ncbi.nlm.nih.gov/entrez/viewer.fcgi?val=CY047206) | 1511 | Spain | 5-Aug | 814 | [CY052873](http://www.ncbi.nlm.nih.gov/entrez/viewer.fcgi?val=CY052873) | 1420 | USA | 15-Aug |
| 929 | [CY047215](http://www.ncbi.nlm.nih.gov/entrez/viewer.fcgi?val=CY047215) | 1551 | Spain | 5-Aug | 815 | [CY047246](http://www.ncbi.nlm.nih.gov/entrez/viewer.fcgi?val=CY047246) | 1244 | Spain | 17-Aug |
| 930 | [CY049635](http://www.ncbi.nlm.nih.gov/entrez/viewer.fcgi?val=CY049635) | 1740 | Singapore | 5-Aug | 815 | [CY050317](http://www.ncbi.nlm.nih.gov/entrez/viewer.fcgi?val=CY050317) | 1244 | Spain | 28-oct |
| 931 | [CY049643](http://www.ncbi.nlm.nih.gov/entrez/viewer.fcgi?val=CY049643) | 1740 | Singapore | 5-Aug | 816 | [CY047252](http://www.ncbi.nlm.nih.gov/entrez/viewer.fcgi?val=CY047252) | 1254 | Spain | 17-Aug |
| 932 | [GU045582](http://www.ncbi.nlm.nih.gov/entrez/viewer.fcgi?val=GU045582) | 1701 | Singapore | 6-Aug | 817 | [CY047255](http://www.ncbi.nlm.nih.gov/entrez/viewer.fcgi?val=CY047255) | 1239 | Spain | 18-Aug |
| 933 | [CY047200](http://www.ncbi.nlm.nih.gov/entrez/viewer.fcgi?val=CY047200) | 1547 | Spain | 6-Aug | 818 | [CY047258](http://www.ncbi.nlm.nih.gov/entrez/viewer.fcgi?val=CY047258) | 1244 | Spain | 18-Aug |
| 934 | [CY047209](http://www.ncbi.nlm.nih.gov/entrez/viewer.fcgi?val=CY047209) | 1532 | Spain | 6-Aug | 819 | [CY051705](http://www.ncbi.nlm.nih.gov/entrez/viewer.fcgi?val=CY051705) | 1420 | USA | 18-Aug |
| 935 | [CY047212](http://www.ncbi.nlm.nih.gov/entrez/viewer.fcgi?val=CY047212) | 1210 | Spain | 6-Aug |  | [CY051721](http://www.ncbi.nlm.nih.gov/entrez/viewer.fcgi?val=CY051721) | 1420 | USA | 20-Aug |
| 936 | [CY049659](http://www.ncbi.nlm.nih.gov/entrez/viewer.fcgi?val=CY049659) | 1741 | Singapore | 6-Aug |  | [CY052164](http://www.ncbi.nlm.nih.gov/entrez/viewer.fcgi?val=CY052164) | 1420 | USA | 21-Aug |
| 937 | [CY049667](http://www.ncbi.nlm.nih.gov/entrez/viewer.fcgi?val=CY049667) | 1741 | Singapore | 6-Aug |  | [CY051713](http://www.ncbi.nlm.nih.gov/entrez/viewer.fcgi?val=CY051713) | 1420 | USA | 21-Aug |
| 938 | [CY049675](http://www.ncbi.nlm.nih.gov/entrez/viewer.fcgi?val=CY049675) | 1739 | Singapore | 6-Aug | 820 | [CY047261](http://www.ncbi.nlm.nih.gov/entrez/viewer.fcgi?val=CY047261) | 1246 | Spain | 19-Aug |
| 939 | [CY049683](http://www.ncbi.nlm.nih.gov/entrez/viewer.fcgi?val=CY049683) | 1740 | Singapore | 6-Aug | 821 | [CY047264](http://www.ncbi.nlm.nih.gov/entrez/viewer.fcgi?val=CY047264) | 1252 | Spain | 19-Aug |
| 940 | [CY049691](http://www.ncbi.nlm.nih.gov/entrez/viewer.fcgi?val=CY049691) | 1739 | Singapore | 6-Aug | 822 | [CY047267](http://www.ncbi.nlm.nih.gov/entrez/viewer.fcgi?val=CY047267) | 1253 | Spain | 19-Aug |
| 941 | [CY049699](http://www.ncbi.nlm.nih.gov/entrez/viewer.fcgi?val=CY049699) | 1740 | Singapore | 6-Aug | 823 | [CY047294](http://www.ncbi.nlm.nih.gov/entrez/viewer.fcgi?val=CY047294) | 1245 | Spain | 19-Aug |
| 942 | [CY051687](http://www.ncbi.nlm.nih.gov/entrez/viewer.fcgi?val=CY051687) | 1734 | USA | 6-Aug | 824 | [CY047285](http://www.ncbi.nlm.nih.gov/entrez/viewer.fcgi?val=CY047285) | 1246 | Spain | 21-Aug |
| 943 | [CY047218](http://www.ncbi.nlm.nih.gov/entrez/viewer.fcgi?val=CY047218) | 1541 | Spain | 7-Aug |  | [CY047302](http://www.ncbi.nlm.nih.gov/entrez/viewer.fcgi?val=CY047302) | 1246 | Spain | 25-Aug |
| 944 | [GU045583](http://www.ncbi.nlm.nih.gov/entrez/viewer.fcgi?val=GU045583) | 1701 | Singapore | 8-Aug |  | [CY047305](http://www.ncbi.nlm.nih.gov/entrez/viewer.fcgi?val=CY047305) | 1246 | Spain | 25-Aug |
| 945 | [GU045584](http://www.ncbi.nlm.nih.gov/entrez/viewer.fcgi?val=GU045584) | 1701 | Singapore | 8-Aug | 825 | [CY051729](http://www.ncbi.nlm.nih.gov/entrez/viewer.fcgi?val=CY051729) | 1420 | USA | 21-Aug |
| 946 | [CY047227](http://www.ncbi.nlm.nih.gov/entrez/viewer.fcgi?val=CY047227) | 1336 | Spain | 10-Aug |  | [CY051745](http://www.ncbi.nlm.nih.gov/entrez/viewer.fcgi?val=CY051745) | 1420 | USA | 21-Aug |
| 947 | [CY047230](http://www.ncbi.nlm.nih.gov/entrez/viewer.fcgi?val=CY047230) | 1511 | Spain | 10-Aug |  | [CY051737](http://www.ncbi.nlm.nih.gov/entrez/viewer.fcgi?val=CY051737) | 1420 | USA | 24-Aug |
| 948 | [CY051989](http://www.ncbi.nlm.nih.gov/entrez/viewer.fcgi?val=CY051989) | 1744 | Norway | 10-Aug | 826 | [CY047270](http://www.ncbi.nlm.nih.gov/entrez/viewer.fcgi?val=CY047270) | 1256 | Spain | 24-Aug |
| 949 | [CY054281](http://www.ncbi.nlm.nih.gov/entrez/viewer.fcgi?val=CY054281) | 1677 | Brazil | 10-Aug | 827 | [CY047273](http://www.ncbi.nlm.nih.gov/entrez/viewer.fcgi?val=CY047273) | 1244 | Spain | 24-Aug |
| 950 | [CY047224](http://www.ncbi.nlm.nih.gov/entrez/viewer.fcgi?val=CY047224) | 1561 | Spain | 11-Aug | 828 | [CY047276](http://www.ncbi.nlm.nih.gov/entrez/viewer.fcgi?val=CY047276) | 1253 | Spain | 24-Aug |
| 951 | [CY051988](http://www.ncbi.nlm.nih.gov/entrez/viewer.fcgi?val=CY051988) | 1744 | Norway | 11-Aug | 829 | [CY047279](http://www.ncbi.nlm.nih.gov/entrez/viewer.fcgi?val=CY047279) | 1247 | Spain | 24-Aug |
| 952 | [CY051991](http://www.ncbi.nlm.nih.gov/entrez/viewer.fcgi?val=CY051991) | 1744 | Norway | 11-Aug | 830 | [CY047297](http://www.ncbi.nlm.nih.gov/entrez/viewer.fcgi?val=CY047297) | 1246 | Spain | 24-Aug |
| 953 | [CY047221](http://www.ncbi.nlm.nih.gov/entrez/viewer.fcgi?val=CY047221) | 1531 | Spain | 12-Aug | 830 | [CY047299](http://www.ncbi.nlm.nih.gov/entrez/viewer.fcgi?val=CY047299) | 1246 | Spain | 24-Aug |
| 954 | [CY047235](http://www.ncbi.nlm.nih.gov/entrez/viewer.fcgi?val=CY047235) | 1560 | Spain | 13-Aug | 831 | [CY047288](http://www.ncbi.nlm.nih.gov/entrez/viewer.fcgi?val=CY047288) | 1245 | Spain | 25-Aug |
| 955 | [CY047284](http://www.ncbi.nlm.nih.gov/entrez/viewer.fcgi?val=CY047284) | 1560 | Spain | 21-Aug |  | [CY047291](http://www.ncbi.nlm.nih.gov/entrez/viewer.fcgi?val=CY047291) | 1245 | Spain | 25-Aug |
|  | [CY047240](http://www.ncbi.nlm.nih.gov/entrez/viewer.fcgi?val=CY047240) | 1517 | Spain | 13-Aug |  | [CY050308](http://www.ncbi.nlm.nih.gov/entrez/viewer.fcgi?val=CY050308) | 1245 | Spain | 26-oct |
| 956 | [CY047243](http://www.ncbi.nlm.nih.gov/entrez/viewer.fcgi?val=CY047243) | 1511 | Spain | 13-Aug | 832 | [CY047308](http://www.ncbi.nlm.nih.gov/entrez/viewer.fcgi?val=CY047308) | 1244 | Spain | 25-Aug |
| 957 | [CY047248](http://www.ncbi.nlm.nih.gov/entrez/viewer.fcgi?val=CY047248) | 1561 | Spain | 14-Aug | 833 | [CY049001](http://www.ncbi.nlm.nih.gov/entrez/viewer.fcgi?val=CY049001) | 1257 | Spain | 28-Aug |
| 958 | [CY051695](http://www.ncbi.nlm.nih.gov/entrez/viewer.fcgi?val=CY051695) | 1734 | USA | 14-Aug | 834 | [CY049004](http://www.ncbi.nlm.nih.gov/entrez/viewer.fcgi?val=CY049004) | 1253 | Spain | 28-Aug |
| 959 | [CY052847](http://www.ncbi.nlm.nih.gov/entrez/viewer.fcgi?val=CY052847) | 1734 | USA | 15-Aug | 835 | [CY049007](http://www.ncbi.nlm.nih.gov/entrez/viewer.fcgi?val=CY049007) | 1257 | Spain | 28-Aug |
| 960 | [CY052871](http://www.ncbi.nlm.nih.gov/entrez/viewer.fcgi?val=CY052871) | 1732 | USA | 15-Aug | 836 | [CY052769](http://www.ncbi.nlm.nih.gov/entrez/viewer.fcgi?val=CY052769) | 1420 | USA | 28-Aug |
| 961 | [GU057022](http://www.ncbi.nlm.nih.gov/entrez/viewer.fcgi?val=GU057022) | 1701 | China | 16-Aug | 837 | [CY052617](http://www.ncbi.nlm.nih.gov/entrez/viewer.fcgi?val=CY052617) | 1420 | USA | 30-Aug |
| 962 | [GU057024](http://www.ncbi.nlm.nih.gov/entrez/viewer.fcgi?val=GU057024) | 1701 | China | 16-Aug | 838 | [CY052625](http://www.ncbi.nlm.nih.gov/entrez/viewer.fcgi?val=CY052625) | 1420 | USA | 30-Aug |
|  | [GU057025](http://www.ncbi.nlm.nih.gov/entrez/viewer.fcgi?val=GU057025) | 1701 | China | 16-Aug |  | [CY052497](http://www.ncbi.nlm.nih.gov/entrez/viewer.fcgi?val=CY052497) | 1420 | USA | 11-sep |
|  | [GU057026](http://www.ncbi.nlm.nih.gov/entrez/viewer.fcgi?val=GU057026) | 1701 | China | 16-Aug | 839 | [CY052220](http://www.ncbi.nlm.nih.gov/entrez/viewer.fcgi?val=CY052220) | 1420 | USA | 30-Aug |
|  | [GU057023](http://www.ncbi.nlm.nih.gov/entrez/viewer.fcgi?val=GU057023) | 1701 | China | 16-Aug | 840 | [CY052228](http://www.ncbi.nlm.nih.gov/entrez/viewer.fcgi?val=CY052228) | 1420 | USA | 30-Aug |
| 963 | [GU057027](http://www.ncbi.nlm.nih.gov/entrez/viewer.fcgi?val=GU057027) | 1701 | China | 16-Aug |  | [CY052817](http://www.ncbi.nlm.nih.gov/entrez/viewer.fcgi?val=CY052817) | 1420 | USA | 07-sep |
| 964 | [GU057028](http://www.ncbi.nlm.nih.gov/entrez/viewer.fcgi?val=GU057028) | 1701 | China | 16-Aug |  | [CY052929](http://www.ncbi.nlm.nih.gov/entrez/viewer.fcgi?val=CY052929) | 1420 | USA | 12-sep |
| 965 | [GU057029](http://www.ncbi.nlm.nih.gov/entrez/viewer.fcgi?val=GU057029) | 1701 | China | 16-Aug |  | [CY052585](http://www.ncbi.nlm.nih.gov/entrez/viewer.fcgi?val=CY052585) | 1420 | USA | 13-sep |
|  | [GU057031](http://www.ncbi.nlm.nih.gov/entrez/viewer.fcgi?val=GU057031) | 1701 | China | 16-Aug | 841 | [CY049010](http://www.ncbi.nlm.nih.gov/entrez/viewer.fcgi?val=CY049010) | 1256 | Spain | 31-Aug |
|  | [GU057030](http://www.ncbi.nlm.nih.gov/entrez/viewer.fcgi?val=GU057030) | 1701 | China | 16-Aug | 842 | [CY052673](http://www.ncbi.nlm.nih.gov/entrez/viewer.fcgi?val=CY052673) | 1420 | USA | 31-Aug |
| 966 | [CY047251](http://www.ncbi.nlm.nih.gov/entrez/viewer.fcgi?val=CY047251) | 1556 | Spain | 17-Aug | 843 | [CY052340](http://www.ncbi.nlm.nih.gov/entrez/viewer.fcgi?val=CY052340) | 1420 | USA | 31-Aug |
| 967 | [AB530486](http://www.ncbi.nlm.nih.gov/entrez/viewer.fcgi?val=AB530486) | 1701 | Japan | 17-Aug |  | [CY052665](http://www.ncbi.nlm.nih.gov/entrez/viewer.fcgi?val=CY052665) | 1420 | USA | 09-sep |
| 968 | [CY047254](http://www.ncbi.nlm.nih.gov/entrez/viewer.fcgi?val=CY047254) | 1512 | Spain | 18-Aug | 844 | [CY049016](http://www.ncbi.nlm.nih.gov/entrez/viewer.fcgi?val=CY049016) | 1254 | Spain | 01-sep |
| 969 | [CY047257](http://www.ncbi.nlm.nih.gov/entrez/viewer.fcgi?val=CY047257) | 1536 | Spain | 18-Aug | 845 | [CY052021](http://www.ncbi.nlm.nih.gov/entrez/viewer.fcgi?val=CY052021) | 1428 | Norway | 01-sep |
| 970 | [CY051703](http://www.ncbi.nlm.nih.gov/entrez/viewer.fcgi?val=CY051703) | 1734 | USA | 18-Aug | 846 | [CY052681](http://www.ncbi.nlm.nih.gov/entrez/viewer.fcgi?val=CY052681) | 1412 | USA | 01-sep |
| 971 | [CY051719](http://www.ncbi.nlm.nih.gov/entrez/viewer.fcgi?val=CY051719) | 1734 | USA | 20-Aug | 847 | [CY051753](http://www.ncbi.nlm.nih.gov/entrez/viewer.fcgi?val=CY051753) | 1420 | USA | 02-sep |
|  | [CY052162](http://www.ncbi.nlm.nih.gov/entrez/viewer.fcgi?val=CY052162) | 1734 | USA | 21-Aug | 848 | [CY052777](http://www.ncbi.nlm.nih.gov/entrez/viewer.fcgi?val=CY052777) | 1420 | USA | 02-sep |
|  | [CY051711](http://www.ncbi.nlm.nih.gov/entrez/viewer.fcgi?val=CY051711) | 1734 | USA | 21-Aug | 849 | [CY052881](http://www.ncbi.nlm.nih.gov/entrez/viewer.fcgi?val=CY052881) | 1412 | USA | 02-sep |
|  | [CY047260](http://www.ncbi.nlm.nih.gov/entrez/viewer.fcgi?val=CY047260) | 1560 | Spain | 19-Aug | 850 | [CY049013](http://www.ncbi.nlm.nih.gov/entrez/viewer.fcgi?val=CY049013) | 1247 | Spain | 03-sep |
| 972 | [CY047263](http://www.ncbi.nlm.nih.gov/entrez/viewer.fcgi?val=CY047263) | 1560 | Spain | 19-Aug | 851 | [CY050005](http://www.ncbi.nlm.nih.gov/entrez/viewer.fcgi?val=CY050005) | 1410 | Mexico | 03-sep |
| 973 | [CY047266](http://www.ncbi.nlm.nih.gov/entrez/viewer.fcgi?val=CY047266) | 1559 | Spain | 19-Aug |  | [CY050037](http://www.ncbi.nlm.nih.gov/entrez/viewer.fcgi?val=CY050037) | 1410 | Mexico | 04-sep |
| 974 | [CY047293](http://www.ncbi.nlm.nih.gov/entrez/viewer.fcgi?val=CY047293) | 1544 | Spain | 19-Aug | 852 | [CY051761](http://www.ncbi.nlm.nih.gov/entrez/viewer.fcgi?val=CY051761) | 1420 | USA | 03-sep |
| 975 | [CY051990](http://www.ncbi.nlm.nih.gov/entrez/viewer.fcgi?val=CY051990) | 1744 | Norway | 19-Aug |  | [CY051769](http://www.ncbi.nlm.nih.gov/entrez/viewer.fcgi?val=CY051769) | 1420 | USA | 03-sep |
| 976 | [AB530488](http://www.ncbi.nlm.nih.gov/entrez/viewer.fcgi?val=AB530488) | 1701 | Japan | 20-Aug |  | [CY051777](http://www.ncbi.nlm.nih.gov/entrez/viewer.fcgi?val=CY051777) | 1420 | USA | 03-sep |
| 977 | [CY051727](http://www.ncbi.nlm.nih.gov/entrez/viewer.fcgi?val=CY051727) | 1734 | USA | 21-Aug |  | [CY051793](http://www.ncbi.nlm.nih.gov/entrez/viewer.fcgi?val=CY051793) | 1420 | USA | 09-sep |
| 978 | [CY051735](http://www.ncbi.nlm.nih.gov/entrez/viewer.fcgi?val=CY051735) | 1734 | USA | 24-Aug |  | [CY052180](http://www.ncbi.nlm.nih.gov/entrez/viewer.fcgi?val=CY052180) | 1420 | USA | 12-sep |
|  | [CY051743](http://www.ncbi.nlm.nih.gov/entrez/viewer.fcgi?val=CY051743) | 1732 | USA | 21-Aug | 853 | [CY052593](http://www.ncbi.nlm.nih.gov/entrez/viewer.fcgi?val=CY052593) | 1420 | USA | 03-sep |
| 979 | [CY047269](http://www.ncbi.nlm.nih.gov/entrez/viewer.fcgi?val=CY047269) | 1571 | Spain | 24-Aug | 854 | [CY052609](http://www.ncbi.nlm.nih.gov/entrez/viewer.fcgi?val=CY052609) | 1420 | USA | 03-sep |
| 980 | [CY047272](http://www.ncbi.nlm.nih.gov/entrez/viewer.fcgi?val=CY047272) | 1561 | Spain | 24-Aug |  | [CY052633](http://www.ncbi.nlm.nih.gov/entrez/viewer.fcgi?val=CY052633) | 1420 | USA | 06-sep |
| 981 | [CY047275](http://www.ncbi.nlm.nih.gov/entrez/viewer.fcgi?val=CY047275) | 1560 | Spain | 24-Aug |  | [CY052953](http://www.ncbi.nlm.nih.gov/entrez/viewer.fcgi?val=CY052953) | 1420 | USA | 06-sep |
| 982 | [CY047278](http://www.ncbi.nlm.nih.gov/entrez/viewer.fcgi?val=CY047278) | 1560 | Spain | 24-Aug | 855 | [CY052905](http://www.ncbi.nlm.nih.gov/entrez/viewer.fcgi?val=CY052905) | 1420 | USA | 03-sep |
| 983 | [CY047281](http://www.ncbi.nlm.nih.gov/entrez/viewer.fcgi?val=CY047281) | 1555 | Spain | 24-Aug | 856 | [CY052785](http://www.ncbi.nlm.nih.gov/entrez/viewer.fcgi?val=CY052785) | 1412 | USA | 04-sep |
| 984 | [CY047296](http://www.ncbi.nlm.nih.gov/entrez/viewer.fcgi?val=CY047296) | 1341 | Spain | 24-Aug | 857 | [CY052865](http://www.ncbi.nlm.nih.gov/entrez/viewer.fcgi?val=CY052865) | 1420 | USA | 04-sep |
| 985 | [CY047287](http://www.ncbi.nlm.nih.gov/entrez/viewer.fcgi?val=CY047287) | 1561 | Spain | 25-Aug |  | [CY052457](http://www.ncbi.nlm.nih.gov/entrez/viewer.fcgi?val=CY052457) | 1420 | USA | 12-sep |
| 986 | [CY047290](http://www.ncbi.nlm.nih.gov/entrez/viewer.fcgi?val=CY047290) | 1552 | Spain | 25-Aug |  | [CY052473](http://www.ncbi.nlm.nih.gov/entrez/viewer.fcgi?val=CY052473) | 1420 | USA | 12-sep |
| 987 | [CY047301](http://www.ncbi.nlm.nih.gov/entrez/viewer.fcgi?val=CY047301) | 1558 | Spain | 25-Aug | 858 | [CY052921](http://www.ncbi.nlm.nih.gov/entrez/viewer.fcgi?val=CY052921) | 1420 | USA | 05-sep |
| 988 | [CY047304](http://www.ncbi.nlm.nih.gov/entrez/viewer.fcgi?val=CY047304) | 1201 | Spain | 25-Aug | 859 | [GU108488](http://www.ncbi.nlm.nih.gov/entrez/viewer.fcgi?val=GU108488) | 1434 | China | 06-sep |
| 989 | [CY047307](http://www.ncbi.nlm.nih.gov/entrez/viewer.fcgi?val=CY047307) | 1546 | Spain | 25-Aug | 860 | [CY051785](http://www.ncbi.nlm.nih.gov/entrez/viewer.fcgi?val=CY051785) | 1420 | USA | 06-sep |
| 990 | [CY048997](http://www.ncbi.nlm.nih.gov/entrez/viewer.fcgi?val=CY048997) | 1560 | Spain | 26-Aug | 861 | [CY051897](http://www.ncbi.nlm.nih.gov/entrez/viewer.fcgi?val=CY051897) | 1420 | USA | 06-sep |
| 991 | [CY051993](http://www.ncbi.nlm.nih.gov/entrez/viewer.fcgi?val=CY051993) | 1745 | Norway | 27-Aug |  | [CY052649](http://www.ncbi.nlm.nih.gov/entrez/viewer.fcgi?val=CY052649) | 1420 | USA | 10-sep |
| 992 | [CY052004](http://www.ncbi.nlm.nih.gov/entrez/viewer.fcgi?val=CY052004) | 1744 | Norway | 27-Aug | 862 | [CY052641](http://www.ncbi.nlm.nih.gov/entrez/viewer.fcgi?val=CY052641) | 1420 | USA | 06-sep |
| 993 | [CY049000](http://www.ncbi.nlm.nih.gov/entrez/viewer.fcgi?val=CY049000) | 1561 | Spain | 28-Aug | 863 | [GU112092](http://www.ncbi.nlm.nih.gov/entrez/viewer.fcgi?val=GU112092) | 1410 | China | 07-sep |
| 994 | [CY049003](http://www.ncbi.nlm.nih.gov/entrez/viewer.fcgi?val=CY049003) | 1559 | Spain | 28-Aug |  | [GU189651](http://www.ncbi.nlm.nih.gov/entrez/viewer.fcgi?val=GU189651) | 1410 | China | 07-sep |
| 995 | [CY049006](http://www.ncbi.nlm.nih.gov/entrez/viewer.fcgi?val=CY049006) | 1560 | Spain | 28-Aug | 864 | [CY052689](http://www.ncbi.nlm.nih.gov/entrez/viewer.fcgi?val=CY052689) | 1420 | USA | 07-sep |
| 996 | [CY051992](http://www.ncbi.nlm.nih.gov/entrez/viewer.fcgi?val=CY051992) | 1744 | Norway | 28-Aug |  | [CY052529](http://www.ncbi.nlm.nih.gov/entrez/viewer.fcgi?val=CY052529) | 1420 | USA | 10-sep |
| 997 | [CY052767](http://www.ncbi.nlm.nih.gov/entrez/viewer.fcgi?val=CY052767) | 1734 | USA | 28-Aug | 865 | [CY052793](http://www.ncbi.nlm.nih.gov/entrez/viewer.fcgi?val=CY052793) | 1420 | USA | 07-sep |
| 998 | [CY052623](http://www.ncbi.nlm.nih.gov/entrez/viewer.fcgi?val=CY052623) | 1724 | USA | 30-Aug | 866 | [CY052809](http://www.ncbi.nlm.nih.gov/entrez/viewer.fcgi?val=CY052809) | 1420 | USA | 07-sep |
| 999 | [CY052218](http://www.ncbi.nlm.nih.gov/entrez/viewer.fcgi?val=CY052218) | 1734 | USA | 30-Aug | 867 | [CY052825](http://www.ncbi.nlm.nih.gov/entrez/viewer.fcgi?val=CY052825) | 1420 | USA | 07-sep |
| 1000 | [CY052226](http://www.ncbi.nlm.nih.gov/entrez/viewer.fcgi?val=CY052226) | 1734 | USA | 30-Aug |  | [CY052945](http://www.ncbi.nlm.nih.gov/entrez/viewer.fcgi?val=CY052945) | 1420 | USA | 12-sep |
| 1001 | [CY052815](http://www.ncbi.nlm.nih.gov/entrez/viewer.fcgi?val=CY052815) | 1734 | USA | 07-sep | 868 | [CY052697](http://www.ncbi.nlm.nih.gov/entrez/viewer.fcgi?val=CY052697) | 1420 | USA | 08-sep |
|  | [CY049009](http://www.ncbi.nlm.nih.gov/entrez/viewer.fcgi?val=CY049009) | 1557 | Spain | 31-Aug |  | [CY052505](http://www.ncbi.nlm.nih.gov/entrez/viewer.fcgi?val=CY052505) | 1420 | USA | 10-sep |
| 1002 | [CY051994](http://www.ncbi.nlm.nih.gov/entrez/viewer.fcgi?val=CY051994) | 1744 | Norway | 31-Aug |  | [CY052553](http://www.ncbi.nlm.nih.gov/entrez/viewer.fcgi?val=CY052553) | 1420 | USA | 12-sep |
| 1003 | [CY052671](http://www.ncbi.nlm.nih.gov/entrez/viewer.fcgi?val=CY052671) | 1734 | USA | 31-Aug | 869 | [CY052092](http://www.ncbi.nlm.nih.gov/entrez/viewer.fcgi?val=CY052092) | 1188 | Italy | 08-sep |
| 1004 | [CY052338](http://www.ncbi.nlm.nih.gov/entrez/viewer.fcgi?val=CY052338) | 1734 | USA | 31-Aug | 870 | [CY049019](http://www.ncbi.nlm.nih.gov/entrez/viewer.fcgi?val=CY049019) | 1258 | Spain | 09-sep |
| 1005 | [CY052679](http://www.ncbi.nlm.nih.gov/entrez/viewer.fcgi?val=CY052679) | 1734 | USA | 01-sep | 871 | [CY049022](http://www.ncbi.nlm.nih.gov/entrez/viewer.fcgi?val=CY049022) | 1253 | Spain | 09-sep |
|  | [CY052663](http://www.ncbi.nlm.nih.gov/entrez/viewer.fcgi?val=CY052663) | 1734 | USA | 09-sep | 872 | [CY050045](http://www.ncbi.nlm.nih.gov/entrez/viewer.fcgi?val=CY050045) | 1410 | Mexico | 09-sep |
|  | [AB540654](http://www.ncbi.nlm.nih.gov/entrez/viewer.fcgi?val=AB540654) | 1701 | Japan | 31-Aug | 873 | [CY050053](http://www.ncbi.nlm.nih.gov/entrez/viewer.fcgi?val=CY050053) | 1410 | Mexico | 10-sep |
| 1006 | [AB530489](http://www.ncbi.nlm.nih.gov/entrez/viewer.fcgi?val=AB530489) | 1701 | Japan | Sep |  | [CY050061](http://www.ncbi.nlm.nih.gov/entrez/viewer.fcgi?val=CY050061) | 1410 | Mexico | 10-sep |
| 1007 | [GU369654](http://www.ncbi.nlm.nih.gov/entrez/viewer.fcgi?val=GU369654) | 1782 | Turkey | Sep | 874 | [CY051801](http://www.ncbi.nlm.nih.gov/entrez/viewer.fcgi?val=CY051801) | 1420 | USA | 09-sep |
| 1008 | [GU369656](http://www.ncbi.nlm.nih.gov/entrez/viewer.fcgi?val=GU369656) | 1770 | Turkey | Sep | 875 | [CY052657](http://www.ncbi.nlm.nih.gov/entrez/viewer.fcgi?val=CY052657) | 1420 | USA | 09-sep |
| 1009 | [GU369660](http://www.ncbi.nlm.nih.gov/entrez/viewer.fcgi?val=GU369660) | 1781 | Turkey | Sep | 876 | [CY052705](http://www.ncbi.nlm.nih.gov/entrez/viewer.fcgi?val=CY052705) | 1420 | USA | 09-sep |
| 1010 | [CY049015](http://www.ncbi.nlm.nih.gov/entrez/viewer.fcgi?val=CY049015) | 1545 | Spain | 01-sep | 877 | [CY052721](http://www.ncbi.nlm.nih.gov/entrez/viewer.fcgi?val=CY052721) | 1420 | USA | 09-sep |
| 1011 | [CY052003](http://www.ncbi.nlm.nih.gov/entrez/viewer.fcgi?val=CY052003) | 1744 | Norway | 01-sep |  | [CY052753](http://www.ncbi.nlm.nih.gov/entrez/viewer.fcgi?val=CY052753) | 1420 | USA | 10-sep |
| #### | [CY052019](http://www.ncbi.nlm.nih.gov/entrez/viewer.fcgi?val=CY052019) | 1744 | Norway | 01-sep |  | [CY052761](http://www.ncbi.nlm.nih.gov/entrez/viewer.fcgi?val=CY052761) | 1420 | USA | 10-sep |
| #### | [CY050019](http://www.ncbi.nlm.nih.gov/entrez/viewer.fcgi?val=CY050019) | 1695 | Mexico | 02-sep |  | [CY052801](http://www.ncbi.nlm.nih.gov/entrez/viewer.fcgi?val=CY052801) | 1420 | USA | 13-sep |
| #### | [CY051751](http://www.ncbi.nlm.nih.gov/entrez/viewer.fcgi?val=CY051751) | 1734 | USA | 02-sep | 878 | [CY052729](http://www.ncbi.nlm.nih.gov/entrez/viewer.fcgi?val=CY052729) | 1420 | USA | 09-sep |
| #### | [CY052775](http://www.ncbi.nlm.nih.gov/entrez/viewer.fcgi?val=CY052775) | 1734 | USA | 02-sep |  | [CY052465](http://www.ncbi.nlm.nih.gov/entrez/viewer.fcgi?val=CY052465) | 1420 | USA | 12-sep |
| #### | [CY052855](http://www.ncbi.nlm.nih.gov/entrez/viewer.fcgi?val=CY052855) | 1734 | USA | 02-sep | 879 | [CY052737](http://www.ncbi.nlm.nih.gov/entrez/viewer.fcgi?val=CY052737) | 1420 | USA | 09-sep |
| #### | [CY052879](http://www.ncbi.nlm.nih.gov/entrez/viewer.fcgi?val=CY052879) | 1734 | USA | 02-sep | 880 | [CY052172](http://www.ncbi.nlm.nih.gov/entrez/viewer.fcgi?val=CY052172) | 1420 | USA | 09-sep |
| #### | [CY049012](http://www.ncbi.nlm.nih.gov/entrez/viewer.fcgi?val=CY049012) | 1560 | Spain | 03-sep | 881 | [CY052513](http://www.ncbi.nlm.nih.gov/entrez/viewer.fcgi?val=CY052513) | 1420 | USA | 10-sep |
| #### | [CY050003](http://www.ncbi.nlm.nih.gov/entrez/viewer.fcgi?val=CY050003) | 1698 | Mexico | 03-sep | 882 | [CY052521](http://www.ncbi.nlm.nih.gov/entrez/viewer.fcgi?val=CY052521) | 1420 | USA | 10-sep |
| 1020 | [CY050051](http://www.ncbi.nlm.nih.gov/entrez/viewer.fcgi?val=CY050051) | 1698 | Mexico | 10-sep | 883 | [CY052745](http://www.ncbi.nlm.nih.gov/entrez/viewer.fcgi?val=CY052745) | 1420 | USA | 10-sep |
|  | [CY051759](http://www.ncbi.nlm.nih.gov/entrez/viewer.fcgi?val=CY051759) | 1734 | USA | 03-sep | 884 | [CY052897](http://www.ncbi.nlm.nih.gov/entrez/viewer.fcgi?val=CY052897) | 1420 | USA | 10-sep |
| 1021 | [CY051775](http://www.ncbi.nlm.nih.gov/entrez/viewer.fcgi?val=CY051775) | 1734 | USA | 03-sep | 885 | [CY052537](http://www.ncbi.nlm.nih.gov/entrez/viewer.fcgi?val=CY052537) | 1420 | USA | 11-sep |
|  | [CY051791](http://www.ncbi.nlm.nih.gov/entrez/viewer.fcgi?val=CY051791) | 1734 | USA | 09-sep |  | [CY052433](http://www.ncbi.nlm.nih.gov/entrez/viewer.fcgi?val=CY052433) | 1420 | USA | 12-sep |
|  | [CY052170](http://www.ncbi.nlm.nih.gov/entrez/viewer.fcgi?val=CY052170) | 1734 | USA | 09-sep |  | [CY052481](http://www.ncbi.nlm.nih.gov/entrez/viewer.fcgi?val=CY052481) | 1420 | USA | 12-sep |
|  | [CY052178](http://www.ncbi.nlm.nih.gov/entrez/viewer.fcgi?val=CY052178) | 1734 | USA | 12-sep |  | [CY052449](http://www.ncbi.nlm.nih.gov/entrez/viewer.fcgi?val=CY052449) | 1420 | USA | 13-sep |
|  | [CY051767](http://www.ncbi.nlm.nih.gov/entrez/viewer.fcgi?val=CY051767) | 1734 | USA | 03-sep |  | [CY052569](http://www.ncbi.nlm.nih.gov/entrez/viewer.fcgi?val=CY052569) | 1420 | USA | 13-sep |
| 1022 | [CY052591](http://www.ncbi.nlm.nih.gov/entrez/viewer.fcgi?val=CY052591) | 1734 | USA | 03-sep | 886 | [CY052913](http://www.ncbi.nlm.nih.gov/entrez/viewer.fcgi?val=CY052913) | 1420 | USA | 11-sep |
| 1023 | [CY052599](http://www.ncbi.nlm.nih.gov/entrez/viewer.fcgi?val=CY052599) | 1736 | USA | 03-sep | 887 | [CY050013](http://www.ncbi.nlm.nih.gov/entrez/viewer.fcgi?val=CY050013) | 1410 | Mexico | 12-sep |
| 1024 | [CY052607](http://www.ncbi.nlm.nih.gov/entrez/viewer.fcgi?val=CY052607) | 1734 | USA | 03-sep | 888 | [CY052441](http://www.ncbi.nlm.nih.gov/entrez/viewer.fcgi?val=CY052441) | 1420 | USA | 12-sep |
| 1025 | [CY052903](http://www.ncbi.nlm.nih.gov/entrez/viewer.fcgi?val=CY052903) | 1734 | USA | 03-sep | 889 | [CY052489](http://www.ncbi.nlm.nih.gov/entrez/viewer.fcgi?val=CY052489) | 1420 | USA | 12-sep |
| 1026 | [CY052935](http://www.ncbi.nlm.nih.gov/entrez/viewer.fcgi?val=CY052935) | 1734 | USA | 03-sep | 890 | [CY052561](http://www.ncbi.nlm.nih.gov/entrez/viewer.fcgi?val=CY052561) | 1420 | USA | 12-sep |
| 1027 | [CY052783](http://www.ncbi.nlm.nih.gov/entrez/viewer.fcgi?val=CY052783) | 1732 | USA | 04-sep | 891 | [CY049653](http://www.ncbi.nlm.nih.gov/entrez/viewer.fcgi?val=CY049653) | 1399 | Singapore | 13-sep |
| 1028 | [CY052751](http://www.ncbi.nlm.nih.gov/entrez/viewer.fcgi?val=CY052751) | 1732 | USA | 10-sep | 892 | [CY052577](http://www.ncbi.nlm.nih.gov/entrez/viewer.fcgi?val=CY052577) | 1420 | USA | 13-sep |
|  | [CY052863](http://www.ncbi.nlm.nih.gov/entrez/viewer.fcgi?val=CY052863) | 1734 | USA | 04-sep | 893 | [CY050029](http://www.ncbi.nlm.nih.gov/entrez/viewer.fcgi?val=CY050029) | 1410 | Mexico | 14-sep |
| 1029 | [CY052919](http://www.ncbi.nlm.nih.gov/entrez/viewer.fcgi?val=CY052919) | 1734 | USA | 05-sep | 894 | [CY052713](http://www.ncbi.nlm.nih.gov/entrez/viewer.fcgi?val=CY052713) | 1420 | USA | 14-sep |
| 1030 | [GU108486](http://www.ncbi.nlm.nih.gov/entrez/viewer.fcgi?val=GU108486) | 1774 | China | 06-sep | 895 | [CY049025](http://www.ncbi.nlm.nih.gov/entrez/viewer.fcgi?val=CY049025) | 1244 | Spain | 15-sep |
| 1031 | [CY051895](http://www.ncbi.nlm.nih.gov/entrez/viewer.fcgi?val=CY051895) | 1734 | USA | 06-sep | 896 | [CY053476](http://www.ncbi.nlm.nih.gov/entrez/viewer.fcgi?val=CY053476) | 1437 | Taiwan | 15-sep |
| 1032 | [CY052543](http://www.ncbi.nlm.nih.gov/entrez/viewer.fcgi?val=CY052543) | 1734 | USA | 13-sep |  | [CY053484](http://www.ncbi.nlm.nih.gov/entrez/viewer.fcgi?val=CY053484) | 1437 | Taiwan | 18-sep |
|  | [CY052631](http://www.ncbi.nlm.nih.gov/entrez/viewer.fcgi?val=CY052631) | 1733 | USA | 06-sep |  | [CY053492](http://www.ncbi.nlm.nih.gov/entrez/viewer.fcgi?val=CY053492) | 1437 | Taiwan | 18-sep |
| 1033 | [CY052639](http://www.ncbi.nlm.nih.gov/entrez/viewer.fcgi?val=CY052639) | 1734 | USA | 06-sep |  | [CY053500](http://www.ncbi.nlm.nih.gov/entrez/viewer.fcgi?val=CY053500) | 1437 | Taiwan | 18-sep |
| 1034 | [CY052887](http://www.ncbi.nlm.nih.gov/entrez/viewer.fcgi?val=CY052887) | 1733 | USA | 06-sep | 897 | [CY049028](http://www.ncbi.nlm.nih.gov/entrez/viewer.fcgi?val=CY049028) | 1244 | Spain | 16-sep |
| 1035 | [CY052951](http://www.ncbi.nlm.nih.gov/entrez/viewer.fcgi?val=CY052951) | 1734 | USA | 06-sep | 898 | [CY049031](http://www.ncbi.nlm.nih.gov/entrez/viewer.fcgi?val=CY049031) | 1245 | Spain | 17-sep |
| 1036 | [GU112090](http://www.ncbi.nlm.nih.gov/entrez/viewer.fcgi?val=GU112090) | 1769 | China | 07-sep | 899 | [CY049034](http://www.ncbi.nlm.nih.gov/entrez/viewer.fcgi?val=CY049034) | 1248 | Spain | 17-sep |
| 1037 | [GU189649](http://www.ncbi.nlm.nih.gov/entrez/viewer.fcgi?val=GU189649) | 1769 | China | 07-sep | 900 | [CY053508](http://www.ncbi.nlm.nih.gov/entrez/viewer.fcgi?val=CY053508) | 1442 | Taiwan | 18-sep |
|  | [CY052007](http://www.ncbi.nlm.nih.gov/entrez/viewer.fcgi?val=CY052007) | 1744 | Norway | 07-sep | 901 | [CY049037](http://www.ncbi.nlm.nih.gov/entrez/viewer.fcgi?val=CY049037) | 1252 | Spain | 21-sep |
| 1038 | [CY052687](http://www.ncbi.nlm.nih.gov/entrez/viewer.fcgi?val=CY052687) | 1734 | USA | 07-sep | 902 | [CY049046](http://www.ncbi.nlm.nih.gov/entrez/viewer.fcgi?val=CY049046) | 1243 | Spain | 21-sep |
| 1039 | [CY052791](http://www.ncbi.nlm.nih.gov/entrez/viewer.fcgi?val=CY052791) | 1734 | USA | 07-sep | 903 | [CY049040](http://www.ncbi.nlm.nih.gov/entrez/viewer.fcgi?val=CY049040) | 1244 | Spain | 22-sep |
| 1040 | [CY052807](http://www.ncbi.nlm.nih.gov/entrez/viewer.fcgi?val=CY052807) | 1734 | USA | 07-sep | 904 | [GU211229](http://www.ncbi.nlm.nih.gov/entrez/viewer.fcgi?val=GU211229) | 1434 | Russia | 22-sep |
| 1041 | [CY052823](http://www.ncbi.nlm.nih.gov/entrez/viewer.fcgi?val=CY052823) | 1734 | USA | 07-sep | 905 | [CY049049](http://www.ncbi.nlm.nih.gov/entrez/viewer.fcgi?val=CY049049) | 1252 | Spain | 28-sep |
| 1042 | [CY051998](http://www.ncbi.nlm.nih.gov/entrez/viewer.fcgi?val=CY051998) | 1744 | Norway | 08-sep | 906 | [CY049052](http://www.ncbi.nlm.nih.gov/entrez/viewer.fcgi?val=CY049052) | 1242 | Spain | 28-sep |
| 1043 | [CY049018](http://www.ncbi.nlm.nih.gov/entrez/viewer.fcgi?val=CY049018) | 1572 | Spain | 09-sep | 907 | [CY049705](http://www.ncbi.nlm.nih.gov/entrez/viewer.fcgi?val=CY049705) | 1252 | Spain | 05-oct |
| 1044 | [CY049021](http://www.ncbi.nlm.nih.gov/entrez/viewer.fcgi?val=CY049021) | 1538 | Spain | 09-sep | 908 | [CY049708](http://www.ncbi.nlm.nih.gov/entrez/viewer.fcgi?val=CY049708) | 1246 | Spain | 05-oct |
| 1045 | [CY050043](http://www.ncbi.nlm.nih.gov/entrez/viewer.fcgi?val=CY050043) | 1698 | Mexico | 09-sep | 909 | [CY049711](http://www.ncbi.nlm.nih.gov/entrez/viewer.fcgi?val=CY049711) | 1251 | Spain | 07-oct |
| 1046 | [CY051799](http://www.ncbi.nlm.nih.gov/entrez/viewer.fcgi?val=CY051799) | 1734 | USA | 09-sep | 910 | [CY049714](http://www.ncbi.nlm.nih.gov/entrez/viewer.fcgi?val=CY049714) | 1250 | Spain | 08-oct |
| 1047 | [CY052005](http://www.ncbi.nlm.nih.gov/entrez/viewer.fcgi?val=CY052005) | 1744 | Norway | 09-sep | 911 | [CY049720](http://www.ncbi.nlm.nih.gov/entrez/viewer.fcgi?val=CY049720) | 1252 | Spain | 13-oct |
| 1048 | [CY052006](http://www.ncbi.nlm.nih.gov/entrez/viewer.fcgi?val=CY052006) | 1744 | Norway | 09-sep | 912 | [CY049726](http://www.ncbi.nlm.nih.gov/entrez/viewer.fcgi?val=CY049726) | 1257 | Spain | 13-oct |
| 1049 | [CY052655](http://www.ncbi.nlm.nih.gov/entrez/viewer.fcgi?val=CY052655) | 1734 | USA | 09-sep | 913 | [CY049723](http://www.ncbi.nlm.nih.gov/entrez/viewer.fcgi?val=CY049723) | 1246 | Spain | 14-oct |
| 1050 | [CY052703](http://www.ncbi.nlm.nih.gov/entrez/viewer.fcgi?val=CY052703) | 1732 | USA | 09-sep | 914 | [GU198203](http://www.ncbi.nlm.nih.gov/entrez/viewer.fcgi?val=GU198203) | 1410 | China | 17-oct |
| 1051 | [CY052719](http://www.ncbi.nlm.nih.gov/entrez/viewer.fcgi?val=CY052719) | 1734 | USA | 09-sep | 915 | [CY049729](http://www.ncbi.nlm.nih.gov/entrez/viewer.fcgi?val=CY049729) | 1252 | Spain | 19-oct |
| 1052 | [CY052727](http://www.ncbi.nlm.nih.gov/entrez/viewer.fcgi?val=CY052727) | 1734 | USA | 09-sep | 916 | [CY049731](http://www.ncbi.nlm.nih.gov/entrez/viewer.fcgi?val=CY049731) | 1252 | Spain | 19-oct |
| 1053 | [CY050059](http://www.ncbi.nlm.nih.gov/entrez/viewer.fcgi?val=CY050059) | 1698 | Mexico | 10-sep | 917 | [CY049734](http://www.ncbi.nlm.nih.gov/entrez/viewer.fcgi?val=CY049734) | 1251 | Spain | 20-oct |
| 1054 | [CY051807](http://www.ncbi.nlm.nih.gov/entrez/viewer.fcgi?val=CY051807) | 1734 | USA | 10-sep | 918 | [CY049740](http://www.ncbi.nlm.nih.gov/entrez/viewer.fcgi?val=CY049740) | 1253 | Spain | 21-oct |
| 1055 | [AB535742](http://www.ncbi.nlm.nih.gov/entrez/viewer.fcgi?val=AB535742) | 1701 | Japan | 10-sep | 919 | [CY049743](http://www.ncbi.nlm.nih.gov/entrez/viewer.fcgi?val=CY049743) | 1252 | Spain | 21-oct |
| 1056 | [CY052511](http://www.ncbi.nlm.nih.gov/entrez/viewer.fcgi?val=CY052511) | 1734 | USA | 10-sep | 920 | [CY049737](http://www.ncbi.nlm.nih.gov/entrez/viewer.fcgi?val=CY049737) | 1251 | Spain | 22-oct |
| 1057 | [CY052519](http://www.ncbi.nlm.nih.gov/entrez/viewer.fcgi?val=CY052519) | 1734 | USA | 10-sep | 921 | [CY050299](http://www.ncbi.nlm.nih.gov/entrez/viewer.fcgi?val=CY050299) | 1245 | Spain | 26-oct |
| 1058 | [CY052527](http://www.ncbi.nlm.nih.gov/entrez/viewer.fcgi?val=CY052527) | 1734 | USA | 10-sep | 922 | [CY050302](http://www.ncbi.nlm.nih.gov/entrez/viewer.fcgi?val=CY050302) | 1244 | Spain | 26-oct |
| 1059 | [CY052647](http://www.ncbi.nlm.nih.gov/entrez/viewer.fcgi?val=CY052647) | 1733 | USA | 10-sep | 923 | [CY050305](http://www.ncbi.nlm.nih.gov/entrez/viewer.fcgi?val=CY050305) | 1243 | Spain | 26-oct |
| 1060 | [CY052743](http://www.ncbi.nlm.nih.gov/entrez/viewer.fcgi?val=CY052743) | 1734 | USA | 10-sep | 924 | [CY050311](http://www.ncbi.nlm.nih.gov/entrez/viewer.fcgi?val=CY050311) | 1245 | Spain | 26-oct |
| 1061 | [CY052759](http://www.ncbi.nlm.nih.gov/entrez/viewer.fcgi?val=CY052759) | 1734 | USA | 10-sep | 925 | [AB537490](http://www.ncbi.nlm.nih.gov/entrez/viewer.fcgi?val=AB537490) | 1410 | Japan | 27-oct |
| 1062 | [CY052799](http://www.ncbi.nlm.nih.gov/entrez/viewer.fcgi?val=CY052799) | 1734 | USA | 13-sep | 926 | [CY050314](http://www.ncbi.nlm.nih.gov/entrez/viewer.fcgi?val=CY050314) | 1250 | Spain | 28-oct |
|  | [CY052895](http://www.ncbi.nlm.nih.gov/entrez/viewer.fcgi?val=CY052895) | 1734 | USA | 10-sep | 927 | [CY050320](http://www.ncbi.nlm.nih.gov/entrez/viewer.fcgi?val=CY050320) | 1247 | Spain | 28-oct |
| 1063 | [CY052495](http://www.ncbi.nlm.nih.gov/entrez/viewer.fcgi?val=CY052495) | 1734 | USA | 11-sep | 928 | [CY050323](http://www.ncbi.nlm.nih.gov/entrez/viewer.fcgi?val=CY050323) | 1244 | Spain | 28-oct |
|  | [CY052535](http://www.ncbi.nlm.nih.gov/entrez/viewer.fcgi?val=CY052535) | 1734 | USA | 11-sep | 929 | [CY050326](http://www.ncbi.nlm.nih.gov/entrez/viewer.fcgi?val=CY050326) | 1250 | Spain | 29-oct |
| 1064 | [CY052431](http://www.ncbi.nlm.nih.gov/entrez/viewer.fcgi?val=CY052431) | 1734 | USA | 12-sep | 930 | [CY053340](http://www.ncbi.nlm.nih.gov/entrez/viewer.fcgi?val=CY053340) | 1411 | China | Nov |
|  | [CY052479](http://www.ncbi.nlm.nih.gov/entrez/viewer.fcgi?val=CY052479) | 1734 | USA | 12-sep | 931 | [CY053344](http://www.ncbi.nlm.nih.gov/entrez/viewer.fcgi?val=CY053344) | 1409 | China | Nov |
|  | [CY052567](http://www.ncbi.nlm.nih.gov/entrez/viewer.fcgi?val=CY052567) | 1734 | USA | 13-sep | 932 | [CY053350](http://www.ncbi.nlm.nih.gov/entrez/viewer.fcgi?val=CY053350) | 1406 | China | Nov |
|  | [CY052911](http://www.ncbi.nlm.nih.gov/entrez/viewer.fcgi?val=CY052911) | 1734 | USA | 11-sep | 933 | [CY053418](http://www.ncbi.nlm.nih.gov/entrez/viewer.fcgi?val=CY053418) | 1451 | Russia | 01-nov |
| 1065 | [CY050011](http://www.ncbi.nlm.nih.gov/entrez/viewer.fcgi?val=CY050011) | 1695 | Mexico | 12-sep | 934 | [CY053683](http://www.ncbi.nlm.nih.gov/entrez/viewer.fcgi?val=CY053683) | 1480 | Russia | 01-nov |
| 1066 | [CY052439](http://www.ncbi.nlm.nih.gov/entrez/viewer.fcgi?val=CY052439) | 1734 | USA | 12-sep | 935 | [CY053691](http://www.ncbi.nlm.nih.gov/entrez/viewer.fcgi?val=CY053691) | 1470 | Russia | 01-nov |
| 1067 | [CY052487](http://www.ncbi.nlm.nih.gov/entrez/viewer.fcgi?val=CY052487) | 1734 | USA | 12-sep | 936 | [CY053730](http://www.ncbi.nlm.nih.gov/entrez/viewer.fcgi?val=CY053730) | 1482 | Russia | 01-nov |
| 1068 | [CY052551](http://www.ncbi.nlm.nih.gov/entrez/viewer.fcgi?val=CY052551) | 1734 | USA | 12-sep | 937 | [CY053738](http://www.ncbi.nlm.nih.gov/entrez/viewer.fcgi?val=CY053738) | 1458 | Russia | 01-nov |
| 1069 | [CY052559](http://www.ncbi.nlm.nih.gov/entrez/viewer.fcgi?val=CY052559) | 1734 | USA | 12-sep | 938 | [CY053746](http://www.ncbi.nlm.nih.gov/entrez/viewer.fcgi?val=CY053746) | 1429 | Russia | 01-nov |
| 1070 | [CY052927](http://www.ncbi.nlm.nih.gov/entrez/viewer.fcgi?val=CY052927) | 1734 | USA | 12-sep | 939 | [CY053754](http://www.ncbi.nlm.nih.gov/entrez/viewer.fcgi?val=CY053754) | 1458 | Russia | 01-nov |
| 1071 | [CY052943](http://www.ncbi.nlm.nih.gov/entrez/viewer.fcgi?val=CY052943) | 1732 | USA | 12-sep | 940 | [CY051932](http://www.ncbi.nlm.nih.gov/entrez/viewer.fcgi?val=CY051932) | 1251 | Spain | 02-nov |
| 1072 | [CY049651](http://www.ncbi.nlm.nih.gov/entrez/viewer.fcgi?val=CY049651) | 1738 | Singapore | 13-sep | 941 | [CY051935](http://www.ncbi.nlm.nih.gov/entrez/viewer.fcgi?val=CY051935) | 1240 | Spain | 02-nov |
| 1073 | [CY052447](http://www.ncbi.nlm.nih.gov/entrez/viewer.fcgi?val=CY052447) | 1734 | USA | 13-sep | 942 | [CY051938](http://www.ncbi.nlm.nih.gov/entrez/viewer.fcgi?val=CY051938) | 1243 | Spain | 02-nov |
| 1074 | [CY052575](http://www.ncbi.nlm.nih.gov/entrez/viewer.fcgi?val=CY052575) | 1734 | USA | 13-sep | 943 | [CY051941](http://www.ncbi.nlm.nih.gov/entrez/viewer.fcgi?val=CY051941) | 1247 | Spain | 02-nov |
| 1075 | [CY052583](http://www.ncbi.nlm.nih.gov/entrez/viewer.fcgi?val=CY052583) | 1734 | USA | 13-sep | 944 | [CY051944](http://www.ncbi.nlm.nih.gov/entrez/viewer.fcgi?val=CY051944) | 1240 | Spain | 02-nov |
| 1076 | [CY052831](http://www.ncbi.nlm.nih.gov/entrez/viewer.fcgi?val=CY052831) | 1734 | USA | 13-sep | 945 | [CY051953](http://www.ncbi.nlm.nih.gov/entrez/viewer.fcgi?val=CY051953) | 1240 | Spain | 02-nov |
| 1077 | [CY052839](http://www.ncbi.nlm.nih.gov/entrez/viewer.fcgi?val=CY052839) | 1734 | USA | 13-sep | 946 | [CY051956](http://www.ncbi.nlm.nih.gov/entrez/viewer.fcgi?val=CY051956) | 1250 | Spain | 02-nov |
| 1078 | [CY050027](http://www.ncbi.nlm.nih.gov/entrez/viewer.fcgi?val=CY050027) | 1698 | Mexico | 14-sep | 947 | [CY051959](http://www.ncbi.nlm.nih.gov/entrez/viewer.fcgi?val=CY051959) | 1246 | Spain | 02-nov |
| 1079 | [CY052711](http://www.ncbi.nlm.nih.gov/entrez/viewer.fcgi?val=CY052711) | 1734 | USA | 14-sep | 948 | [CY051947](http://www.ncbi.nlm.nih.gov/entrez/viewer.fcgi?val=CY051947) | 1246 | Spain | 03-nov |
| 1080 | [AB539047](http://www.ncbi.nlm.nih.gov/entrez/viewer.fcgi?val=AB539047) | 1701 | Japan | 14-sep | 949 | [CY051950](http://www.ncbi.nlm.nih.gov/entrez/viewer.fcgi?val=CY051950) | 1235 | Spain | 03-nov |
| 1081 | [CY049024](http://www.ncbi.nlm.nih.gov/entrez/viewer.fcgi?val=CY049024) | 1558 | Spain | 15-sep | 950 | [CY051928](http://www.ncbi.nlm.nih.gov/entrez/viewer.fcgi?val=CY051928) | 1250 | Spain | 04-nov |
| 1082 | [CY053474](http://www.ncbi.nlm.nih.gov/entrez/viewer.fcgi?val=CY053474) | 1758 | Taiwan | 15-sep | 951 | [CY051962](http://www.ncbi.nlm.nih.gov/entrez/viewer.fcgi?val=CY051962) | 1244 | Spain | 09-nov |
| 1083 | [CY047713](http://www.ncbi.nlm.nih.gov/entrez/viewer.fcgi?val=CY047713) | 1701 | China | 16-sep | 952 | [CY051965](http://www.ncbi.nlm.nih.gov/entrez/viewer.fcgi?val=CY051965) | 1246 | Spain | 09-nov |
| 1084 | [CY049030](http://www.ncbi.nlm.nih.gov/entrez/viewer.fcgi?val=CY049030) | 1559 | Spain | 17-sep | 953 | [CY051968](http://www.ncbi.nlm.nih.gov/entrez/viewer.fcgi?val=CY051968) | 1245 | Spain | 09-nov |
| 1085 | [CY049033](http://www.ncbi.nlm.nih.gov/entrez/viewer.fcgi?val=CY049033) | 1555 | Spain | 17-sep | 954 | [CY051971](http://www.ncbi.nlm.nih.gov/entrez/viewer.fcgi?val=CY051971) | 1246 | Spain | 09-nov |
| 1086 | [CY053482](http://www.ncbi.nlm.nih.gov/entrez/viewer.fcgi?val=CY053482) | 1778 | Taiwan | 18-sep | 955 | [CY051974](http://www.ncbi.nlm.nih.gov/entrez/viewer.fcgi?val=CY051974) | 1243 | Spain | 09-nov |
| 1087 | [CY053490](http://www.ncbi.nlm.nih.gov/entrez/viewer.fcgi?val=CY053490) | 1764 | Taiwan | 18-sep | 956 | [CY051977](http://www.ncbi.nlm.nih.gov/entrez/viewer.fcgi?val=CY051977) | 1244 | Spain | 09-nov |
| 1088 | [CY053498](http://www.ncbi.nlm.nih.gov/entrez/viewer.fcgi?val=CY053498) | 1758 | Taiwan | 18-sep | 957 | [CY051980](http://www.ncbi.nlm.nih.gov/entrez/viewer.fcgi?val=CY051980) | 1245 | Spain | 09-nov |
| 1089 | [CY053506](http://www.ncbi.nlm.nih.gov/entrez/viewer.fcgi?val=CY053506) | 1758 | Taiwan | 18-sep | 958 | [CY051983](http://www.ncbi.nlm.nih.gov/entrez/viewer.fcgi?val=CY051983) | 1241 | Spain | 09-nov |
|  | [CY047714](http://www.ncbi.nlm.nih.gov/entrez/viewer.fcgi?val=CY047714) | 1701 | China | 19-sep | 959 | [CY053629](http://www.ncbi.nlm.nih.gov/entrez/viewer.fcgi?val=CY053629) | 1539 | Russia | 10-nov |
| 1090 | [CY047712](http://www.ncbi.nlm.nih.gov/entrez/viewer.fcgi?val=CY047712) | 1701 | China | 20-sep | 960 | [CY052033](http://www.ncbi.nlm.nih.gov/entrez/viewer.fcgi?val=CY052033) | 1252 | Spain | 12-nov |
| 1091 | [CY052008](http://www.ncbi.nlm.nih.gov/entrez/viewer.fcgi?val=CY052008) | 1744 | Norway | 20-sep | 961 | [CY052039](http://www.ncbi.nlm.nih.gov/entrez/viewer.fcgi?val=CY052039) | 1250 | Spain | 12-nov |
| 1092 | [CY049036](http://www.ncbi.nlm.nih.gov/entrez/viewer.fcgi?val=CY049036) | 1544 | Spain | 21-sep | 962 | [CY052029](http://www.ncbi.nlm.nih.gov/entrez/viewer.fcgi?val=CY052029) | 1253 | Spain | 13-nov |
| 1093 | [CY049045](http://www.ncbi.nlm.nih.gov/entrez/viewer.fcgi?val=CY049045) | 1562 | Spain | 21-sep | 963 | [CY052031](http://www.ncbi.nlm.nih.gov/entrez/viewer.fcgi?val=CY052031) | 1252 | Spain | 13-nov |
| 1094 | [CY049039](http://www.ncbi.nlm.nih.gov/entrez/viewer.fcgi?val=CY049039) | 1559 | Spain | 22-sep | 964 | [CY052035](http://www.ncbi.nlm.nih.gov/entrez/viewer.fcgi?val=CY052035) | 1244 | Spain | 13-nov |
| 1095 | [CY049042](http://www.ncbi.nlm.nih.gov/entrez/viewer.fcgi?val=CY049042) | 1557 | Spain | 23-sep | 965 | [CY052037](http://www.ncbi.nlm.nih.gov/entrez/viewer.fcgi?val=CY052037) | 1248 | Spain | 13-nov |
| 1096 | [CY049048](http://www.ncbi.nlm.nih.gov/entrez/viewer.fcgi?val=CY049048) | 1561 | Spain | 28-sep | 966 | [GU451256](http://www.ncbi.nlm.nih.gov/entrez/viewer.fcgi?val=GU451256) | 1428 | Russia | 14-nov |
| 1097 | [CY049051](http://www.ncbi.nlm.nih.gov/entrez/viewer.fcgi?val=CY049051) | 1562 | Spain | 28-sep | 967 | [GU216651](http://www.ncbi.nlm.nih.gov/entrez/viewer.fcgi?val=GU216651) | 1390 | Italy | 16-nov |
| 1098 | [CY049054](http://www.ncbi.nlm.nih.gov/entrez/viewer.fcgi?val=CY049054) | 1560 | Spain | 30-sep | 968 | [CY052043](http://www.ncbi.nlm.nih.gov/entrez/viewer.fcgi?val=CY052043) | 1242 | Spain | 16-nov |
| 1099 | [CY052051](http://www.ncbi.nlm.nih.gov/entrez/viewer.fcgi?val=CY052051) | 950 | Belarus | Oct | 969 | [GU371257](http://www.ncbi.nlm.nih.gov/entrez/viewer.fcgi?val=GU371257) | 1428 | Russia | 16-nov |
| 1100 | [GU369663](http://www.ncbi.nlm.nih.gov/entrez/viewer.fcgi?val=GU369663) | 1769 | Turkey | Oct | 970 | [GU361110](http://www.ncbi.nlm.nih.gov/entrez/viewer.fcgi?val=GU361110) | 1378 | Sweden | 17-nov |
| 1101 | [GU369664](http://www.ncbi.nlm.nih.gov/entrez/viewer.fcgi?val=GU369664) | 1782 | Turkey | Oct | 971 | [CY053374](http://www.ncbi.nlm.nih.gov/entrez/viewer.fcgi?val=CY053374) | 1254 | Spain | 23-nov |
| 1102 | [GU369665](http://www.ncbi.nlm.nih.gov/entrez/viewer.fcgi?val=GU369665) | 1787 | Turkey | Oct | 972 | [CY053377](http://www.ncbi.nlm.nih.gov/entrez/viewer.fcgi?val=CY053377) | 1250 | Spain | 23-nov |
| 1103 | [CY052009](http://www.ncbi.nlm.nih.gov/entrez/viewer.fcgi?val=CY052009) | 1744 | Norway | 02-oct | 973 | [CY053383](http://www.ncbi.nlm.nih.gov/entrez/viewer.fcgi?val=CY053383) | 1245 | Spain | 23-nov |
| 1104 | [CY049704](http://www.ncbi.nlm.nih.gov/entrez/viewer.fcgi?val=CY049704) | 1560 | Spain | 05-oct | 974 | [CY053386](http://www.ncbi.nlm.nih.gov/entrez/viewer.fcgi?val=CY053386) | 1252 | Spain | 23-nov |
| 1105 | [CY049707](http://www.ncbi.nlm.nih.gov/entrez/viewer.fcgi?val=CY049707) | 1558 | Spain | 05-oct | 975 | [CY053389](http://www.ncbi.nlm.nih.gov/entrez/viewer.fcgi?val=CY053389) | 1251 | Spain | 23-nov |
| 1106 | [AB535743](http://www.ncbi.nlm.nih.gov/entrez/viewer.fcgi?val=AB535743) | 1701 | Japan | 06-oct | 976 | [CY053392](http://www.ncbi.nlm.nih.gov/entrez/viewer.fcgi?val=CY053392) | 1251 | Spain | 23-nov |
| 1107 | [AB539048](http://www.ncbi.nlm.nih.gov/entrez/viewer.fcgi?val=AB539048) | 1701 | Japan | Dec | 977 | [CY053395](http://www.ncbi.nlm.nih.gov/entrez/viewer.fcgi?val=CY053395) | 1251 | Spain | 24-nov |
| 1107 | [CY049710](http://www.ncbi.nlm.nih.gov/entrez/viewer.fcgi?val=CY049710) | 1560 | Spain | 07-oct | 978 | [CY052025](http://www.ncbi.nlm.nih.gov/entrez/viewer.fcgi?val=CY052025) | 1261 | Spain | 25-nov |
| 1108 | [CY049713](http://www.ncbi.nlm.nih.gov/entrez/viewer.fcgi?val=CY049713) | 1556 | Spain | 08-oct | 979 | [CY052027](http://www.ncbi.nlm.nih.gov/entrez/viewer.fcgi?val=CY052027) | 1247 | Spain | 26-nov |
| 1109 | [AB535744](http://www.ncbi.nlm.nih.gov/entrez/viewer.fcgi?val=AB535744) | 1701 | Japan | 09-oct | 980 | [CY053398](http://www.ncbi.nlm.nih.gov/entrez/viewer.fcgi?val=CY053398) | 1243 | Spain | 30-nov |
| 1110 | [AB535745](http://www.ncbi.nlm.nih.gov/entrez/viewer.fcgi?val=AB535745) | 1701 | Japan | 09-oct |  | [CY053703](http://www.ncbi.nlm.nih.gov/entrez/viewer.fcgi?val=CY053703) | 1243 | Spain | 9-Dec |
| 1111 | [CY052011](http://www.ncbi.nlm.nih.gov/entrez/viewer.fcgi?val=CY052011) | 1744 | Norway | 12-oct | 981 | [CY053402](http://www.ncbi.nlm.nih.gov/entrez/viewer.fcgi?val=CY053402) | 1250 | Spain | 30-nov |
| 1112 | [CY049716](http://www.ncbi.nlm.nih.gov/entrez/viewer.fcgi?val=CY049716) | 1567 | Spain | 13-oct | 982 | [CY053410](http://www.ncbi.nlm.nih.gov/entrez/viewer.fcgi?val=CY053410) | 1250 | Spain | 30-nov |
| 1113 | [CY049719](http://www.ncbi.nlm.nih.gov/entrez/viewer.fcgi?val=CY049719) | 1557 | Spain | 13-oct | 983 | [CY053406](http://www.ncbi.nlm.nih.gov/entrez/viewer.fcgi?val=CY053406) | 1251 | Spain | 1-Dec |
| 1114 | [CY049725](http://www.ncbi.nlm.nih.gov/entrez/viewer.fcgi?val=CY049725) | 1560 | Spain | 13-oct | 984 | [CY053707](http://www.ncbi.nlm.nih.gov/entrez/viewer.fcgi?val=CY053707) | 1089 | Spain | 2-Dec |
| 1115 | [CY049722](http://www.ncbi.nlm.nih.gov/entrez/viewer.fcgi?val=CY049722) | 1545 | Spain | 14-oct | 985 | [CY053711](http://www.ncbi.nlm.nih.gov/entrez/viewer.fcgi?val=CY053711) | 1246 | Spain | 2-Dec |
| 1116 | [CY052010](http://www.ncbi.nlm.nih.gov/entrez/viewer.fcgi?val=CY052010) | 1744 | Norway | 14-oct | 986 | [CY053370](http://www.ncbi.nlm.nih.gov/entrez/viewer.fcgi?val=CY053370) | 1252 | Spain | 3-Dec |
| 1117 | [CY052015](http://www.ncbi.nlm.nih.gov/entrez/viewer.fcgi?val=CY052015) | 1744 | Norway | 14-oct | 987 | [CY053715](http://www.ncbi.nlm.nih.gov/entrez/viewer.fcgi?val=CY053715) | 1247 | Spain | 3-Dec |
| 1118 | [GU198201](http://www.ncbi.nlm.nih.gov/entrez/viewer.fcgi?val=GU198201) | 1701 | China | 17-oct | 988 | [CY053719](http://www.ncbi.nlm.nih.gov/entrez/viewer.fcgi?val=CY053719) | 1246 | Spain | 7-Dec |
| 1119 | [CY049728](http://www.ncbi.nlm.nih.gov/entrez/viewer.fcgi?val=CY049728) | 1546 | Spain | 19-oct | 989 | [CY053723](http://www.ncbi.nlm.nih.gov/entrez/viewer.fcgi?val=CY053723) | 1251 | Spain | 7-Dec |
| 1120 | [CY049730](http://www.ncbi.nlm.nih.gov/entrez/viewer.fcgi?val=CY049730) | 1549 | Spain | 19-oct | 990 | [CY053696](http://www.ncbi.nlm.nih.gov/entrez/viewer.fcgi?val=CY053696) | 1244 | Spain | 10-Dec |
|  | [CY052012](http://www.ncbi.nlm.nih.gov/entrez/viewer.fcgi?val=CY052012) | 1744 | Norway | 19-oct | 991 | [CY053700](http://www.ncbi.nlm.nih.gov/entrez/viewer.fcgi?val=CY053700) | 1250 | Spain | 10-Dec |
| 1121 | [CY052013](http://www.ncbi.nlm.nih.gov/entrez/viewer.fcgi?val=CY052013) | 1744 | Norway | 24-oct |  |  |  |  |  |
|  | [CY049733](http://www.ncbi.nlm.nih.gov/entrez/viewer.fcgi?val=CY049733) | 1560 | Spain | 20-oct |  |  |  |  |  |
| 1122 | [CY049739](http://www.ncbi.nlm.nih.gov/entrez/viewer.fcgi?val=CY049739) | 1538 | Spain | 21-oct |  |  |  |  |  |
| 1123 | [CY049742](http://www.ncbi.nlm.nih.gov/entrez/viewer.fcgi?val=CY049742) | 1561 | Spain | 21-oct |  |  |  |  |  |
| 1124 | [CY049736](http://www.ncbi.nlm.nih.gov/entrez/viewer.fcgi?val=CY049736) | 1561 | Spain | 22-oct |  |  |  |  |  |
| 1125 | [AB539046](http://www.ncbi.nlm.nih.gov/entrez/viewer.fcgi?val=AB539046) | 1701 | Japan | 22-oct |  |  |  |  |  |
| 1126 | [CY052014](http://www.ncbi.nlm.nih.gov/entrez/viewer.fcgi?val=CY052014) | 1744 | Norway | 23-oct |  |  |  |  |  |
| 1127 | [CY050298](http://www.ncbi.nlm.nih.gov/entrez/viewer.fcgi?val=CY050298) | 1541 | Spain | 26-oct |  |  |  |  |  |
| 1128 | [CY050301](http://www.ncbi.nlm.nih.gov/entrez/viewer.fcgi?val=CY050301) | 1557 | Spain | 26-oct |  |  |  |  |  |
| 1129 | [CY050304](http://www.ncbi.nlm.nih.gov/entrez/viewer.fcgi?val=CY050304) | 1550 | Spain | 26-oct |  |  |  |  |  |
| 1130 | [CY050307](http://www.ncbi.nlm.nih.gov/entrez/viewer.fcgi?val=CY050307) | 1562 | Spain | 26-oct |  |  |  |  |  |
| 1131 | [CY050310](http://www.ncbi.nlm.nih.gov/entrez/viewer.fcgi?val=CY050310) | 1561 | Spain | 26-oct |  |  |  |  |  |
| 1132 | [CY050844](http://www.ncbi.nlm.nih.gov/entrez/viewer.fcgi?val=CY050844) | 1701 | Mongolia | 26-oct |  |  |  |  |  |
| 1133 | [AB536768](http://www.ncbi.nlm.nih.gov/entrez/viewer.fcgi?val=AB536768) | 1701 | Japan | 26-oct |  |  |  |  |  |
| 1134 | [AB535746](http://www.ncbi.nlm.nih.gov/entrez/viewer.fcgi?val=AB535746) | 1701 | Japan | 27-oct |  |  |  |  |  |
| 1135 | [AB536769](http://www.ncbi.nlm.nih.gov/entrez/viewer.fcgi?val=AB536769) | 1701 | Japan | 27-oct |  |  |  |  |  |
| 1136 | [CY050313](http://www.ncbi.nlm.nih.gov/entrez/viewer.fcgi?val=CY050313) | 1560 | Spain | 28-oct |  |  |  |  |  |
| 1137 | [CY050316](http://www.ncbi.nlm.nih.gov/entrez/viewer.fcgi?val=CY050316) | 1558 | Spain | 28-oct |  |  |  |  |  |
| 1138 | [CY050319](http://www.ncbi.nlm.nih.gov/entrez/viewer.fcgi?val=CY050319) | 1554 | Spain | 28-oct |  |  |  |  |  |
| 1139 | [CY050322](http://www.ncbi.nlm.nih.gov/entrez/viewer.fcgi?val=CY050322) | 1543 | Spain | 28-oct |  |  |  |  |  |
| 1140 | [AB535741](http://www.ncbi.nlm.nih.gov/entrez/viewer.fcgi?val=AB535741) | 1701 | Japan | 28-oct |  |  |  |  |  |
| 1141 | [CY050325](http://www.ncbi.nlm.nih.gov/entrez/viewer.fcgi?val=CY050325) | 1558 | Spain | 29-oct |  |  |  |  |  |
| 1142 | [CY050845](http://www.ncbi.nlm.nih.gov/entrez/viewer.fcgi?val=CY050845) | 1701 | Mongolia | 29-oct |  |  |  |  |  |
| 1143 | [CY050846](http://www.ncbi.nlm.nih.gov/entrez/viewer.fcgi?val=CY050846) | 1701 | Mongolia | 29-oct |  |  |  |  |  |
| 1144 | [AB535748](http://www.ncbi.nlm.nih.gov/entrez/viewer.fcgi?val=AB535748) | 1701 | Japan | 29-oct |  |  |  |  |  |
| 1145 | [AB536770](http://www.ncbi.nlm.nih.gov/entrez/viewer.fcgi?val=AB536770) | 1701 | Japan | 29-oct |  |  |  |  |  |
| 1146 | [GU369674](http://www.ncbi.nlm.nih.gov/entrez/viewer.fcgi?val=GU369674) | 1746 | Turkey | 31-oct |  |  |  |  |  |
| 1147 | [CY053338](http://www.ncbi.nlm.nih.gov/entrez/viewer.fcgi?val=CY053338) | 1724 | China | Nov |  |  |  |  |  |
| 1148 | [CY053348](http://www.ncbi.nlm.nih.gov/entrez/viewer.fcgi?val=CY053348) | 1722 | China | Nov |  |  |  |  |  |
| 1149 | [CY053353](http://www.ncbi.nlm.nih.gov/entrez/viewer.fcgi?val=CY053353) | 1721 | China | Nov |  |  |  |  |  |
| 1150 | [GU369667](http://www.ncbi.nlm.nih.gov/entrez/viewer.fcgi?val=GU369667) | 881 | Turkey | Nov |  |  |  |  |  |
| 1151 | [CY053416](http://www.ncbi.nlm.nih.gov/entrez/viewer.fcgi?val=CY053416) | 1763 | Russia | 01-nov |  |  |  |  |  |
| 1152 | [CY053736](http://www.ncbi.nlm.nih.gov/entrez/viewer.fcgi?val=CY053736) | 1753 | Russia | 01-nov |  |  |  |  |  |
| 1153 | [CY053744](http://www.ncbi.nlm.nih.gov/entrez/viewer.fcgi?val=CY053744) | 1747 | Russia | 01-nov |  |  |  |  |  |
| 1154 | [CY053752](http://www.ncbi.nlm.nih.gov/entrez/viewer.fcgi?val=CY053752) | 1793 | Russia | 01-nov |  |  |  |  |  |
| 1155 | [CY051931](http://www.ncbi.nlm.nih.gov/entrez/viewer.fcgi?val=CY051931) | 1554 | Spain | 02-nov |  |  |  |  |  |
| 1156 | [CY051934](http://www.ncbi.nlm.nih.gov/entrez/viewer.fcgi?val=CY051934) | 1551 | Spain | 02-nov |  |  |  |  |  |
| 1157 | [CY051937](http://www.ncbi.nlm.nih.gov/entrez/viewer.fcgi?val=CY051937) | 1542 | Spain | 02-nov |  |  |  |  |  |
| 1158 | [CY051940](http://www.ncbi.nlm.nih.gov/entrez/viewer.fcgi?val=CY051940) | 1351 | Spain | 02-nov |  |  |  |  |  |
| 1159 | [CY051943](http://www.ncbi.nlm.nih.gov/entrez/viewer.fcgi?val=CY051943) | 1553 | Spain | 02-nov |  |  |  |  |  |
| 1160 | [CY051952](http://www.ncbi.nlm.nih.gov/entrez/viewer.fcgi?val=CY051952) | 1498 | Spain | 02-nov |  |  |  |  |  |
| 1161 | [CY051955](http://www.ncbi.nlm.nih.gov/entrez/viewer.fcgi?val=CY051955) | 1557 | Spain | 02-nov |  |  |  |  |  |
| 1162 | [CY051958](http://www.ncbi.nlm.nih.gov/entrez/viewer.fcgi?val=CY051958) | 1553 | Spain | 02-nov |  |  |  |  |  |
| 1163 | [GU369668](http://www.ncbi.nlm.nih.gov/entrez/viewer.fcgi?val=GU369668) | 922 | Turkey | 02-nov |  |  |  |  |  |
| 1164 | [CY051946](http://www.ncbi.nlm.nih.gov/entrez/viewer.fcgi?val=CY051946) | 1554 | Spain | 03-nov |  |  |  |  |  |
| 1165 | [CY051949](http://www.ncbi.nlm.nih.gov/entrez/viewer.fcgi?val=CY051949) | 1559 | Spain | 03-nov |  |  |  |  |  |
| 1166 | [GU323343](http://www.ncbi.nlm.nih.gov/entrez/viewer.fcgi?val=GU323343) | 1767 | China | 03-nov |  |  |  |  |  |
| 1167 | [CY051927](http://www.ncbi.nlm.nih.gov/entrez/viewer.fcgi?val=CY051927) | 1509 | Spain | 04-nov |  |  |  |  |  |
| 1168 | [CY051961](http://www.ncbi.nlm.nih.gov/entrez/viewer.fcgi?val=CY051961) | 1570 | Spain | 09-nov |  |  |  |  |  |
| 1169 | [CY051964](http://www.ncbi.nlm.nih.gov/entrez/viewer.fcgi?val=CY051964) | 1569 | Spain | 09-nov |  |  |  |  |  |
| 1170 | [CY051967](http://www.ncbi.nlm.nih.gov/entrez/viewer.fcgi?val=CY051967) | 1568 | Spain | 09-nov |  |  |  |  |  |
| 1171 | [CY051970](http://www.ncbi.nlm.nih.gov/entrez/viewer.fcgi?val=CY051970) | 1568 | Spain | 09-nov |  |  |  |  |  |
| 1172 | [CY051973](http://www.ncbi.nlm.nih.gov/entrez/viewer.fcgi?val=CY051973) | 1570 | Spain | 09-nov |  |  |  |  |  |
| 1173 | [CY051976](http://www.ncbi.nlm.nih.gov/entrez/viewer.fcgi?val=CY051976) | 1570 | Spain | 09-nov |  |  |  |  |  |
| 1174 | [CY051979](http://www.ncbi.nlm.nih.gov/entrez/viewer.fcgi?val=CY051979) | 1555 | Spain | 09-nov |  |  |  |  |  |
| 1175 | [CY051982](http://www.ncbi.nlm.nih.gov/entrez/viewer.fcgi?val=CY051982) | 1568 | Spain | 09-nov |  |  |  |  |  |
| 1176 | [CY052366](http://www.ncbi.nlm.nih.gov/entrez/viewer.fcgi?val=CY052366) | 1701 | Mongolia | 10-nov |  |  |  |  |  |
| 1177 | [GU371263](http://www.ncbi.nlm.nih.gov/entrez/viewer.fcgi?val=GU371263) | 1744 | Russia | 11-nov |  |  |  |  |  |
| 1178 | [GU369671](http://www.ncbi.nlm.nih.gov/entrez/viewer.fcgi?val=GU369671) | 1782 | Turkey | 11-nov |  |  |  |  |  |
| 1179 | [CY052032](http://www.ncbi.nlm.nih.gov/entrez/viewer.fcgi?val=CY052032) | 1559 | Spain | 12-nov |  |  |  |  |  |
| 1180 | [CY052038](http://www.ncbi.nlm.nih.gov/entrez/viewer.fcgi?val=CY052038) | 1562 | Spain | 12-nov |  |  |  |  |  |
| 1181 | [GU369672](http://www.ncbi.nlm.nih.gov/entrez/viewer.fcgi?val=GU369672) | 1774 | Turkey | 12-nov |  |  |  |  |  |
| 1182 | [CY052028](http://www.ncbi.nlm.nih.gov/entrez/viewer.fcgi?val=CY052028) | 1553 | Spain | 13-nov |  |  |  |  |  |
| 1183 | [CY052030](http://www.ncbi.nlm.nih.gov/entrez/viewer.fcgi?val=CY052030) | 1557 | Spain | 13-nov |  |  |  |  |  |
| 1184 | [CY052034](http://www.ncbi.nlm.nih.gov/entrez/viewer.fcgi?val=CY052034) | 1561 | Spain | 13-nov |  |  |  |  |  |
| 1185 | [CY052036](http://www.ncbi.nlm.nih.gov/entrez/viewer.fcgi?val=CY052036) | 1561 | Spain | 13-nov |  |  |  |  |  |
| 1186 | [GU451254](http://www.ncbi.nlm.nih.gov/entrez/viewer.fcgi?val=GU451254) | 1744 | Russia | 14-nov |  |  |  |  |  |
| 1187 | [CY052040](http://www.ncbi.nlm.nih.gov/entrez/viewer.fcgi?val=CY052040) | 1539 | Spain | 16-nov |  |  |  |  |  |
| 1188 | [CY052042](http://www.ncbi.nlm.nih.gov/entrez/viewer.fcgi?val=CY052042) | 1563 | Spain | 16-nov |  |  |  |  |  |
| 1189 | [GU371256](http://www.ncbi.nlm.nih.gov/entrez/viewer.fcgi?val=GU371256) | 1744 | Russia | 16-nov |  |  |  |  |  |
| 1190 | [GU369673](http://www.ncbi.nlm.nih.gov/entrez/viewer.fcgi?val=GU369673) | 1782 | Turkey | 18-nov |  |  |  |  |  |
| 1191 | [GU371270](http://www.ncbi.nlm.nih.gov/entrez/viewer.fcgi?val=GU371270) | 1701 | China | 21-nov |  |  |  |  |  |
| 1192 | [CY053373](http://www.ncbi.nlm.nih.gov/entrez/viewer.fcgi?val=CY053373) | 1555 | Spain | 23-nov |  |  |  |  |  |
| 1193 | [CY053376](http://www.ncbi.nlm.nih.gov/entrez/viewer.fcgi?val=CY053376) | 1562 | Spain | 23-nov |  |  |  |  |  |
| 1194 | [CY053379](http://www.ncbi.nlm.nih.gov/entrez/viewer.fcgi?val=CY053379) | 1559 | Spain | 23-nov |  |  |  |  |  |
| 1195 | [CY053382](http://www.ncbi.nlm.nih.gov/entrez/viewer.fcgi?val=CY053382) | 1564 | Spain | 23-nov |  |  |  |  |  |
| 1196 | [CY053385](http://www.ncbi.nlm.nih.gov/entrez/viewer.fcgi?val=CY053385) | 1557 | Spain | 23-nov |  |  |  |  |  |
| 1197 | [CY053388](http://www.ncbi.nlm.nih.gov/entrez/viewer.fcgi?val=CY053388) | 1561 | Spain | 23-nov |  |  |  |  |  |
| 1198 | [CY053391](http://www.ncbi.nlm.nih.gov/entrez/viewer.fcgi?val=CY053391) | 1569 | Spain | 23-nov |  |  |  |  |  |
| 1199 | [CY053394](http://www.ncbi.nlm.nih.gov/entrez/viewer.fcgi?val=CY053394) | 1537 | Spain | 24-nov |  |  |  |  |  |
| 1200 | [GU371271](http://www.ncbi.nlm.nih.gov/entrez/viewer.fcgi?val=GU371271) | 1701 | China | 24-nov |  |  |  |  |  |
| 1201 | [CY052026](http://www.ncbi.nlm.nih.gov/entrez/viewer.fcgi?val=CY052026) | 1571 | Spain | 26-nov |  |  |  |  |  |
| 1202 | [GU371272](http://www.ncbi.nlm.nih.gov/entrez/viewer.fcgi?val=GU371272) | 1701 | China | 26-nov |  |  |  |  |  |
| 1203 | [CY053397](http://www.ncbi.nlm.nih.gov/entrez/viewer.fcgi?val=CY053397) | 1559 | Spain | 30-nov |  |  |  |  |  |
| 1204 | [CY053401](http://www.ncbi.nlm.nih.gov/entrez/viewer.fcgi?val=CY053401) | 1559 | Spain | 30-nov |  |  |  |  |  |
| 1205 | [CY053409](http://www.ncbi.nlm.nih.gov/entrez/viewer.fcgi?val=CY053409) | 1562 | Spain | 30-nov |  |  |  |  |  |
| 1206 | [CY053405](http://www.ncbi.nlm.nih.gov/entrez/viewer.fcgi?val=CY053405) | 1560 | Spain | 1-Dec |  |  |  |  |  |
| 1207 | [CY053706](http://www.ncbi.nlm.nih.gov/entrez/viewer.fcgi?val=CY053706) | 1562 | Spain | 2-Dec |  |  |  |  |  |
| 1208 | [CY053710](http://www.ncbi.nlm.nih.gov/entrez/viewer.fcgi?val=CY053710) | 1562 | Spain | 2-Dec |  |  |  |  |  |
| 1209 | [CY053369](http://www.ncbi.nlm.nih.gov/entrez/viewer.fcgi?val=CY053369) | 1571 | Spain | 3-Dec |  |  |  |  |  |
| 1210 | [CY053714](http://www.ncbi.nlm.nih.gov/entrez/viewer.fcgi?val=CY053714) | 1546 | Spain | 3-Dec |  |  |  |  |  |
| 1211 | [CY053718](http://www.ncbi.nlm.nih.gov/entrez/viewer.fcgi?val=CY053718) | 1567 | Spain | 7-Dec |  |  |  |  |  |
| 1212 | [CY053722](http://www.ncbi.nlm.nih.gov/entrez/viewer.fcgi?val=CY053722) | 1560 | Spain | 7-Dec |  |  |  |  |  |
| 1213 | [GU361111](http://www.ncbi.nlm.nih.gov/entrez/viewer.fcgi?val=GU361111) | 881 | China | 7-Dec |  |  |  |  |  |
| 1214 | [CY053695](http://www.ncbi.nlm.nih.gov/entrez/viewer.fcgi?val=CY053695) | 1514 | Spain | 10-Dec |  |  |  |  |  |
| 1215 | [CY053699](http://www.ncbi.nlm.nih.gov/entrez/viewer.fcgi?val=CY053699) | 1565 | Spain | 10-Dec |  |  |  |  |  |
| VV. Viral Variant | |  |  |  |  |  |  |  |  |
