# Supplementary material for: What happened after the initial global spread of pandemic human influenza virus A (H1N1)? A population genetics approach
Source: Virol J. 2010 Aug 20;7:196. doi: 10.1186/1743-422X-7-196 (PMC2936310; doi:10.1186/1743-422X-7-196)
Supplement: Additional file 2 — Population genetic indexes among paired sequences of A H1N1 obtained from different countries. List of values (indexes) obtained for population genetic analysis among paired sequences from different countries after DnaSP v4 analysis. [file 1743-422X-7-196-S2.DOC]

Supplementary table 2. Population genetic indexes among paired sequences of A H1N1 obtained from different countries

| Analysis of the sequences published in April-July 2009 | | | | | | | | | | |
| --- | --- | --- | --- | --- | --- | --- | --- | --- | --- | --- |
| Country 1 | Country 2 |  | G_ST_^a^ | |  | F_ST_^b^ | |  | Nm^c^ | |
|  |  |  | HA | NA |  | HA | NA |  | HA | NA |
| USA | Australia |  | 0.060 | 0.036 |  | 0.101 | 0.022 |  | 4.46 | ∞ |
|  | Canada |  | 0.030 | 0.308 |  | 0.065 | 0.016 |  | 7.14 | ∞ |
|  | Chile |  | 0.045 | 0.057 |  | 0.009 | -0.038 |  | ∞ | ∞ |
|  | China |  | 0.009 | 0.024 |  | 0.001 | 0.015 |  | ∞ | ∞ |
|  | France |  | 0.051 | 0.051 |  | 0.184 | 0.159 |  | 2.22 | 2.65 |
|  | Germany |  | 0.043 | 0.072 |  | -0.036 | -0.037 |  | ∞ | ∞ |
|  | Greece |  | 0.025 | 0.107 |  | 0.182 | 0.159 |  | 2.24 | 2.65 |
|  | Japan |  | 0.022 | 0.059 |  | 0.016 | 0.109 |  | ∞ | 4.09 |
|  | Mexico |  | 0.098 | 0.225 |  | 0.243 | 0.441 |  | 1.56 | 0.63 |
|  | New Zealand |  | 0.056 | 0.051 |  | 0.234 | 0.190 |  | 1.64 | 2.13 |
|  | Philippines |  | 0.057 | 0.107 |  | -0.058 | -0.198 |  | ∞ | ∞ |
|  | Russia |  | 0.049 | 0.064 |  | 0.010 | 0.030 |  | ∞ | ∞ |
|  | Spain |  | 0.033 | 0.105 |  | 0.094 | 0.485 |  | 4.80 | 0.05 |
|  | Thailand |  | 0.052 | 0.056 |  | 0.067 | 0.525 |  | 6.93 | 0.45 |
| China | Australia |  | 0.050 | 0.018 |  | 0.105 | 0.125 |  | 4.28 | 3.48 |
|  | Canada |  | 0.020 | 0.043 |  | 0.018 | 0.016 |  | ∞ | ∞ |
|  | Chile |  | 0.046 | 0.052 |  | 0.057 | 0.002 |  | 8.34 | ∞ |
|  | France |  | 0.037 | 0.037 |  | 0.113 | 0.157 |  | 3.94 | 2.69 |
|  | Germany |  | 0.015 | 0.045 |  | -0.038 | 0.012 |  | ∞ | ∞ |
|  | Italy |  | 0.069 | 0.144 |  | 0.139 | 0.140 |  | 3.09 | 3.06 |
|  | Japan |  | 0.018 | 0.114 |  | 0.008 | 0.078 |  | ∞ | 5.88 |
|  | Mexico |  | 0.041 | 0.075 |  | 0.096 | 0.316 |  | 4.70 | 1.08 |
|  | New Zealand |  | 0.065 | 0.043 |  | 0.092 | -0.001 |  | 4.92 | ∞ |
|  | Philippines |  | 0.037 | 0.088 |  | -0.042 | 0.117 |  | ∞ | 3.72 |
|  | Russia |  | 0.042 | 0.053 |  | 0.005 | 0.000 |  | ∞ | ∞ |
|  | Spain |  | 0.169 | 0.019 |  | 0.396 | 0.049 |  | 0.76 | 9.71 |
|  | Thailand |  | 0.087 | 0.066 |  | 0.472 | 0.416 |  | 0.56 | 0.70 |
|  | USA |  | 0.009 | 0.024 |  | 0.001 | 0.015 |  | ∞ | ∞ |
| Mexico | Australia |  | 0.075 | 0.621 |  | 0.337 | 0.387 |  | 0.10 | 0.79 |
|  | Canada |  | 0.000 | 0.087 |  | 0.091 | 0.461 |  | 5.01 | 0.58 |
|  | Chile |  | 0.199 | 0.075 |  | 0.372 | 0.414 |  | 0.08 | 0.71 |
|  | China |  | 0.041 | 0.075 |  | 0.096 | 0.316 |  | 4.70 | 1.08 |
|  | France |  | 0.054 | 0.077 |  | 0.124 | 0.444 |  | 3.52 | 0.63 |
|  | Germany |  | 0.177 | 0.082 |  | 0.110 | 0.377 |  | 4.03 | 0.83 |
|  | Italy |  | 0.397 | 0.125 |  | 0.434 | 0.521 |  | 0.65 | 0.46 |
|  | Japan |  | 0.038 | 0.107 |  | 0.058 | 0.417 |  | 8.10 | 0.70 |
|  | New Zealand |  | 0.024 | 0.102 |  | 0.135 | 0.521 |  | 3.20 | 0.46 |
|  | Philippines |  | 0.058 | 0.125 |  | 0.076 | 0.521 |  | 6.07 | 0.46 |
|  | Russia |  | 0.063 | 0.075 |  | 0.138 | 0.414 |  | 3.12 | 0.71 |
|  | Spain |  | 0.035 | 0.255 |  | 0.068 | 0.474 |  | 6.86 | 0.55 |
|  | Thailand |  | 0.099 | 0.061 |  | 0.563 | 0.451 |  | 0.39 | 0.61 |
|  | USA |  | 0.098 | 0.225 |  | 0.243 | 0.441 |  | 1.56 | 0.63 |
| Spain | Australia |  | 0.087 | 0.188 |  | 0.230 | 0.552 |  | 1.67 | 0.41 |
|  | Canada |  | 0.029 | 0.168 |  | 0.079 | 0.458 |  | 5.84 | 0.59 |
|  | Chile |  | 0.101 | 0.180 |  | 0.211 | 0.511 |  | 1.87 | 0.48 |
|  | China |  | 0.169 | 0.019 |  | 0.396 | 0.049 |  | 0.76 | 9.71 |
|  | France |  | 0.053 | 0.162 |  | 0.013 | 0.415 |  | ∞ | 0.07 |
|  | Germany |  | 0.049 | 0.044 |  | 0.016 | -0.033 |  | ∞ | ∞ |
|  | Italy |  | 0.140 | 0.208 |  | 0.218 | 0.802 |  | 1.80 | 0.12 |
|  | Japan |  | 0.002 | 0.223 |  | 0.016 | 0.375 |  | ∞ | 0.83 |
|  | Mexico |  | 0.035 | 0.255 |  | 0.068 | 0.474 |  | 6.86 | 0.55 |
|  | New Zealand |  | ND | ND |  | ND | ND |  | ND | ND |
|  | Philippines |  | 0.067 | 0.116 |  | -0.001 | 0.331 |  | ∞ | 1.01 |
|  | Russia |  | 0.078 | 0.180 |  | 0.085 | 0.511 |  | 5.40 | 0.48 |
|  | Thailand |  | 0.184 | 0.147 |  | 0.562 | 0.662 |  | 0.39 | 0.25 |
|  | USA |  | 0.033 | 0.105 |  | 0.094 | 0.485 |  | 4.80 | 0.05 |
| Japan | Australia |  | 0.073 | 0.090 |  | 0.207 | 0.248 |  | 1.92 | 1.52 |
|  | Canada |  | 0.034 | 0.170 |  | 0.042 | 0.098 |  | ∞ | 4.62 |
|  | Chile |  | 0.076 | 0.144 |  | 0.092 | 0.125 |  | 4.93 | 3.51 |
|  | China |  | 0.018 | 0.114 |  | 0.008 | 0.078 |  | ∞ | 5.88 |
|  | France |  | 0.028 | 0.186 |  | 0.047 | 0.198 |  | 10.10 | 2.02 |
|  | Germany |  | -0.006 | 0.096 |  | -0.044 | 0.029 |  | ∞ | ∞ |
|  | Italy |  | 0.014 | 0.213 |  | 0.162 | 0.442 |  | 2.59 | 0.63 |
|  | Mexico |  | 0.038 | 0.107 |  | 0.058 | 0.417 |  | 8.10 | 0.70 |
|  | New Zealand |  | 0.080 | 0.169 |  | 0.087 | 0.179 |  | 5.22 | 2.29 |
|  | Philippines |  | 0.025 | 0.120 |  | -0.046 | 0.201 |  | ∞ | 1.99 |
|  | Russia |  | 0.042 | 0.124 |  | 0.028 | 0.101 |  | ∞ | 4.47 |
|  | Spain |  | 0.002 | 0.223 |  | 0.016 | 0.375 |  | ∞ | 0.83 |
|  | Thailand |  | 0.107 | 0.134 |  | 0.456 | 0.595 |  | 0.06 | 0.34 |
|  | USA |  | 0.022 | 0.059 |  | 0.016 | 0.109 |  | ∞ | 4.09 |
| Global analysis | | | | | | | | | | |
| USA | Argentina |  | 0,017 | 0,027 |  | 0,054 | 0,053 |  | 8,83 | 8,86 |
|  | Australia |  | 0,017 | 0,039 |  | 0,052 | 0,047 |  | 9,18 | 10,08 |
|  | Brazil |  | 0,008 | 0,058 |  | 0,046 | 0,052 |  | 10,46 | 9,10 |
|  | Canada |  | 0,009 | 0,007 |  | 0,046 | 0,051 |  | 10,46 | 9,24 |
|  | Chile |  | 0,022 | 0,059 |  | 0,054 | 0,041 |  | 8,82 | 11,83 |
|  | China |  | 0,008 | 0,009 |  | 0,448 | 0,045 |  | 10,66 | 10,52 |
|  | Denmark |  | 0,059 | 0,058 |  | 0,046 | 0,043 |  | 10,47 | 11,26 |
|  | Dominican Republic |  | 0,023 | 0,024 |  | 0,143 | 0,111 |  | 2,99 | 4,00 |
|  | Ecuador |  | 0,047 | ND |  | 0,042 | ND |  | 11,54 | ND |
|  | Finland |  | 0,058 | 0,076 |  | 0,041 | 0,052 |  | 11,58 | 9,05 |
|  | France |  | 0,017 | 0,022 |  | 0,041 | 0,052 |  | 11,56 | 9,16 |
|  | Germany |  | 0,034 | 0,034 |  | 0,045 | 0,066 |  | 10,37 | 7,07 |
|  | Iran |  | 0,076 | 0,077 |  | 0,050 | 0,054 |  | 9,44 | 9,43 |
|  | Israel |  | 0,059 | 0,077 |  | 0,053 | 0,032 |  | 8,97 | 15,31 |
|  | Italy |  | 0,010 | 0,013 |  | 0,048 | 0,060 |  | 10,14 | 7,83 |
|  | Japan |  | 0,007 | 0,007 |  | 0,048 | 0,047 |  | 10,01 | 10,05 |
|  | Mexico |  | 0,019 | 0,005 |  | 0,048 | 0,049 |  | 10,03 | 9,80 |
|  | Mongolia |  | 0,058 | ND |  | 0,070 | ND |  | 6,66 | ND |
|  | Myanmar |  | 0,059 | 0,059 |  | 0,053 | 0,058 |  | 8,87 | 8,19 |
|  | New Zealand |  | 0,037 | 0,027 |  | 0,047 | 0,049 |  | 10,20 | 9,78 |
|  | Norway |  | 0,010 | 0,034 |  | 0,047 | 0,047 |  | 10,09 | 10,25 |
|  | Philippines |  | 0,058 | 0,039 |  | 0,039 | 0,487 |  | 12,23 | 0,53 |
|  | Poland |  | ND | 0,021 |  | ND | 0,060 |  | ND | 7,77 |
|  | Russia |  | 0,008 | 0,012 |  | 0,137 | 0,117 |  | 3,14 | 3,79 |
|  | Singapore |  | 0,006 | 0,024 |  | 0,045 | 0,058 |  | 10,72 | 8,15 |
|  | Spain |  | 0,006 | 0,030 |  | 0,043 | 0,052 |  | 11,01 | 9,13 |
|  | Switzerland |  | 0,047 | 0,015 |  | 0,039 | 0,049 |  | 12,42 | 9,71 |
|  | Taiwan |  | 0,018 | 0,020 |  | 0,049 | 0,058 |  | 9,67 | 8,08 |
|  | Thailand |  | 0,021 | 0,016 |  | 0,241 | 0,171 |  | 1,58 | 2,43 |
|  | Turkey |  | 0,013 | ND |  | 0,051 | ND |  | 9,37 | ND |
| Spain | Australia |  | 0,020 | 0,045 |  | 0,059 | 0,060 |  | 7,94 | 7,80 |
|  | Brazil |  | 0,013 | ND |  | 0,076 | ND |  | 6,09 | ND |
|  | Canada |  | 0,046 | ND |  | 0,141 | ND |  | 3,04 | ND |
|  | Chile |  | 0,026 | 0,013 |  | 0,049 | 0,075 |  | 9,73 | 6,21 |
|  | China |  | 0,012 | 0,114 |  | 0,063 | 0,066 |  | 7,45 | 7,12 |
|  | Denmark |  | 0,058 | ND |  | 0,067 | ND |  | 6,91 | ND |
|  | Dominican Republic |  | 0,039 | 0,026 |  | 0,397 | 0,277 |  | 0,08 | 1,31 |
|  | Ecuador |  | 0,044 | ND |  | 0,099 | ND |  | 4,54 | ND |
|  | Finland |  | 0,054 | ND |  | 0,031 | ND |  | 15,84 | ND |
|  | France |  | 0,015 | 0,260 |  | 0,062 | 0,089 |  | 7,61 | 5,10 |
|  | Germany |  | 0,032 | 0,040 |  | 0,097 | 0,160 |  | 4,68 | 2,63 |
|  | Iran |  | 0,076 | ND |  | 0,067 | ND |  | 6,93 | ND |
|  | Israel |  | 0,058 | ND |  | 0,182 | ND |  | 2,24 | ND |
|  | Italy |  | 0,021 | 0,016 |  | 0,029 | 0,046 |  | 16,84 | 10,48 |
|  | Japan |  | 0,013 | 0,016 |  | 0,074 | 0,066 |  | 6,28 | 7,07 |
|  | Mexico |  | 0,058 | 0,036 |  | 0,160 | 0,094 |  | 2,63 | 4,79 |
|  | Mongolia |  | 0,060 | ND |  | 0,153 | ND |  | 2,76 | ND |
|  | Myanmar |  | 0,057 | ND |  | 0,059 | ND |  | 7,93 | ND |
|  | New Zealand |  | 0,042 | 0,035 |  | 0,197 | 0,072 |  | 2,03 | 6,44 |
|  | Norway |  | 0,006 | ND |  | 0,024 | ND |  | 20,49 | ND |
|  | Philippines |  | 0,044 | 0,038 |  | 0,099 | 0,574 |  | 5,54 | 0,37 |
|  | Poland |  | ND | 0,025 |  | ND | 0,095 |  | ND | 4,74 |
|  | Russia |  | 0,007 | 0,012 |  | 0,231 | 0,216 |  | 1,66 | 1,79 |
|  | Singapore |  | 0,006 | 0,099 |  | 0,012 | 0,125 |  | 40,09 | 3,49 |
|  | Switzerland |  | 0,044 | 0,025 |  | 0,022 | 0,046 |  | 22,44 | 10,32 |
|  | Taiwan |  | 0,023 | 0,042 |  | 0,055 | 0,132 |  | 8,55 | 3,29 |
|  | Thailand |  | 0,021 | 0,018 |  | 0,347 | 0,277 |  | 0,94 | 1,30 |
|  | Turkey |  | 0,013 | ND |  | 0,028 | ND |  | 17,52 | ND |
|  | USA |  | 0,006 | 0,030 |  | 0,043 | 0,052 |  | 11,01 | 9,13 |
| Japan | Argentina |  | 0,035 | 0,235 |  | 0,148 | 0,060 |  | 2,88 | 7,81 |
|  | Australia |  | 0,020 | 0,039 |  | 0,145 | 0,142 |  | 2,94 | 3,02 |
|  | Brazil |  | 0,013 | ND |  | 0,117 | ND |  | 3,79 | ND |
|  | Canada |  | 0,015 | 0,015 |  | 0,175 | 0,083 |  | 2,35 | 5,50 |
|  | Chile |  | 0,033 | ND |  | 0,115 | ND |  | 3,87 | ND |
|  | China |  | 0,016 | 0,014 |  | 0,087 | 0,036 |  | 5,24 | 13,49 |
|  | Denmark |  | 0,058 | ND |  | 0,102 | ND |  | 4,42 | ND |
|  | Dominican Republic |  | 0,033 | 0,048 |  | 0,468 | 0,453 |  | 0,57 | 0,60 |
|  | Ecuador |  | 0,043 | ND |  | 0,100 | ND |  | 4,50 | ND |
|  | Finland |  | 0,054 | ND |  | 0,064 | ND |  | 7,34 | ND |
|  | France |  | 0,015 | 0,033 |  | 0,096 | 0,072 |  | 4,72 | 6,40 |
|  | Germany |  | 0,035 | 0,035 |  | 0,175 | 0,176 |  | 2,36 | 2,34 |
|  | Iran |  | 0,071 | ND |  | 0,129 | ND |  | 3,37 | ND |
|  | Israel |  | 0,060 | ND |  | 0,245 | ND |  | 1,54 | ND |
|  | Italy |  | 0,031 | 0,030 |  | 0,076 | 0,116 |  | 6,04 | 3,82 |
|  | Mexico |  | 0,073 | 0,005 |  | 0,183 | 0,023 |  | 2,23 | 21,04 |
|  | Mongolia |  | 0,054 | ND |  | 0,225 | ND |  | 1,73 | ND |
|  | Myanmar |  | 0,060 | ND |  | 0,177 | ND |  | 2,32 | ND |
|  | New Zealand |  | 0,056 | 0,035 |  | 0,238 | 0,026 |  | 1,60 | 18,80 |
|  | Norway |  | 0,012 | 0,033 |  | 0,057 | -0,010 |  | 8,25 | ∞ |
|  | Philippines |  | 0,053 | 0,038 |  | 0,060 | 0,581 |  | 7,81 | 0,36 |
|  | Poland |  | ND | 0,032 |  | ND | 0,134 |  | ND | 3,24 |
|  | Russia |  | 0,012 | 0,025 |  | 0,242 | 0,225 |  | 1,56 | 1,72 |
|  | Singapore |  | 0,054 | 0,099 |  | 0,114 | 0,101 |  | 3,87 | 4,43 |
|  | Spain |  | 0,013 | 0,016 |  | 0,074 | 0,066 |  | 6,28 | 7,07 |
|  | Switzerland |  | 0,042 | 0,036 |  | 0,033 | 0,024 |  | 14,80 | 20,78 |
|  | Taiwan |  | 0,025 | 0,058 |  | 0,106 | 0,106 |  | 4,21 | 4,20 |
|  | Thailand |  | 0,020 | 0,015 |  | 0,357 | 0,280 |  | 0,90 | 1,29 |
|  | Turkey |  | 0,016 | ND |  | 0,092 | ND |  | 4,93 | ND |
|  | USA |  | 0,007 | 0,007 |  | 0,048 | 0,047 |  | 10,01 | 10,05 |
| Singapore | Argentina |  | 0,007 | 0,046 |  | 0,012 | 0,115 |  | 40,13 | 3,85 |
|  | Australia |  | 0,015 | 0,033 |  | 0,032 | -0,002 |  | 15,17 | ∞ |
|  | Brazil |  | 0,027 | ND |  | 0,055 | ND |  | 8,54 | ND |
|  | Canada |  | 0,057 | 0,103 |  | 0,162 | 0,256 |  | 2,59 | 1,46 |
|  | Chile |  | 0,012 | ND |  | 0,013 | ND |  | 39,36 | ND |
|  | China |  | 0,071 | 0,125 |  | 0,065 | 0,143 |  | 7,15 | 3,00 |
|  | Denmark |  | 0,073 | ND |  | 0,095 | ND |  | 4,74 | ND |
|  | Dominican Republic |  | 0,067 | 0,140 |  | 0,518 | 0,662 |  | 0,47 | 0,26 |
|  | Ecuador |  | 0,049 | ND |  | ND | ND |  | ND | ND |
|  | Finland |  | 0,064 | ND |  | ND | ND |  | ND | ND |
|  | France |  | 0,036 | 0,058 |  | 0,101 | 0,292 |  | 4,47 | 1,21 |
|  | Germany |  | 0,049 | 0,062 |  | 0,252 | 0,388 |  | 1,48 | 0,79 |
|  | Iran |  | 0,068 | ND |  | 0,004 | ND |  | 121,38 | ND |
|  | Isarel |  | 0,061 | ND |  | 0,230 | ND |  | 1,68 | ND |
|  | Italy |  | 0,002 | 0,108 |  | 0,013 | 0,116 |  | 36,61 | 3,82 |
|  | Japan |  | 0,054 | 0,099 |  | 0,114 | 0,101 |  | 3,87 | 4,43 |
|  | Mexico |  | 0,007 | 0,039 |  | 0,011 | 0,025 |  | 44,60 | 19,70 |
|  | Mongolia |  | 0,065 | ND |  | 0,341 | ND |  | 0,97 | ND |
|  | Myanmar |  | 0,073 | ND |  | 0,095 | ND |  | 4,74 | ND |
|  | New Zealand |  | 0,024 | 0,025 |  | 0,002 | 0,106 |  | 276,89 | 4,22 |
|  | Norway |  | 0,044 | 0,082 |  | 0,039 | 0,086 |  | 12,17 | 5,31 |
|  | Philippines |  | 0,048 | 0,066 |  | 0,079 | 0,583 |  | 5,80 | 0,35 |
|  | Poland |  | ND | 0,049 |  | ND | 0,116 |  | ND | 3,80 |
|  | Russia |  | 0,025 | 0,135 |  | 0,247 | 0,226 |  | 1,53 | 1,70 |
|  | Spain |  | 0,006 | 0,099 |  | 0,012 | 0,125 |  | 40,09 | 3,49 |
|  | Switzerland |  | 0,047 | 0,020 |  | 0,006 | 0,034 |  | 84,16 | 14,18 |
|  | Taiwan |  | 0,046 | 0,006 |  | 0,073 | 0,015 |  | 6,31 | 31,92 |
|  | Thailand |  | 0,050 | 0,072 |  | 0,360 | 0,283 |  | 0,89 | 1,27 |
|  | Turkey |  | 0,051 | ND |  | 0,027 | ND |  | 18,35 | ND |
|  | USA |  | 0,006 | 0,024 |  | 0,045 | 0,058 |  | 10,72 | 8,15 |
| Mexico | Argentina |  | 0,139 | 0,019 |  | 0,416 | 0,076 |  | 0,70 | 6,12 |
|  | Australia |  | 0,101 | 0,027 |  | 0,354 | 0,017 |  | 0,91 | 28,96 |
|  | Brazil |  | 0,097 | ND |  | 0,175 | ND |  | 2,36 | ND |
|  | Canada |  | -0,001 | 0,012 |  | 0,003 | 0,028 |  | 196,87 | 17,20 |
|  | Chile |  | 0,130 | 0,050 |  | 0,400 | 0,011 |  | 0,75 | 46,07 |
|  | China |  | 0,071 | 0,025 |  | 0,197 | 0,043 |  | 2,04 | 11,12 |
|  | Denmark |  | 0,067 | ND |  | 0,262 | ND |  | 1,41 | ND |
|  | Dominican Republic |  | 0,132 | 0,050 |  | 0,655 | 0,226 |  | 0,26 | 1,71 |
|  | Ecuador |  | 0,051 | ND |  | ND | ND |  | ND | ND |
|  | Finland |  | 0,052 | ND |  | 0,043 | ND |  | 11,17 | ND |
|  | France |  | 0,063 | 0,026 |  | 0,032 | 0,012 |  | 15,02 | 39,61 |
|  | Germany |  | 0,023 | 0,027 |  | 0,037 | 0,048 |  | 13,13 | 9,92 |
|  | Iran |  | 0,099 | ND |  | 0,451 | ND |  | 0,61 | ND |
|  | Israel |  | 0,068 | ND |  | 0,131 | ND |  | 3,32 | ND |
|  | Italy |  | 0,139 | 0,039 |  | 0,237 | 0,102 |  | 1,61 | 4,40 |
|  | Japan |  | 0,073 | 0,005 |  | 0,183 | 0,023 |  | 2,23 | 21,04 |
|  | Mongolia |  | 0,076 | ND |  | 0,378 | ND |  | 0,82 | ND |
|  | Myanmar |  | 0,081 | ND |  | 0,366 | ND |  | 0,87 | ND |
|  | New Zealand |  | 0,022 | ND |  | 0,015 | ND |  | 32,06 | ND |
|  | Norway |  | 0,111 | 0,026 |  | 0,231 | 0,049 |  | 1,66 | 9,78 |
|  | Philippines |  | 0,072 | 0,033 |  | 0,023 | 0,581 |  | 20,87 | 0,36 |
|  | Poland |  | ND | 0,022 |  | ND | 0,095 |  | ND | 4,75 |
|  | Russia |  | 0,108 | 0,040 |  | 0,245 | 0,227 |  | 1,54 | 1,70 |
|  | Singapore |  | 0,007 | 0,039 |  | 0,011 | 0,025 |  | 44,60 | 19,70 |
|  | Spain |  | 0,058 | 0,036 |  | 0,160 | 0,094 |  | 2,63 | 4,79 |
|  | Switzerland |  | 0,067 | 0,026 |  | 0,069 | 0,026 |  | 6,72 | 18,45 |
|  | Taiwan |  | 0,104 | 0,027 |  | 0,311 | 0,025 |  | 1,11 | 19,59 |
|  | Thailand |  | 0,081 | 0,016 |  | 0,360 | 0,281 |  | 0,89 | 1,28 |
|  | Turkey |  | 0,092 | ND |  | 0,251 | ND |  | 1,49 | ND |
|  | USA |  | 0,019 | 0,005 |  | 0,048 | 0,049 |  | 10,03 | 9,80 |
| China | Argentina |  | 0,051 | 0,024 |  | 0,084 | 0,079 |  | 5,42 | 5,81 |
|  | Australia |  | 0,033 | 0,024 |  | 0,079 | 0,127 |  | 5,81 | 3,42 |
|  | Brazil |  | 0,020 | ND |  | 0,090 | ND |  | 5,04 | ND |
|  | Canada |  | 0,016 | ND |  | 0,149 | ND |  | 2,86 | ND |
|  | Chile |  | 0,050 | ND |  | 0,085 | ND |  | 5,36 | ND |
|  | Denmark |  | 0,577 | ND |  | 0,058 | ND |  | 8,17 | ND |
|  | Dominican Republic |  | 0,035 | 0,043 |  | 0,461 | 0,272 |  | 0,59 | 1,34 |
|  | Ecuador |  | 0,038 | ND |  | 0,121 | ND |  | 3,64 | ND |
|  | Finland |  | 0,051 | ND |  | 0,033 | ND |  | 14,77 | ND |
|  | France |  | 0,013 | 0,040 |  | 0,073 | 0,036 |  | 6,37 | 13,56 |
|  | Germany |  | 0,035 | 0,035 |  | 0,143 | 0,103 |  | 3,00 | 4,36 |
|  | Iran |  | 0,069 | ND |  | 0,075 | ND |  | 6,14 | ND |
|  | Israel |  | 0,059 | ND |  | 0,216 | ND |  | 1,81 | ND |
|  | Italy |  | 0,046 | ND |  | 0,061 | ND |  | 7,67 | ND |
|  | Japan |  | 0,016 | 0,014 |  | 0,087 | 0,036 |  | 5,24 | 13,49 |
|  | Mexico |  | 0,071 | 0,025 |  | 0,197 | 0,043 |  | 2,04 | 11,12 |
|  | Mongolia |  | 0,051 | ND |  | 0,201 | ND |  | 1,99 | ND |
|  | Myanmar |  | 0,059 | ND |  | 0,123 | ND |  | 3,55 | ND |
|  | New Zealand |  | 0,049 | 0,045 |  | 0,188 | 0,019 |  | 2,16 | 26,35 |
|  | Norway |  | 0,008 | 0,025 |  | 0,062 | 0,011 |  | 7,51 | 45,29 |
|  | Philippines |  | 0,050 | 0,029 |  | 0,010 | 0,579 |  | 48,21 | 0,36 |
|  | Poland |  | ND | 0,044 |  | ND | 0,131 |  | ND | 3,32 |
|  | Russia |  | 0,019 | 0,007 |  | 0,240 | 0,218 |  | 1,58 | 1,80 |
|  | Singapore |  | 0,071 | 0,125 |  | 0,065 | 0,143 |  | 7,15 | 3,00 |
|  | Spain |  | 0,012 | 0,114 |  | 0,063 | 0,066 |  | 7,45 | 7,12 |
|  | Switzerland |  | 0,040 | 0,054 |  | 0,033 | 0,041 |  | 14,74 | 11,78 |
|  | Taiwan |  | 0,027 | 0,095 |  | 0,080 | 0,112 |  | 5,76 | 3,97 |
|  | Thailand |  | 0,017 | 0,011 |  | 0,355 | 0,278 |  | 0,91 | 1,30 |
|  | Turkey |  | 0,013 | ND |  | 0,079 | ND |  | 5,80 | ND |
|  | USA |  | 0,008 | 0,009 |  | 0,448 | 0,045 |  | 10,66 | 10,52 |

^a^G_ST_, genetic differentiation index; ^b^Fst, coancestry coefficient; ^d^Nm, migration index;

∞, populations with constant gene flow; ND, not determined
